# Molecular Basis for Activation to Inhibition Switching in Kv7.2 Channel Modulators

**DOI:** 10.1002/anie.7948330

**Published:** 2026-08-22

**Authors:** Tania Ciaglia, Giusy Carleo, Zhenni Yang, Francesca Di Matteo, Federica De Rosa, Gerardina Smaldone, Zhao‐Bing Gao, Marco D'Alì, Rita Turcio, Demin Ma, Veronica Di Sarno, Nannan Su, Salvatore Vincenzo Giofrè, Giacomo Pepe, Pietro Campiglia, Alessia Bertamino, Francesco Miceli, Jiangtao Guo, Nunzio Iraci, Carmine Ostacolo, Maurizio Taglialatela

**Affiliations:** ^1^ Department of Pharmacy University of Salerno Fisciano (SA) Italy; ^2^ Department of Neuroscience, Reproductive Sciences and Dentistry Section of Pharmacology University of Naples Federico II Naples Italy; ^3^ Nanhu Brain‐Computer Interface Institute Hangzhou Zhejiang China; ^4^ Department of Biophysics and Neurology of the Fourth Affiliated Hospital School of Medicine Zhejiang University Hangzhou China; ^5^ State Key Laboratory of Drug Research Shanghai Institute of Materia Medica Chinese Academy of Sciences Pudong Shanghai China; ^6^ Department of Chemical, Biological, Pharmaceutical and Environmental Sciences (CHIBIOFARAM) University of Messina Viale Ferdinando Stagno d'Alcontres 31 Messina Italy; ^7^ Department of Neurology of the Fourth Affiliated Hospital Center For Membrane Receptors and Brain Medicine School of Medicine Zhejiang University Hangzhou China

**Keywords:** Cryo‐EM, drug discovery, electrophysiology, Kv7 channels modulators, molecular modelling

## Abstract

Kv7 voltage‐gated potassium channels play a critical role in controlling electrical properties of excitable tissues. Neuronally‐expressed Kv7 channels are involved in both common and rare neuropsychiatric disorders, ranging from epilepsy to depression and neurodegenerative diseases; thus, they represent attractive therapeutic targets. However, no Kv7 modulator is currently available for clinical use. Improved knowledge of the functional and structural determinants responsible for ligand‐induced Kv7 channel modulation is likely to fill this gap. In the present work, we describe the cryo‐electron microscopy structures of human Kv7.2 channels in complex with two retigabine analogues: compound 60 (**c60**), which we previously described as a Kv7 activator with improved pharmacokinetic and pharmacodynamic properties, and the newly designed compound 106 (**c106**), which acts as a potent Kv7 blocker. Although both compounds occupy the same pocket at the S5–S6 interface in the pore domain, docking and molecular dynamics simulations and electrophysiological experiments revealed that the opposite functional behavior is due to their differential interaction, involving the L307 residue. Thus, we herein provide novel mechanistic insights into the molecular mechanisms governing Kv7 channel modulation by exogenous ligands which may prove useful to target Kv7 channels with more potent and selective modulators.

## Graphical abstract

The paper describes the serendipitous discovery of chemical manipulation allowing the activator‐to‐inhibitor switching in Kv7.2 channel modulators. The molecular determinants driving this switch have been rationalized by multidisciplinary investigation encompassing synthetic and analytical chemistry, in silico methods, cryo‐EM analysis, electrophysiological and mutagenesis studies, providing solid clues for the rational design of Kv7.2 modulators.

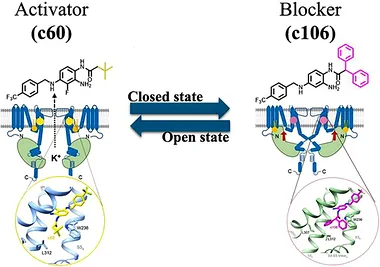

## 1 Introduction

A wide variety of K^+^ currents, each with distinct sub‐cellular localization, biophysical properties, modulation, and pharmacological profile, shape the functional properties of individual neurons at distinct developmental stages [1]. Among neuronally‐expressed voltage‐gated K^+^ channels, those formed by Kv7.2‐7.5 subunits, encoded by the KCNQ (*KCNQ2‐5*) gene subfamily, underlie the M‐current (I_KM_), a slowly‐activating and ‐deactivating, non‐inactivating K^+^‐selective current suppressed upon activation of G_q_‐coupled M_1_, M_3_, or M_5_ muscarinic receptors which regulates the resting membrane potential and suppresses repetitive neuronal firing [2, 3].

I_KM_‐mediated control of neuronal excitability in the peripheral and/or the central nervous system is altered in both common and rare neuropsychiatric disorders, including epilepsy, pain, depression, sleep disturbance and neurodegenerative diseases. In fact, variants in KCNQ2 and, more rarely, KCNQ3 and KCNQ5 genes are responsible for mostly early‐onset (neonatal) epileptic and non‐epileptic phenotypes with great clinical heterogeneity, spanning from self‐limiting forms to severe Developmental and Epileptic Encephalopathies (DEEs) [4]. In vitro functional studies have revealed that most KCNQ2 pathogenic variants promote loss‐of‐function (LoF) effects, and that a correlation exists between the extent of channel dysfunction and disease clinical severity [5, 6]. In addition to LoF, a smaller number of gain‐of‐function (GoF) variants in KCNQ2 have been reported in patients with a distinct clinical phenotype of profound neonatal encephalopathy in the absence of neonatal seizures [7, 8, 9]. GoF variants have also been described in KCNQ3 in patients with autism and electrographic status epilepticus in sleep [9, 10], and in KCNQ5 in patients with various degrees of neurodevelopmental delay, with or without epilepsy [11, 12].

In addition to rare genetically determined monogenic epilepsies, changes in the expression or function of neuronal KCNQ channels contribute to ictogenesis also in mesial temporal lobe epilepsy [13], the most prevalent form of focal epilepsy in humans [14].

Given their relevant role in blunting neuronal excitability, Kv7 channel activation represents an attractive pharmacological strategy for epilepsy and other neuropsychiatric conditions. However, two prototypic activators of neuronal Kv7 channels approved for clinical use in hyperexcitability disorders, namely the analgesic flupirtine and the anticonvulsant ezogabine (retigabine) were later withdrawn due to toxicities unrelated to their mechanism of action, but rather consequent to their chemical liability, off‐target effects, and lack of selectivity. Therefore, novel Kv7 activators with improved pharmacokinetic and pharmacodynamic properties are currently being developed, with some in advanced phases of clinical experimentation [15]. On the other hand, pharmacological inhibition of neuronal Kv7 channels has been suggested as a suitable pharmacological strategy to enhance neuronal excitability and neurotransmitter release, thus counteracting neurotransmission deficits responsible for cognitive decline in Alzheimer disease (AD) [16, 17] and motor symptoms in Parkinson's disease (PD) [18, 19]. However, clinical trials with the pan‐Kv7 inhibitor linopirdine failed to demonstrate efficacy in AD patients [20], and second‐generation Kv7 blockers (DMP‐543, XE991), though effective in animal models of PD, never advanced to clinical use [21], possibly because of their poor potency, unfavorable pharmacokinetics, lack of subtype selectivity, and off‐target cardiac effects [18]. Moreover, reducing Kv7 channel function with the tricyclic antidepressant amitriptyline [22], or with the cholinesterase inhibitor donepezil [23] has been suggested as a personalized treatment strategy for patients with KCNQ2 or KCNQ3 DEE‐causing GoF variants; however, neither drug is a selective Kv7 channel inhibitor. Subtype‐selective Kv7 blockers would also allow to investigate the role of I_KM_ in neuroplasticity and long‐term potentiation, and to validate the mechanism of action of candidate Kv7 activators [24].

Altogether, these results provide solid theoretical and experimental evidence for a critical pharmacological role of neuronal Kv7 channels as research and clinical tools; however, no compound (either as activator or as a blocker) provided of suitable pharmacokinetics and pharmacodynamic specificity and selectivity is currently available. To fill this gap, we have recently performed systematic structure‐activity and pharmacological studies to identify and characterize novel series of retigabine analogues with improved potency as Kv7 activators and better pharmacokinetic properties over the parent compound [25, 26]. These efforts have recently led to the identification of compound 60 (**c60**), which carries a substitution at the benzene‐1,2,4‐triamine core scaffold, as a chemically‐stable, highly potent and effective Kv7 channel activator exhibiting stronger anticonvulsant effects in an in vivo acute seizure model when compared to retigabine [26].

Preliminary results from modelling and mutagenesis experiments performed using the cryo‐EM structures of retigabine‐bound Kv7.2 [27] suggested that the newly‐introduced 2,2‐dimethylbuthyl group of **c60** accommodates into a large lipophilic cavity positioned at the S5–S6 interface of the pore domain (PD), a region undergoing critical structural rearrangements during channel opening [28]. In the present work, we report the cryo‐EM structure of Kv7.2 in complex with **c60**; in addition, we describe the results of a novel structure‐activity campaign aimed at exploring the bottom part of this pore cavity by a novel series of analogues in which bulkier substituents were introduced at the amide nitrogen of retigabine. Our results led to the identification of compound 106 (**c106**) acting as a subtype‐selective Kv7 blocker with nanomolar potency; comparison of the cryo‐EM structure of Kv7.2 in complex with **c60** and that with **c106**, together with additional modelling and mutagenesis results, revealed a critical role for a pivotal leucine residue (L307) in the opposite functional consequences prompted by **c60** and **c106**. These results, besides providing structural and functional insights into Kv7 channel gating, expand the arsenal of pharmacological tools available to activate and inhibit Kv7 channels, which may prove essential to guide the future design of Kv7 modulators with enhanced selectivity and specificity.

## 2 Results and Discussion

### 2.1 From Kv7.2 Activation to Inhibition by Chemical Remodelling

In our recent work, we have described a retigabine analogue (**c60**) showing a 20‐fold increase in potency as a Kv7.2/3 channel activator in vitro, no photoinduced dimer formation, higher brain/plasma ratio, longer plasma half‐life, and increased antiseizure activity in an in vivo model of seizures when compared to the parent compound [26]. Preliminary molecular docking and molecular dynamics studies, performed using the cryo‐EM structure of retigabine‐bound Kv7.2 [27], which was available at the beginning of the investigation, corroborated by mutagenesis studies, suggested that, **c60** occupies a hydrophobic cleft in the PD formed by S6 helix of one subunit (S6_I_), and the S4–S5 linker, S5, pore helix (PH), and S6 of the adjacent subunit (S4–S5 linker_II_, S5_II_, PH_II_, S6_II_). In particular, when compared to retigabine, **c60** displays a tighter binding within two hydrophobic pockets, one located toward the extracellular side occupied by the trifluoromethyl group and involving interactions with L243 in the S5_II_ and L272 in the pore helix (PH_II_), and another one accommodating the newly‐introduced 2,2‐dimethylbuthyl group positioned close to the inner pore gate and lined by residues F304 and L307 in S6_I_, L221 and V225 in S4–S5 linker_II_, L232 in S5_II_, I311 and L312 in S6_II_. Noteworthy, this latter pocket is not fully occupied by **c60**, leaving additional space available for further exploration, particularly toward the bottom of the pocket near residues L307 (S6_I_) and L312 (S6_II_) (grey sphere in Figure 1a). Noteworthy, this region undergoes a distinctive inward movement during channel gating, as revealed by recent structural data comparing the open and closed structures of Kv7.2 bound to HN37 (Figure S90) [29].

**FIGURE 1:**
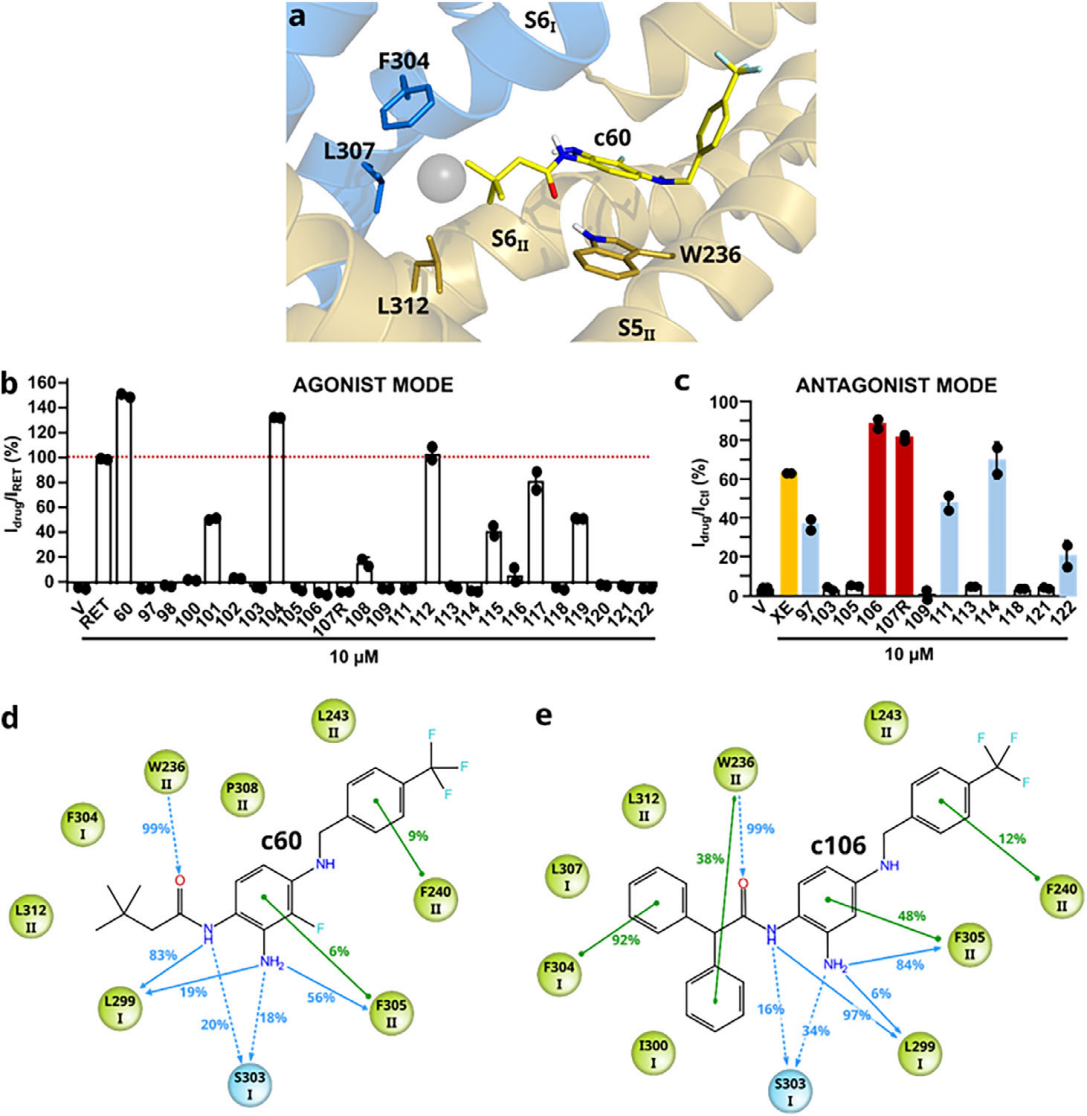
In silico and in vitro characterization of retigabine analogues carrying different substituents at the amide nitrogen. (a) Docking‐predicted bound conformation of **c60**, depicted in yellow thick solid sticks. The transparent grey sphere highlights the space available toward the bottom of the binding site, next to residues L307 in S6_I_ and L312 in S6_II_. Only two Kv7.2 subunits are shown in thick sticks and transparent cartoons and coloured in gold and marine blue. (b) Effect of the newly synthesized retigabine analogues (each at 10 µM) on Kv7.2/3 currents expressed as *I*_DRUG_/*I*_RET_ ratio at −40 mV (agonist mode). Each bar is the mean ± SEM of 2 cells (n), as indicated by the individual dots; *N* = 1. (c) Effect of the newly synthesized retigabine analogues (each at 10 µM) on Kv7.2/3 currents expressed as I_DRUG_/I_CTL_ ratio at +20 mV (antagonist mode). Each bar is the mean ± SEM of 2 cells (n), as indicated by the individual dots; *N* = 1. DMSO (V, black bar) and XE‐991 (XE, orange bar) are negative and positive controls, respectively. (d, e) Ligand interaction diagrams of 1200 ns‐long MD simulations of Kv7.2 in complex with **c60** (d) and **c106** (e). Only residues interacting with the ligand for at least 60 ns out of 1200 ns of MD simulation time are shown. Hydrophobic residues are shown in light green, polar ones in cyan. H‐bonds are represented by light blue arrows (dashed when side chain atoms are involved); green solid lines represent π–π interactions.

To allow chemical exploration of this lower pocket, a small library of 24 derivatives was designed to introduce, at the amide nitrogen, chemical moieties able to bind deeper within the pocket lined by residues F304 and L307 in S6_I_, and L312 in S6_II_. Following chemical synthesis (described in the SI file), the ability of these compounds to modulate Kv7.2/3 channels was evaluated by automated patch‐clamp using a commercially‐available provider (Ion Channel Profiler hKv7.2/hKv7.3 Agonist Qube Assay at Eurofins; https://emea.eurofinsdiscovery.com/catalog/hkv7‐2‐7‐3‐potassium‐channel‐agonist‐qube‐apc‐1‐conc/CYL8059QB; Study code: US034‐0018912). In this assay, CHO cells stably expressing Kv7.2/3 subunits were held at −120 mV and depolarized at −40 mV for 1 sec, followed by a return to −120 mV. Each compound was tested at a single concentration (10 µM) for its ability to increase the peak current amplitude measured at −40 mV, a membrane potential value very close to the current activation threshold and where most channels have low open probabilities (*agonist mode*). The results obtained revealed that compound **c60** was 50% more effective than retigabine (Table S1), as previously described [26]. Among newly synthesized compounds, seven were able to enhance Kv7.2/3 currents (**c101, c104, c108, c112, c115, c117, and c119)**, although their efficacy was lower than that of **c60** used as reference compound (Figure 1b). Instead, most compounds within the library failed to increase Kv7 currents, suggesting that they were either inactive or acted as potential inhibitors. To discriminate between these two possibilities, a second screening was performed on twelve selected compounds, all showing *I*_drug_/*I*_RET_ between +10% and −10% in the “*agonist mode*” assay (**c97, c103, c105, c106, c107R, c109, c111, c113, c114, c118, c121, c122**; each at 10 µM). In this second assay, the ability of the selected molecules to block the currents activated by a depolarizing pulse at +20 mV, a membrane potential value where channels have the highest open probability and are mostly open (*antagonist mode*) was assessed. In these experiments, again performed using a commercially‐available provider (Ion Channel Profiler hKv7.2/hKv7.3 Antagonist Qube Assay at Eurofins; https://emea.eurofinsdiscovery.com/catalog/hkv7‐2‐7‐3‐potassium‐channel‐antagonist‐qube‐apc‐6‐conc/CYL8059QB2DR; study codes: US034‐0025980 and US034‐0026562), the prototype Kv7 blocker XE991 was used as reference compound; in our experimental conditions, XE991 (10 µM) inhibited Kv7.2/3 currents by 63.1 ± 0.2% (*n* = 2). As shown in Figure 1c, six of the twelve compounds (**c103**, **c105**, **c109**, **c113**, **c118**, and **c121**), similarly to vehicle (DMSO), showed no inhibitory activity on the Kv7.2/3 currents, and were therefore deemed as inactive. Conversely, the other six compounds tested behaved as Kv7.2/3 current inhibitors; for four of them the extent of inhibition was similar to or lower than that of XE991 (**c97**, **c111**, **c114**, and **c122**; light‐blue histograms in Figure 1c), whereas two of them inhibited Kv7.2/3 more than XE991 (**c106** and **c107R**; light‐red histograms in Figure 1c), with compound **c106** showing the highest inhibitory activity.

Notably, the Kv7 agonist **c60** and the Kv7 blocker **c106** share common overall chemical features but differ for their substituent at the amide nitrogen, with **c60** carrying a smaller *t*‐butyl group and **c106** a larger diphenyl group. Another structural difference between these compounds is the presence of a halogen atom (F) at position 3 of the central triamino phenyl ring of **c60**, which is missing in **c106**; the role of this F will be specifically investigated in subsequent experiments (see below). Comparative in silico analyses between **c60** and **c106** were thus performed through induced fit docking (IFD) and molecular dynamics (MD) simulations; a simplified model of the Cryo‐EM structure of the Kv7.2 channel in complex with retigabine [27] (Kv7.2_RET/216‐330‐closed_—see chapter 4 of the Supporting Information file) was used as the target structure, since at the time **c106** was identified, no experimentally resolved Kv7 channel structure in complex with an inhibitor was available, thus the Kv7.2_RET/216‐330‐closed_ model provided an appropriate structural framework for the initial in silico investigation. Compound **c106,** similarly to **c60**, occupies a site between the S6 helix of one subunit (S6_I_) and the S5 and S6 helices of the adjacent Kv7.2 subunit (S5_II_—S6_II_). Both compounds form hydrogen bonds with residues L299 and S303 in S6_I_, W236 in S5_II_, and F305 in S6_II_, while the trifluoromethyl‐benzyl group interacts with residues F240 and L243 in S5_II_. Specifically, our in silico simulations predict that one of the two phenyl rings of **c106**, is accommodated within the pocket lined by residues F304 and L307 in S6_I_, and L312 in S6_II_, establishing interaction with these latter residues, while the second ring may contribute to stabilizing the first one within the pocket through a π−π stacking interaction with the side chain of W236 (S5_II_) (Figure 1d,e). In contrast, as previously described, **c60** exhibited less pronounced interactions with the F304 and L307 (S6_I_), and L312 (S6_II_) residues as the smaller size of its amide carbonyl substituent did not fully occupy the binding pocket (Figure 1d,e). Such differences in F304, L307, and L312 interactions between **c60** and **c106** were also analysed by estimating ligand/protein hydrophobic interaction frequencies throughout the MD simulations. The results obtained revealed that **c60** exhibited a 31.6% occupancy for the interaction with F304, compared to almost 95% for **c106**. No interaction with residue L307 was observed for **c60**, whereas **c106** showed approximately 5% occupancy. Regarding residue L312, **c60** exhibited a 11.5% occupancy, while **c106** reached about 60%. These results suggest that the hydrophobic tail of **c106** preferentially positions itself closer to key residues L307 and L312 than that of **c60**, and that such interactions potentially contribute to its inhibitory action on Kv7.2/3 currents.

### 2.2 Electrophysiological Characterization of the Effects of **c106** on Kv7.2/3 Currents

To further characterize the action mechanism of action of **c106,** whole‐cell manual patch‐clamp measurements were carried out in mammalian CHO cells transiently expressing Kv7.2/3 channels. Cells were held at −80 mV and depolarized from −80 to +20 mV for 1.5 s in 10 mV increments followed by an isopotential pulse to 0 mV. Kv7.2/3 channels activate around −50 mV with slow activation kinetics and a half‐activation potential (*V*_1/2_) of −30.2 ± 0.7 mV (Figure 2a). As previously reported [26], when compared to 1 µM retigabine, 1 µM **c60** induced a larger leftward gating shift in Kv7.2/3 currents; in fact, the Δ*V*_1/2_ was −15.3 ± 2.2 mV (*n* = 13; *N* = 5) for retigabine, and −55.7 ± 3.2 mV (*n* = 7, *N* = 3) for **c60** (*p* < 0.05). Qualitatively similar effects have been reported with other retigabine analogues [30].

**FIGURE 2:**
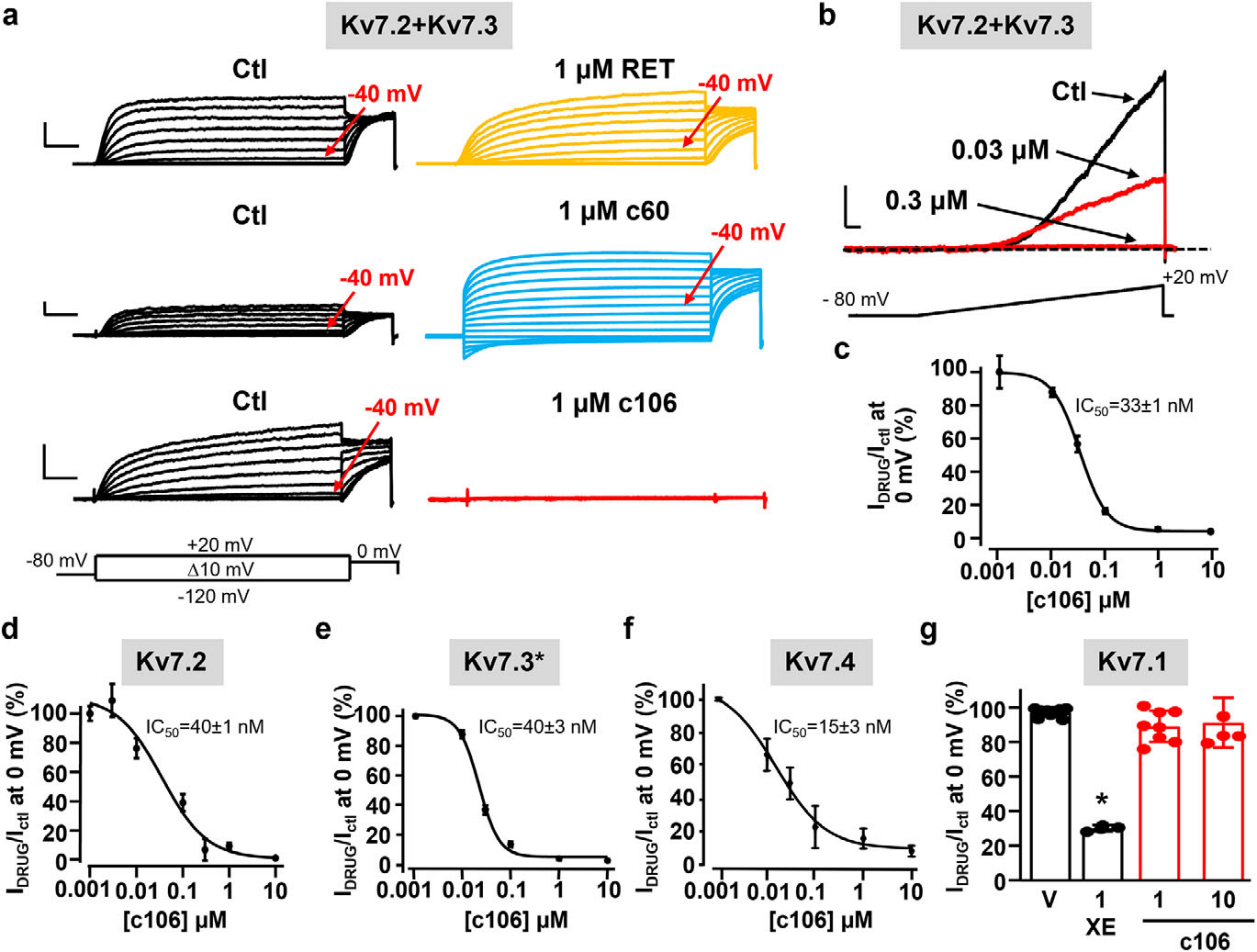
Functional characterization of **c106**, in comparison to retigabine and **c60**. (a) Representative macroscopic current traces recorded from CHO cells expressing Kv7.2/3 channels in response to the indicated voltage protocol before (left) and after (right) application of retigabine (RET), **c60** and **c106** (each at 1 µM), as indicated. Current scale, 200 pA; time scale, 200 ms. (b) Representative whole‐cell current traces from Kv7.2/3 channels activated by the indicated ramp protocol recorded in control conditions and upon exposure to 0.03 and 0.3 µM **c106**. (c–f) Concentration‐response curves for the effects of **c106** on current density (*I*_DRUG_/*I*_control_) calculated at +20 mV for Kv7.2/Kv7.3 (c), Kv7.2 (d), Kv7.3 A315T (Kv7.3*) (e) and Kv7.4 (f) channels. Solid lines represent fits of the experimental data to the four‐parameter logistic equation used to estimate EC_50_ values. Each data point is mean ± SEM. The numbers of recorded cells (n) and experimental sessions (*N*) are the following: Kv7.2 + Kv7.3 (*n* = 50, *N* = 12), Kv7.2 (*n* = 77, *N* = 10), Kv7.3* (*n* = 42, *N* = 5), Kv7.4 (*n* = 23, *N* = 4). g) Comparative effect of XE991 (XE) and **c106** (at 1 µM and 10 µM, red bars) on the Kv7.1 current density (I_DRUG_/I_control_) compared to vehicle (V). Each bar represents the mean ± SEM of 3–8 cells (n) as indicated by the individual dots (N = 3). The asterisk denotes a value significantly different (*p* < 0.05) from controls (vehicle, V).

In contrast, **c106**, when used at the same concentration (1 µM), fully blocked Kv7.2/3 currents (Figure 2a,b), confirming the previously described automated patch‐clamp data. To quantify the blocking potency of **c106**, Kv7.2/3 currents were activated using a 2 s ramp protocol from −80 to +20 mV; perfusion with increasing concentrations of **c106** (from 0.001 to 10 µM) produced a concentration‐dependent block of Kv7.2/3 channels with an IC_50_ of 33.1 ± 1 nM (Figure 2b,c). At 30 nM, **c106** inhibited roughly 50% of the current (*I*_DRUG_/*I*_CTL_ at 0 mV was 43.9% ± 6.0%), with no significant change in *V*_1/2_ (Figure S83); indeed, the current *V*_1/2_ before and after application of **c106** was −28.5 ± 1.4 and −28.7 ± 2.5, respectively (*p* > 0.05).

To assess Kv7 subtype selectivity of **c106**, its concentration‐dependent effects were also evaluated in CHO cells expressing homomeric Kv7.1, Kv7.2, Kv7.3, and Kv7.4 channels using the previously described ramp protocol. Owing to the small size of wild‐type Kv7.3 currents, which prevented accurate IC_50_ determinations, Kv7.3 A315T mutant channels (Kv7.3*) were used for these experiments. Introducing this mutation in Kv7.3 channels markedly enhanced macroscopic current size, without significantly affecting other biophysical features [31, 32]. Perfusion with increasing concentrations of **c106** (from 0.001 to 10 µM) produced a concentration‐dependent block of similar potency of the currents carried by all tested Kv7 subtypes, except Kv7.1. The calculated IC_50_ values were: 40 ± 1 nM for Kv7.2, 40 ± 3 nM for Kv7.3*, and 15 ± 3 nM for Kv7.4 channels (Figure 2d–f). Importantly while 1 µM XE991 inhibited by about 70% the currents carried by cardiac Kv7.1 channels, **c106** failed to affect Kv7.1 currents up to 10 µM (Figure 2g). These results demonstrate that **c106** effectively blocks multiple Kv7 channel subtypes with potency in the low nanomolar range, without affecting cardiac Kv7.1 channels.

### 2.3 Structural Basis for **c60** and **c106** Recognition in Human Kv7.2 Channels

Although preliminary in silico analyses described above in this study provided initial hypotheses on the molecular determinants responsible for the divergent effects of **c60**, an activator, and **c106**, a blocker, on Kv7.2 currents, experimental structural studies were required to challenge and validate these predictions. Therefore, cryo‐EM studies were performed using a truncated human Kv7.2 construct (residues A64–S674) co‐expressed with calmodulin (CaM) in HEK293S cells, as previously reported [27, 29, 33, 34]. 1 mM PIP_2_ was added during cryo‐EM sample preparation.

The 3D cryo‐EM map of Kv7.2–CaM bound to **c60** and PIP_2_ was resolved at 3.1 Å (Figures 3a,b, andS91, Table S5); both ligands are clearly detectable in the structure. Consistent with our silico studies, the binding pocket for each of the four **c60** molecules is formed by S6 of one subunit (S6_I_) and S5–S6 from a neighboring subunit (S5_II_‐S6_II_; Figure 3c). Hydrophobic interactions are formed between the drug and L299, I300, and F304 in S6_I_; F240 and L243 in S5_II_; F305, P308, and L312 in S6_II_. Moreover, the amide group of **c60** forms hydrogen bonds with S303 (S6_I_) and W236 (S5_II_) (Figure 3c) in agreement with previous mutagenesis results [26]. The shortest diagonal distances measured at G310, S314, and L318 in the **c60**‐bound Kv7.2 structure are 8.6, 9.9, and 14.7 Å, respectively consistent with an open inner gate (Figure 3d). The S4 segment adopts an activated position, with R198 exposed to the extracellular side and R210 interacting with E140 and D172 of the gating charge transfer center (Figure 3e) [27, 29, 33, 34]. Four PIP_2_ molecules are clearly resolved, with the two fatty acid chains of each PIP_2_ located in a cleft formed by S1 and S4 of one subunit and S5 of the adjacent subunits; the negatively‐charged inositol 4,5 bisphosphate head group points toward the cytosol, and interacts with a cluster of positively charged amino acid residues, including R214 in S4 and K327 in S6 (Figure 3f). Instead, in retigabine‐bound Kv7.2 structure, despite being added to the sample during preparation, no density corresponding to PIP_2_ molecules was observed in the EM map [27]. In our **c60**‐bound Kv7.2 structure, at the C‐terminal domain, the transmembrane helix S6 and the intracellular helix A (HA) form a single and continuous α‐helix, at the EKR motif (residues 330–332, Figure 3f). The C‐terminal portion of S6 is bent at the conserved PAG motif (residues 308–310), directing it away from the central pore axis and thereby opening the activation gate. Overall, the conformation closely resembles previously resolved open‐state Kv7.2 structures bound to the activators cannabidiol (CBD) (PDB 8J01, [29]), HN37 (PDB 8W4U, [29]), Ebio1 (PDB 8IJK, [34]), Ebio2 (PDB 9IXY, [33]), QO‐58 (PDB 9XB9, [35]) and QO‐83 (PDB 9XED, [35]) (Figure S94). Notably, the binding site for **c60** formed by S6_I_ and S5_II_‐S6_II_ largely overlaps that for retigabine [27], distinct from that positioned in the S3–S4 interface of the voltage‐sensing domain (VSD) and occupied by compounds such as ztz240 [27] and QO‐58 [35].

**FIGURE 3:**
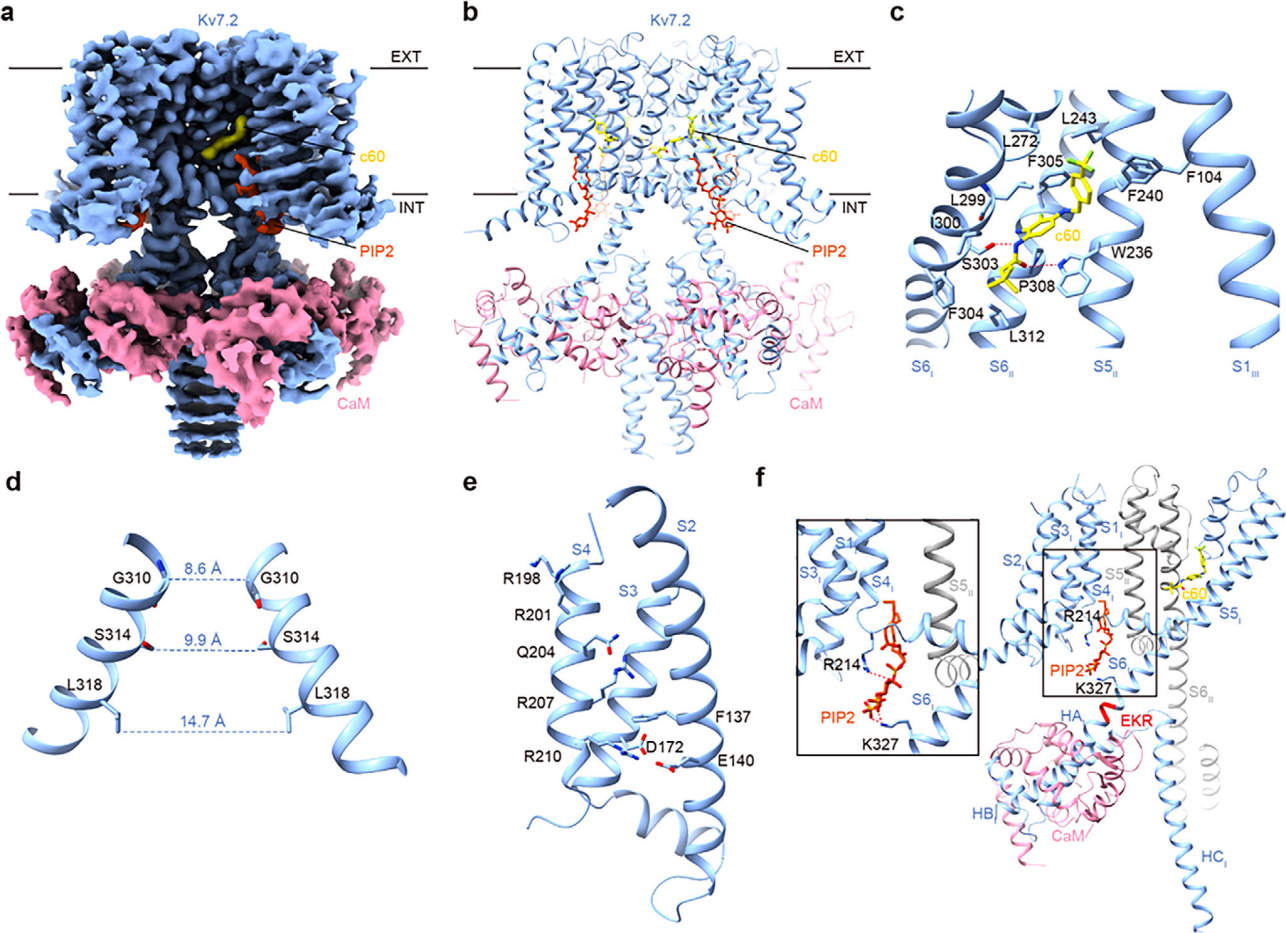
Cryo‐EM structure of the human Kv7.2–**c60** complex. (a) Cryo‐EM reconstruction of human Kv7.2 (blue) in complex with **c60** (yellow) in a side view. CaM and PIP_2_ are colored pink and orange red, respectively. (b) Overall cartoon structure model of the Kv7.2‐**c60** complex. (c) Enlarged view of the region corresponding to the **c60** binding site in the pore domain of Kv7.2. Key residues interacting with **c60** are rendered as sticks. (d) The open inner gate of Kv7.2‐**c60**. The dashed lines show diagonal atom‐to‐atom distance (in Å) at the constriction lining residues G310, S314, and L318. (e) Enlarged view of the isolated structure of Kv7.2 VSD. (f) Structural arrangement of the C‐term domain and CaM in the Kv7.2‐**c60** complex. CaM is colored in pink and the EKR motif (330‐EKR‐332) in red. The inset shows the interaction of PIP_2_ with R214 in S4 and K327 in S6.

Notably, at least in Kv7.2 [27] and Kv7.5 [36], but not in Kv7.4 [37], exogenous addition of PIP_2_ during sample preparation (either in apo or apo‐CaM state) is unable to trigger pore opening, which instead requires the presence of an exogenous ligand. However, an exogenous ligand, even in the presence of PIP_2_ is not always sufficient to detect an open pore; for example, in Kv7.2, the canonical activator retigabine, even when added together with PIP_2_ is unable to open the pore [27]; instead, similarly to **c60**, CBD [29], HN37 [29], Ebio1/2 [33, 34], as well as QO‐58 and QO‐83 [35] were able to do so.

By contrast, when identical experimental conditions were used to investigate the structural features of **c106** binding to Kv7.2 expressed with CaM and in the presence of PIP_2_, a low‐resolution (3.8 Å) map was reconstructed, where a weak density of **c106** but not PIP_2_ was observed (Figure S92, Table S5). Although at a lower resolution, the map aligns well with the closed‐conformation structure of Kv7.2 (Figure S92e). Thus, the observation of closed‐conformation structure of Kv7.2 in the presence of both the activator PIP_2_ and the blocker **c106** provides the opportunity to define the atomistic basis for **c106**‐induced channel inhibition. Moreover, CaM is positioned close to the intracellular side of the VSD, overlapping with the canonical PIP_2_ binding site [29], possibly explaining the absence of PIP_2_ density in the structure, as previously described for another Kv7.2 blocker (Ebio3) [33].

To better define the **c106** recognition mode, PIP_2_ was not included in the sample preparation and a Kv7.2‐CaM 3D structure bound to **c106** at 3.0 Å resolution was resolved (Figures 4a,b, and S93, Table S5). In this structure, four molecules of **c106** bind to four different hydrophobic grooves formed by S6_I_, together with the S5_II_ and S6_II_. Similarly, to **c60**, the trifluoromethyl‐benzyl group of **c106** interacts with residues F240 and L243 in S5_II_, whereas one of the two phenyl rings of its diphenyl acetamido tail is accommodated within the pocket lined by residues F304 and L307 in S6_I_, and L312 in S6_II_, while the second phenyl ring stabilizes the first one within the pocket. Noteworthy, the amide group of **c106** forms hydrogen bonds with S303 (S6_I_) and W236 (S5_II_) (Figure 4c).

**FIGURE 4:**
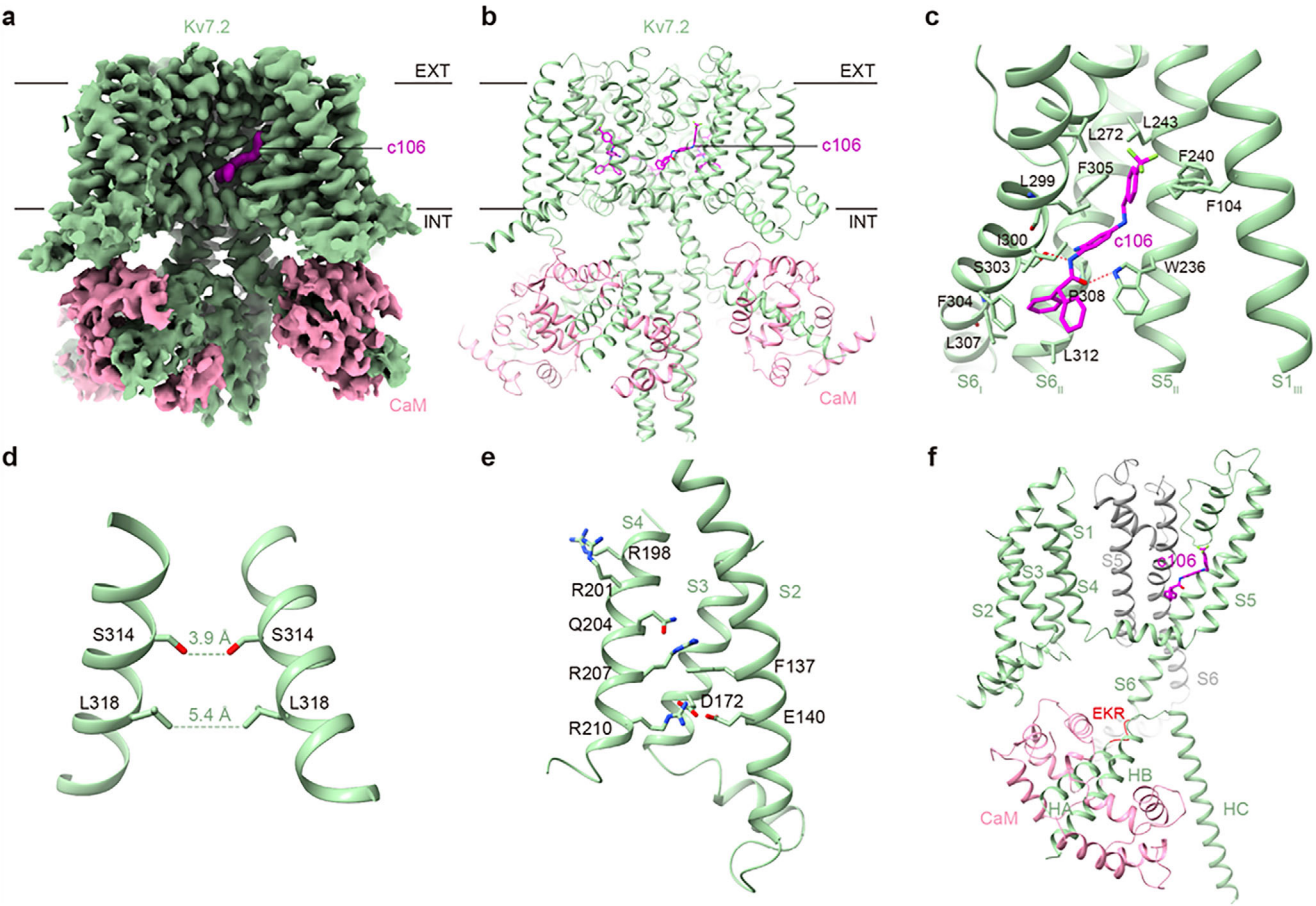
Cryo‐EM structure of the human Kv7.2–**c106** complex. (a) Cryo‐EM reconstructions of human Kv7.2 (green) in complex with **c106** (magenta) in a side view. CaM is colored in pink. (b) Overall cartoon structure model of the Kv7.2‐**c106** complex. (c) Enlarged view of the region corresponding to the binding site of **c106** in the pore domain of Kv7.2. Key residues interacting with **c106** are indicated. (d) The closed inner gate of Kv7.2‐**c106**. The dashed lines show diagonal atom‐to‐atom distance (in Å) at the constriction lining residues S314 and L318. (e) Enlarged view of the isolated structure of the VSD. (f) Structural arrangement of the C‐term domain and CaM in Kv7.2. CaM is colored in pink and the EKR motif (330‐EKR‐332) in red.

The diphenyl acetamido tail of **c106** inserts deeply into a pocket formed by F304 and L307 in S6_I_, and L312 in S6_II_, wedging the S6 helices inward; thus, the narrowest diagonal atom‐to‐atom distances at the side chains of S314 and L318 are 3.9 Å and 5.4 Å, respectively, substantially smaller than the >8 Å distances observed in open‐state Kv7.2 structures (Figure 4d) [32, 33], indicating a closed pore gate. Instead, similarly to the **c60**‐bound Kv7.2 structure, the VSD adopts an activated conformation, with R198 in S4 exposed to the extracellular side and R210 interacting with E140 and D172 of the charge transfer center (Figure 4e). Moreover, the terminal end of S6 (330‐EKR‐332) assumed a flexible loop structure and CaM was positioned close to the intracellular side of the VSD, overlapping with the canonical PIP_2_ binding site (Figure 4f) [27]. Overall, the global structure of the Kv7.2‐**c106** complex resembles the apo (closed) state of Kv7.2 (Figure S95).

### 2.4 Potential Mechanism for Kv7.2 Activation by **c60** and Inhibition by **c106**

Structural comparisons of the Kv7.2‐**c60** complex and the Kv7.2**‐c106** complexes reveal potential ligand activation and inhibition mechanisms of Kv7.2 (Figure 5). From closed to open conformations, along with the bending of S6 at the PAG motif, L312 moves upwards by ∼1 Å, with its side chain approaching the *t*‐butyl group of **c60** (Figure 5a,c). Thus, it is likely that **c60** activates Kv7.2 by hydrophobic interactions between the *t*‐butyl group and the L312 side chain, which would reduce the energy barrier required in the transition from closed to open conformations. By contrast, in the Kv7.2‐**c106** complex structure, the large diphenyl group of **c106** occupies the pocket on top of L312 and would inhibit its upwards motion, thus locking the channel in a closed conformation (Figure 5b,c). In contrast to L312 upward movement, L307, which points toward the ligand binding pocket and shows significant interaction with the diphenyl group of **c106** but not with the t‐butyl group of **c60**, does not undergo detectable movements, likely acting as an anchor for the diphenyl tail of **c106**.

**FIGURE 5:**
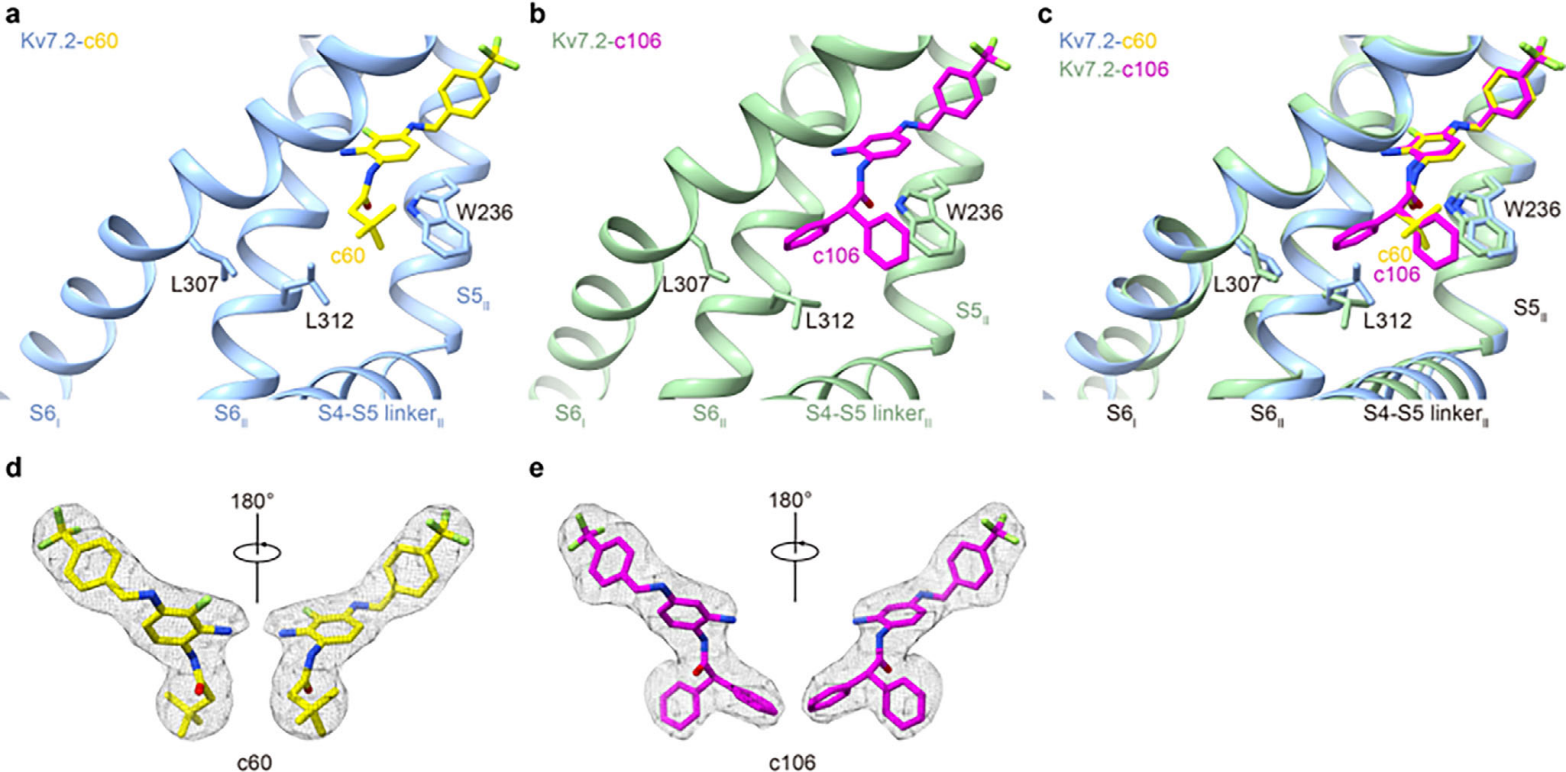
Structural comparisons of the Kv7.2‐**c60** and the Kv7.2‐**c106** complexes. (a) Enlarged view of the **c60** (yellow) binding site in Kv7.2 (blue). (b) Enlarged view of the **c106** (magenta) binding site in Kv7.2 (green). (c) Overlay of the binding sites of **c60** (yellow) and **c106** (magenta) in Kv7.2 structures showing conformational differences. Key residues L307 (S6_I_), L312 (S6_II_), and W236 (S5_II_) are rendered as sticks. (d) The density map of **c60** molecule in different orientations at the contour level of 0.007 in UCSF ChimeraX. (e) The density map of **c106** molecule in different orientations at the contour level of 0.007 in UCSF ChimeraX.

To functionally validate some of the interactions identified by the described cryo‐EM maps, electrophysiological experiments in Kv7.2 channels carrying mutations at critical residues were performed. Kv7.2 channels carrying either the W236L or the S303A mutations, which impeded **c106** hydrogen bond formation at S303 (S6_I_) and W236 (S5_II_) were completely insensitive (W236L) or significantly less sensitive (S303A) to blockade by **c106**, confirming a critical functional role for these interactions (Figure 6a,b). Noteworthy, a similar role was previously shown for these same residues (W236 and S303) in determining Kv7.2 channels activation by **c60**. Thus, despite their opposite effects on Kv7.2 channel function, both **c106** and **c60** establish critical interactions with W236 and S303. This observation implies that interactions with other regions of Kv7.2 are instead responsible for their differential pharmacological actions.

**FIGURE 6:**
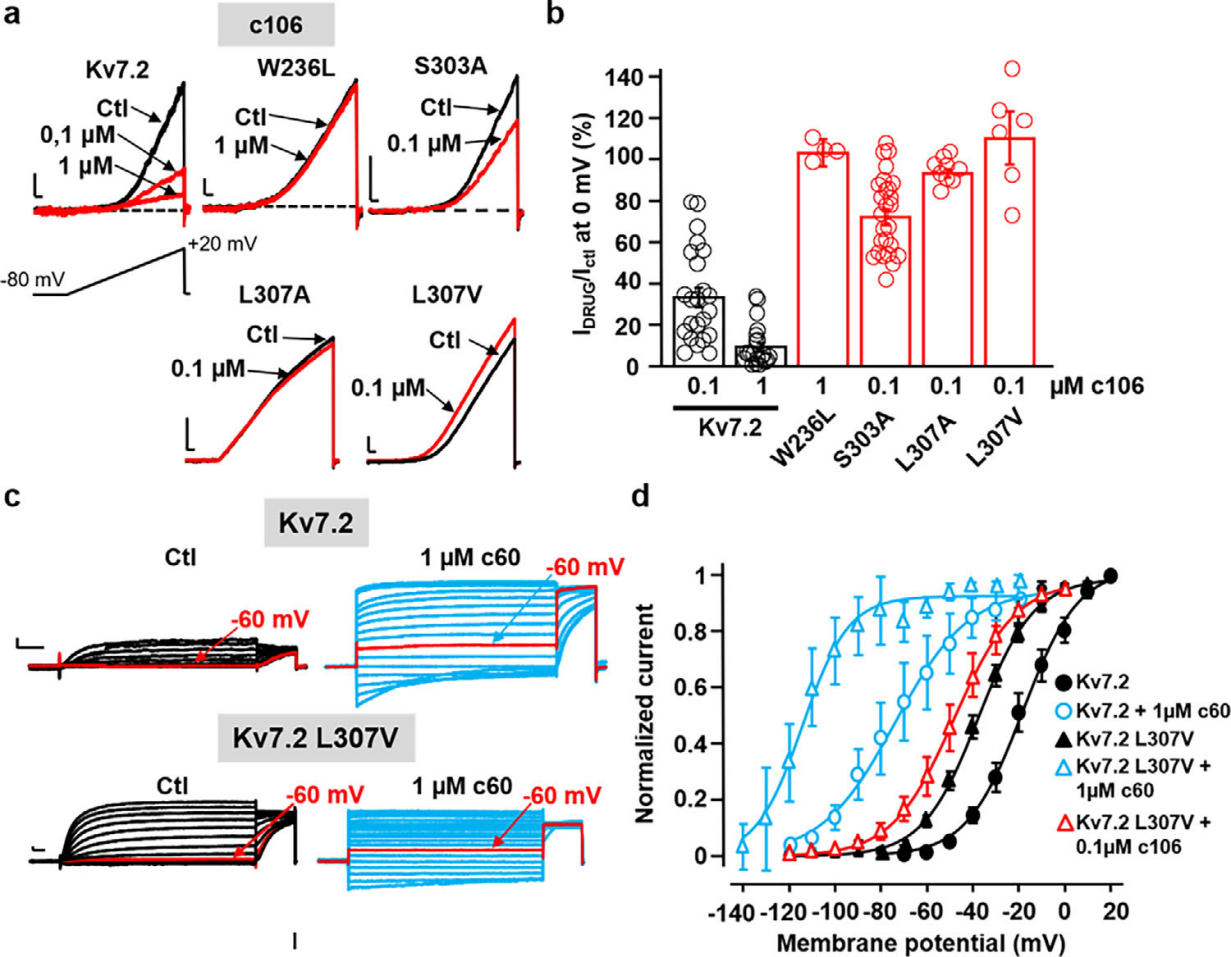
Investigating **c106** and **c60** effects on Kv7.2 currents by mutagenesis studies. (a) Representative traces of whole‐cell ramp‐evoked currents from Kv7.2, Kv7.2W236L, Kv7.2S303A, Kv7.2L307V and Kv7.2L307A channels upon perfusion with the indicated concentrations of **c106**. (b) Quantification of the effects of **c106** on the indicated channels; data are expressed as the ratio between current amplitude at 0 mV in the presence and absence of **c106** (I_DRUG_/I_CTL_). Each data point is the mean ± SEM of 4–26 cells (n), as indicated by the individual dots; *N* = 3. (c) Representative macroscopic current traces recorded from CHO cell expressing Kv7.2 or Kv7.2L307V channels in response to the indicated voltage protocol before (left) and after (right) application of 1 µM **c60**, as indicated. Current scale, 200 pA; time scale, 200 ms. (d) Normalized Kv7.2 and Kv7.2L307V conductance/voltage curves before and after perfusion with the indicated concentrations of **c60** or **c106**. Continuous lines represent fits of the experimental data to a single Boltzmann distribution. Each data point is mean ± SEM. The numbers of recorded cells (n) and experimental sessions (N) are the following: Kv7.2 (*n* = 9, *N* = 3), Kv7.2 + 1 µM **c60** (*n* = 9, *N* = 3), Kv7.2 L307V (*n* = 17, *N* = 4), Kv7.2 L307V + 1 µM **c60** (*n* = 6, *N* = 3), and Kv7.2 L307V + 0.1 µM **c106** (*n* = 12, *N* = 3).

Thus, based on our in silico and structural data, the effects of **c60** and **c106** were investigated in Kv7.2 channels carrying smaller side chain substitutions at positions 307 (L307A and L307V) and 312 (L312V). Electrophysiological experiments revealed that Kv7.2 L312V mutant channels were non‐functional (Figure S84), whereas both Kv7.2 L307A and Kv7.2 L307V channels were functional, with currents showing a leftward shift in activation gating; in fact, the activation *V*_1/2_ were −92.5 ± 4.1 mV and −36.1 ± 2.3 mV, respectively (Figure S84). Perfusion with 0.1 µM **c106**, a concentration almost fully blocking Kv7.2 channels, had no effect on the currents carried by the Kv7.2 L307A mutant, while it slightly increased Kv7.2 L307V currents (Figure 6a,b); a slight leftward shift of Kv7.2 L307V currents was in fact observed with **c106** (the Δ*V*_1/2_ was −10.1 ± 0.2 mV). These results provided strong functional evidence for a key role of the L307 residue in **c106**‐induced Kv7.2 current blockade.

To investigate the role of the L307 residue in the current activation mechanism of **c60**, we tested this compound on Kv7.2 L307V channels; the L307V was selected over the L307A variant due to its closer resemblance to Kv7.2 biophysical properties (Figure S84). Perfusion with 1 µM **c60** induced a leftward shift of the activation curve of the currents carried by both Kv7.2 and Kv7.2 L307V channels; indeed, the Δ*V*_1/2_ were −46.2 ± 5.2 and −71.9 ± 6.5 mV, respectively (Figure 6c,d). Although our modelling and structural evidence reveal that a complex network of interactions (including those with F304 in S6_I_ and L312 in S6_II_) likely explains the differential effects of **c60** and **c106** on Kv7 channel behavior, these results nevertheless suggest that, while the inhibitory action of **c106** required the presence of a bulkier leucine residue at position 307, Kv7.2 activation by **c60** was completely insensitive to such molecular change.

Notably, **c60** shares the same *t*‐butyl group at the amide nitrogen as Ebio2, a Kv7.2 channel activator featuring a distinct dihydroacenaphthylene scaffold (Figure 7a) [33]. Similar to **c60**, Ebio2 occupies the hydrophobic cleft within the pore domain (PD) located at the interface between the S6 helix of one subunit (S6_I_) and the S4–S5 linker, S5 helix, pore helix (PH), and S6 helix of the adjacent subunit (S4–S5 linker_II_, S5_II_, PH_II_, and S6_II_). Within this pocket, Ebio2 establishes extensive hydrophobic contacts with L299 and F304 in S6_I_, L221 and V225 in the S4–S5 linker_II_, W236 and F240 in S5_II_, and F305 and L312 in S6_II_, thereby closely mirroring the interaction pattern previously described for **c60**. In addition, the amide group of Ebio2 forms two hydrogen bonds involving the backbone carbonyl oxygen of L299 in S6_I_ and the indole side chain of W236 in S5_II_. By comparison, **c60** preserves the interaction with W236 and L299 and establishes an additional hydrogen bond with the hydroxyl side chain of S303 in S6_I_ through its 2‐amino substituent, a functional group absent in Ebio2 (Figure 7b). Collectively, these structural findings provide a mechanistic basis for the similar ability of the two compounds to stabilize the open state of Kv7.2 channels. Consistent with this hypothesis, mutations of W236 and S303 – two residues identified as key determinants of channel activation – markedly impair Kv7.2 activation by Ebio2, mirroring the effects observed for **c60** [33].

**FIGURE 7:**
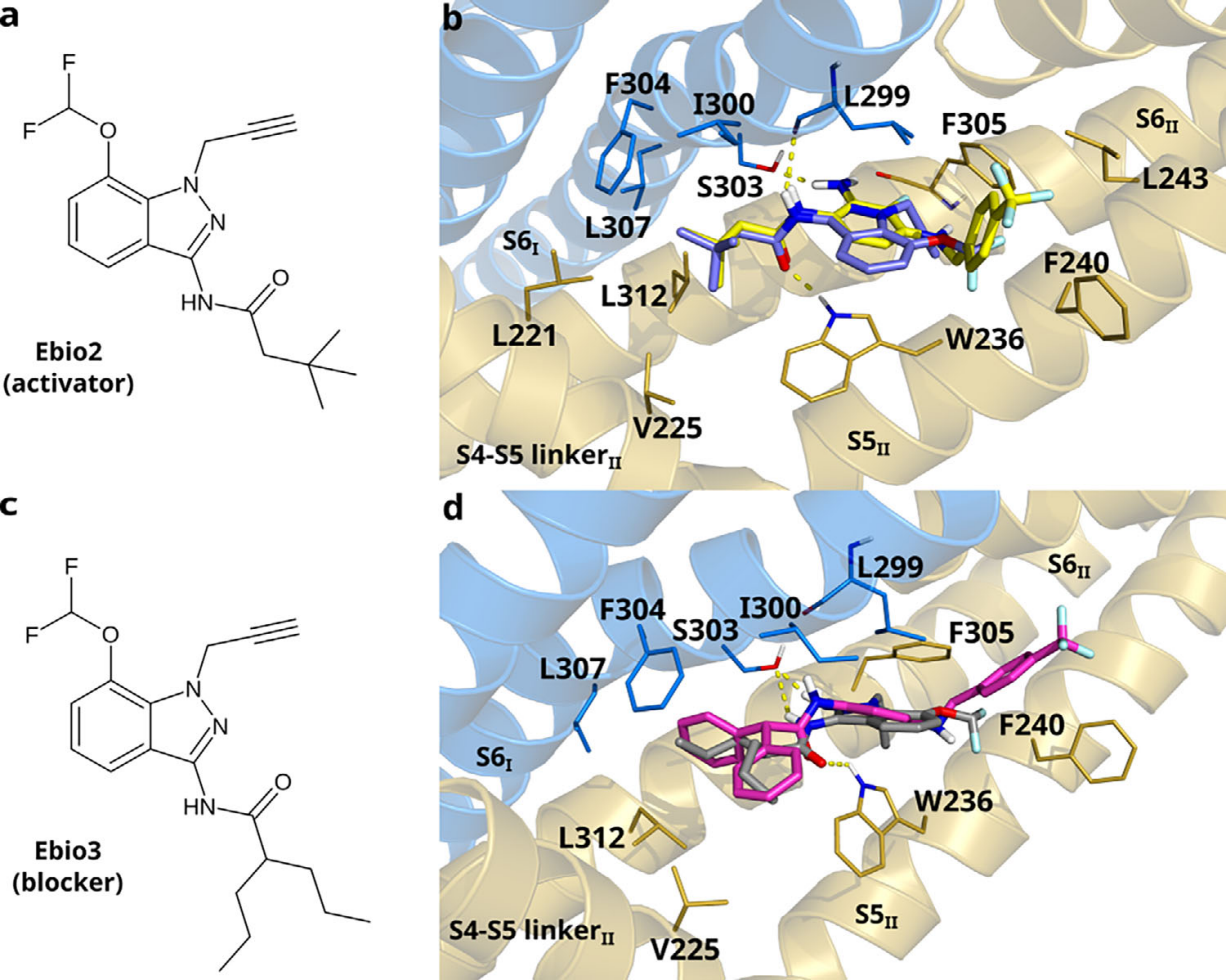
Comparisons between **c60**/Ebio2 and **c106**/Ebio3 binding modes. (a) Chemical structure of activator Ebio2. (b) Superposition of the experimental bound conformations of Ebio2 (purple) (PDB 9IXY) and **c60** (yellow), depicted as thick solid sticks. H‐bonds are represented by yellow dashes lines. The two different Kv7.2 subunits are depicted in thick sticks and transparent cartoons and colored in gold and marine blue. (c) Chemical structure of blocker Ebio3. (d) Superposition of the experimental bound conformations of Ebio3 (dark grey) (PDB 9IXZ) and **c106** (magenta), depicted as thick solid sticks. H‐bonds are represented by yellow dashes lines. The two different Kv7.2 subunits are depicted in thick sticks and transparent cartoons and colored in gold and marine blue.

Moreover, the inhibitory mechanism of **c106** closely resembles the inhibitory mechanism recently described for Kv7.2 channels by Ebio3, a potent blocker characterized by a distinct dihydroacenaphthylene scaffold and a hydrophobic dipropyl tail (Figure 7c) [33]. Similar to **c106**, Ebio3 occupies a hydrophobic pocket formed by S6_I_, S4–S5 linker_II_, S5_II_ and S6_II_, establishing hydrophobic interactions involving L299, I300, F304, and L307 in S6_I_, V225 in the S4–S5 linker_II_, W236 and F240 in S5_II_, and F305 and L312 in S6_II_ yielding a hydrophobic interaction pattern that is highly similar to that observed for **c106**. In addition, the amide moiety of Ebio3 forms hydrogen bonds with the hydroxyl side chain of S303 in S6_I_ and the indole side chain of W236 in S5_II_ (Figure 7d). These structural similarities provide a mechanistic rationale for the shared inhibitory activity of the two compounds. In particular, the dipropyl tail of Ebio3, analogous to the diphenyl moiety of **c106** is proposed to pack against the hydrophobic surface formed by F304 and L307 (S6_I_), thereby squeezing the inner pore gate through contacts with L312 in S6_II_ and ultimately stabilizing a closed channel conformation. Consistent with this hypothesis, mutation of F304 and L307 with smaller residues provided functional evidence for the proposed mechanism. Whereas the F304A mutation resulted in a non‐functional channel, the L307A and L307V variants exhibited altered gating properties in the presence of 10 µM Ebio3, highlighting the critical contribution of these residues to Ebio3‐mediated inhibition [33]. Notably, these findings resemble the effects of the L307 mutation on **c106** activity, indicating a common inhibitory mechanism shared by the two compounds. Such non‐blocking inhibitory mechanism of action of **c106** and Ebio3 is clearly distinct from that of the non‐selective Kv7 blocker linopirdine; in fact, at least in Kv7.4 channels [38], linopirdine directly blocks the ion permeation pathway by binding immediately beneath the inner pore gate. Noteworthy, despite the described similarities in the mechanisms of action between Kv7 activators (**c60** and Ebio2) and blockers (**c106** and Ebio3), photostability and anticonvulsant efficacy have only been studied for **c60**, showing its improved profile in terms of potency and efficacy over retigabine [26]. Moreover, both **c106** and Ebio3 [33], opposite to XE991 have been shown not to affect cardiac Kv7.1 channels. Thus, it is currently difficult to identify potential advantages among these Kv7 activators or blockers, and additional in vitro and in vivo tests are warranted.

The hypothesis that the high affinity blockade of Kv7.2 currents by **c106** is due to the occupancy of the L307‐containing cavity by one of the two phenyl groups was then tested by investigating the effects of both enantiomers (**R** and **S**) of a novel analogue (**c107**) in which an asymmetric and chiral phenyl‐ethyl group replaced the diphenyl substituent of **c106**. Molecular docking simulations, using a simplified model of the experimental cryo‐EM structure of Kv7.2 (Kv7.2_c106/216‐330‐closed_ – described in Figure 3; see chapter 4 of Supporting Information file), revealed two possible binding modes for each of these **c107** enantiomers: one in which the phenyl group (*phenyl‐in* pose) occupies the hydrophobic pocket lined by residues F304 and L307 in S6_I_, and L312 in S6_II_ (Figure 8a,b), and a second one in which the same pocket is occupied by the ethyl group (*ethyl‐in* pose, Figure 8a,c). Docking scores of *ethyl‐in* and *phenyl‐in* poses for **c107R** and **c107S** suggested the *phenyl‐in* pose as the most favorable for both enantiomers, with **c107R** predicted as the tightest binder (Table S3). We challenged these molecular docking predictions by performing 1200 ns‐long MD simulations of the **c107R**/Kv7.2_c106/216‐330‐closed_ and **c107S**/Kv7.2_c106/216‐330‐closed_ complexes, each simulated in both the *phenyl‐in* and *ethyl‐in* bound state. For **c107R**, the *phenyl‐in* configuration remained stable throughout the simulation; conversely, when the *ethyl‐in* configuration was simulated, the compound rearranged to the *phenyl‐in* state (Figure S86). Conversely, the most stable bound conformation for **c107S** corresponds to the *ethyl‐in* state, while its *phenyl‐in* configuration rearranged into *ethyl‐in* (Figure S86). Of note, when in *ethyl‐in* pose, the ethyl group binds into the pocket deeper than the amide substituents of the agonist **c60**, in a manner comparable to the phenyl substituent of **c106** (Figure 8c; Table S4). This is due to the external phenyl ring stabilizing the ethyl group deeper within the pocket by its multiple hydrophobic interactions with residues I300 and F304 in S6_I_, L221 and V225 in S4–S5 linker_II_, and F232 and W236 in S5_II_. This arrangement appears to compensate for the smaller size of the ethyl group, which interacts less efficiently with F304 and L307 (S6_I_), and L312 (S6_II_, Table S2). Consistently, when the ethyl group is located outside the pocket, the interaction scores for residues L221, V225, and L232 decrease (Table S2).

**FIGURE 8:**
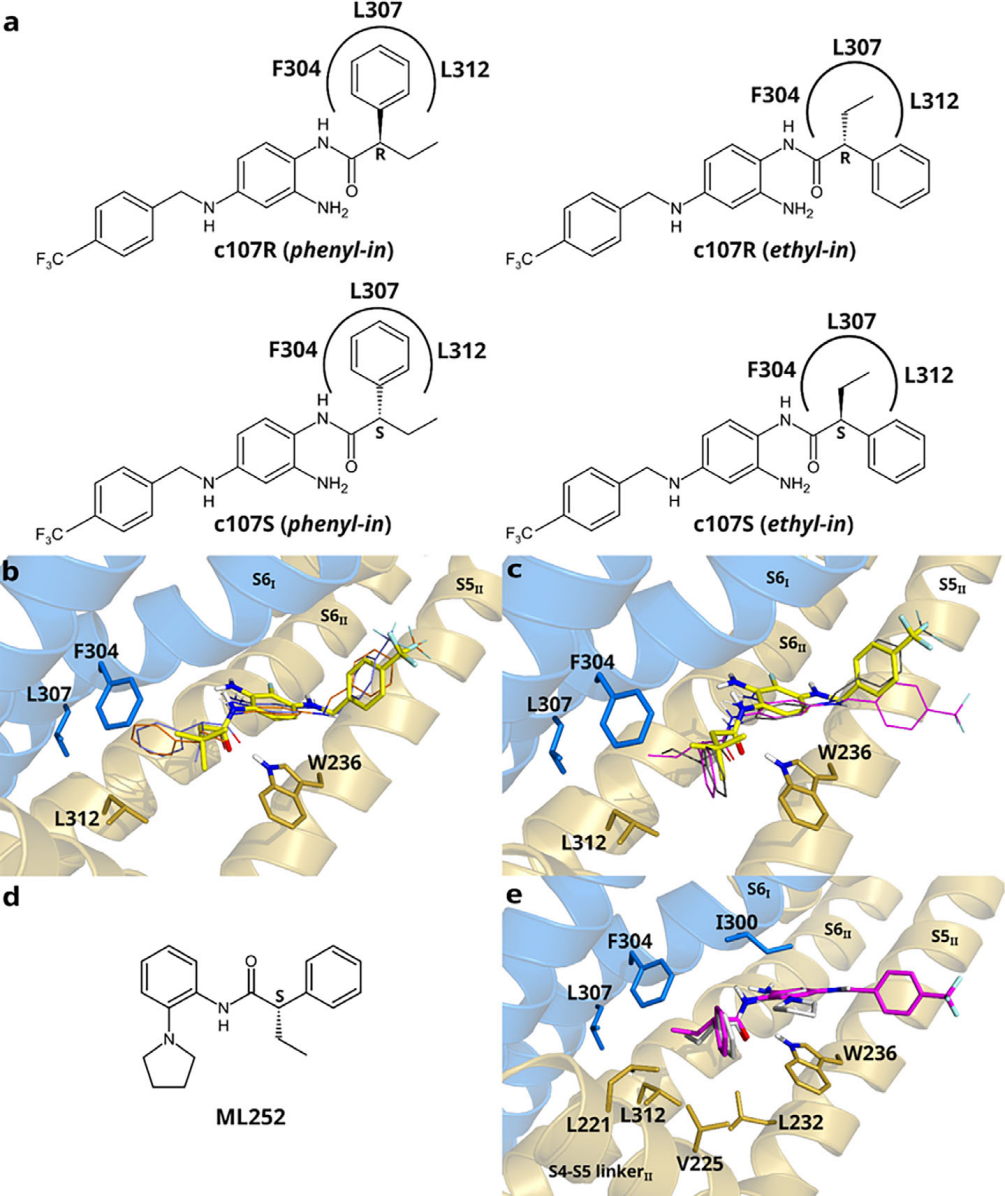
Investigating **c106** binding pose by a chiral analogue (**c107**). (a) Chemical structures of **c107R** and **c107S**, in *phenyl‐in* and *ethyl‐in* configuration. (b) Docking‐predicted bound conformations of **c107R** and **c107S** (*phenyl‐in* pose) compared to experimental bound conformation of **c60**. Docking poses of **c107R** (purple) and **c107S** (orange) are depicted in thin solid sticks. Experimental bound conformation of **c60** is shown in yellow thick solid sticks. (b) Docking‐predicted bound conformations of **c107R** and **c107S** (*ethyl‐in* pose) compared to experimental bound conformation of **c60**. Docking poses of **c107R** (dark gray) and **c107S** (magenta) are depicted in thin solid sticks. Experimental bound conformation of **c60** is shown in yellow thick solid sticks. In both panels b and c the two different Kv7.2 subunits are depicted in thick sticks and transparent cartoons and colored in gold and marine blue. (d) Chemical structure of ML252. (e) Docking‐predicted bound conformations of **c107S** (*ethyl‐in* pose – magenta sticks) and ML252 (light gray sticks). The two different Kv7.2 subunits are depicted in thick sticks and transparent cartoons and colored in gold and marine blue.

To functionally investigate whether the *phenyl‐in* configuration is associated with a more stable Kv7.2 blocking action, the two **c107** enantiomers were synthesized and their effect evaluated on Kv7.2 currents by patch‐clamp electrophysiology. The results obtained revealed that **c107S** displayed a lower inhibitory potency when compared to **c107R** (Figure S85); the **c107S** and **c107R** IC_50s_ were in fact 0.51 ± 0.19 µM and 0.08 ± 0.05 µM, respectively (*p* < 0.05). Noteworthy, the slightly lower potency of **c107R** when compared to **c106** (see Figure 2d) further suggests that the phenyl‐in configuration, the only one possible for **c106** is associated with more potent Kv7.2 blockade. This is also likely due to the fact that the external ethyl group of **c107R** cannot stabilize the phenyl ring within the pocket as effectively as the phenyl substituent of **c106**, which firmly anchors the other phenyl ring inside the binding cavity.

Intriguingly, compound **ML252**, a previously reported Kv7 inhibitor [39, 40] shares the same asymmetric phenyl‐ethyl tail as **c107S** (Figure 8d). In our hands, docking simulations and following MD results predicted that **ML252** bound in *ethyl‐in* orientation, with its phenyl ring establishing hydrophobic interactions with residues I300 and F304 in S6_I_, L221 and V225 in S4–S5 linker_II_, and F232 and W236 in S5_II_; this configuration closely resembles the bound conformation previously described for **c107S** (Figure 8e, Tables S2 and S4).

As previously highlighted, when compared to **c60**, **c106** lacks a fluorine atom in position 3 of the central triamino phenyl scaffold; this substitution was originally introduced in **c60** to improve chemical stability [26, 27, 28, 29, 30]. Thus, a fluorinated analogue of **c106** (namely **c106F**) was synthesized (Scheme S2). **c106F** was three times more potent than **c106** in inhibiting Kv7.2/Kv7.3 currents (Figure 9a,b). This increased inhibitory potency of **c106F** could be ascribed to the ability of fluorine to act as an electron‐withdrawing atom, thus decreasing the electron density of the triamino phenyl ring and strengthening the π–π stacking interactions with the indole ring of W236 (S5_II_) and, to a lesser extent (approximately 3%), with the phenyl ring of F305 (S6_II_), as also suggested for other Kv7 activators [33, 41, 42, 43]. These interactions are completely absent in the **c106**‐bound complex, according to MD simulations of the Kv7.2‐**c106** (Figure 9c) and Kv7.2‐**c106F** (Figure 9d) complexes (Kv7.2_c106/216‐330‐closed_ and **c106F**/Kv7.2_c106/216‐330‐closed_, respectively – see chapter 4 of Supporting Information file) and may contribute to the enhanced stability of ligand binding. Although these findings are preliminary and require further validation, they are consistent with structural observations reported for XEN1101 (Azetukalner), a recently described Kv7 activator, in which the π–π stacking with W236 has also been proposed to contribute to ligand stabilization [43]. Noteworthy, fluorination of the triamino phenyl scaffold also improved the chemical stability of retigabine and retigabine analogues [26].

**FIGURE 9:**
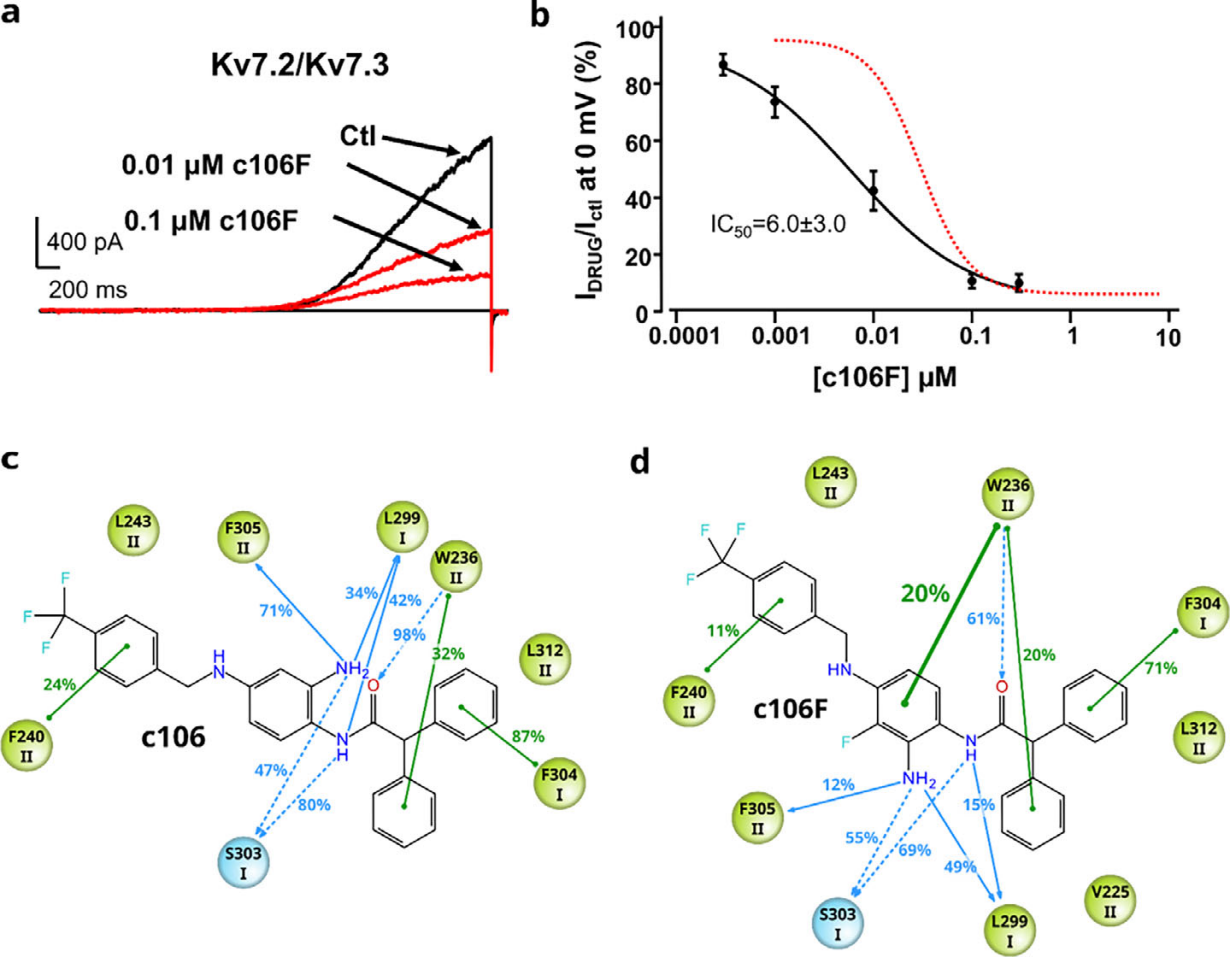
Effect of **c106F** on Kv7.2/7.3 currents. (a) Representative whole‐cell current traces from Kv7.2/Kv7.3 channels activated by the indicated ramp protocol recorded in control conditions and upon exposure to 0.01 and 0.1 µM c106F. (b) Concentration‐response curves reporting the effects of **c106F** on maximal current density (*I*_DRUG_/*I*_control_) calculated at +20 mV for Kv7.2/Kv7.3. Each data point is the mean ± SEM of 32 recorded cells (n); *N* = 4. The red dotted line represents the concentration‐response curve of **c106**. (c, d) Ligand interaction diagrams of 1200 ns‐long MD simulations of Kv7.2 in complex with **c106** (c) and **c106F** (d). Only residues interacting with the ligand for at least 120 ns out of 1200 ns of MD simulation time are shown. Hydrophobic residues are depicted in light green, polar ones in cyan. H‐bonds are represented by light blue arrows (dashed when side chain atoms are involved); green solid lines represent π–π interactions. The π–π stacking interaction with W236 is highlighted by a thicker green line.

An alternative hypothesis to explain the higher potency associated to the introduction of the F atom at position 3 of the central triamino phenyl scaffold is that fluorine might establish a halogen bond with the backbone carbonyl oxygens of nearby residues F304 and F305 (S6_II_). However, this possibility was considered much less likely for the following reasons: in the case of F304, the distance between fluorine and the oxygen largely exceeded the sum of their van der Waals radii, rendering this interaction impossible according to the IUPAC criteria for halogen bond formation (Figure S87) [44]. Regarding F305, although in a fraction of the MD simulation the fluorine–oxygen distance fell within the parameters indicated by IUPAC (Figure S88) [44], the C─F and C═O bonds are neither collinear nor coplanar, suggesting an unfavorable geometric arrangement (Figure S89); in addition, the high electronegativity and low polarizability of fluorine would prevent the development of a significant halogen bond.

### 2.5 **c106** Blocks Currents From Homomeric or Heteromeric Channels Incorporating the Kv7.2 R201C GoF Variant

Although most pathogenic DEE‐causing variants in Kv7.2 are LoF [5, 6], and uncontrolled studies have revealed that children with KCNQ2‐DEE exhibit clinically significant improvement in seizures as well as developmental outcomes when treated with retigabine [45, 46], a significant fraction prompts GoF effects when expressed in vitro, and it has been suggested that these latter might require a therapeutic approach with a Kv7 blocker [47]. A paradigmatic example of a Kv7.2 GoF variant is the R201C, which is found recurrently in patients with neonatal‐onset encephalopathy, prominent startle‐like myoclonus triggered by sound or touch, and burst‐suppression EEG pattern [7]. In vitro, homomeric Kv7.2 R201C channels showed an almost complete loss of time dependence in current activation kinetics, and a mostly linear G/V, indicative of a loss of voltage‐dependent gating [9]. To determine whether **c106** could counteract the GoF effects caused by the R201C variant, we compared its ability to inhibit currents carried by homomeric Kv7.2 R201C channels with those of wild‐type Kv7.2. Following application of a voltage‐ramp protocol from −80 to +20 mV, **c106** produced a concentration‐dependent inhibition of both Kv7.2 and Kv7.2 R201C currents (Figure 10a,b). Specifically, 0.01 and 0.1 µM c106 inhibited approximately 10% and 70% of the currents measured at 0 mV (Figure 10b).

**FIGURE 10:**
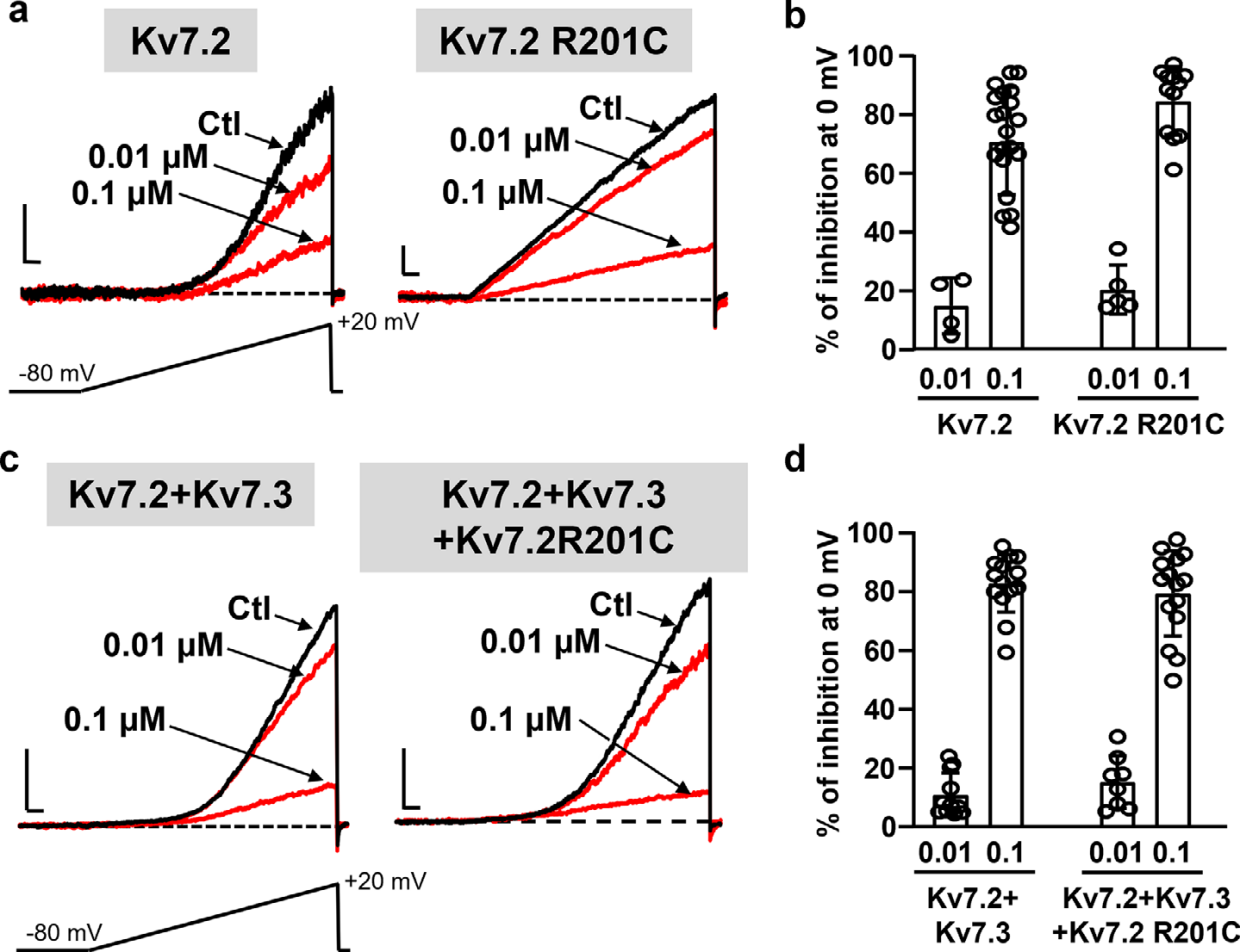
Effect of **c106** on homomeric and heteromeric Kv7.2/Kv7.3 channels carrying the DEE‐causing Kv7.2 GoF variant R201C. (a) Representative whole‐cell current traces from Kv7.2 and Kv7.2 R201C channels heterologously‐expressed in CHO cells activated by the indicated ramp protocol recorded in control conditions and upon exposure to 0.01 and 0.1 µM **c106**. Current scale, 200 pA; time scale, 200 ms. (b) Percent (%) of current inhibition by **c106** calculated at 0 mV. Each bar is mean ± SEM of 4–22 recorded cells (n), as indicated by the individual dots (*N* = 3). (c) Representative whole‐cell current traces from Kv7.2 + Kv7.3 and Kv7.2 + Kv7.2R201C + Kv7.3 channels heterologously‐expressed in CHO cells activated by the indicated ramp protocol recorded in control conditions and upon exposure to 0.01 and 0.1 µM **c106**. Current scale, 500 pA; time scale, 200 ms. (d) Percent (%) of current inhibition by **c106** calculated at 0 mV. Each bar is mean ± SEM of 12–16 recorded cells (n), as indicated by the individual dots (*N* = 3).

Comparable results were obtained upon expression of heteromeric Kv7.2/Kv7.3 channels and Kv7.2/Kv7.2 R201C/Kv7.3 channels (cDNA transfection ratio 0.5:0.5:1), an experimental condition designed to mimic the heterozygous genetic status of affected patients (Figure 10c,d). Although the hypothesis that Kv7 blockers may serve as therapeutic candidates for GoF KCNQ disorders remains currently speculative and requires in vivo validation in both preclinical and clinical settings, these findings highlight **c106** as a possible investigational and, possibly, therapeutic tool in these conditions [7, 23].

## 3 Conclusion

In the present study we describe the results from modelling, functional and structural (cryo‐EM) experiments performed to investigate the molecular basis for the opposite pharmacological profile shown by two analogues of the neuronal Kv7 channels activator retigabine, a clinically approved anticonvulsant drug. In particular, we herein demonstrate that, in Kv7.2 channels, the activator **c60,** bearing a smaller (*t*‐butyl), and the inhibitor **c106** which carries a larger (diphenyl) substituent at the amide nitrogen of retigabine, occupy the same binding pocket located at the S5–S6 interface in the PD between two adjacent subunits. However, within this pocket, the replacement of a bulky leucine at position 307 with a smaller valine (L307V) fully prevented the inhibitory action of **c106**, whereas it failed to interfere with the stimulatory action of **c60**. Our results, achieved by a multidisciplinary pipeline of complementary technical approaches, reveal the detailed molecular mechanisms by which small structural changes in a common pharmacophoric scaffold leads to opposite functional effects. The mechanisms described may be targeted for the optimized design of more potent and selective Kv7 channel modulators, a critical medical need for the treatment of a wide spectrum of neuropsychiatric disorders.

## Author Contributions

**Tania Ciaglia**: Investigation, formal analysis. **Giusy Carleo**: Investigation, formal analysis. **Zhenni Yang**: Investigation, methodology. **Francesca Di Matteo**: Investigation. **Federica De Rosa**: Investigation. **Gerardina Smaldone**: Investigation. **Zhao‐Bing Gao**: Conceptualization. **Marco D'alì**: Investigation. **Rita Turcio**: Investigation. **Demin Ma**: Investigation. **Veronica Di Sarno**: Investigation. **Nannan Su**: Validation. **Salvatore Vincenzo Giofrè**: Formal analysis. **Giacomo Pepe**: Data curation. **Pietro Campiglia**: Conceptualization. **Alessia Bertamino**: Formal analysis. **Francesco Miceli**: Writing – original draft, supervision. **Jiangtao Guo**: Conceptualization, funding acquisition, supervision, writing – review and editing. **Nunzio Iraci**: Conceptualization, supervision, writing – original draft, writing – review and editing, software, visualization. **Carmine Ostacolo**: Conceptualization, data curation, writing – original draft, writing – review and editing, project administration, funding acquisition, methodology. **Maurizio Taglialatela**: Methodology, conceptualization, writing – original draft, writing – review and editing, project administration, funding acquisition, resources.

## Conflicts of Interest

The University of Salerno has filed IP (WO2020157126A1A1) on potassium channel modulators on which CO, MT, FM, PC, AB and NI are inventors. Some of the molecules disclosed in the present paper are object of the Italian patent application n.102026000021997 filed on 07/28/2026 by CO, MT, FM, AB and NI. The other authors declare no conflict of interest.

## Supporting information




**Supporting File**: anie74291‐sup‐0001‐SuppMat.pdf.

## Data Availability

The data that support the findings of this study are also available in the supplementary material of this article. The authors have cited additional references within the Supporting Information [48, 49, 50, 51, 52, 53, 54, 55, 56, 57, 58, 59, 60, 61, 62, 63]. The 3D cryo‐EM density maps and structure coordinates have been deposited in the Electron Microscopy Data Bank (EMDB) and the Protein Data Bank (PDB) with accession codes EMD‐68639 and PDB 22SA for Kv7.2‐**c60** and EMD‐68640 and PDB 22SB for Kv7.2‐**c106**, respectively.

## References

[1] D. Lopez‐Mateos , B. J. Harris , A. Hernández‐González , V. Yarov‐Yarovoy , and H. Wulff , “Recent Advances in the Pharmacology of Voltage‐Gated Ion Channels,” Pharmacological Reviews 77 (2025): 100090, 10.1016/j.pharmr.2025.100090. PMC13095425 41092810

[2] D. A. Brown and P. R. Adams , “Muscarinic Suppression of a Novel Voltage‐Sensitive K^+^ Current in a Vertebrate Neuron,” Nature 283 (1980): 673–676, 10.1038/283673a0. 6965523

[3] H. Wang , “KCNQ2 and KCNQ3 Potassium Channel Subunits: Molecular Correlates of the M‐Channel,” Science 282 (1998): 1890–1893, 10.1126/science.282.5395.1890. 9836639

[4] P. Nappi , F. Miceli , M. V. Soldovieri , P. Ambrosino , V. Barrese , and M. Taglialatela , “Epileptic Channelopathies Caused by Neuronal Kv7 (KCNQ) Channel Dysfunction,” Pflügers Archiv – European Journal of Physiology 472 (2020): 881–898. 10.1007/s00424-020-02404-2 32506321

[5] F. Miceli , M. V. Soldovieri , P. Ambrosino , et al., “Genotype–Phenotype Correlations in Neonatal Epilepsies Caused by Mutations in the Voltage Sensor of Kv7.2 Potassium Channel Subunits,” Proceedings of the National Academy of Sciences USA 110 (2013): 4386–4391, 10.1073/pnas.1216867110. PMC3600471 23440208

[6] G. Orhan , M. Bock , D. Schepers , et al., “Dominant‐Negative Effects of KCNQ2 Mutations are Associated With Epileptic Encephalopathy,” Annals of Neurology 75 (2014): 382–394, 10.1002/ana.24080. 24318194

[7] S. B. Mulkey , B. Ben‐Zeev , J. Nicolai , et al., “Neonatal Nonepileptic Myoclonus is a Prominent Clinical Feature of KCNQ2 Gain‐of‐Function Variants R201C and R201H,” Epilepsia 58 (2017): 436–445, 10.1111/epi.13676. PMC5339037 28139826

[8] M. Nappi , G. Alberini , A. Berselli , et al., “Constitutive Opening of the Kv7.2 Pore Activation Gate Causes KCNQ2‐Developmental Encephalopathy,” Proceedings of the National Academy of Sciences 121 (2024): e2412388121, 10.1073/pnas.2412388121. PMC11626135 39602259

[9] F. Miceli , M. V. Soldovieri , P. Ambrosino , et al., “Early‐Onset Epileptic Encephalopathy Caused by Gain‐of‐Function Mutations in the Voltage Sensor of Kv7.2 and Kv7.3 Potassium Channel Subunits,” Journal of Neuroscience 35 (2015): 3782–3793, 10.1523/JNEUROSCI.4423-14.2015. PMC6605567 25740509

[10] T. T. Sands , F. Miceli , G. Lesca , et al., “Autism and Developmental Disability Caused by KCNQ3 Gain‐Of‐Function Variants,” Annals of Neurology 86 (2019): 181–192, 10.1002/ana.25522. 31177578

[11] A. D. Wei , P. Wakenight , T. A. Zwingman , et al., “Human KCNQ5 De Novo Mutations Underlie Epilepsy and Intellectual Disability,” Journal of Neurophysiology 128 (2022): 40–61, 10.1152/jn.00509.2021. PMC9236882 35583973

[12] M. Nappi , V. Barrese , L. Carotenuto , et al., “Gain of Function Due to Increased Opening Probability by Two KCNQ5 Pore Variants Causing Developmental and Epileptic Encephalopathy,” Proceedings of the National Academy of Sciences USA 119 (2022): e2116887119, 10.1073/pnas.2116887119. PMC9169635 35377796

[13] S. Jiang , B. Liu , K. Lin , et al., “Impacted Spike Frequency Adaptation Associated With Reduction of KCNQ2/3 Exacerbates Seizure Activity in Temporal Lobe Epilepsy,” Hippocampus 34 (2024): 58–72, 10.1002/hipo.23587. 38049972

[14] A. C. Coan and F. Cendes , “Understanding the Spectrum of Temporal Lobe Epilepsy: Contributions for the Development of Individualized Therapies,” Expert Review of Neurotherapeutics 13 (2013): 1383–1394, 10.1586/14737175.2013.857604. 24236904

[15] E. Perucca and M. Taglialatela , “Targeting Kv7 Potassium Channels for Epilepsy,” CNS Drugs 39 (2025): 263–288, 10.1007/s40263-024-01155-3. PMC11850491 39853501

[16] B. C. Baculis , J. Zhang , and H. J. Chung , “The Role of Kv7 Channels in Neural Plasticity and Behavior,” Frontiers in Physiology 11 (2020): 568667, 10.3389/fphys.2020.568667. PMC7530275 33071824

[17] M. Wang , N. J. Gamo , Y. Yang , et al., “Neuronal Basis of Age‐Related Working Memory Decline,” Nature 476 (2011): 210–213, 10.1038/nature10243. PMC3193794 21796118

[18] H. Liu , L. Jia , X. Chen , L. Shi , and J. Xie , “The Kv7/KCNQ Channel Blocker XE991 Protects Nigral Dopaminergic Neurons in the 6‐Hydroxydopamine Rat Model of Parkinson's Disease,” Brain Research Bullettin 137 (2018): 132–139, 10.1016/j.brainresbull.2017.11.011. 29174294

[19] B. Yang , H. Liu , S. Zhang , et al., “Pharmacological and Electrophysiological Characterization of the Novel Kv7 Channel Inhibitor Racecadotril in Parkinson's Disease,” Neuropharmacology 278 (2025): 110565, 10.1016/j.neuropharm.2025.110565. 40532787

[20] K. Rockwood , B. L. Beattie , M. R. Eastwood , et al., “A Randomized, Controlled Trial of Linopirdine in the Treatment of Alzheimer's Disease,” Canadian Journal of Neurological Sciences 24 (2015): 140–145, 10.1017/S031716710002148X. 9164692

[21] R. Zaczek , R. J. Chorvat , J. A. Saye , et al., “Two New Potent Neurotransmitter Release Enhancers, 10,10‐bis(4‐Pyridinylmethyl)‐9(10H)‐Anthracenone and 10,10‐bis(2‐Fluoro‐4‐Pyridinylmethyl)‐9(10H)‐Anthracenone: Comparison to Linopirdine,” The Journal of Pharmacology and Experimental Therapeutics 285 (1998): 724–730. 9580619

[22] A. Bayat , S. Iavarone , F. Miceli , et al., “Phenotypic and Functional Assessment of Two Novel KCNQ2 Gain‐of‐Function Variants Y141N and G239S and Effects of Amitriptyline Treatment,” Neurotherapeutics 21 (2024): e00296, 10.1016/j.neurot.2023.10.006. PMC10903081 38241158

[23] A. Nissenkorn , L. Bar , A. Ben‐Bassat , et al., “Donepezil as a New Therapeutic Potential in KCNQ2‐ and KCNQ3‐Related Autism,” Frontiers in Cellular Neuroscience 18 (2024): 1380442, 10.3389/fncel.2024.1380442. PMC11338814 39175503

[24] F. A. Vigil , C. M. Carver , and M. S. Shapiro , “Pharmacological Manipulation of Kv7 Channels as a New Therapeutic Tool for Multiple Brain Disorders,” Frontiers in Physiology 11 (2020): 688, 10.3389/fphys.2020.00688. PMC7317068 32636759

[25] C. Ostacolo , F. Miceli , V. Di Sarno , et al., “Synthesis and Pharmacological Characterization of Conformationally Restricted Retigabine Analogues as Novel Neuronal Kv7 Channel Activators,” Journal of Medicinal Chemistry 63 (2019): 163–185, 10.1021/acs.jmedchem.9b00796. 31815462

[26] S. Musella , L. Carotenuto , N. Iraci , et al., “Beyond Retigabine: Design, Synthesis, and Pharmacological Characterization of a Potent and Chemically Stable Neuronal Kv7 Channel Activator With Anticonvulsant Activity,” Journal of Medicinal Chemistry 65 (2022): 11340–11364, 10.1021/acs.jmedchem.2c00911. PMC9421656 35972998

[27] X. Li , Q. Zhang , P. Guo , et al., “Molecular Basis for Ligand Activation of the Human KCNQ2 Channel,” Cell Research 31 (2020): 52–61, 10.1038/s41422-020-00410-8. PMC7852908 32884139

[28] Y. Huang , D. Ma , Z. Yang , Y. Zhao , and J. Guo , “Voltage‐Gated Potassium Channels KCNQs: Structures, Mechanisms, and Modulations,” Biochemical and Biophysical Research Communications 689 (2023): 149218, 10.1016/j.bbrc.2023.149218. 37976835

[29] D. Ma , Y. Zheng , X. Li , et al., “Ligand Activation Mechanisms of Human KCNQ2 Channel,” Nature Communication 14 (2023): 6632, 10.1038/s41467-023-42416-x. PMC10587151 37857637

[30] M. Kumar , N. Reed , R. Liu , E. Aizenman , P. Wipf , and T. Tzounopoulos , “Synthesis and Evaluation of Potent KCNQ2/3‐Specific Channel Activators,” Molecular Pharmacology 89 (2016): 667–677, 10.1124/mol.115.103200. 27005699

[31] A. Etxeberria , I. Santana‐Castro , M. P. Regalado , P. Aivar , and A. Villarroel , “Three Mechanisms Underlie KCNQ2/3 Heteromeric Potassium M‐Channel Potentiation,” Journal of Neuroscience 24 (2004): 9146–9152, 10.1523/JNEUROSCI.3194-04.2004. PMC6730048 15483133

[32] O. Zaika , C. C. Hernandez , M. Bal , G. P. Tolstykh , and M. S. Shapiro , “Determinants Within the Turret and Pore‐Loop Domains of KCNQ3 K^+^ Channels Governing Functional Activity,” Biophysical Journal 95 (2008): 5121–5137, 10.1529/biophysj.108.137604. PMC2586577 18790849

[33] J. Li , Z. Yang , S. Zhang , et al., “Small Molecule Inhibits KCNQ Channels With a Non‐Blocking Mechanism,” Nature Chemical Biology 21 (2025): 1100–1109, 10.1038/s41589-024-01834-8. 39814994

[34] S. Zhang , D. Ma , K. Wang , et al., “A Small‐Molecule Activation Mechanism That Directly Opens the KCNQ2 Channel,” Nature Chemical Biology 20 (2024): 847–856, 10.1038/s41589-023-01515-y. 38167918

[35] Y. Zhao , Z. Yang , S. Shi , et al., “Structure Basis for the Activation of KCNQ2 by Endogenous and Exogenous Ligands,” Cell Report 45 (2026): 116771, 10.1016/j.celrep.2025.116771. 41442279

[36] Z. Yang , Y. Zheng , D. Ma , et al., “Phosphatidylinositol 4,5‐Bisphosphate Activation Mechanism of Human KCNQ5,” Proceedings of the National Academy of Sciences USA 122 (2025): e2416738122, 10.1073/pnas.2416738122. PMC12002238 40172963

[37] Y. Zheng , H. Liu , Y. Chen , et al., “Structural Insights Into the Lipid and Ligand Regulation of a human Neuronal KCNQ Channel,” Neuron 110 (2022): 237–247.e4, 10.1016/j.neuron.2021.10.029. 34767770

[38] T. Li , K. Wu , Z. Yue , Y. Wang , F. Zhang , and H. Shen , “Structural Basis for the Modulation of Human KCNQ4 by Small‐Molecule Drugs,” Molecular Cell 81 (2021): 25–37.e4, 10.1016/j.molcel.2020.10.037. 33238160

[39] R. Kanyo , S. M. Lamothe , A. Urrutia , et al., “Site and Mechanism of ML252 Inhibition of Kv7 Voltage‐Gated Potassium Channels,” Function 4 (2023): zqad021, 10.1093/function/zqad021. PMC10278987 37342413

[40] M. Borgini , P. Mondal , R. Liu , and P. Wipf , “Chemical Modulation of Kv7 Potassium Channels,” RSC Medicinal Chemistry 12 (2021): 483–537, 10.1039/D0MD00328J. PMC8128042 34046626

[41] M. Pökl , A. Sridhar , D. J. A. Frampton , V. A. Linhart , L. Delemotte , and S. I. Liin , “Subtype‐Specific Modulation of Human KV7 Channels by the Anticonvulsant Cannabidiol Through a Lipid‐Exposed Pore‐Domain Site,” British Journal of Pharmacology 180 (2023): 2956–2972. 10.1111/bph.16183 37377025

[42] D. Zhang , W. Xiang , J. Liu , et al., “Design, synthesis, and Structure‐Activity Relationship of 5,7‐dimethylbenzo[d]thiazoles as Novel Kv7.2/7.3 Activators With Antiepileptic Effects,” European Journal of Medicinal Chemistry 292 (2025): 117660, 10.1016/j.ejmech.2025.117660. 40315730

[43] F. Lu , X. Huang , G. Cai , et al., “Structural Basis for the Assembly and Modulation of Human M‐Channels,” Vita (2026), E‐pub ahead of print, 10.15302/vita.2026.05.0035.

[44] G. R. Desiraju , P. S. Ho , L. Kloo , et al., “Definition of the Halogen Bond (IUPAC Recommendations 2013),” Pure and Applied Chemistry 85 (2013): 1711–1713, 10.1351/PAC-REC-12-05-10.

[45] J. J. Millichap , F. Miceli , M. De Maria , et al., “Infantile Spasms and Encephalopathy Without Preceding Neonatal Seizures Caused by KCNQ2 R198Q, a Gain‐of‐function Variant,” Epilepsia 58 (2016): e10–e15. 10.1111/epi.13601 PMC5219941 27861786

[46] D. Knight , S. Mahida , M. Kelly , A. Poduri , and H. E. Olson , “Ezogabine Impacts Seizures and Development in Patients With KCNQ2 Developmental and Epileptic Encephalopathy,” Epilepsia 64 (2023): e143–e147, 10.1111/epi.17627. 37096745

[47] eds. S. Weckhuysen and A. L. George , KCNQ2‐ and KCNQ3‐Associated Epilepsy (Cambridge University Press, 2022), 57.

[48] G. Madhavi Sastry , M. Adzhigirey , T. Day , R. Annabhimoju , and W. Sherman , “Protein and Ligand Preparation: Parameters, Protocols, and Influence on Virtual Screening Enrichments,” Journal of Computer‐Aided Molecular Design 27 (2013): 221–234. 10.1007/s10822-013-9644-8 23579614

[49] W. L. Jorgensen , D. S. Maxwell , and J. Tirado‐Rives , “Development and Testing of the OPLS All‐Atom Force Field on Conformational Energetics and Properties of Organic Liquids,” Journal of Americal Chemical Society 118 (1996): 11225–11236, 10.1021/ja9621760.

[50] M. A. Lomize , I. D. Pogozheva , H. Joo , H. I. Mosberg , and A. L. Lomize , “OPM Database and PPM Web Server: Resources for Positioning of Proteins in Membranes,” Nucleic Acids Research 40 (2012): D370–D376, 10.1093/nar/gkr703. PMC3245162 21890895

[51] R. A. Friesner , J. L. Banks , R. B. Murphy , et al., “Glide: A New Approach for Rapid, Accurate Docking and Scoring. 1. Method and Assessment of Docking Accuracy,” Journal of Medicinal Chemistry 47 (2004): 1739–1749, 10.1021/jm0306430. 15027865

[52] T. A. Halgren , R. B. Murphy , R. A. Friesner , et al., “Glide: A New Approach for Rapid, Accurate Docking and Scoring. 2. Enrichment Factors in Database Screenin,” Journal of Medicinal Chemistry 47 (2004): 1750–1759, 10.1021/jm030644s. 15027866

[53] R. A. Friesner , R. B. Murphy , M. P. Repasky , et al., “Extra Precision Glide: Docking and Scoring Incorporating a Model of Hydrophobic Enclosure for Protein−Ligand Complexes,” Journal of Medicinal Chemistry 49 (2006): 6177–6196, 10.1021/jm051256o. 17034125

[54] W. L. Jorgensen , J. Chandrasekhar , J. D. Madura , R. W. Impey , and M. L. Klein , “Comparison of Simple Potential Functions for Simulating Liquid Water,” The Journal of Chemical Physics 79 (1983): 926–935, 10.1063/1.445869.

[55] W. Humphrey and A. Dalke , “VMD: Visual Molecular Dynamics,” Journal of Molecular Graphic 14 (1996): 33–38, 10.1016/0263-7855(96)00018-5. 8744570

[56] C. L. Morales‐Perez , C. M. Noviello , and R. E. Hibbs , “Manipulation of Subunit Stoichiometry in Heteromeric Membrane Proteins,” Structure 24 (2016): 797–805, 10.1016/j.str.2016.03.004. PMC4856541 27041595

[57] S. Q. Zheng , E. Palovcak , J.‐P. Armache , K. A. Verba , Y. Cheng , and D. A. Agard , “MotionCor2: Anisotropic Correction of Beam‐induced Motion for Improved Cryo‐electron Microscopy,” Nature Methods 14 (2017): 331–332, 10.1038/nmeth.4193. PMC5494038 28250466

[58] K. Zhang , “Gctf: Real‐Time CTF Determination and Correction,” Journal of Structural Biology 193 (2016): 1–12, 10.1016/j.jsb.2015.11.003. PMC4711343 26592709

[59] S. H. Scheres , “RELION: Implementation of a Bayesian Approach to Cryo‐EM Structure Determination,” Journal of Structural Biology 180 (2012): 519–530, 10.1016/j.jsb.2012.09.006. PMC3690530 23000701

[60] P. Emsley , B. Lohkamp , W. G. Scott , and K. Cowtan , “Features and Development of Coot,” Acta Crystallographica D—Biological crystallography 66 (2010): 486–501. 10.1107/S0907444910007493 PMC2852313 20383002

[61] P. D. Adams , P. V. Afonine , G. Bunkóczi , et al., “PHENIX: A Comprehensive Python‐Based System for Macromolecular Structure Solution,” Acta Crystallographica D—Biological Crystallography 66 (2010): 213–221. 10.1107/S0907444909052925 PMC2815670 20124702

[62] V. B. Chen , et al., “MolProbity: All‐atom Structure Validation for Macromolecular Crystallography,” Acta Crystallographica D—Biological Crystallography 66 (2010): 12–21, 10.1107/S0907444909042073. PMC2803126 20057044

[63] E. F. Pettersen , T. D. Goddard , C. C. Huang , et al., “UCSF ChimeraX: Structure Visualization for Researchers, Educators, and Developers,” Protein Science 30 (2021): 70–82, 10.1002/pro.3943. PMC7737788 32881101

