# Supplementary material for: Molecular Basis for Activation to Inhibition Switching in Kv7.2 Channel Modulators
Source: Angew Chem Int Ed Engl. 2026 Aug 22;65(42):e7948330. doi: 10.1002/anie.7948330 (PMC13653627; doi:10.1002/anie.7948330)
Supplement: Supplementary file 1 [file ANIE-65-e7948330-s001.pdf]

Supplementary Information for

Molecular Basis for Activation to Inhibition Switching in Kv7.2 Channel Modulators

Tania Ciaglia,[a],# Giusy Carleo,[b],# Zhenni Yang, [c,d],# Francesca Di Matteo,[a] Federica De Rosa,[b] Gerardina Smaldone,[a] Zhao-Bing Gao,[e],# Marco D'Alì, [f] Rita Turcio, [a], Demin Ma, [c,d] Veronica Di Sarno,[a] Nannan Su,[g] Salvatore Vincenzo Giofrè,[f] Giacomo Pepe,[a] Pietro Campiglia,[a] Alessia Bertamino,[a] Francesco Miceli,[b] Jiangtao Guo,*[c,d] Nunzio Iraci,*[f] Carmine Ostacolo*[a] and Maurizio Taglialatela*[b]

[a] T Ciaglia, F Di Matteo, G Smaldone, R Turcio, V. Di Sarno, G Pepe, P Campiglia, A Bertamino, C Ostacolo
Department of Pharmacy
University of Salerno
Via G. Paolo II, 84084, Fisciano (SA), Italy.

[b] G Carleo, F De Rosa, F Miceli, M Taglialatela
Department of Neuroscience, Reproductive Sciences and Dentistry, Section of Pharmacology
University of Naples Federico II
Via Pansini 5, 80131, Naples, Italy

[c] Z Yang, D Ma, J Guo
Nanhu Brain-computer Interface Institute
Hangzhou, Zhejiang, China

[d] Z Yang, D Ma, J Guo
Department of Biophysics and Neurology of the Fourth Affiliated Hospital
School of Medicine, Zhejiang University
866 Yuhangtang Rd, Hangzhou 310058, China

[e] Z-B Gao
State Key Laboratory of Drug Research
Shanghai Institute of Materia Medica, Chinese Academy of Sciences
555 Zuchongzhi Rd, Pudong, Shanghai, China

[f] M D'Alì, SV Giofrè, N Iraci
Department of Chemical, Biological, Pharmaceutical and Environmental Sciences (CHIBIOFARAM)
University of Messina
Viale Ferdinando Stagno d'Alcontres 31, 98166, Messina, Italy

[g] N Su
Department of Neurology of the Fourth Affiliated Hospital, Center for Membrane Receptors and Brain Medicine
School of Medicine, Zhejiang University
866 Yuhangtang Rd, Hangzhou 310058, China

#TC, GC, and YZ contributed equally as first Authors

*JG, NI, CO and MT contributed equally as last and corresponding Authors

E-mail: jiangtaoguo@zju.edu.cn; nunzio.iraci@unime.it; costacolo@unisa.it; mtaglial@unina.it;

CONTENTS

**1. CHEMISTRY**	4-99
1.1 GENERAL	4
1.2 SYNTHETIC PROCEDURES AND SCHEMES	4
1.3 SYNTHETIC EXPERIMENTAL PROCEDURES	6
**Figures S1-S82**: NMR, HPLC and CD spectra of the synthesized compounds	18-99
**2. AUTOMATED PATCH-CLAMP EXPERIMENTS**	100-102
2.1 GENERAL	100
**Table S1.** Molecular structures of the synthesized compounds and their effects	100
**3. MANUAL PATCH-CLAMP EXPERIMENTS**	103-106
3.1 CELL CULTURE, MUTAGENESIS AND TRANSIENT TRANSFECTION	103
3.2 MANUAL PATCH-CLAMP	103
3.3 STATISTICAL ANALYSIS	103
**Figure S83.** Effect of c106 on the activation voltage-dependence of Kv7.2/3 currents	104
**Figure S84.** Functional properties of the currents carried by homomeric Kv7.2 (CTL), Kv7.2 L312V, Kv7.2 L307V, and Kv7.2 L307A channels	105
**Figure S85.** Effects of c107R and c107S on Kv7.2/3 currents	106
**4. MOLECULAR MODELING**	107-113
4.1 LIGANDS PREPARATION FOR MOLECULAR MODELLING EXPERIMENTS	107
4.2 hKV7.2216-330 MODELS PREPARATION	107
4.3 INDUCED FIT DOCKING	107
4.4 GRID PREPARATION OF "Kv7.2c106/216-330-CLOSED"	107
4.5 MOLECULAR DOCKING OF c106F	107
4.6 MOLECULAR DOCKING OF c107R, c107S AND ML252	107
4.7 MOLECULAR DYNAMICS OF "c60/Kv7.$2_{RET/216-330-CLOSED}$", "c106/Kv7.$2_{RET/216-330-CLOSED}$" "Kv7.$2_{c60/216-330-OPEN}$", "Kv7.$2_{c106/216-330-CLOSED}$", "c106F/Kv7.$2_{c106/216-330-CLOSED}$", "c107R/Kv7.$2_{c106/216-330-CLOSED}$", "c107S/Kv7.$2_{c106/216-330-CLOSED}$", and "ML252/Kv7.$2_{c106/216-330-CLOSED}$" COMPLEXES	108
**Table S2**: Docking-predicted residue interaction scores of **c107R**, **c107S** and ML252	109
**Table S3**: Docking scores of **c107R**, **c107S** and ML252	110
**Figure S86**: RMSD values of **c107R** and **c107S** in phenyl-in and ethyl-in pose	110

**Table S4**: Average distances between Kv7.2 residues F304, L307, and L312 and the terminal groups of agonists **c60**, retigabine, and Kv7 blockers	111
**Figure S87**: Distance between the fluorine of **c60** and **c106** and the carbonyl oxygen of the F304 backbone	111
**Figure S88**: Distance between the fluorine of **c60** and **c106** and the carbonyl oxygen of the F305 backbone	112
**Figure S89:** Representative bound conformations of compounds **c60** and **c106** from the most populated conformational clusters from the MD simulations	112
**Figure S90:** Superimposed transmembrane Pore Domains of Kv7.2 -CaM-PIP2-HN37 (open state) and Kv7.2 -CaM-HN37 (closed state) cryo-EM structures	113
**5. SINGLE-PARTICLE CRYO-ELECTRON MICROSCOPY**	114-121
5.1 PROTEIN EXPRESSION AND PURIFICATION	114
5.2 CRYO-EM DATA ACQUISITION	114
5.3 IMAGE PROCESSING	115
5.4 MODEL BUILDING, REFINEMENT, AND VALIDATION	115
**Table S5.** Data collection and refinement statistics	116
**Figure S91.** Structure determination of Kv7.2-c60	117
**Figure S92.** Structure determination of Kv7.2-PIP_2(-)-c106 in the presence of PIP_2	118
**Figure S93.** Structure determination of Kv7.2-c106	119
**Figure S94.** Comparisons of the Kv7.2-c60 structure with related Kv7.2 structures.	120
**Figure S95.** Structural comparison of Kv7.2-c106 and Kv7.2 in apo state	121

1. CHEMISTRY

1.1 GENERAL

All the reagents were purchased by Fluorochem Ltd (Hadfield, UK) while solvents were purchased from Merck (Milan, Italy), unless otherwise reported. Reactions were performed under magnetic stirring in round-bottomed flasks unless otherwise noted. Moisture-sensitive reactions were conducted in oven-dried glassware under nitrogen stream, using freshly distilled solvents. TLC analysis of reaction mixtures was performed on precoated glass silica gel plates (F254, 0.25 mm, VWR International), while crude products were purified by the Isolera Spektra One automated flash chromatography system (Biotage, Uppsala, Sweden), using commercial silica gel cartridges (SNAP KP-Sil, Biotage).
NMR spectra were recorded on a Bruker Avance 400 MHz apparatus, at room temperature. Chemical shifts were reported in δ values (ppm) relative to internal Me_4Si for ^1H and ^{13}C NMR. J values were reported in hertz (Hz). ^1H NMR peaks were described using the following abbreviations: s (singlet), d (doublet), t (triplet), and m (multiplet).
HR-MS spectra were recorded by LTQ-Orbitrap-XL-ETD mass spectrometer (Thermo Scientific, Bremen, Germany), equipped with an ESI source.
All the final compounds showed a purity ≥95% as assessed by RP-UHPLC-PDA analysis, performed using a Nexera UHPLC system (Shimadzu, Kyoto, Japan) consisting of a CBM-40 lite controller, two LC-40B X3 pumps, an SPD-M 40 photo diode array detector, a CTO-30A column oven and, a SIL-40C X3 autosampler. The chromatographic analysis was accomplished on a Kinetex® Evo C18 column, 150 × 2.1 mm × 2.6 µm (Phenomenex®, Bologna, Italy) maintained at 40 °C. The optimal mobile phase consisted of 0.1% HCOOH/H2O v/v (A) and 0.1% HCOOH/ACN v/v (B) delivered at constant flow rate of 0.3 mL /min -1. Analysis was performed in gradient elution as follows: 0–20.00 min, 5–95% B; 20.00–25.00 min, isocratic to 95% B; then five minutes for column re-equilibration. Data acquisition was set in the range 190–800 nm and chromatograms were monitored at 254 nm.
UHPLC-PDA analyses of chiral compounds (**c107R** and **c107S**) were performed using the same instrumentation described above. Chromatographic analyses were performed on a Lux Cellulose-1 column (250 × 4.6 mm, 5 µm; Phenomenex, Bologna, Italy) maintained at 20°C. The mobile phase consisted of acetonitrile/isopropanol (95:5, v/v) containing 0.1% (v/v) diethylamine, delivered at a constant flow rate of 0.8 mL min^{-1} under isocratic conditions. The injection volume was 2 µL. PDA detection was performed with a sampling rate of 40 Hz and a time constant of 0.005 s, and chromatograms were extracted at 254 nm. Data acquisition and processing were carried out using LabSolutions® software (version 3.50.346, Shimadzu).

The circular dichroism spectra of compounds **c107R/S** were recorded on a JASCO J810 spectropolarimeter at room temperature, using a 1 mm pathlength quartz cell. The data were collected over a wavelength range 190-260 nm (1 nm bandwidth, 4 accumulation and scanning speed of 10 nm/min). Solvent spectra were subtracted from each measurement, obtaining the final spectra. The compounds were dissolved in methanol at 10 mM concentration and then diluted to reach the operative concentration of 0.1 mM. Curve analysis were performed using GraphPad Prism 8.0.2 (GraphPad Software, LaJolla, CA).
Optical rotations for chiral compounds were measured on an Atago Polax 2-L polarimeter (Atago, Tokio, Japan) at a concentration of 0.1 g/100 mL and 25°C temperature.

1.2 SYNTHETIC PROCEDURES AND SCHEMES

Derivatives **c97-c122** were synthesized as described in **Scheme S1**. 2-Nitro-1,4-phenylenediamine was reacted with 4-(trifluoromethyl) benzyl bromide in basic medium furnishing intermediate **1** in 75% of yield. The following reaction with benzyl chloroformate using diisopropylethylamine (DIPEA) as base led to the protected intermediate **2** (83% of yield). Intermediate **2** was then reacted with different commercially available acyl chlorides under microwaves irradiation employing 1,5-diazabiciclo(5.4.0)undec-7-ene (DBU) as base, yielding the amidic intermediates **3-27** (48-73% yield). The final reduction/deprotection of **3-27** performed in a mixture of MeOH/THF, using ammonium formate and Pd/C as catalyst, gave the final compounds **c97-122** (44-67% yield).

Scheme S1. Synthesis of derivatives **c97-c122**.

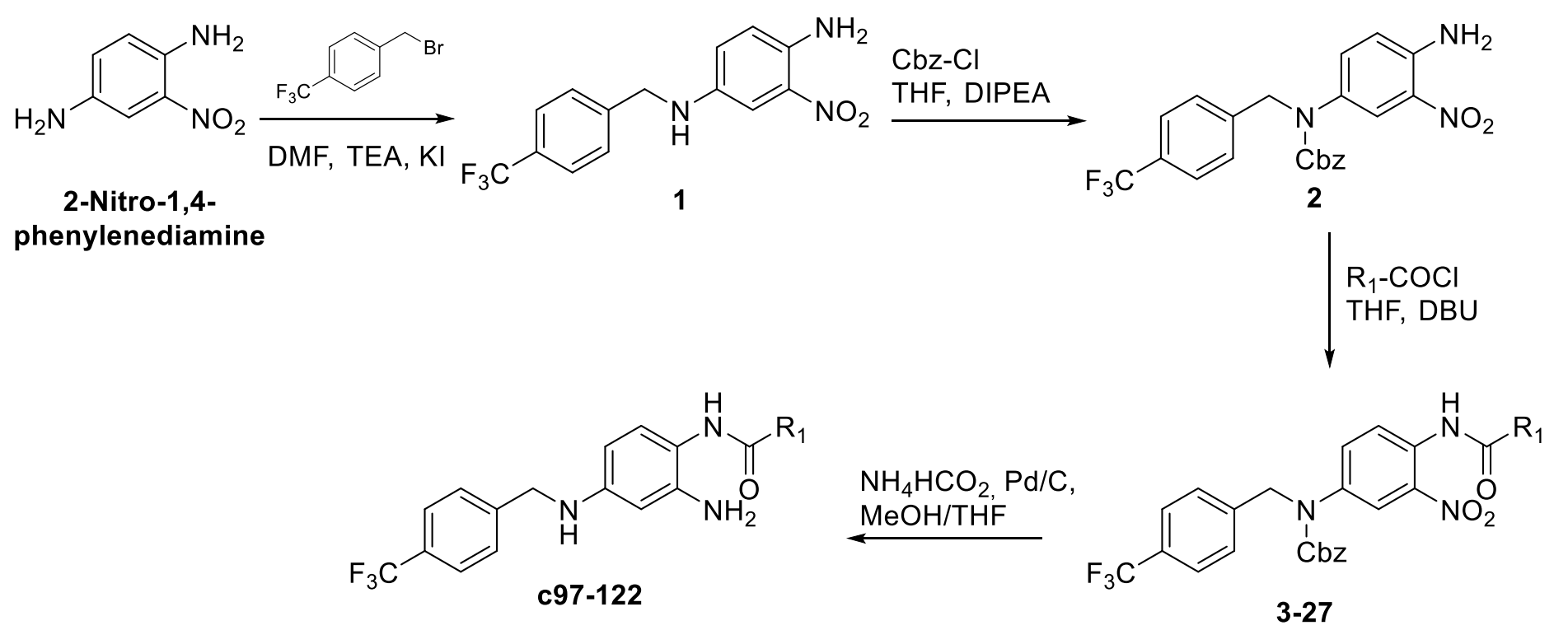

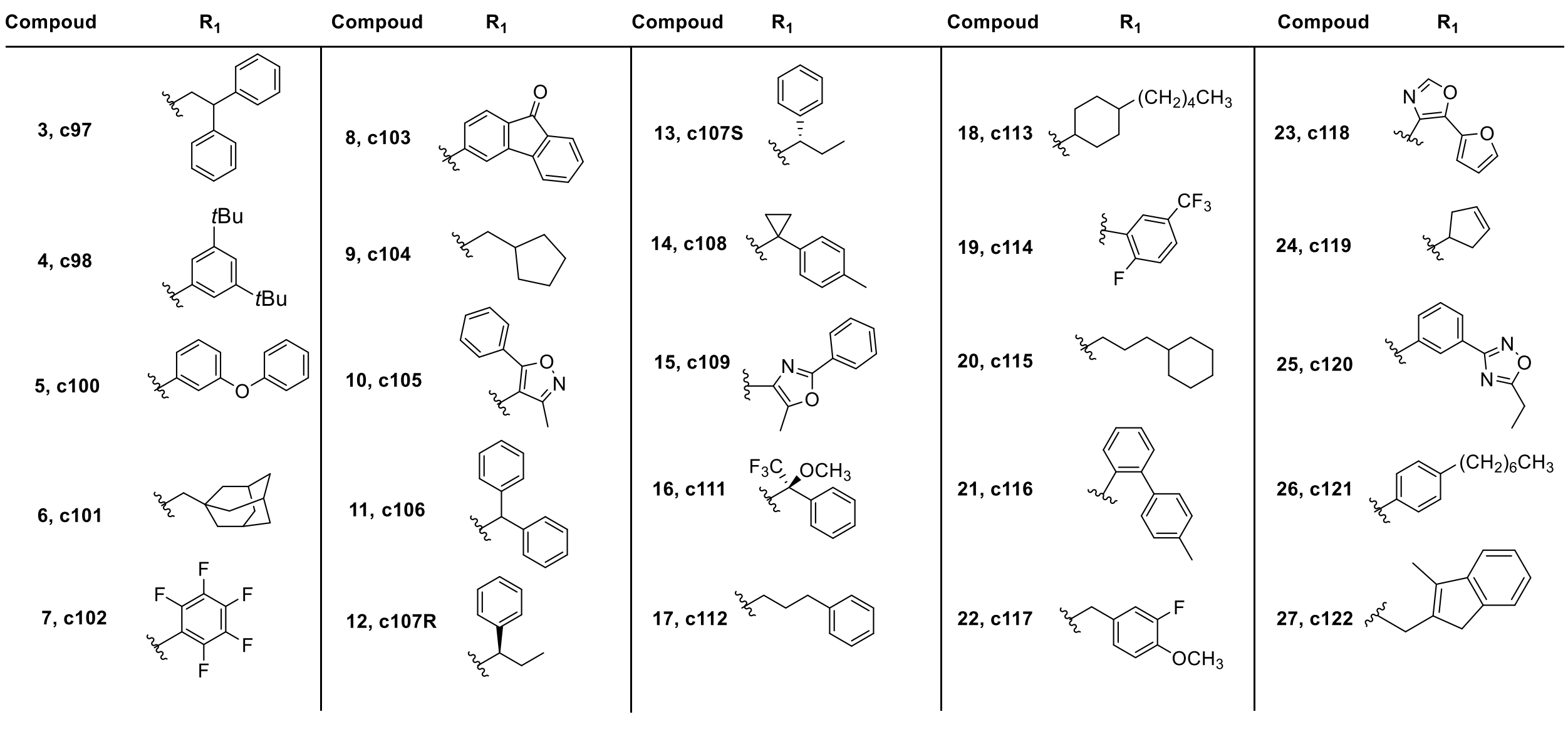

Derivative **c106F** was synthesized as depicted in **Scheme S2**. The Buchwald reaction between 2,3-difluoro-6-nitroaniline and 4-(trifluoromethyl)benzylamine led to the intermediate **28** (76% yield), that was subjected to a one-pot reaction using Zn dust and NH_4Cl to obtain the corresponding amine intermediate and coupled with 2,2-diphenylacetyl chloride, furnishing the final compound **c106F** (64% yield).

Scheme S2. Synthesis of derivative **c106F**.

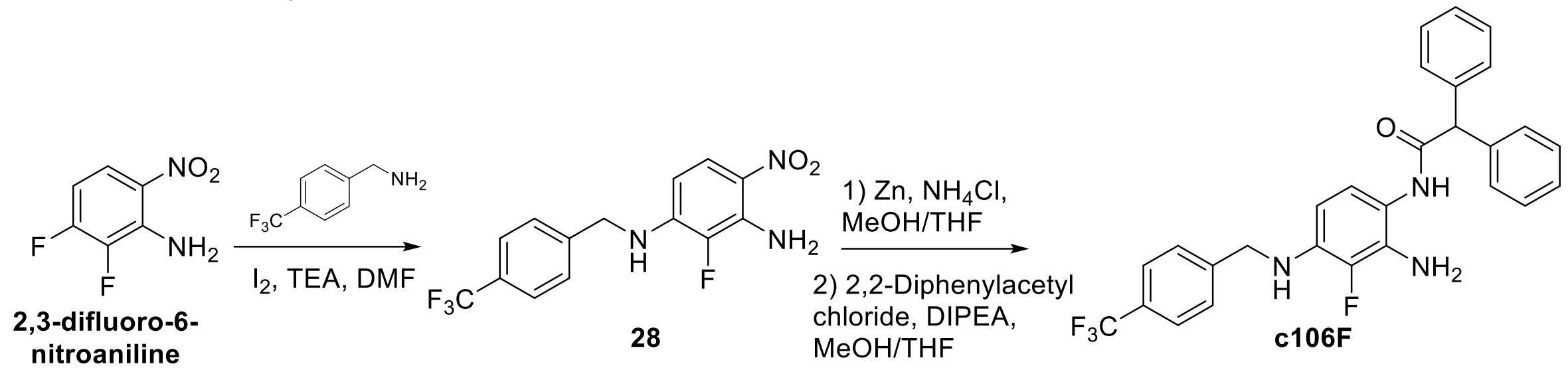

1.3 SYNTHETIC EXPERIMENTAL PROCEDURES

General procedure A: N-acylation
Intermediate **2** (1.0 eq) was dissolved in THF and added with 1,8-diazabicyclo[5.4.0]undec-7-ene (1.2 eq), and the proper acyl chloride (1.2 eq). The reaction was subjected to microwave assisted closed vessel reaction for 1 hour at 120 °C. Then, the crude mixture was evaporated under *vacuum*, diluted with dichloromethane and washed with a saturated solution of $NaHCO_3$ (3 x 20 mL). The organic layer was dried over Na_2SO_4, concentrated *in vacuo* and purified by flash chromatography (*n*-hexane/ethyl acetate as mobile phase), yielding the corresponding acylated intermediates **3**-**27**.

General procedure B: Catalytic Hydrogenation
1.0 equivalent of the proper intermediate (**3-27**) was dissolved in a solution of THF/MeOH (ratio 1:1 v:v); then, ammonium formate (10 eq) and Pd/C (6% mol) were added, and the resulting solution was stirred at 100 °C until disappearance of the starting material, monitored by TLC. The crude layer was filtered through Celite 503 (Merck Millipore, Burlington, USA), concentrated *in vacuo* and reconstituted in ethyl acetate. Then, it was washed with a concentrated solution of K_2CO_3, filtrated and concentrated under vacuum. The purification was performed by flash chromatography, using mixtures of *n*-hexane/ethyl acetate as eluent.

Synthesis of 3-nitro-N1-(4-(trifluoromethyl)benzyl)benzene-1,4-diamine (1)
2-Nitro-1,4-phenylenediamine (**1**, 1.0 g, 6.5 mmol, 1.0 eq), was dissolved in DMF, and added with 1.2 equivalents of TEA (1.09 mL, 7.8 mmol) and 1.2 equivalents of 4-(trifluoromethyl)benzyl bromide (1.87 g, 7.8 mmol). The reaction mixture was stirred for 2 hours at room temperature. Then, the crude was diluted with dichloromethane and washed with a saturated solution of $NaHCO_3$ (3 x 20 mL), and brine (3 x 20 mL). The organic phase was extracted, dried over Na_2SO_4, filtered and concentrated under vacuum. The crude reaction was purified by flash chromatography using a mixture of *n*-hexane/ethyl acetate (ratio 7/3: v/v) as mobile phase. Yield 1.49 g, 75%, R_f = 0.45. ^{1}H NMR ($CDCl_3$, 400 MHz, ppm): δ: 7.63 (d, 2H, Ar-H, *J* = 8.1 Hz); 7.51 (d, 2H, Ar-H, *J* = 8.0); 7.29 (d, 1H, Ar-H, *J* = 2.7 Hz); 6.87 (dd, 1H, Ar-H, *J'* = 2.8 Hz and *J''* = 8.9 Hz); 6.73 (d, 1H, Ar-H, *J* = 8.8 Hz); 5.77 (bs, 2H, N*H*$_2$); 4.39 (s, 2H, C*H*$_2$). HR-MS *m/z*: calcd for $C_{14}H_{12}F_3N_3O_2$, $[(M+H)]^+$: 312.0974; found 312.0981.

Benzyl (3-amino-4-nitrophenyl)(4-(trifluoromethyl)benzyl)carbamate (2)
Intermediate **1** (1.49 g, 4.77 mmol, 1 eq) was dissolved in THF, then 1.2 equivalents of DIPEA (1.01 mL, 5.72 mmol) and 1.2 equivalents of benzyl chloroformate (0.814 mL, 5.72 mmol) were added. The obtained solution was stirred at room temperature until completion, monitored by TLC. Then, the crude reaction was diluted with DCM, washed with a saturated solution of $NaHCO_3$ (3 x 20 mL), dried, filtered and concentrated *in vacuo*. Flash chromatography of the crude mixtures using *n*-hexane/ethyl acetate (ratio 8/2: v/v) as eluent yielded the corresponding protected intermediate **2**. R_f = 0.49. Yellowish oil (1.76 g, 83% yield). ^{1}H NMR (400 MHz, $CDCl_3$, ppm): δ: 7.85 (bs, 1H, Ar-H); 7.46 (d, 2H, Ar-H, *J* = 8.0); 7.23-7.28 (m, 6H, Ar-H); 7.15-7.18 (m, 2H, Ar-H);

6.62 (d, 1H, Ar-H, *J* = 8.8 Hz); 5.10 (s, 2H, C*H*$_2$); 4.79 (s, 2H, C*H*$_2$). HR-MS *m/z*: calcd for $C_{22}H_{18}F_3N_3O_4$, [(M+H)]$^+$: 446.1322; found 446.1328.

Benzyl (4-(3,3-diphenylpropanamido)-3-nitrophenyl)(4-(trifluoromethyl)benzyl)carbamate (3)
Obtained from **2** (0.350 g, 0.78 mmol) and 3,3-diphenylpropanoyl chloride (0.23 g, 0.94 mmol) applying the general procedure A. R_f = 0.42 (*n*-hexane/ethyl acetate 9/1). Yellowish oil (0.300 g, 59% yield). ^{1}H NMR (400 MHz, CDCl$_3$, ppm): δ: 8.53 (d, 1H, Ar-H, *J* = 8.5 Hz); 8.24 (s, 1H); 7.58 (d, 2H, Ar-H, *J* = 8.2 Hz); 7.51 (d, 2H, Ar-H, *J* = 8.1 Hz); 7.32-7.37 (m, 7H, Ar-H); 7.18-7.25 (m, 7H, Ar-H); 6.59 (d, 1H, Ar-H, *J* = 8.6 Hz); 5.01 (s, 2H, C*H*$_2$); 4.66 (t, 1H, C*H*, *J* = 8.1 Hz); 4.35 (s, 2H, C*H*$_2$); 3.14 (d, 2H, C*H*$_2$, *J* = 8.1 Hz). HR-MS *m/z* calcd for $C_{37}H_{30}F_3N_3O_5$ [(M - H)]$^-$: 652.2065; found 652.2077.

Benzyl (4-(3,5-di-*tert*-butylbenzamido)-3-nitrophenyl)(4-(trifluoromethyl)benzyl)carbamate (4)
Intermediate **4** was synthesized from **2** (0.190 g, 0.426 mmol) and 3,5-di-*tert*-butylbenzoyl chloride (0.13 g, 0.52 mmol), according to general procedure A. R_f = 0.49 (*n*-hexane/ethyl acetate 4/1). Yellowish oil (0.177 g, 63% yield). ^{1}H NMR (400 MHz, CDCl$_3$, ppm): δ: 11.40 (s, 1H, N*H*); 9.04 (d, 1H, Ar-H, *J* = 9.2 Hz); 8.16 (bs, 1H, Ar-H); 7.86 (d, 2H, Ar-H, *J* = 1.7 Hz); 7.72 (t, 2H, Ar-H, *J* = 1.6 Hz); 7.59 (d, 2H, Ar-H, *J* = 8.1 Hz); 7.35-7.38 (m, 5H, Ar-H); 7.28-7.30 (m, 2H, Ar-H); 5.24 (s, 2H, C*H*$_2$); 5.01 (s, 2H, C*H*$_2$); 1.43 (s, 18H, C*H*$_3$). HR-MS *m/z*: calcd for $C_{37}H_{38}F_3N_3O_5$, [(M-H)]$^-$: 660.2691; found 660.2697.

Benzyl (3-nitro-4-(3-phenoxybenzamido)phenyl)(4-(trifluoromethyl)benzyl)carbamate (5)
Intermediate **5** was obtained from **2** (0.185 g, 0.415 mmol) and 3-phenoxybenzoyl chloride (0.116 g, 0.498 mmol), according to general procedure A. R_f = 0.40 (*n*-hexane/ethyl acetate 4/1). Yellowish oil (0.157 g, 58% yield). ^{1}H NMR (400 MHz, CDCl$_3$, ppm): δ: 11.24 (s, 1H, N*H*); 8.93 (d, 1H, Ar-H, *J* = 9.2 Hz); 8.12 (bs, 1H, Ar-H); 7.66 (d, 1H, Ar-H, *J* = 7.8 Hz); 7.62 (t, 1H, Ar-H, *J* = 1.8 Hz); 7.58 (d, 2H, Ar-H, *J* = 8.1 Hz); 7.51 (t, 2H, Ar-H, *J* = 7.9 Hz); 7.41 (t, 2H, Ar-H, *J* = 7.5 Hz); 7.33-7.37 (m, 5H, Ar-H); 7.24-7.28 (m, 3H, Ar-H); 7.19 (t, 1H, Ar-H, *J* = 7.4 Hz); 7.07-7.10 (m, 2H, Ar-H); 5.23 (s, 2H, C*H*$_2$); 4.99 (s, 2H, C*H*$_2$). HR-MS *m/z*: calcd for $C_{35}H_{26}F_3N_3O_6$, [(M-H)]$^-$: 640.1701; found 640.1724.

Benzyl (4-(2-((3r,5r,7r)-adamantan-1-yl)acetamido)-3-nitrophenyl)(4-(trifluoromethyl)benzyl)carbamate (6)
Intermediate **6** was obtained from **2** (0.183 g, 0.411 mmol) and 2-((3r,5r,7r)-adamantan-1-yl)acetyl chloride (0.104 g, 0.493 mmol), according to general procedure A. R_f = 0.45 (*n*-hexane/ethyl acetate 4/1). Yellowish oil (0.150 g, 59% yield). ^{1}H NMR (400 MHz, CDCl$_3$, ppm): δ: 10.25 (s, 1H, N*H*); 8.81 (d, 1H, Ar-H, *J* = 9.1 Hz); 8.06 (bs, 1H, Ar-H); 7.57 (d, 2H, Ar-H, *J* = 8.2 Hz); 7.42 (d, 1H, Ar-H, *J* = 5.9 Hz); 7.32-7.37 (m, 4H, Ar-H); 7.25-7.28 (m, 3H, Ar-H); 5.21 (s, 2H, C*H*$_2$); 4.96 (s, 2H, C*H*$_2$); 1.25-1.30 (m, 3H, C*H* and C*H*$_2$); 2.21 (s, 2H, C*H*$_2$); 2.01 (bs, 3H, C*H* and C*H*$_2$); 1.65-1.75 (m, 9H, C*H* and C*H*$_2$). HR-MS *m/z*: calcd for $C_{34}H_{34}F_3N_3O_5$, [(M-H)]$^-$: 620.2378; found 620.2388.

Benzyl (3-nitro-4-(peRfluorobenzamido)phenyl)(4-(trifluoromethyl)benzyl)carbamate (7)
Intermediate **7** was obtained from **2** (0.190 g, 0.426 mmol) and 2,3,4,5,6-pentafluorobenzoyl chloride (0.074 mL, 0.511 mmol), according to general procedure A. R_f = 0.60 (*n*-hexane/ethyl acetate 4/1). Yellowish oil (0.188 g, 69% yield). ^{1}H NMR (400 MHz, CDCl$_3$, ppm): δ: 10.88 (s, 1H, N*H*); 8.84 (d, 1H, Ar-H, *J* = 9.1 Hz); 8.16 (bs, 1H, Ar-H); 7.59 (d, 2H, Ar-H, *J* = 8.1 Hz); 7.54 (d, 1H, Ar-H, *J* = 8.7 Hz); 7.33-7.38 (m, 5H, Ar-H); 7.27-7.29 (m, 2H, Ar-H); 5.24 (s, 2H, C*H*$_2$); 5.01 (s, 2H, C*H*$_2$). HR-MS *m/z*: calcd for $C_{29}H_{17}F_8N_3O_5$, [(M-H)]$^-$: 638.0968; found 638.0973.

Benzyl (3-nitro-4-(9-oxo-9H-fluorene-3-carboxamido)phenyl)(4-(trifluoromethyl)benzyl)carbamate (8)
Intermediate **8** was obtained from **2** (0.180 g, 0.404 mmol) and 9-oxo-9H-fluorene-3-carbonyl chloride (0.118 g, 0.485 mmol), according to general procedure A. R_f = 0.40 (*n*-hexane/ethyl acetate 4/1). Yellowish oil (0.166 g, 63% yield). ^{1}H NMR (400 MHz, CDCl$_3$, ppm): δ: 10.89 (s, 1H,

N*H*); 9.02 (d, 1H, Ar-H, *J* = 9.1 Hz); 8.17 (bs, 1H, Ar-H); 7.85-7.88 (m, 2H, Ar-H); 7.71-7.73 (m, 2H, Ar-H); 7.55-7.61 (m, 3H, Ar-H); 7.44-7.49 (m, 2H, Ar-H); 7.33-7.38 (m, 6H, Ar-H); 7.29-7.31 (m, 1H, Ar-H); 7.25-7.27 (m, 1H, Ar-H); 5.26 (s, 2H, C*H*$_2$); 5.03 (s, 2H, C*H*$_2$). HR-MS *m/z*: calcd for $C_{36}H_{24}F_3N_3O_6$, $[(M-H)]^-$: 650.1544; found 650.1564.

Benzyl (4-(2-cyclopentylacetamido)-3-nitrophenyl)(4-(trifluoromethyl)benzyl)carbamate (9)
Intermediate **9** was obtained from **2** (0.175 g, 0.393 mmol) and 2-cyclopentylacetyl chloride (0.064 mL, 0.472 mmol), according to general procedure A. R_f = 0.52 (*n*-hexane/ethyl acetate 4/1). Yellowish oil (0.157 g 72% yield). ^{1}H NMR (400 MHz, $CDCl_3$, ppm): δ: 10.31 (s, 1H, N*H*); 8.79 (d, 1H, Ar-H, *J* = 9.1 Hz); 8.06 (bs, 1H, Ar-H); 7.57 (d, 2H, Ar-H, *J* = 8.1 Hz); 7.32-7.36 (m, 5H, Ar-H); 7.25-7.28 (m, 3H, Ar-H); 5.22 (s, 2H, C*H*$_2$); 4.96 (s, 2H, C*H*$_2$); 2.50 (d, 2H, C*H*$_2$, *J* = 7.5 Hz); 2.22-2.30 (m, 1H, C*H*); 1.84-1.95 (m, 2H, C*H*$_2$); 1.56-1.71 (m, 4H, C*H*$_2$); 1.16-1.30 (m, 2H, C*H*$_2$). HR-MS *m/z*: calcd for $C_{29}H_{28}F_3N_3O_5$, $[(M-H)]^-$: 554.1908; found 554.1956.

Benzyl (4-(3-methyl-5-phenylisoxazole-4-carboxamido)-3-nitrophenyl)(4-(trifluoromethyl)benzyl)carbamate (10)
Intermediate **10** was obtained from **2** (0.200 g, 0.449 mmol) and 3-methyl-5-phenylisoxazole-4-carbonyl chloride (0.119 g, 0.539 mmol), according to general procedure A. Rf = 0.47 (*n*-hexane/ethyl acetate 3/1). Yellowish oil (0.155 g, 55% yield). ^{1}H NMR (400 MHz, $CDCl_3$, ppm): δ: 9.88 (s, 1H, N*H*); 8.66 (d, 1H, Ar-H, *J* = 9.1 Hz); 7.87 (bs, 1H, Ar-H); 7.53-7.55 (m, 2H, Ar-H); 7.47 (d, 2H, Ar-H, *J* = 8.1 Hz); 7.39-7.44 (m, 4H, Ar-H); 7.20-7.26 (m, 5H, Ar-H); 7.15-7.17 (m, 2H, Ar-H); 5.11 (s, 2H, C*H*$_2$); 4.85 (s, 2H, C*H*$_2$); 2.68 (s, 3H, C*H*$_3$). HR-MS *m/z*: calcd for $C_{33}H_{25}F_3N_4O_6$, $[(M+H)]^+$: 631.1799; found 631.1806.

Benzyl (4-(2,2-diphenylacetamido)-3-nitrophenyl)(4-(trifluoromethyl)benzyl)carbamate (11)
Intermediate **11** was obtained from **2** (0.150 g, 0.337 mmol) and 2,2-diphenylacetyl chloride (0.093 g, 0.404 mmol), according to general procedure A. R_f = 0.52 (*n*-hexane/ethyl acetate 4/1). Yellowish oil (0.130 g, 60% yield). ^{1}H NMR (400 MHz, $CDCl_3$, ppm): δ: 10.32 (s, 1H, N*H*); 8.75 (d, 1H, Ar-H, *J* = 9.1 Hz); 7.92 (bs, 1H, Ar-H); 7.46 (d, 2H, Ar-H, *J* = 8.1 Hz); 7.28-7.32 (m, 4H, Ar-H); 7.19-7.26 (m, 12H, Ar-H); 7.14-7.16 (m, 2H, Ar-H); 5.10 (s, 2H, C*H*$_2$); 5.07 (s, 1H, C*H*); 4.84 (s, 2H, C*H*$_2$). HR-MS *m/z*: calcd for $C_{36}H_{28}F_3N_3O_5$, $[(M-H)]^-$: 638.1908; found 638.1922.

Benzyl (*R*)-(3-nitro-4-(2-phenylbutanamido)phenyl)(4-(trifluoromethyl)benzyl)carbamate (12)
Intermediate **12** was obtained from **2** (0.210 g, 0.472 mmol) and (*R*)-2-phenylbutanoyl chloride (0.094 mL, 0.566 mmol) according to general procedure A. R_f = 0.56 (*n*-hexane/ethyl acetate 4/1). Yellowish oil (0.162 g, 58% yield). ^{1}H NMR (400 MHz, $CDCl_3$, ppm): δ: 10.35 (s, 1H, N*H*); 8.80 (d, 1H, Ar-H, *J* = 9.2 Hz); 8.02 (bs, 1H, Ar-H); 7.58 (d, 2H, Ar-H, *J* = 8.0 Hz); 7.46-7.40 (m, 4H, Ar-H); 7.39-7.36 (m, 4H, Ar-H); 7.34-7.27 (m, 5H, Ar-H); 5.22 (s, 2H, C*H*$_2$); 4.96 (s, 2H, C*H*$_2$); 3.54 (t, 1H, C*H*, *J* = 7.6 Hz); 2.27-2.38 (m, 1H, C*H*); 1.94-2.01 (m, 1H, C*H*); 0.99 (t, 3H, C*H*$_3$, *J* = 7.3 Hz). HR-MS *m/z*: calcd for $C_{32}H_{28}F_3N_3O_5$, $[(M-H)]^-$: 590.1908; found 590.1915.

Benzyl (*S*)-(3-nitro-4-(2-phenylbutanamido)phenyl)(4-(trifluoromethyl)benzyl)carbamate (13)
Intermediate **13** was obtained from **2** (0.200 g, 0.449 mmol) and (*S*)-2-phenylbutanoyl chloride (0.089 mL, 0.538 mmol), according to general procedure A. R_f = 0.56 (*n*-hexane/ethyl acetate 4/1). Yellowish oil (0.143 g, 54% yield). ^{1}H NMR (400 MHz, $CDCl_3$, ppm): δ: 10.33 (s, 1H, N*H*); 8.78 (d, 1H, Ar-H, *J* = 9.2 Hz); 8.01 (bs, 1H, Ar-H); 7.56 (d, 2H, Ar-H, *J* = 8.0 Hz); 7.24-7.43 (m, 13H, Ar-H); 5.20 (s, 2H, C*H*$_2$); 4.94 (s, 2H, C*H*$_2$); 3.52 (t, 1H, C*H*, *J* = 7.4 Hz); 2.25-2.34 (m, 1H, C*H*); 1.91-1.99 (m, 1H, C*H*); 0.97 (t, 3H, C*H*$_3$, *J* = 7.4 Hz). HR-MS *m/z*: calcd for $C_{32}H_{28}F_3N_3O_5$, $[(M-H)]^-$: 590.1908; found 590.1922.

Benzyl (3-nitro-4-(1-(p-tolyl)cyclopropane-1-carboxamido)phenyl)(4-(trifluoromethyl)benzyl)carbamate (14)
Intermediate **14** was synthesized from **2** (0.195 g, 0.438 mmol) and 1-(p-tolyl)cyclopropane-1-carbonyl chloride (0.103 g, 0.525 mmol), according to general procedure A. R_f = 0.42 (*n*-

hexane/ethyl acetate 3/1). Yellowish oil (0.187 g, 71% yield). ^{1}H NMR (400 MHz, CD_3OD, ppm): δ: 8.46 (d, 1H, Ar-H, *J* = 9.1 Hz); 7.87 (bs, 1H, Ar-H); 7.46 (d, 2H, Ar-H, *J* = 8.1 Hz); 7.39 (d, 1H, Ar-H, *J* = 7.6 Hz); 7.27 (d, 4H, Ar-H, *J* = 8.1 Hz); 7.12-7.19 (m, 7H, Ar-H); 5.06 (s, 2H, C*H*$_2$); 4.88 (s, 2H, C*H*$_2$); 1.50-1.53 (m, 2H, C*H*$_2$); 1.08-1.11 (m, 2H, C*H*$_2$). HR-MS *m/z*: calcd for $C_{33}H_{28}F_3N_3O_5$, $[(M-H)]^-$: 602.1908; found 602.1923.

Benzyl (4-(5-methyl-2-phenyloxazole-4-carboxamido)-3-nitrophenyl)(4-(trifluoromethyl)benzyl)carbamate (15)

Intermediate **15** was synthesized from **2** (0.186 g, 0.418 mmol) and 5-methyl-2-phenyloxazole-4-carbonyl chloride (0.111 g, 0.502 mmol), according to general procedure A. R_f = 0.53 (*n*-hexane/ethyl acetate 3/1). Yellowish oil (0.161 g, 61% yield). ^{1}H NMR (400 MHz, CD_3OD, ppm): δ: 8.96 (d, 1H, Ar-H, *J* = 9.1 Hz); 8.11-8.13 (m, 2H, Ar-H); 7.58 (d, 2H, Ar-H, *J* = 8.1 Hz); 7.52 (t, 3H, Ar-H, *J* = 3.4 Hz); 7.47 (bs, 1H, Ar-H); 7.35-7.36 (m, 5H, Ar-H); 7.26-7.28 (m, 3H, Ar-H); 5.23 (s, 2H, C*H*$_2$); 4.99 (s, 2H, C*H*$_2$); 2.80 (s, 3H, C*H*$_3$). HR-MS *m/z*: calcd for $C_{33}H_{25}F_3N_4O_6$, $[(M-H)]^-$: 629.1653; found 629.1685.

Benzyl (*S*)-(3-nitro-4-(3,3,3-trifluoro-2-methoxy-2-phenylpropanamido)phenyl)(4-(trifluoromethyl)benzyl)carbamate (16)

Intermediate **16** was synthesized from **2** (0.200 g, 0.449 mmol) and (*R*)-3,3,3-trifluoro-2-methoxy-2-phenylpropanoyl chloride (0.101 mL, 0.538 mmol), according to general procedure A. R_f = 0.54 (*n*-hexane/ethyl acetate 3/1). Yellowish oil (0.163 g, 55% yield). ^{1}H NMR (400 MHz, CD_3OD, ppm): δ: 8.82 (d, 1H, Ar-H, *J* = 9.1 Hz); 8.15 (bs, 1H, Ar-H); 7.63 (d, 2H, Ar-H, *J* = 3.8 Hz); 7.59 (d, 2H, Ar-H, *J* = 8.1 Hz); 7.47 (t, 4H, Ar-H, *J* = 2.8 Hz); 7.28-7.38 (m, 7H, Ar-H); 5.25 (s, 2H, C*H*$_2$); 5.00 (s, 2H, C*H*$_2$); 3.59 (s, 3H, C*H*$_3$). HR-MS *m/z*: calcd for $C_{32}H_{25}F_6N_3O_6$, $[(M-H)]^-$: 660.1575; found 660.1588.

Benzyl (3-nitro-4-(4-phenylbutanamido)phenyl)(4-(trifluoromethyl)benzyl)carbamate (17)

Intermediate **17** was synthesized from **2** (0.200 g, 0.449 mmol) and 4-phenylbutanoyl chloride (0.088 mL, 0.538 mmol), according to general procedure A. R_f = 0.53 (*n*-hexane/ethyl acetate 3/1). Yellowish oil (0.183 g, 69% yield). ^{1}H NMR (400 MHz, $CDCl_3$, ppm): δ: 10.27 (s, 1H, N*H*); 8.76 (d, 1H, Ar-H, *J* = 9.2 Hz); 8.07 (bs, 1H, Ar-H); 7.58 (d, 2H, Ar-H, *J* = 8.1 Hz); 7.43 (d, 1H, Ar-H, *J* = 7.4 Hz); 7.31-7.36 (m, 6H, Ar-H); 7.26-7.29 (m, 3H, Ar-H); 7.22 (d, 3H, Ar-H, *J* = 7.5 Hz); 5.22 (s, 2H, C*H*$_2$); 4.97 (s, 2H, C*H*$_2$); 2.75 (t, 2H, C*H*$_2$, *J* = 7.6 Hz); 2.51 (t, 2H, C*H*$_2$, *J* = 7.4 Hz); 2.07-2.15 (m, 2H, C*H*$_2$). HR-MS *m/z*: calcd for $C_{32}H_{28}F_3N_3O_5$, $[(M-H)]^-$: 590.1908; found 590.1936.

Benzyl (3-nitro-4-(4-pentylcyclohexane-1-carboxamido)phenyl)(4-(trifluoromethyl)benzyl)carbamate (18)

Intermediate **18** was synthesized from **2** (0.150 g, 0.337 mmol) and 4-pentylcyclohexane-1-carbonyl chloride (0.087 mL, 0.404 mmol), according to general procedure A. R_f = 0.59 (*n*-hexane/ethyl acetate 3/1). Yellowish oil (0.137 g, 65% yield). ^{1}H NMR (400 MHz, $CDCl_3$, ppm): δ: 0.79-0.83 (m, 3H, C*H*$_3$); 0.86-0.96 (m, 2H, C*H*$_2$); 1.10-1.25 (m, 8H, C*H*$_2$); 10.29 (s, 1H, N*H*); 8.69 (d, 1H, Ar-H, *J* = 9.2 Hz); 7.95 (bs, 1H, Ar-H); 7.47 (d, 2H, Ar-H, *J* = 8.1 Hz); 7.32 (d, 1H, Ar-H, *J* = 7.0 Hz); 7.21-7.27 (m, 5H, Ar-H); 7.15-7.17 (m, 2H, Ar-H); 5.11 (s, 2H, C*H*$_2$); 4.86 (s, 2H, C*H*$_2$); 2.17-2.25 (m, 1H, C*H*); 1.91-1.98 (m, 2H, C*H*$_2$); 1.72-1.82 (m, 3H, C*H* and C*H*$_2$); 1.39-1.50 (m, 2H, C*H*$_2$). HR-MS *m/z*: calcd for $C_{34}H_{38}F_3N_3O_5$, $[(M-H)]^-$: 624.2691; found 624.2699.

Benzyl (4-(2-fluoro-5-(trifluoromethyl)benzamido)-3-nitrophenyl)(4-(trifluoromethyl)benzyl)carbamate (19)

Intermediate **19** was synthesized from **2** (0.200 g, 0.449 mmol) and 2-fluoro-5-(trifluoromethyl)benzoyl chloride (0.122 g, 0.538 mmol), according to general procedure A. R_f = 0.56 (*n*-hexane/ethyl acetate 3/1). Yellowish oil (0.188 g, 66% yield). ^{1}H NMR (400 MHz, $CDCl_3$, ppm): δ: 11.46 (s, 1H, N*H*); 8.91 (d, 1H, Ar-H, *J* = 9.1 Hz); 8.47 (dd, 1H, Ar-H, *J'* = 2.1 Hz and *J''* = 6.8 Hz); 8.14 (bs, 1H, Ar-H); 7.85-7.89 (m, 1H, Ar-H); 7.59 (d, 2H, Ar-H, *J* = 8.1 Hz); 7.53 (d, 1H, Ar-H, *J* = 8.1 Hz); 7.40-7.43 (m, 1H, Ar-H); 7.34-7.37 (m, 5H, Ar-H); 7.27-7.29 (m, 2H, Ar-H); 5.24

(s, 2H, CH_2); 5.01 (s, 2H, CH_2). HR-MS *m/z*: calcd for $C_{30}H_{20}F_7N_3O_5$, $[(M-H)]^-$: 634.1218; found 634.1238.

Benzyl (4-(4-cyclohexylbutanamido)-3-nitrophenyl)(4-(trifluoromethyl)benzyl)carbamate (20)
Intermediate **20** was synthesized from **2** (0.200 g, 0.449 mmol) and 4-cyclohexylbutanoyl chloride (0.085 g, 0.538 mmol), according to general procedure A. R_f = 0.55 (*n*-hexane/ethyl acetate 3/1). Yellowish oil (0.187 g, 73% yield). ^{1}H NMR (400 MHz, $CDCl_3$, ppm): δ: 10.31 (s, 1H, N*H*); 8.78 (d, 1H, Ar-H, *J* = 9.1 Hz); 8.06 (bs, 1H, Ar-H); 7.57 (d, 2H, Ar-H, *J* = 8.1 Hz); 7.43 (d, 1H, Ar-H, *J* = 7.3 Hz); 7.32-7.36 (m, 4H, Ar-H); 7.25-7.27 (m, 3H, Ar-H); 5.21 (s, 2H, CH_2); 4.96 (s, 2H, CH_2); 2.47 (t, 2H, CH_2, *J* = 7.6 Hz); 1.65-1.81 (m, 9H, C*H* and CH_2); 0.90-0.95 (m, 2H, CH_2); 1.23-1.30 (m, 4H, CH_2). HR-MS *m/z*: calcd for $C_{32}H_{34}F_3N_3O_5$, $[(M-H)]^-$: 596.2378; found 596.2382.

Benzyl (4-(4'-methyl-[1,1'-biphenyl]-2-carboxamido)-3-nitrophenyl)(4-(trifluoromethyl)benzyl)carbamate (21)
Synthetized from **2** (0.200 g, 0.449 mmol) and 4'-methylbiphenyl-2-carbonyl chloride (0.124 g, 0.538 mmol), applying the general procedure A. R_f = 0.38 (*n*-hexane/ethyl acetate 9/1). Yellowish oil (0.186 g, 65% yield). ^{1}H NMR (400 MHz, CD_3OD, ppm): δ: 7.93 (s, 1H, Ar-H); 7.70 (d, 1H, Ar-H, *J* = 8.0 Hz); 7.60-7.62 (m, 3H, Ar-H); 7.50 (t, 2H, Ar-H, *J* = 6.6 Hz); 7.42 (d, 3H, Ar-H, *J* = 7.9 Hz); 7.27-7.33 (m, 7H, Ar-H); 7.13 (d, 2H, Ar-H, *J* = 7.3 Hz); 6.90 (d, 1H, Ar-H, *J* = 8.9 Hz); 6.60 (d, 1H, Ar-H, *J* = 8.8 Hz); 5.21 (s, 2H, CH_2); 5.04 (s, 2H, CH_2); 2.30 (s, 3H, CH_3). HR-MS *m/z* calcd for $C_{36}H_{28}F_3N_3O_5$ $[(M-H)]^-$: 638.1908; found 638.1915.

Benzyl (4-(2-(3-fluoro-4-methoxyphenyl)acetamido)-3-nitrophenyl)(4-(trifluoromethyl)benzyl)carbamate (22)
Intermediate **22** was synthesized from **2** (0.200 g, 0.449 mmol) and 2-(3-fluoro-4-methoxyphenyl)acetyl chloride (0.109 g, 0.538 mmol), according to general procedure A. R_f = 0.57 (*n*-hexane/ethyl acetate 4/1). Yellowish oil (0.151 g, 55% yield). ^{1}H NMR (400 MHz, $CDCl_3$, ppm): 11.55 (s, 1H, N*H*); 8.69 (d, 1H, Ar-H, *J* = 9.1 Hz); 8.12 (bs, 1H, Ar-H); 7.54-7.58 (m, 4H, Ar-H); 7.31-7.36 (m, 4H, Ar-H); 7.25-7.28 (m, 3H, Ar-H); 7.01 (t, 2H, Ar-H, *J* = 8.4 Hz); 5.22 (s, 2H, CH_2); 4.98 (s, 2H, CH_2); 3.94 (s, 5H, CH_2 and CH_3). HR-MS *m/z*: calcd for $C_{31}H_{25}F_4N_3O_6$, $[(M-H)]^-$: 610.1607; found 610.1623.

Benzyl (4-(5-(furan-2-yl)oxazole-4-carboxamido)-3-nitrophenyl)(4-(trifluoromethyl)benzyl)carbamate (23)
Intermediate **23** was synthesized from **2** (0.200 g, 0.449 mmol) and 5-(furan-2-yl)oxazole-4-carbonyl chloride (0.106 g, 0.538 mmol), according to general procedure A. R_f = 0.44 (*n*-hexane/ethyl acetate 3/1). Yellowish oil (0.131 g, 48% yield). ^{1}H NMR (400 MHz, $CDCl_3$, ppm): 8.96 (d, 1H, Ar-H, *J* = 9.1 Hz); 8.13 (bs, 1H, Ar-H); 7.93 (s, 1H, Ar-H); 7.87 (d, 1H, Ar-H, *J* = 3.5 Hz); 7.66 (d, 1H, Ar-H, *J* = 1.4 Hz); 7.58 (d, 2H, Ar-H, *J* = 8.1 Hz); 7.47 (d, 1H, Ar-H, *J* = 6.6 Hz); 7.34-7.36 (m, 5H, Ar-H); 7.26-7.28 (m, 1H, Ar-H); 6.64 (dd, 1H, Ar-H, *J'* = 1.8 Hz and *J''* = 3.6 Hz); 5.23 (s, 2H, CH_2); 4.99 (s, 2H, CH_2). HR-MS *m/z*: calcd for $C_{30}H_{21}F_3N_4O_7$, $[(M+H)]^+$: 607.1435; found 607.1444.

Benzyl (4-(cyclopent-3-ene-1-carboxamido)-3-nitrophenyl)(4-(trifluoromethyl)benzyl)carbamate (24)
Intermediate **24** was synthetized from **2** (0.200 g, 0.449 mmol) and cyclopent-3-ene-1-carbonyl chloride (0.070 g, 0.538 mmol), according to the general procedure A. R_f = 0.46 (*n*-hexane/ethyl acetate 8/2). Yellowish oil (0.164 g, 68% yield). ^{1}H NMR (400 MHz, CD_3OD, ppm): δ: 7.48 (d, 2H, Ar-H, *J* = 8.0 Hz); 7.27 (d, 2H, Ar-H, *J* = 8.0 Hz); 6.99 (d, 1H, Ar-H, *J* = 8.4 Hz); 6.50 (d, 1H, Ar-H, *J* = 2.6 Hz); 6.44 (dd, 1H, Ar-H, *J'* = 2.0 Hz and *J''* = 8.4 Hz); 5.62 (s, 2H, CH_2); 4.72 (s, 2H, CH_2); 4.68 (s, 2H, CH_2); 3.00-3.11 (m, 1H, C*H*); 2.62 (dd, 4H, CH_2, *J'* = 3.1 Hz and *J''* = 5.8 Hz). HR-MS *m/z* calcd for $C_{28}H_{24}F_3N_3O_5$ $[(M-H)]^-$: 538.1595; found 538.1566.

Benzyl (4-(3-(5-ethyl-1,2,4-oxadiazol-3-yl)benzamido)-3-nitrophenyl)(4-(trifluoromethyl)benzyl)carbamate (25)
Intermediate **25** was obtained from **2** (0.200 g, 0.449 mmol) and 3-(5-ethyl-1,2,4-oxadiazol-3-yl)benzoyl chloride (0.128 g, 0.538 mmol), according to the general procedure A. R_f = 0.40 (*n*-hexane/ethyl acetate 9/1). Yellowish oil (0.182 g, 63% yield). ^{1}H NMR (400 MHz, $CDCl_3$, ppm): δ: 11.3 (s, 1H, N*H*); 8.97 (d, 1H, Ar-H, *J* = 9.1 Hz); 8.72 (s, 1H, Ar-H); 8.34 (d, 1H, Ar-H, *J* = 6.6 Hz); 8.15 (bs, 1H, Ar-H); 8.10 (d, 1H, Ar-H, *J* = 8.1 Hz); 7.69 (t, 1H, Ar-H, *J* = 7.8 Hz); 7.51-7.60 (m, 3H, Ar-H); 7.34-7.36 (m, 5H, Ar-H); 7.27-7.29 (m, 1H, Ar-H); 5.24 (s, 2H, CH_2); 5.00 (s, 2H, CH_2); 3.03 (dd, 1H, CH_2, *J'* = 7.6 Hz and *J''* = 15.0 Hz); 1.50 (t, 3H, CH_3, *J* = 7.6 Hz). HR-MS *m/z* calcd for $C_{33}H_{26}F_3N_5O_6$ $[(M+H)]^+$: 646.1908; found 646.1932.

Benzyl (4-(4-heptylbenzamido)-3-nitrophenyl)(4-(trifluoromethyl)benzyl)carbamate (26)
Intermediate **26** was synthesized from **2** (0.200 g, 0.449 mmol) and 4-heptylbenzoyl chloride (0.128 g, 0.538 mmol), according to general procedure A. R_f = 0.40 (*n*-hexane/ethyl acetate 2/1). Yellowish oil (0.200 g, 69% yield). ^{1}H NMR (400 MHz, $CDCl_3$, ppm): δ: 11.27 (s, 1H, N*H*); 8.99 (d, 1H, Ar-H, *J* = 9.1 Hz); 8.12 (bs, 1H, Ar-H); 7.90 (d, 2H, Ar-H, *J* = 8.2 Hz); 7.58 (d, 2H, Ar-H, *J* = 8.1 Hz); 7.49 (d, 1H, Ar-H, *J* = 8.2 Hz); 7.34-7.37 (m, 7H, Ar-H); 7.27-7.28 (m, 2H, Ar-H); 5.23 (s, 2H, CH_2); 4.99 (s, 2H, CH_2); 2.71 (t, 2H, CH_2, *J* = 7.8 Hz); 1.64-1.69 (m, 2H, CH_2); 1.30-1.36 (m, 8H, CH_2); 0.91 (t, 3H, CH_3, *J* = 7.0 Hz). HR-MS *m/z*: calcd for $C_{36}H_{36}F_3N_3O_5$, $[(M-H)]^-$: 646.2534; found 646.2543.

Benzyl (4-(2-(2-methyl-1H-inden-3-yl)acetamido)-3-nitrophenyl)(4-(trifluoromethyl)benzyl)carbamate (27)
Intermediate **27** was synthetized from **2** (0.200 g, 0.449 mmol) and 2-(2-methyl-1H-inden-3-yl)acetyl chloride (0.111 g, 0.538 mmol), according to the general procedure A. R_f = 0.46 (*n*-hexane/ethyl acetate 8/2). Yellowish oil (0.146 g, 53% yield). ^{1}H NMR (400 MHz, $CDCl_3$, ppm): δ: 10.4 (bs, 1H, N*H*); 8.73 (d, 2H, Ar-H, *J* = 9.1 Hz); 7.45 (d, 2H, Ar-H, *J* = 8.0 Hz); 7.23-7.27 (m, 3H, Ar-H); 7.19-7.21 (m, 2H, Ar-H); 7.14-7.16 (m, 4H, Ar-H); 6.86 (d, 1H, Ar-H, *J* = 2.3 Hz and *J''* = 9.0 Hz); 6.77 (t, 1H, Ar-H, *J* = 8.0 Hz); 4.83 (s, 2H, CH_2); 3.62 (s, 2H, CH_2); 3.42 (s, 2H, CH_2); 2.15 (s, 3H, CH_3). HR-MS *m/z* calcd for $C_{34}H_{28}F_3N_3O_5$ $[(M+H)]^+$: 616.2054; found 616.2067.

2-Fluoro-4-nitro-N1-(4-(trifluoromethyl)benzyl)benzene-1,3-diamine (28)
2,3-Difluoro-6-nitroaniline (0.500 g, 2.87 mmol, 1.0 eq), 4-(trifluoromethyl)benzylamine (0.491 mL, 3.44 mmol, 1.2 eq), DIPEA (0.610 mL, 3.44 mmol, 1.2 eq) and a catalytic amount of I_2 were dissolved in DMF. The solution was refluxed for 4 h at 170 °C, then reaction mixture was diluted with DCM, washed successively with a saturated solution of $Na_2S_2O_3$, K_2CO_3, and brine. The organic phase was extracted, dried over anhydrous Na_2SO_4, filtered, and concentrated *in vacuo*. The crude product was purified by flash chromatography using *n*-hexane/ethyl acetate (ratio 7:3, v:v) as mobile phase giving intermediate **28** in 76% yield (0.717 g). R_f = 0.44. Yellowish powder. ^{1}H NMR (400 MHz, CD_3OD, ppm): δ: 7.64 (dd, 1H, Ar-H, *J'* = 8.0 Hz, *J''* = 1.7 Hz); 7.53 (d, 2H, Ar-H, *J* = 8.0 Hz); 7.42 (d, 2H, Ar-H, *J* = 8.0 Hz); 5.95 (dd, 1H, Ar-H, *J'* = 1.5 Hz, *J''* = 8.1 Hz); 4.49 (s, 2H, CH_2). HR-MS *m/z*: calcd for $C_{14}H_{12}F_4N_3O_2$, $[(M+H)]^+$: 330.0866; found 330.0862.

N-(2-Amino-4-((4-(trifluoromethyl)benzyl)amino)phenyl)-3,3-diphenylpropanamide (c97)
Obtained from **3** (0.300 g, 0.459 mmol, 1 eq) applying the general procedure B. R_f = 0.39 (*n*-hexane/ethyl acetate 3/7). Off-white powder (0.123 g, 55% yield). ^{1}H NMR (400 MHz, CD_3OD, ppm): δ: 7.58 (d, 2H, Ar-H, *J* = 8.2 Hz); 7.51 (d, 2H, Ar-H, *J* = 8.1 Hz); 7.28-7.35 (m, 8H, Ar-H); 7.18-7.22 (m, 2H, Ar-H); 6.51 (d, 1H, Ar-H, *J* = 8.8 Hz); 5.96-5.99 (m, 2H, Ar-H); 4.58 (t, 1H, C*H*, *J* = 8.2 Hz); 4.35 (s, 2H, CH_2); 3.09 (d, 2H, CH_2, *J* = 8.2 Hz); ^{13}C NMR (100 MHz, CD_3OD, ppm): δ: 172.2, 148.3, 145.3, 143.7, 143.3, 128.2,127.6, 127.3, 126.2, 124.8 (q, $^3J_{C,F}$= 3.8 Hz), 113.1, 103.8, 99.9, 46.6, 42.1. HR-MS *m/z* calcd for $C_{29}H_{26}F_3N_3O$ $[(M+H)]^+$: 490.2101; found 490.2086.

N-(2-Amino-4-((4-(trifluoromethyl)benzyl)amino)phenyl)-3,5-di-*tert*-butylbenzamide (c98)
Final derivative **c98** was synthetized starting from **4** (0.177 g, 0.267 mmol, 1 eq), applying the

general procedure B. R_f = 0.40 (*n*-hexane/ethyl acetate 3/7). Off-white powder (0.076 g, 57% yield). ^{1}H NMR (400 MHz, CD_3OD, ppm): δ: 7.83 (d, 2H, Ar-H, *J* = 1.8 Hz); 7.68 (t, 1H, Ar-H, *J* = 1.7 Hz); 7.61 (d, 2H, Ar-H, *J* = 8.4 Hz); 7.57 (d, 2H, Ar-H, *J* = 8.4 Hz); 6.92 (d, 1H, Ar-H, *J* = 8.4 Hz); 6.19 (d, 1H, Ar-H, *J* = 2.4 Hz); 6.15 (dd, 1H, Ar-H, *J'* = 2.5 Hz and *J''* = 8.2 Hz); 4.42 (s, 2H, C*H₂*); 1.39 (s, 18H, C*H₃*); ^{13}C NMR (100 MHz, CD_3OD, ppm): δ: 168.8; 151.0, 148.2, 145.3, 143.2, 133.7, 127.4, 127.3, 125.5, 124.82 (q, $^{3}J_{C,F}$= 3.8 Hz), 124.4 (q, $^{1}J_{C,F}$= 269.2 Hz), 121.6, 114.5, 104.4, 100.9, 46.7, 30.4. HR-MS *m/z* calcd for $C_{29}H_{34}F_3N_3O$ [(M+H)]$^+$: 498.2727; found 498.2709.

N-(2-Amino-4-((4-(trifluoromethyl)benzyl)amino)phenyl)-3-phenoxybenzamide (c100)
Obtained from **5** (0.157 g, 0.244 mmol, 1 eq), according to the general procedure B. R_f = 0.44 (*n*-hexane/ethyl acetate 1/1). Off-white powder (0.075 g, 65% yield). ^{1}H NMR (400 MHz, CD_3OD, ppm): δ: 7.70 (d, 1H, Ar-H, *J* = 7.8 Hz); 7.58 (dd, 5H, Ar-H, *J* = 8.3 Hz); 7.48 (t, 1H, Ar-H, *J* = 7.9 Hz); 7.40 (t, 2H, Ar-H, *J* = 8.2 Hz); 7.14-7.20 (m, 2H, Ar-H); 7.05 (d, 1H, Ar-H, *J* = 7.8 Hz); 6.89 (d, 1H, Ar-H, *J* = 8.4 Hz); 6.16 (d, 1H, Ar-H, *J* = 2.4 Hz); 6.13 (dd, 1H, Ar-H, *J'* = 2.5 Hz and *J''* = 8.4 Hz); 4.41 (s, 2H, C*H₂*); ^{13}C NMR (100 MHz, CD_3OD, ppm): δ: 167.0, 157.7, 156.8, 148.3, 145.2, 143.2, 136.2, 129.7, 129.6, 128.4, 127.3, 127.27, 124.8 (q, $^{3}J_{C,F}$= 3.8 Hz), 123.5, 121.9, 121.4, 118.8, 117.5, 114.0, 100.3, 100.7, 46.7. HR-MS *m/z* calcd for $C_{27}H_{22}F_3N_3O_2$ [(M+H)]$^+$: 478.1737; found 478.1737.

2-((3r,5r,7r)-Adamantan-1-yl)-N-(2-amino-4-((4-(trifluoromethyl)benzyl)amino)phenyl)acetamide (c101)
Synthetized from **6** (0.150 g, 0.241 mmol, 1 eq), applying the general procedure B. R_f = 0.41 (*n*-hexane/ethyl acetate 1/1). Off-white powder (45% yield). ^{1}H NMR (400 MHz, CD_3OD, ppm): δ: 7.60 (d, 2H, Ar-H, *J* = 8.3 Hz); 7.54 (d, 2H, Ar-H, *J* = 8.2 Hz); 6.77 (d, 1H, Ar-H, *J* = 8.4 Hz); 6.12 (d, 1H, Ar-H, *J* = 2.4 Hz); 6.08 (dd, 1H, Ar-H, *J'* = 2.5 Hz and *J''* = 8.4 Hz); 4.39 (s, 2H, C*H₂*); 2.11 (s, 2H, C*H₂*); 1.99 (bs, 3H, C*H* and C*H₂*); 1.70-1.76 (m, 11H, C*H* and C*H₂*); ^{13}C NMR (100 MHz, CD_3OD, ppm): δ: 171.6, 148.0, 145.2, 142.9, 128.7, 127.3, 127.1 (q, $^{1}J_{C,F}$= 269.3 Hz), 126.9, 125.8 (q, $^{3}J_{C,F}$= 3.8 Hz), 123.1, 114.3, 104.2, 100.7, 50.4, 46.7, 42.4, 36.5, 32.7, 28.8. HR-MS *m/z* calcd for $C_{26}H_{30}F_3N_3O$ [(M+H)]$^+$: 458.2414; found 458.2413.

N-(2-Amino-4-((4-(trifluoromethyl)benzyl)amino)phenyl)-2,3,4,5,6-pentafluorobenzamide (c102)
Compound **c102** was obtained from **7** (0.188 g, 0.294 mmol, 1 eq), according to the general procedure B. R_f = 0.44 (*n*-hexane/ethyl acetate 2/8). Off-white powder (0.089 g, 64% yield). **A** ^{1}H NMR (400 MHz, CD_3OD, ppm): δ: 7.61 (d, 2H, Ar-H, *J* = 8.3 Hz); 7.56 (d, 2H, Ar-H, *J* = 8.2 Hz); 6.93 (d, 1H, Ar-H, *J* = 8.3 Hz); 6.11-6.15 (m, 2H, Ar-H); 4.42 (s, 2H, C*H₂*); **B** ^{1}H NMR (400 MHz, CD_3OD, ppm): 7.47 (d, 2H, Ar-H, *J* = 8.0 Hz); 6.62 (d, 1H, Ar-H, *J* = 2.5 Hz); 5.93 (d, 1H, Ar-H, *J* = 2.5 Hz); 5.86 (dd, 1H, Ar-H, *J'* = 2.5 Hz and *J''* = 8.5 Hz); 4.32 (s, 2H, C*H₂*); **A+B** ^{13}C NMR (100 MHz, CD_3OD, ppm): δ: 156.9, 148.7, 145.1, 144.9, 143.4, 142.9, 142.6, 140.7, 138.8, 136.3, 128.7, 128.4, 127.3, 126.8, 125.8, 124.83 (q, $^{3}J_{C,F}$= 3.8 Hz), 123.1, 112.4, 104.0, 102.8, 100.1, 98.6, 46.6, 46.4. HR-MS *m/z* calcd for $C_{21}H_{13}F_8N_3O$ [(M+H)]$^+$: 476.1004; found 476.1003. 168.8;

N-(2-Amino-4-((4-(trifluoromethyl)benzyl)amino)phenyl)-9-oxo-9H-fluorene-3-carboxamide (c103)
Obtained from **8** (0.166 g, 0.254 mmol, 1 eq), applying the general procedure B. R_f = 0.42 (*n*-hexane/ethyl acetate 1/1). Off-white powder (0.059 g, 48% yield). **A** ^{1}H NMR (400 MHz, $(CD_3)_2SO$), ppm): δ: 9.68 (s, 1H, N*H*); 7.79 (dd, 2H, Ar-H, *J'* = 7.7 Hz and *J''* = 13.8 Hz); 7.66-7.74 (m, 4H, Ar-H); 7.57-7.61 (m, 3H, Ar-H); 7.48 (t, 1H, Ar-H, *J* = 7.5 Hz); 7.41 (t, 1H, Ar-H, *J* = 7.5 Hz); 6.99 (d, 1H, Ar-H, *J* = 8.6 Hz); 6.21 (t, 1H, N*H*, *J* = 6.2 Hz); 6.02 (d, 1H, Ar-H, *J* = 2.4 Hz); 5.95 (dd, 1H, Ar-H, *J'* = 2.4 Hz and *J''* = 8.5 Hz); 4.74 (bs, 2H, N*H₂*); 4.35 (d, 2H, C*H₂*, *J* = 8.3 Hz); **B** ^{1}H NMR (400 MHz, $(CD_3)_2SO$), ppm): 9.20 (s, 1H, N*H*); 7.85 (d, 1H, Ar-H, *J* = 7.5 Hz); 7.63 (d, 1H, Ar-H, *J* = 4.4 Hz); 7.33 (d, 1H, Ar-H, *J* = 7.6 Hz); 7.17 (t, 1H, Ar-H, *J* = 7.5 Hz); 6.99 (d, Ar-H, *J* = 8.7 Hz); 6.27 (d, 1H, N*H*, *J* = 8.4 Hz); 5.81 (d, 1H, Ar-H, *J* = 2.4 Hz); 5.51 (dd, 1H, Ar-H, *J'* = 2.4 Hz and *J''* = 8.4 Hz); 4.72 (bs, 1H, N*H*); 4.16 (d, 2H, C*H₂*, *J* = 5.5 Hz); **A+B** ^{13}C NMR (100 MHz, $(CD_3)_2SO$), ppm):

δ: 192.9, 166.7, 166.6, 147.9, 147.87, 146.4, 144.0, 143.4, 140.7, 135.9, 134.8, 134.3, 133.9, 133.7, 133.6, 130.2, 129.8, 129.0, 128.1, 127.7 (q, $^2J_{C,F}$= 31.0 Hz), 127.4, 126.3, 125.6 (q, $^3J_{C,F}$= 3.8 Hz), 125.5, 125.1, 124.9 (q, $^1J_{C,F}$= 271.1 Hz), 124.4, 123.6, 113.2, 113.1, 102.3, 99.6, 46.6, 46.5. HR-MS *m/z* calcd for $C_{28}H_{20}F_3N_3O_2$ [(M+H)]$^+$: 488.1580; found 488.1582.

N-(2-Amino-4-((4-(trifluoromethyl)benzyl)amino)phenyl)-2-cyclopentylacetamide (c104)
Derivative **c104** was obtained from **9** (0.157 g, 0.282 mmol, 1 eq) following the general procedure B. R_f = 0.44 (*n*-hexane/ethyl acetate 1/1). Off-white powder (0.069 g, 63% yield). **A** ^{1}H NMR (400 MHz, CD_3OD, ppm): δ: 7.59 (d, 2H, Ar-H, *J* = 8.3 Hz); 7.54 (d, 2H, Ar-H, *J* = 8.2 Hz); 6.77 (d, 1H, Ar-H, *J* = 8.4 Hz); 6.11 (d, 1H, Ar-H, *J* = 2.4 Hz); 6.08 (dd, 1H, Ar-H, *J'* = 2.5 Hz and *J''* = 8.5 Hz); 4.39 (s, 2H, C*H₂*); 2.27-2.37 (m, 3H, C*H* and C*H₂*); 1.83-1.91 (m, 2H, C*H₂*); 1.58-1.72 (m, 4H, C*H₂*); 1.24-1.32 (m, 2H, C*H₂*); **B** ^{1}H NMR (400 MHz, CD_3OD, ppm): δ: 6.72 (d, 1H, Ar-H, *J* = 8.4 Hz); 6.03 (dd, 1H, Ar-H, *J'* = 2.5 Hz and *J''* = 8.6 Hz); 2.16-2.22 (m, 3H, C*H* and C*H₂*); 1.50-1.56 (m, 4H, C*H₂*); 1.05-1.10 (m, 2H, C*H₂*); **A+B** ^{13}C NMR (100 MHz, CD_3OD, ppm): δ: 173.6; 148.1, 145.2, 142.9, 129.5, 128.6 (q, $^2J_{C,F}$= 33.6 Hz), 127.3, 127.0, 125.8, 124.8 (q, $^3J_{C,F}$= 3.8 Hz), 124.4 (q, $^1J_{C,F}$= 270.0 Hz), 123.1, 114.1, 104.2, 103.4, 100.7, 99.0, 46.7, 41.8, 37.4, 36.2, 32.0, 24.5, 24.4. HR-MS *m/z* calcd for $C_{21}H_{24}F_3N_3O$ [(M+H)]$^+$: 392.1944; found 392.1946.

N-(2-Amino-4-((4-(trifluoromethyl)benzyl)amino)phenyl)-3-methyl-5-phenylisoxazole-4-carboxamide (c105)
Synthesized from **10** (0.155g, 0.242 mmol, 1 eq), according to the general procedure B. R_f = 0.40 (*n*-hexane/ethyl acetate 3/7). Pale oil (0.071 g, 63% yield). ^{1}H NMR (400 MHz, CD_3OD): δ: 2.27 (s, 3H, C*H₃*); 4.33 (s, 2H, C*H₂*); 5.92 (dd, 1H, Ar-H, *J'* = 2.4 Hz and *J''* = 8.5 Hz); 5.97 (d, 1H, Ar-H, *J* = 2.2 Hz); 6.21 (d, 1H, Ar-H, *J* = 8.4 Hz); 7.44-7.51 (m, 5H, Ar-H); 7.58 (d, 4H, Ar-H, *J* = 6.8 Hz); ^{13}C NMR (100 MHz, CD_3OD, ppm): δ: 27.0, 46.6, 100.2, 103.8, 107.0, 113.7, 123.1, 124.7, 124.78, 124.81, 124.85 (q, $^3J_{C,F}$= 3.7 Hz), 125.8, 126.6, 127.3, 127.5, 128.4, 128.5 (q, $^2J_{C,F}$= 32.0 Hz), 128.7, 130.1, 136.8, 143.0, 145.2, 148.0, 164.8, 170.7, 195.4. HR-MS *m/z* calcd for $C_{25}H_{21}F_3N_4O_2$ [(M + H)]$^+$: 467.1689; found 467.1770.

N-(2-Amino-4-((4-(trifluoromethyl)benzyl)amino)phenyl)-2,2-diphenylacetamide (c106)
Obtained from **11** (0.130 g, 0.203 mmol, 1 eq), applying the general procedure B. R_f = 0.45 (*n*-hexane/ethyl acetate 3/7). Greyish powder (0.050 g, 52% yield). ^{1}H NMR (400 MHz, CD_3OD, ppm): δ: 7.56 (d, 2H, Ar-H, *J* = 8.0 Hz); 7.49 (d, 2H, Ar-H, *J* = 8.0 Hz); 7.37 (d, 4H, Ar-H, *J* = 7.2 Hz); 7.31 (t, 4H, Ar-H, *J* = 7.6 Hz); 7.25 (d, 2H, Ar-H, *J* = 8.0 Hz); 6.77 (d, 1H, Ar-H, *J* = 8.4 Hz); 6.04-6.07 (m, 2H, Ar-H); 5.17 (s, 1H, C*H*); 4.32 (s, 2H, C*H₂*); ^{13}C NMR (100 MHz, CD_3OD, ppm): δ: 172.5; 148.2, 145.2, 143.0, 139.8, 128.5, 128.1, 127.3, 127.0, 126.7, 124.8, 113.9, 104.2, 100.6, 57.6, 46.7. HR-MS *m/z* calcd for $C_{28}H_{24}F_3N_3O$ [(M+H)]$^+$: 476.1944; found 476.1928.

(*R*)-N-(2-Amino-4-((4-(trifluoromethyl)benzyl)amino)phenyl)-2-phenylbutanamide (c107R)
Compound **c107R** was synthetized from **12** (0.162 g, 0.274, 1 eq), applying the general procedure B. R_f = 0.39 (*n*-hexane/ethyl acetate 1/1). Pale oil (0.064 g, 55% yield). [α $_D$25= -41 (c= 0.1 methanol)]; **A** ^{1}H NMR (400 MHz, CD_3OD, ppm): δ: 7.63 (d, 2H, Ar-H, *J* = 8.1 Hz); 7.59 (d, 2H, Ar-H, *J* = 8.1 Hz); 7.53 (d, 2H, Ar-H, *J* = 8.1 Hz); 7.43 (d, 2H, Ar-H, *J* = 7.3 Hz); 7.33 (t, 2H, Ar-H, *J* = 7.6 Hz); 7.26 (d, 1H, Ar-H, *J* = 7.2 Hz); 6.71 (d, 1H, Ar-H, *J* = 8.2 Hz); 6.04-6.07 (m, 2H, Ar-H); 4.37 (s, 2H, C*H₂*); 3.56 (t, 1H, C*H*, *J* = 7.1 Hz); 1.76-1.87 (m, 1H, C*H*); 2.09-2.20 (m, 1H, C*H*); 0.97 (t, 3H, C*H₃*, *J* = 7.3 Hz); **B** ^{1}H NMR (400 MHz, CD_3OD, ppm): 7.63 (d, 1H, Ar-H, *J* = 7.7 Hz); 7.16-7.17 (m, 2H, Ar-H); 7.01-7.04 (m, 1H, Ar-H); 6.40 (d, 1H, Ar-H, *J* = 8.4 Hz); 5.94 (dd, 2H, Ar-H, *J'* = 2.2 Hz and *J''* = 8.2 Hz); 4.42 (s, 2H, C*H₂*); 3.44 (t, 1H, C*H*, *J* = 7.4 Hz); 1.95-2.05 (m, 1H, C*H*); 1.61-1.70 (m, 1H, C*H*); 0.82 (t, 3H, C*H₃*, *J* = 7.4 Hz); **A+B** ^{13}C NMR (100 MHz, CD_3OD, ppm): δ: 174.2, 148.1, 145.2, 143.0, 140.6, 128.2, 127.9, 127.8, 127.4, 127.34, 127.3, 127.0, 126.7, 124.85, 124.8 (q, $^3J_{C,F}$= 3.8 Hz), 113.9, 104.1, 100.5, 54.2, 46.7, 26.3, 11.3, 11.2. HR-MS *m/z* calcd for $C_{24}H_{24}F_3N_3O$ [(M+H)]$^+$: 428.1944; found 428.1944.

(*S*)-N-(2-Amino-4-((4-(trifluoromethyl)benzyl)amino)phenyl)-2-phenylbutanamide (c107S)

Final derivative **c107S** was synthetized from **13** (0.143 g, 0.241 mmol, 1 eq), applying the general procedure B. R_f = 0.39 (*n*-hexane/ethyl acetate 1/1). Pale oil (0.060 g, 58% yield); [α $_D$25= +58 (c= 0.1 methanol)]; **A** ^{1}H NMR (400 MHz, CD_3OD, ppm): δ: 7.63 (d, 2H, Ar-H, *J* = 8.1 Hz); 7.58 (d, 2H, Ar-H, *J* = 8.1 Hz); 7.52 (d, 2H, Ar-H, *J* = 8.1 Hz); 7.43 (d, 2H, Ar-H, *J* = 7.3 Hz); 7.32 (t, 2H, Ar-H, *J* = 7.6 Hz); 7.23-7.27 (m, 1H, Ar-H); 6.71 (d, 1H, Ar-H, *J* = 8.2 Hz); 6.04-6.08 (m, 2H, Ar-H); 4.36 (s, 2H, C*H₂*); 3.56 (t, 1H, C*H*, *J* = 7.1 Hz); 2.09-2.20 (m, 1H, C*H*); 1.76-1.86 (m, 1H, C*H*); 0.97 (t, 3H, CH_3, *J* = 7.3 Hz); **B** ^{1}H NMR (400 MHz, CD_3OD, ppm): 7.63 (d, 1H, Ar-H, *J* = 7.7 Hz); 7.16-7.17 (m, 2H, Ar-H); 7.02-7.04 (m, 1H, Ar-H); 6.40 (d, 1H, Ar-H, *J* = 8.4 Hz); 5.94 (dd, 2H, Ar-H, *J'* = 2.2 Hz and *J"* = 8.2 Hz); 4.41 (s, 2H, CH_2); 3.45 (t, 1H, C*H*, *J* = 13.4 Hz); 3.44 (t, 1H, C*H*, *J* = 7.4 Hz); 1.98-2.05 (m, 1H, C*H*); 1.63-1.72 (m, 1H, C*H*); 0.82 (t, 3H, CH_3, *J* = 7.4 Hz); **A+B** ^{13}C NMR (100 MHz, CD_3OD, ppm): δ: 174.2, 149.2, 148.1, 145.2, 143.0, 140.6,130.2, 128.5 (q, $^2J_{C,F}$= 31.2 Hz), 128.2, 127.9, 127.8, 127.4, 127.3, 127.0, 126.7, 126.4, 124.8 (q, $^3J_{C,F}$= 3.7 Hz), 123.1 (q, $^1J_{C,F}$= 269.2 Hz), 113.9, 104.1, 103.1, 100.5, 98.9, 54.2, 46.6, 27.0, 26.3, 11.3, 11.2. HR-MS *m/z* calcd for $C_{24}H_{24}F_3N_3O$ [(M+H)]$^+$: 428.1944; found 428.1947.

N-(2-Amino-4-((4-(trifluoromethyl)benzyl)amino)phenyl)-1-(p-tolyl)cyclopropane-1-carboxamide (c108)

Obtained from **14** (0.187 g, 0.310 mmol, 1 eq), applying the general procedure B. R_f = 0.43 (*n*-hexane/ethyl acetate 1/1). Pale powder (0.064 g, 47% yield). **A** ^{1}H NMR (400 MHz, CD_3OD, ppm): δ: 7.58 (d, 2H, Ar-H, *J* = 8.2 Hz); 7.51 (d, 2H, Ar-H, *J* = 8.3 Hz); 7.42 (d, 2H, Ar-H, *J* = 8.2 Hz); 7.24 (d, 2H, Ar-H, *J* = 7.8 Hz); 6.70 (d, 1H, Ar-H, *J* = 8.4 Hz); 6.06 (d, 1H, Ar-H, *J* = 2.3 Hz); 6.03 (dd, 2H, Ar-H, *J'* = 2.5 Hz and *J"* = 8.4 Hz); 4.36 (s, 2H, CH_2); 2.36 (s, 3H, CH_3); 1.56 (dd, 2H, *J'* = 3.6 Hz and *J"* = 6.6 Hz); 1.12 (dd, 2H, CH_2, *J'* = 3.9 Hz and *J"* = 6.8 Hz); **B** ^{1}H NMR (400 MHz, CD_3OD, ppm): δ: 7.30 (d, 2H, Ar-H, *J* = 8.0 Hz); 7.14 (d, 2H, Ar-H, *J* = 9.4 Hz); 2.32 (s, 2H, CH_2); 0.95 (dd, 2H, CH_2, *J'* = 8.2 Hz and *J"* = 15.6 Hz); **A+B** ^{13}C NMR (100 MHz, CD_3OD, ppm): δ: 174.5, 148.1, 148.0, 145.2, 142.7, 137.7, 136.5, 130.3, 129.4, 128.7, 128.4, 127.3, 126.9, 126.8, 125.8, 124.8 (q, $^3J_{C,F}$= 3.7 Hz), 124.4 (q, $^1J_{C,F}$= 269.2 Hz), 123,1, 114.1, 104.2, 100.6, 46.7, 19.8, 19.7, 14.9. HR-MS *m/z* calcd for $C_{25}H_{24}F_3N_3O$ [(M+H)]$^+$: 440.1944; found 440.1945.

N-(2-Amino-4-((4-(trifluoromethyl)benzyl)amino)phenyl)-5-methyl-2-phenyloxazole-4-carboxamide (c109)

Obtained from **15** (0.161 g, 0.255 mmol, 1 eq), applying the general procedure B. R_f = 0.45 (*n*-hexane/ethyl acetate 3/7). Greyish powder (0.075 g, 63% yield). ^{1}H NMR (400 MHz, $(CD_3)_2SO$), ppm): δ: 9.06 (s, 1H, N*H*); 8.02-8.04 (m, 2H, Ar-H); 7.56-7.58 (m, 5H, Ar-H); 6.92 (d, 1H, Ar-H, *J* = 8.4 Hz); 6.69 (d, 2H, Ar-H, *J* = 8.1 Hz); 6.15 (t, 1H, N*H*, *J* = 6.2 Hz); 6.01 (d, 1H, Ar-H, *J* = 2.3 Hz); 5.91 (dd, 1H, Ar-H, *J'* = 2.3 Hz and *J"* = 8.5 Hz); 4.63 (s, 2H, CH_2); 4.33 (d, 2H, NH_2, *J* = 5.9 Hz); 2.67 (s, 3H, CH_3); ^{13}C NMR (100 MHz, $(CD_3)_2SO$), ppm): δ: 160.2, 158.2, 153.2, 147.7, 146.4, 144.0, 131.3, 131.0, 129.6, 128.1, 127.3, 126.8, 126.5, 125.5 (q, $^3J_{C,F}$= 3.7 Hz), 113.3, 102.5, 100.0, 46.6, 39.4, 12.0. HR-MS *m/z* calcd for $C_{25}H_{21}F_3N_4O_2$ [(M+H)]$^+$: 467.1689; found 467.1686.

(*S*)-N-(2-Amino-4-((4-(trifluoromethyl)benzyl)amino)phenyl)-3,3,3-trifluoro-2-methoxy-2-phenylpropanamide (c111)

Final product **c111** was synthetized from **16** (0.163 g, 0.246 mmol, 1 eq), following the general procedure B. R_f = 0.40 (*n*-hexane/ethyl acetate 1/1). [α $_D$25= -64 (c= 0.1 methanol)]; Whitish oil (0.073 g, 60% yield). ^{1}H NMR (400 MHz, CD_3OD, ppm): δ: 7.66-7.68 (m, 2H, Ar-H); 7.60 (d, 2H, Ar-H, *J* = 8.3 Hz); 7.54 (d, 2H, Ar-H, *J* = 8.2 Hz); 7.45-7.48 (m, 3H, Ar-H); 6.81 (d, 1H, Ar-H, *J* = 8.2 Hz); 6.09-6.12 (m, 2H, Ar-H); 4.40 (s, 2H, CH_2); 3.58 (s, 3H, CH_3); ^{13}C NMR (100 MHz, CD_3OD, ppm): δ: 165.0, 147.6, 143.6, 142.4, 132.4, 129.7, 129.3, 128.7, 127.7 (q, $^3J_{C,F}$= 1.6 Hz), 127.3, 126.8, 125.6 (q, $^3J_{C,F}$= 3.5 Hz), 124.8 (q, $^3J_{C,F}$= 3.7 Hz), 123.7, 122.6, 122.4, 113.4, 112.5, 104.7, 104.2, 101.2,100.5, 55.2, 54.4, 47.7. HR-MS *m/z* calcd for $C_{24}H_{21}F_6N_3O_2$ [(M+H)]$^+$: 498.1611; found 498.1590.

N-(2-Amino-4-((4-(trifluoromethyl)benzyl)amino)phenyl)-4-phenylbutanamide (c112)

Derivative **c112** was synthetized from **17** (0.183 g, 0.309 mmol, 1 eq), according to the general

procedure B. R_f = 0.40 (*n*-hexane/ethyl acetate 3/7). Pale oil (0.087 g, 66% yield). **A** ^{1}H NMR (400 MHz, CD_3OD, ppm): δ: 7.59 (d, 2H, Ar-H, *J* = 8.2 Hz); 7.54 (d, 2H, Ar-H, *J* = 8.2 Hz); 7.16-7.30 (m, 5H, Ar-H); 6.78 (d, 1H, Ar-H, *J* = 8.4 Hz); 6.11 (d, 1H, Ar-H, *J* = 2.2 Hz); 6.08 (dd, 1H, Ar-H, *J'* = 2.4 Hz and *J"* = 8.4 Hz); 4.39 (s, 2H, C*H₂*); 2.70 (t, 2H, CH_2, *J* = 7.8 Hz); 2.39 (t, 2H, CH_2, *J* = 7.6 Hz); 1.97-2.05 (m, 2H, CH_2); **B** ^{1}H NMR (400 MHz, CD_3OD, ppm): δ: 7.08-7.14 (m, 2H, Ar-H); 6.70 (d, 1H, Ar-H, *J* = 8.4 Hz); 6.01 (dd, 1H, Ar-H, *J'* = 2.4 Hz and *J"* = 8.4 Hz); 2.53 (t, 2H, CH_2, *J* = 7.8 Hz); 2.14 (t, 2H, CH_2, *J* = 7.8 Hz); 1.81-1.88 (m, 2H, CH_2); **A+B** ^{13}C NMR (100 MHz, CD_3OD, ppm): δ: 173.6, 148.0, 145.2, 142.9, 141.6, 129.3, 128.7, 128.4, 128.2 (q, $^2J_{C,F}$= 31.5 Hz),127.9, 127.3, 126.9, 126.8, 125.8 (q, $^1J_{C,F}$= 271.1 Hz), 125.5, 124.9, 124.8 (q, $^3J_{C,F}$= 3.8 Hz), 124.7, 123.1, 114.1, 104.2, 103.4, 100.7, 99.0, 46.7, 35.1, 34.9, 31.3, 27.5, 26.5,. HR-MS *m/z* calcd for $C_{24}H_{24}F_3N_3O$ $[(M+H)]^+$: 428.1944; found 428.1942.

N-(2-Amino-4-((4-(trifluoromethyl)benzyl)amino)phenyl)-4-pentylcyclohexane-1-carboxamide (c113)

Obtained from **18** (0.150 g, 0.240 mmol, 1 eq), applying the general procedure B. R_f = 0.45 (*n*-hexane/ethyl acetate 1/1). Pale oil (0.062 g, 56% yield). ^{1}H NMR (400 MHz, $(CD_3)_2SO$), ppm): δ: 8.72 (s, 1H, N*H*); 7.67 (d, 2H, Ar-H, *J* = 8.2 Hz); 7.54 (d, 2H, Ar-H, *J* = 8.0 Hz); 6.70 (d, 1H, Ar-H, *J* = 8.4 Hz); 6.03 (t, 1H, N*H*, *J* = 6.2 Hz); 5.92 (d, 1H, Ar-H, *J* = 2.3 Hz); 5.82 (dd, 1H, Ar-H, *J'* = 2.3 Hz and *J"* = 8.4 Hz); 4.46 (d, 2H, CH_2, *J* = 10.1 Hz); 4.29 (d, 2H, NH_2, *J* = 5.8 Hz); 2.22 (t, 1H, C*H*, *J* = 12.0 Hz); 1.78 (t, 4H, CH_2, *J* = 12.4 Hz); 1.15-1.44 (m, 12H, C*H* and CH_2); 0.85-0.91 (m, 4H, C*H* and CH_2); ^{13}C NMR (100 MHz, $(CD_3)_2SO$), ppm): δ: 174.7, 147.3, 147.27, 146.4, 143.7, 143.6, 128.1, 127.8, 127.5, 127.2, 126.3 (q, $^1J_{C,F}$= 272.5 Hz), 125.5 (q, $^3J_{C,F}$= 3.8 Hz), 114.2, 114.1, 102.1, 99.7, 46.6, 44.8, 37.3, 37.0, 32.6, 32.1, 29.8, 26.4, 22.6, 14.4. HR-MS *m/z* calcd for $C_{26}H_{34}F_3N_3O$ $[(M+H)]^+$: 462.2727; found 462.2725.

N-(2-Amino-4-((4-(trifluoromethyl)benzyl)amino)phenyl)-2-fluoro-5-(trifluoromethyl)benzamide (c114)

Derivative **c114** was obtained from **19** (0.188 g, 0.296 mmol, 1 eq), applying the general procedure B. R_f = 0.42 (*n*-hexane/ethyl acetate 1/1). Off white powder (0.081 g, 58% yield). ^{1}H NMR (400 MHz, CD_3OD, ppm): δ: 8.14 (dd, 1H, Ar-H, *J'* = 2.0 Hz and *J"* = 6.2 Hz); 7.88-7.92 (m, 1H, Ar-H); 7.61 (d, 2H, Ar-H, *J* = 8.3 Hz); 7.56 (d, 2H, Ar-H, *J* = 8.2 Hz); 7.48 (t, 2H, Ar-H, *J* = 9.2 Hz); 6.97 (t, 1H, Ar-H, *J* = 4.1 Hz); 6.14-6.18 (m, 2H, Ar-H); 4.42 (s, 2H, CH_2); ^{13}C NMR (100 MHz, CD_3OD, ppm): δ: 163.0 (d, $^1J_{C,F}$= 2.0 Hz), 162.9 (d, $^4J_{C,F}$= 2.0 Hz), 160.5 (q, $^4J_{C,F}$= 3.4 Hz), 148.44, 148.3 (d, $^3J_{C,F}$= 6.0 Hz), 145.2, 142.6, 142.5 (d, $^2J_{C,F}$= 1.5 Hz), 129.7 (m, $^3J_{C,F}$= 3.8 Hz), 128.7, 128.4, 127.9 (m, $^3J_{C,F}$= 4.0 Hz), 127.3, 127.1 (d, $^3J_{C,F}$= 2.6 Hz), 127.0 (d, $^3J_{C,F}$= 2.6 Hz), 125.8, 124.8 (q, $^3J_{C,F}$= 3.8 Hz), 124.5, 124.4, 123.1, 122.2, 117.3, 117.0, 113.4, 104.4 (d, $^3J_{C,F}$= 3.5 Hz), 100.6 (d, $^3J_{C,F}$= 3.5 Hz), 46.7. HR-MS *m/z* calcd for $C_{22}H_{16}F_7N_3O$ $[(M+H)]^+$: 472.1254; found 472.1260.

N-(2-Amino-4-((4-(trifluoromethyl)benzyl)amino)phenyl)-4-cyclohexylbutanamide (c115)

Obtained from **20** (0.187 g, 0.313 mmol, 1 eq), applying the general procedure B. R_f = 0.39 (*n*-hexane/ethyl acetate 3/7). Greyish powder (0.091 g, 67% yield). **A** ^{1}H NMR (400 MHz, CD_3OD, ppm): δ: 7.60 (d, 2H, Ar-H, *J* = 8.3 Hz); 7.54 (d, 2H, Ar-H, *J* = 8.2 Hz); 6.77 (d, 1H, Ar-H, *J* = 8.4 Hz); 6.11 (d, 1H, Ar-H, *J* = 2.5 Hz); 6.08 (dd, 2H, Ar-H, *J'* = 2.5 Hz and *J"* = 8.4 Hz); 4.39 (s, 2H, CH_2); 2.34 (t, 2H, CH_2, *J* = 7.6 Hz); 1.66-1.79 (m, 8H, CH_2); 1.18-1.31 (m, 5H, CH_2 and CH_3); 0.93 (dd, 2H, CH_2, *J'* = 9.8 Hz and *J"* = 19.9 Hz); **B** ^{1}H NMR (400 MHz, CD_3OD, ppm): δ: 6.73 (d, 1H, Ar-H, *J* = 8.4 Hz); 6.03 (dd, 1H, Ar-H, *J'* = 2.5 Hz and *J"* = 8.4 Hz); 4.59 (s, 2H, CH_2); 2.08 (t, 2H, CH_2, *J* = 7.9 Hz); 1.51-1.58 (m, 4H, CH_2); 1.10-1.15 (m, 5H, CH_2 and CH_3); 0.78-0.84 (m, 2H, CH_2); ^{13}C NMR (100 MHz, CD_3OD): δ: 174.1, 148.0, 145.2, 142.9, 128.4 (q, $^2J_{C,F}$= 31.5 Hz), 127.3, 126.9, 126.8 (q, $^3J_{C,F}$= 3.8 Hz), 123.1, 114.1, 104.2, 100.7, 46.7, 37.4, 36.8, 36.0, 33.1, 32.9, 26.4, 26.1, 23.1. HR-MS *m/z* calcd for $C_{24}H_{30}F_3N_3O$ $[(M+H)]^+$: 434.2414; found 434.2413.

N-(2-Amino-4-((4-(trifluoromethyl)benzyl)amino)phenyl)-4'-methyl-[1,1'-biphenyl]-2-carboxamide (c116)

Synthetized from **21** (0.186 g, 0.291 mmol, 1 eq), applying the general procedure B. R_f = 0.38 (*n*-hexane/ethyl acetate 3/7). Off-white powder (0.076 g, 55% yield). ^{1}H NMR (400 MHz, CD_3OD, ppm): δ: 7.64 (d, 1H, Ar-H, *J* = 7.6 Hz); 7.59 (d, 2H, Ar-H, *J* = 8.2 Hz); 7.52-7.56 (m, 3H, Ar-H); 7.45 (t, 2H, Ar-H, *J* = 7.8 Hz); 7.40 (d, 2H, Ar-H, *J* = 8.0 Hz); 7.26 (d, 2H, Ar-H, *J* = 7.8 Hz); 6.60 (d, 1H, Ar-H, *J* = 8.8 Hz); 6.01-6.03 (m, 2H, Ar-H); 4.37 (s, 2H, C*H₂*); 2.40 (s, 3H, C*H₃*); ^{13}C NMR (100 MHz, CD_3OD, ppm): δ: 171.0, 148.1, 145.2, 143.0, 139.9, 137.6, 137.3, 136.4, 129.8, 129.6, 128.9, 128.4, 127.6, 127.3, 127.0, 126.8, 124.8 (q, $^3J_{C,F}$= 3.8 Hz), 113.3, 103.9, 100.2, 46.6, 19.8. HR-MS *m/z* calcd for $C_{28}H_{24}F_3N_3O$ [(M+H)]$^+$: 476.1944; found 476.1902.

N-(2-Amino-4-((4-(trifluoromethyl)benzyl)amino)phenyl)-2-(3-fluoro-4-methoxyphenyl)acetamide (c117)
Obtained from **22** (0.151 g, 0.247 mmol, 1 eq), applying the general procedure B. R_f = 0.45 (*n*-hexane/ethyl acetate 1/1). Greyish powder (0.067 g, 61% yield). **A** ^{1}H NMR (400 MHz, CD_3OD, ppm): δ: 7.59 (d, 2H, Ar-H, *J* = 8.2 Hz); 7.54 (d, 2H, Ar-H, *J* = 8.3 Hz); 7.03-7.16 (m, 3H, Ar-H); 6.77 (d, 1H, Ar-H, *J* = 8.3 Hz); 6.09 (d, 1H, Ar-H, *J* = 2.4 Hz); 6.07 (dd, 1H, Ar-H, *J'* = 2.4 Hz and *J"* = 8.4 Hz); 4.38 (s, 2H, CH_2); 3.87 (s, 3H, CH_3); 3.60 (s, 2H, CH_2); **B** ^{1}H NMR (400 MHz, CD_3OD, ppm): δ: 6.83 (dd, 1H, Ar-H, *J'* = 2.0 Hz and *J"* = 12.3 Hz); 6.67 (d, 1H, Ar-H, *J* = 8.4 Hz); 6.02 (dd, 2H, Ar-H, *J'* = 2.5 Hz and *J"* = 8.4 Hz); 4.42 (s, 2H, CH_2); 3.83 (s, 3H, CH_3); **A+B** ^{13}C NMR (100 MHz, CD_3OD, ppm): δ: 171.6, 153.4, 150.9, 148.2, 145.2, 143.0, 128.7, 127.3, 127.0, 124.8 (q, $^3J_{C,F}$= 3.8 Hz), 124.7 (d, $^3J_{C,F}$= 3.6 Hz), 116.4, 116.2, 113.8, 113.49, 113.48, 104.1, 100.5, 55.4, 46.7, 41.4. HR-MS *m/z* calcd for $C_{23}H_{21}F_4N_3O_2$ [(M+H)]$^+$: 448.1643; found 448.1642.

N-(2-Amino-4-((4-(trifluoromethyl)benzyl)amino)phenyl)-5-(furan-2-yl)oxazole-4-carboxamide (c118)
Synthetized from **23** (0.131 g, 0.216 mmol, 1 eq), following the general procedure B. R_f = 0.40 (*n*-hexane/ethyl acetate 3/7). Grey powder (0.042 g, 44% yield). ^{1}H NMR (400 MHz, $(CD_3)_2SO$), ppm): δ: 9.19 (s, 1H, N*H*); 8.59 (s, 1H, Ar-H); 7.93 (dd, 1H, Ar-H, *J* = 1.0 Hz); 7.68-7.70 (m, 3H, Ar-H); 6.91 (d, 1H, Ar-H, *J* = 8.4 Hz); 7.57 (d, 2H, CH_2, *J* = 8.1 Hz); 6.71 (dd, 1H, Ar-H, *J'* = 1.8 Hz and *J"* = 3.5 Hz); 6.16 (t, 1H, N*H*, *J* = 6.3 Hz); 6.00 (d, 1H, Ar-H, *J* = 2.4 Hz); 5.90 (dd, 1H, Ar-H, *J'* = 2.4 Hz and *J"* = 8.5 Hz); 4.62 (bs, 2H, NH_2); 4.33 (d, 2H, CH_2, *J* = 5.9 Hz); ^{13}C NMR (100 MHz, $(CD_3)_2SO$), ppm): δ: 159.2, 150.3, 147.8, 146.4, 145.5, 144.0, 143.9, 142.4, 128.3,128.1, 127.8, 127.5, 127.4, 126.3, 125.6 (q, $^3J_{C,F}$= 3.8 Hz), 123.6, 115.0, 113.5, 112.8, 102.5, 100.0, 46.6. HR-MS *m/z* calcd for $C_{22}H_{17}F_3N_4O_3$ [(M+H)]$^+$: 443.1326; found 443.1320.

N-(2-Amino-4-((4-(trifluoromethyl)benzyl)amino)phenyl)cyclopent-3-ene-1-carboxamide (c119)
Final compound **c119** was synthetized from **24** (0.164 g, 0.304 mmol, 1 eq), according to the general procedure B. R_f = 0.42 (*n*-hexane/ethyl acetate 1/1). Greyish powder (0.067 g, 59% yield). **A** ^{1}H NMR (400 MHz, CD_3OD, ppm): δ: 7.60 (d, 2H, Ar-H, *J* = 8.3 Hz); 7.55 (d, 2H, Ar-H, *J* = 8.2 Hz); 6.79-6.81 (m, 1H, Ar-H); 6.12 (d, 1H, Ar-H, *J* = 2.3 Hz); 6.08 (dd, 1H, Ar-H, *J'*= 2.5 Hz and *J"* = 8.4 Hz); 5.69 (s, 2H, C*H*); 4.39 (s, 2H, CH_2); 3.19-3.28 (m, 1H, C*H*); 2.68 (d, 4H, CH_2, *J* = 8.4 Hz); **B** ^{1}H NMR (400 MHz, CD_3OD, ppm): δ: 6.75 (d, 1H, Ar-H, *J* = 7.0 Hz); 6.04 (dd, 1H, Ar-H, *J'* = 2.5 Hz and *J"* = 8.4 Hz); 5.59 (s, 2H, CH_2); 3.04-3.11 (m, 1H, C*H*); 2.56 (d, 4H, CH_2, *J* = 7.0 Hz); **A+B** ^{13}C NMR (100 MHz, CD_3OD, ppm): δ: 176.6, 148.0, 145.2, 142.8, 128.6, 128.4, 127.3, 126.9, 126.8, 124.8 (q, $^3J_{C,F}$= 3.8 Hz), 114.3, 104.3, 100.8, 46.7, 42.9, 36.8. HR-MS *m/z* calcd for $C_{20}H_{20}F_3N_3O$ [(M H)]$^+$: 376.1631; found 376.1631.

N-(2-Amino-4-((4-(trifluoromethyl)benzyl)amino)phenyl)-3-(5-ethyl-1,2,4-oxadiazol-3-yl)benzamide (c120)
Derivative **c120** was obtained from **25** (0.182, 0.282 mmol, 1 eq), according to the general procedure B. R_f = 0.39 (*n*-hexane/ethyl acetate 3/7). Grey powder (0.065 g, 48% yield). ^{1}H NMR (400 MHz, CD_3OD, ppm): δ: 8.54 (bs, 1H, Ar-H); 8.13 (d, 1H, Ar-H, *J* = 7.8 Hz); 8.01 (d, 1H, Ar-H, *J* = 9.4 Hz); 7.55 (t, 1H, Ar-H, *J* = 7.8 Hz); 7.50 (dd, 4H, Ar-H, *J'* = 8.2 Hz and *J"* = 16.8 Hz); 6.82 (d, 2H, Ar-H, *J* = 8.4 Hz); 6.02-6.07 (m, 2H, Ar-H); 4.31 (s, 2H, CH_2); 2.91 (dd, 1H, CH_2, *J'* = 7.6 Hz

and *J"* = 15.2 Hz); 1.34 (t, 3H, C*H*$_3$, *J* = 7.6 Hz); ^{13}C NMR (100 MHz, CD_3OD, ppm): δ: 181.6, 167.6, 166.9, 148.3, 145.3, 143.2, 135.4, 129.9, 129.8, 128.9, 127.33, 127.3, 126.3, 124.8 (q, $^3J_{C,F}$= 3.8 Hz), 113.95, 113.9, 104.3, 100.7, 46.7, 19.6, 9.6. HR-MS *m/z* calcd for $C_{25}H_{22}F_3N_5O_2$ [(M+H)]$^+$: 482.1798; found 482.1752.

N-(2-Amino-4-((4-(trifluoromethyl)benzyl)amino)phenyl)-4-heptylbenzamide (c121)
Obtained from **26** (0.200 g, 0.309 mmol, 1 eq), applying the general procedure B. R_f = 0.44 (*n*-hexane/ethyl acetate 7/3). Pale oil (0.083 g, 56% yield). ^{1}H NMR (400 MHz, $(CD_3)_2SO$), ppm): δ: 9.32 (s, 1H, N*H*); 7.86 (d, 2H, Ar-H, *J* = 8.2 Hz); 7.68 (d, 2H, Ar-H, *J* = 8.1 Hz); 7.56 (d, 2H, Ar-H, *J* = 8.1 Hz); 7.28 (d, 2H, Ar-H, *J* = 8.2 Hz); 6.78 (d, 1H, Ar-H, *J* = 8.4 Hz); 6.12 (t, 1H, NH, *J* = 6.3 Hz); 5.99 (d, 1H, Ar-H, *J* = 2.4 Hz); 5.90 (dd, 1H, Ar-H, *J'* = 2.4 Hz and *J"* = 8.5 Hz); 4.60 (bs, 2H, N*H*$_2$); 4.32 (d, 2H, C*H*$_2$, *J* = 6.0 Hz); 3.17 (d, 2H, C*H*$_2$, *J* = 5.5 Hz); 2.63 (t, 2H, C*H*$_2$, *J* = 7.6 Hz); 1.55-1.62 (m, 2H, C*H*$_2$); 1.24-1.30 (m, 6H, C*H*$_2$); 0.86 (t, 3H, C*H*$_3$, *J* = 7.0 Hz); ^{13}C NMR (100 MHz, $(CD_3)_2SO$), ppm): δ: 165.6, 147.8, 146.4, 146.2, 144.5, 132.8, 128.5, 128.1, 127.8, 127.5, 127.2, 126.3, 125.5 (q, $^3J_{C,F}$= 3.8 Hz), 123.6, 114.0, 102.3, 99.7, 46.6, 35.4, 31.7, 31.2, 29.02, 28.97, 22.5,14.4. HR-MS *m/z* calcd for $C_{28}H_{32}F_3N_3O$ [(M+H)]$^+$: 484.2570; found 484.2569.

N-(2-Amino-4-((4-(trifluoromethyl)benzyl)amino)phenyl)-2-(2-methyl-1H-inden-3-yl)acetamide (c122)
Final compound **c122** was synthetized from **27** (0.146 g, 0.237 mmol, 1 eq), according to the general procedure B. R_f = 0.42 (*n*-hexane/ethyl acetate 1/1). Whitish oil (0.043 g, 40% yield). **A** ^{1}H NMR (400 MHz, CD_3OD, ppm): δ: 7.58 (d, 2H, Ar-H, *J* = 8.1 Hz); 7.42-7.45 (m, 3H, Ar-H); 7.34 (t, 1H, Ar-H, *J* = 8.4 Hz); 7.28 (d, 1H, Ar-H, *J* = 5.8 Hz); 7.17-7.21 (m, 1H, Ar-H); 6.93 (bs, 1H, N*H*); 6.77 (d, 1H, Ar-H, *J* = 8.5 Hz); 6.00 (dd, 1H, Ar-H, *J'* = 2.5 Hz and *J"* = 8.5 Hz); 5.91 (d, 1H, Ar-H, *J* = 2.5 Hz); 4.32 (s, 2H, C*H*$_2$); 2.21 (s, 3H, C*H*$_3$); **B** ^{1}H NMR (400 MHz, CD_3OD, ppm): 7.63 (d, 2H, Ar-H, *J* = 8.0 Hz); 7.49 (d, 3H, Ar-H, *J* = 8.4 Hz); 7.08-7.11 (m, 1H, Ar-H); 6.87 (d, 1H, Ar-H, *J* = 8.5 Hz); 6.03 (d, 1H, Ar-H, *J* = 2.6 Hz); 5.93 (d, 1H, Ar-H, *J* = 2.5 Hz); 4.41 (s, 2H, C*H*$_2$); 2.20 (s, 3H, C*H*$_3$); **A+B** ^{13}C NMR (100 MHz, CD_3OD, ppm): δ: 169.2, 147.5, 145.2, 143.7, 143.4, 142.6, 142.2, 130.9, 129.4 (q, $^2J_{C,F}$= 31.0 Hz), 127.5, 127.4, 127.3, 126.6, 125.55, 125.51 (q, $^3J_{C,F}$= 3.8 Hz), 124.7, 123.6, 123.0, 122.8, 120.3 (q, $^1J_{C,F}$= 271.4 Hz), 118.3, 117.6, 114.1, 104.4, 100.8, 47.7, 47.6, 43.0, 42.6, 33.8, 33.5, 14.3,14.1. HR-MS *m/z* calcd for $C_{26}H_{24}F_3N_3O$ [(M+H)]$^+$: 452.1944; found 452.1921.

N-(2-Amino-3-fluoro-4-((4-(trifluoromethyl)benzyl)amino)phenyl)-2,2-diphenylacetamide (c106F)
Intermediate **28** (0.717 g, 2.18 mmol, 1.0 eq) was dissolved in a mixture of methanol/THF (ratio 1:1, v:v), and zinc dust (0.712 g, 10.9 mmol, 5 eq) and NH_4Cl (0.528 g, 10.9 mmol, 5 eq) were added. The mixture was stirred at room temperature until disappearance of the starting material assessed by TLC. Then, 2,2-diphenylacetyl chloride (0.630 g, 2.62 mmol, 1.2 eq), and DIPEA (0.462 mL, 2.62 mmol, 1.2 eq) were added, and the reaction was stirred at room temperature for 1 hour. The resulting mixture was evaporated *in vacuo*, reconstituted in DCM, and extracted with an aqueous solution of K_2CO_3 (3 x 20 mL). After drying over Na_2SO_4, the organic phase was filtered and concentrated in vacuo. The obtained crude was purified using *n*-hexane/ethyl acetate (1:1, v:v) as mobile phase (R_f = 0.44). Off white powder (0.689 g, 64% yield). **A** ^{1}H NMR (400 MHz, CD_3OD, ppm): δ: 7.47 (d, 2H, Ar-H, *J* = 8.2 Hz); 7.41 (d, 2H, Ar-H, *J* = 8.2 Hz); 7.26 (d, 4H, Ar-H, *J* = 7.2 Hz); 7.21 (t, 4H, Ar-H, *J* = 7.7 Hz); 7.12-7.16 (m, 2H, Ar-H); 7.07-7.11 (m, 4H, Ar-H); 6.48 (dd, 1H, Ar-H, *J'* = 1.8 Hz and *J"* = 8.7 Hz); 5.84 (t, 1H, Ar-H, *J* = 8.8 Hz); 5.05 (s, 1H, C*H*); 4.33 (s, 2H, C*H*$_2$); **B** ^{1}H NMR (400 MHz, CD_3OD, ppm): δ: 7.00-7.02 (m, 4H, Ar-H); 6.05 (dd, 1H, Ar-H, *J'* = 1.6 Hz and *J"* = 7.0 Hz); 5.64 (t, 1H, Ar-H, *J* = 8.8 Hz); 4.83 (s, 1H, C*H*); **A+B** ^{13}C NMR (100 MHz, CD_3OD, ppm): δ: 172.6, 145.0, 142.3, 140.0, 139.7, 135.3, 135.2, 131.0, 130.9, 128.7, 128.5, 128.1, 127.9, 127.3 (q, $^1J_{C,F}$= 272.5 Hz), 127.1, 126.7, 126.5, 125.8, 124.9 (q, $^3J_{C,F}$= 3.3 Hz), 121.3, 121.26, 114.85, 114.8, 101.4 (d, $^3J_{C,F}$= 3.0 Hz), 57.5, 46.2. HR-MS *m/z* calcd for $C_{28}H_{23}F_4N_3O$ [(M+H)]$^+$: 494.1850; found 494.1848.

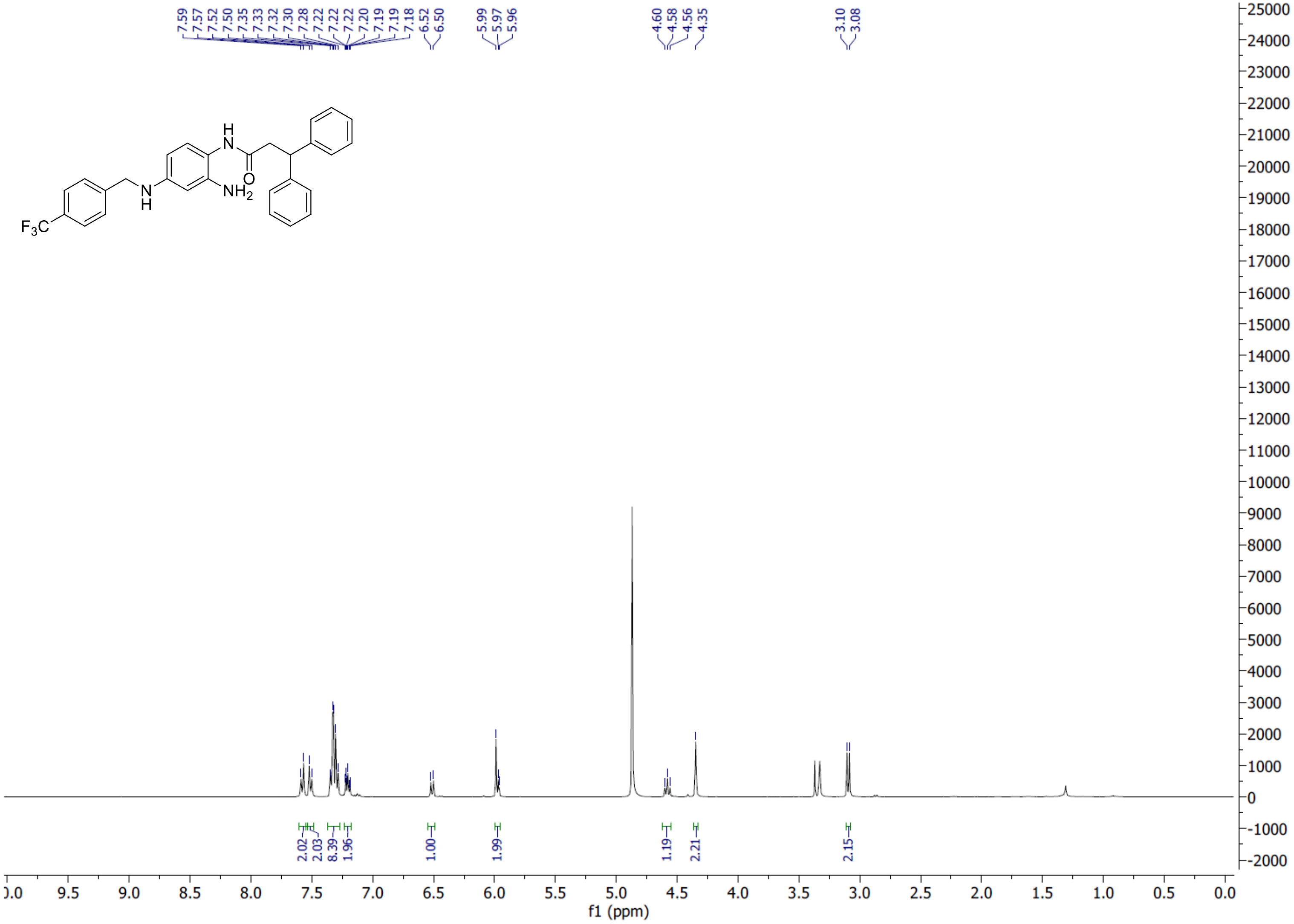

Figure S1: Compound **c97** ^{1}H-NMR spectrum

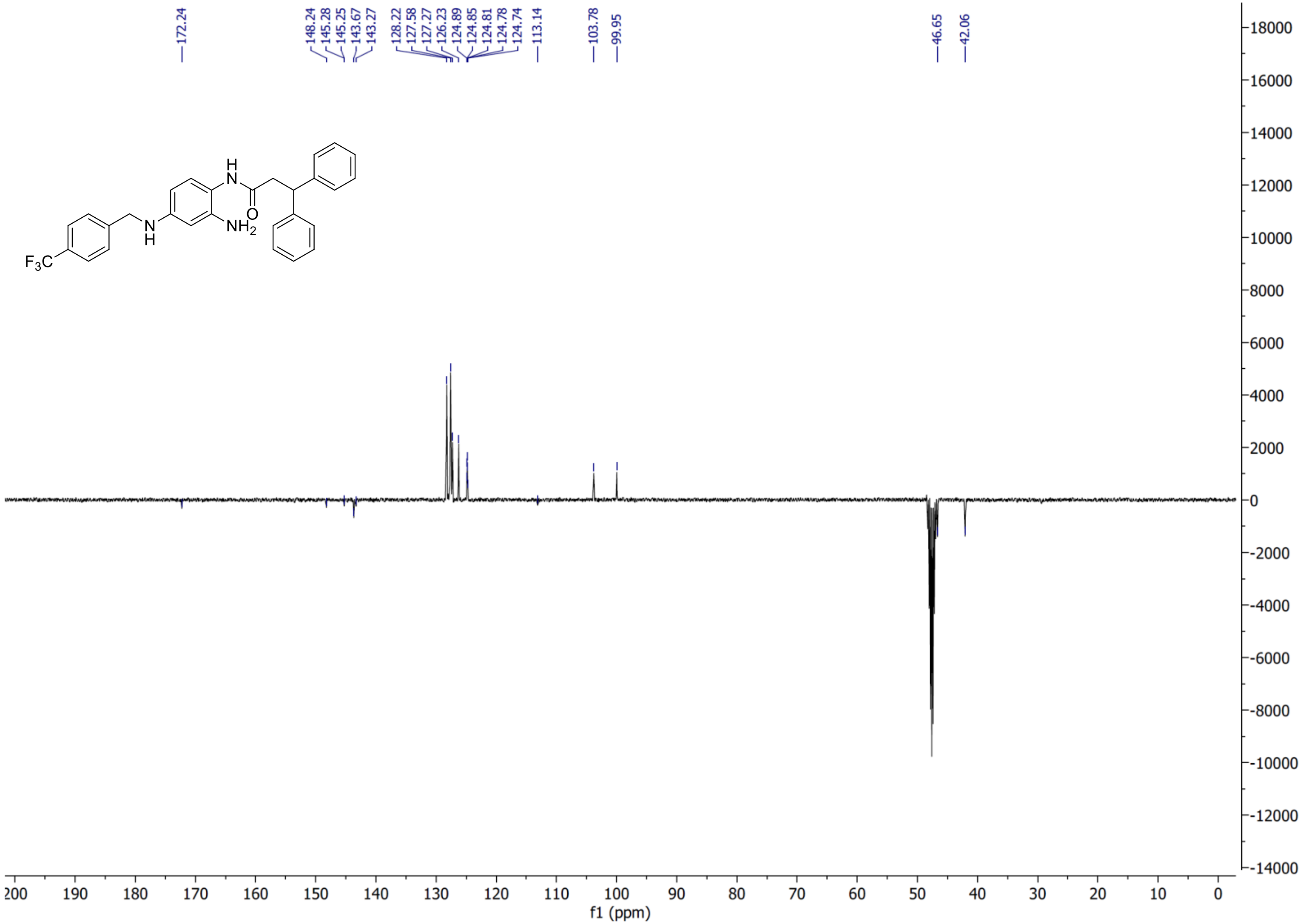

Figure S2: Compound **c97 q**DEPT NMR spectrum

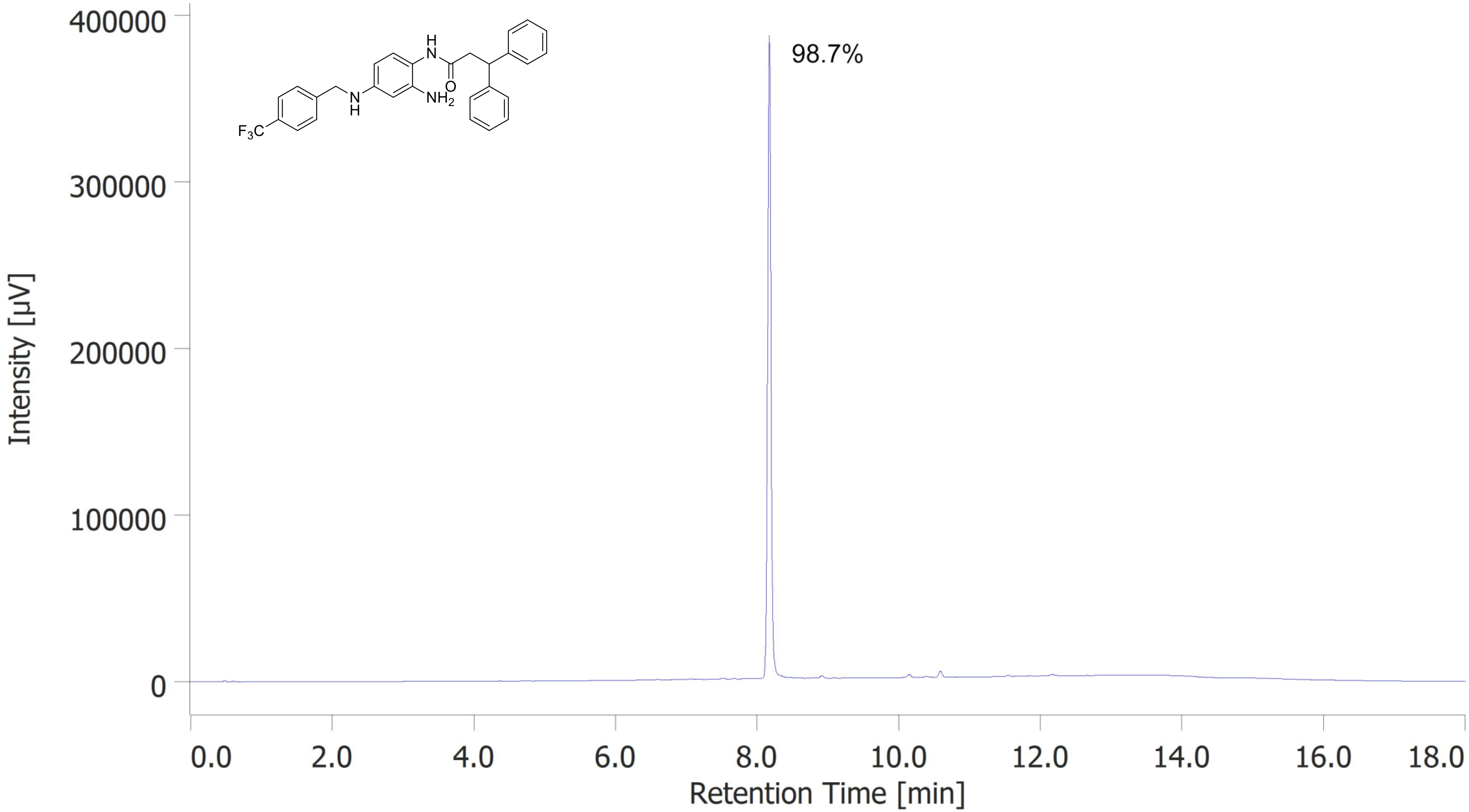

Figure S3: Compound **c97** HPLC trace

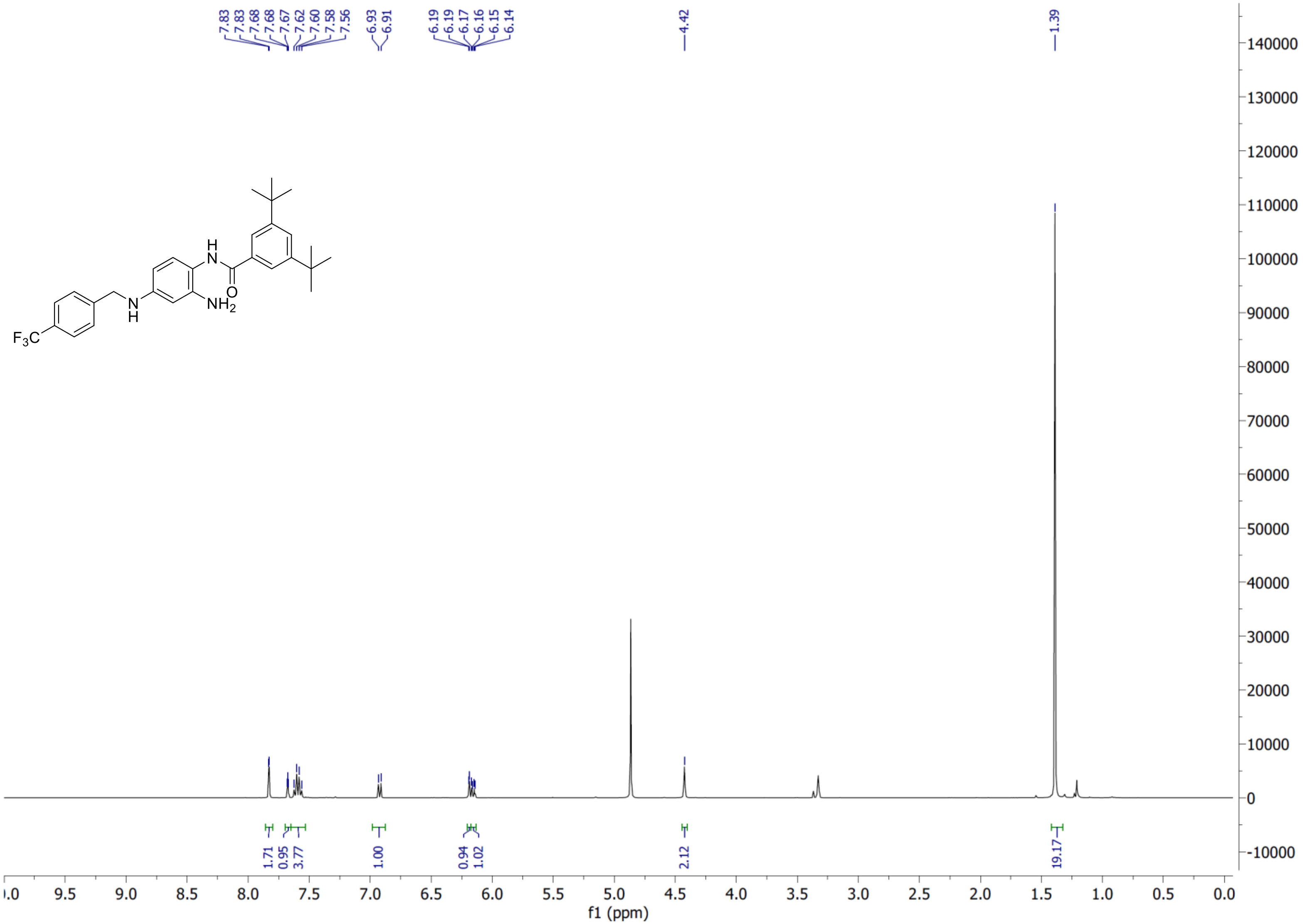

Figure S4: Compound **c98** ^{1}H-NMR spectrum

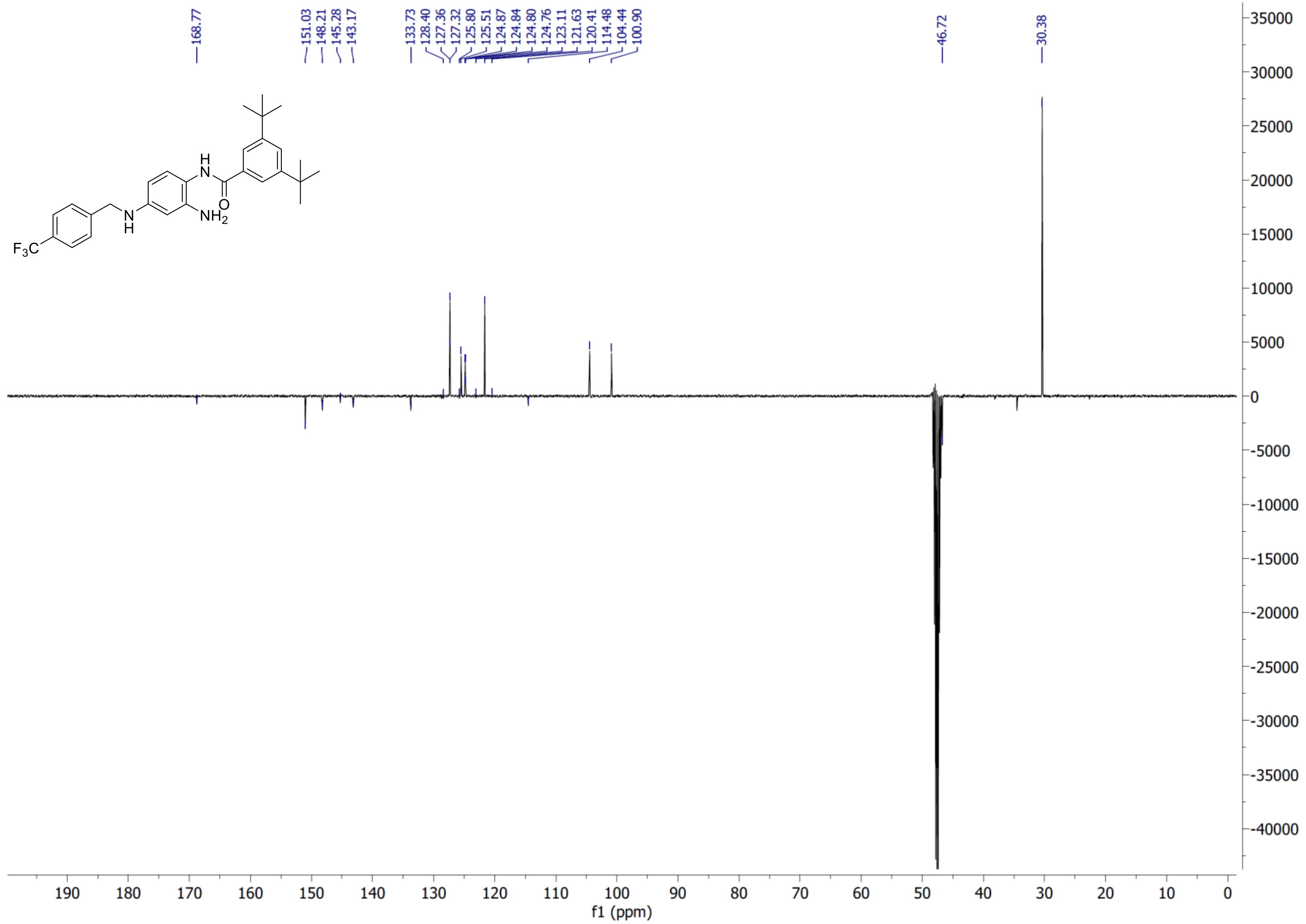

Figure S5: Compound **c98 q**DEPT NMR spectrum

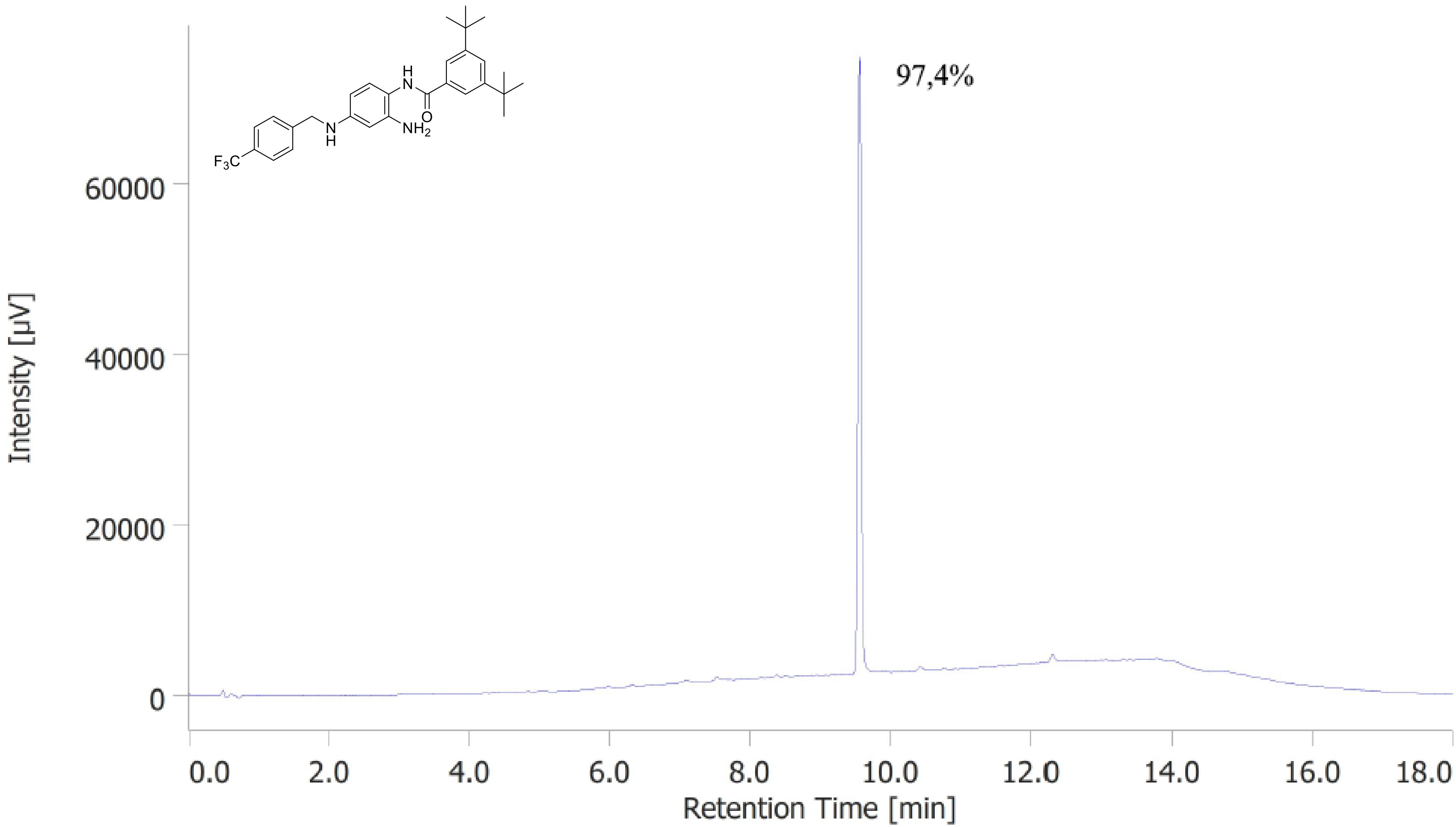

Figure S6: Compound **c98** HPLC trace

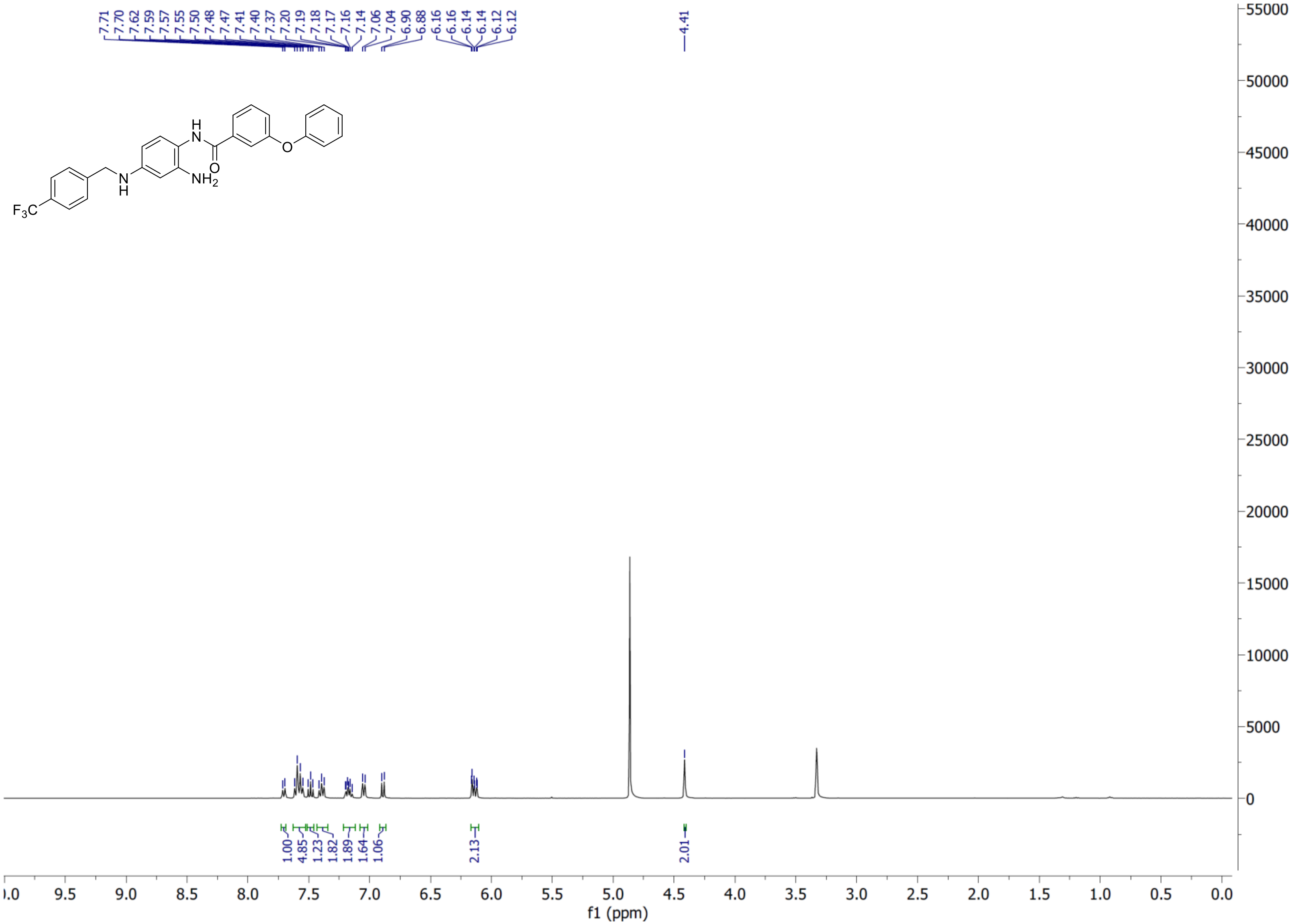

Figure S7: Compound **c100** ^{1}H-NMR spectrum

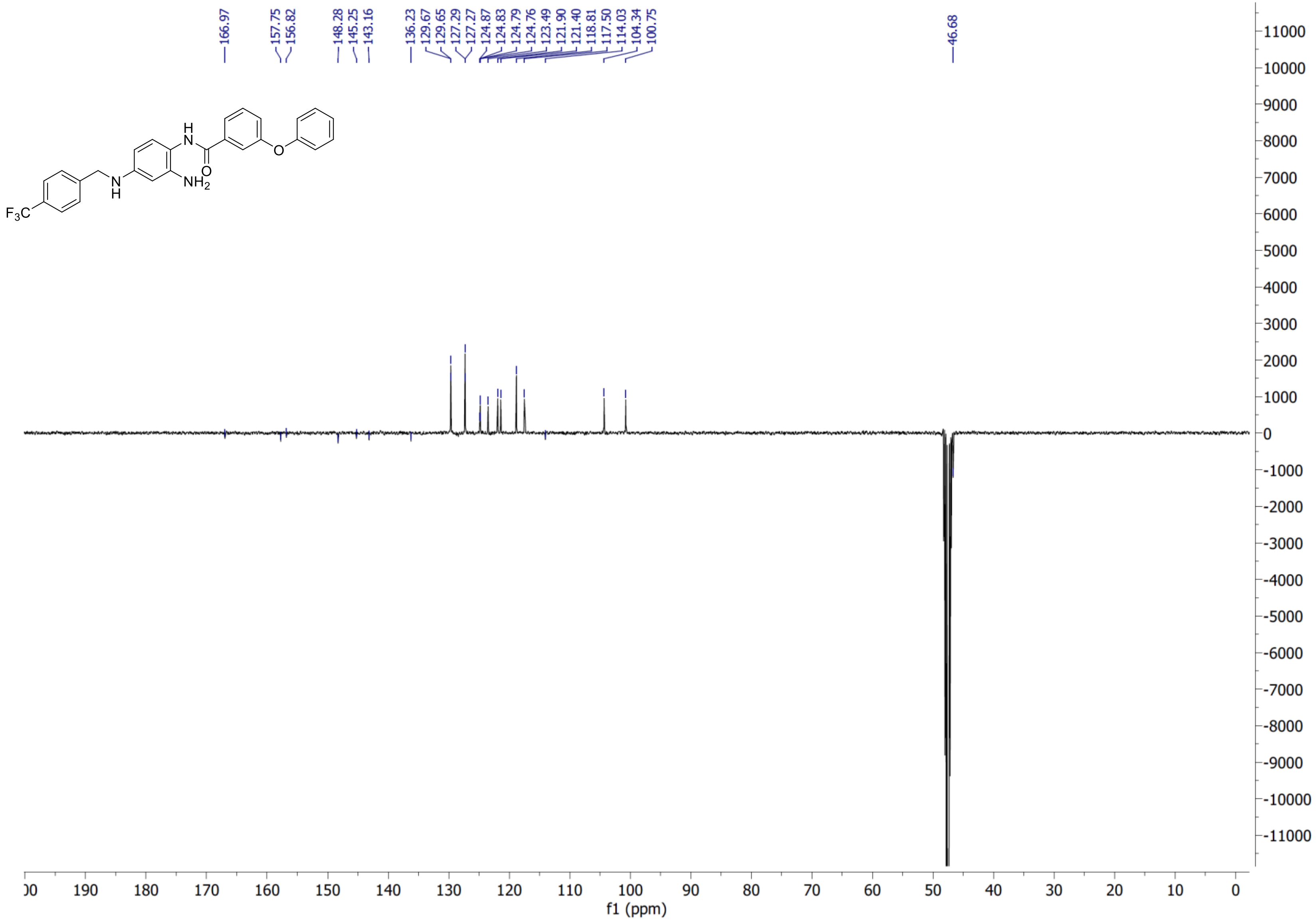

Figure S8: Compound **c100 q**DEPT NMR spectrum

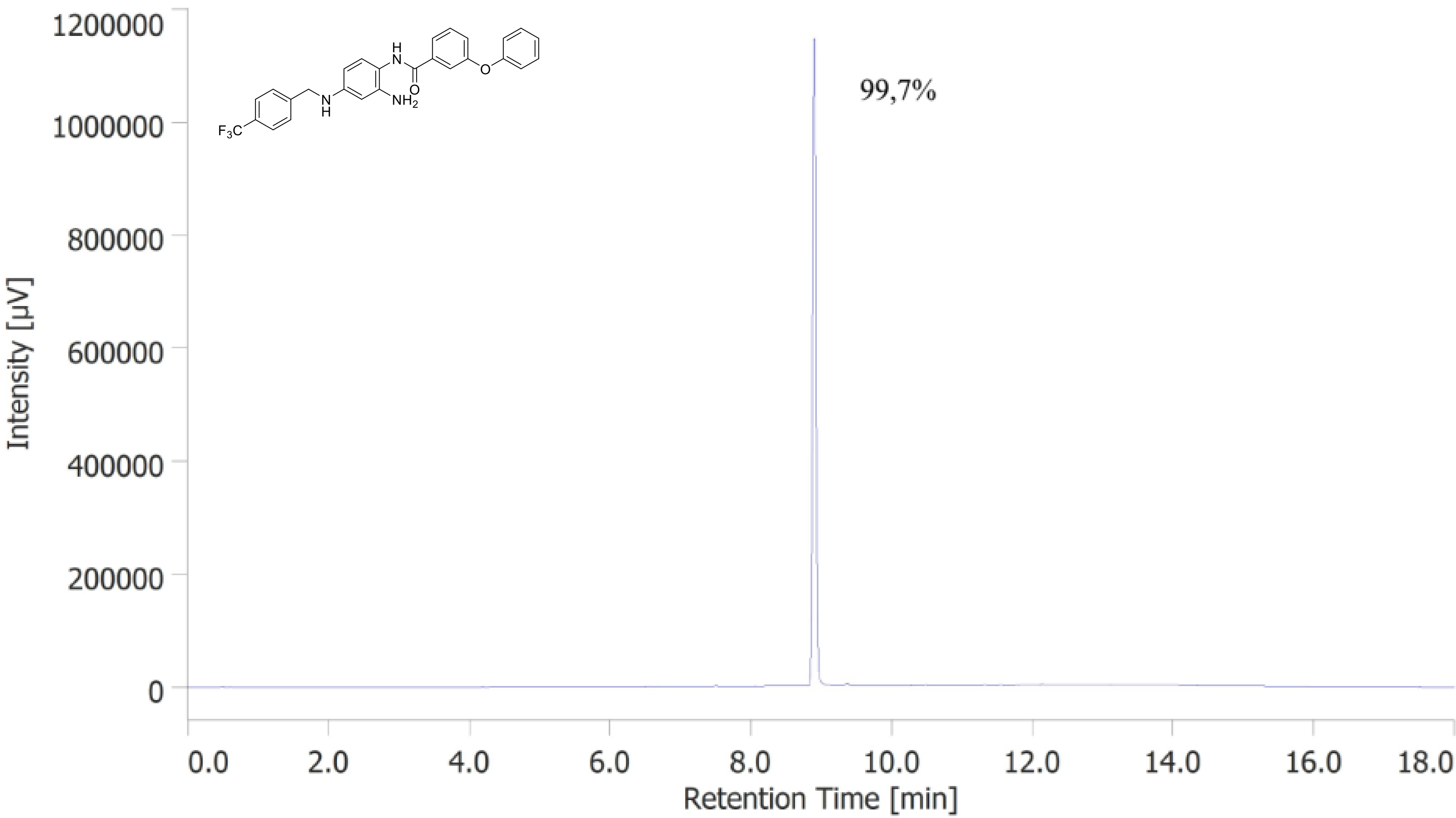

Figure S9: Compound **c100** HPLC trace

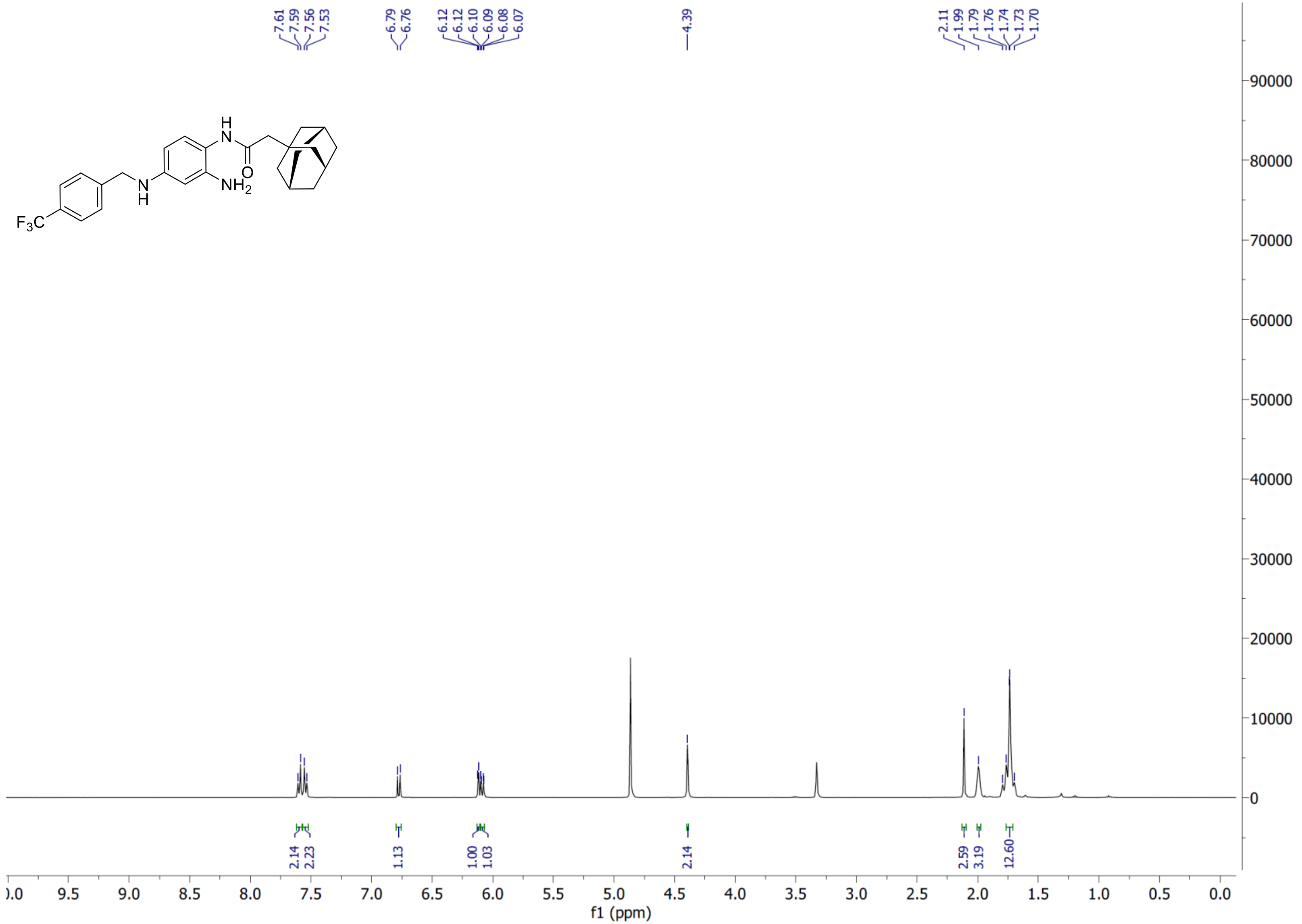

Figure S10: Compound **c101** ¹H-NMR spectrum

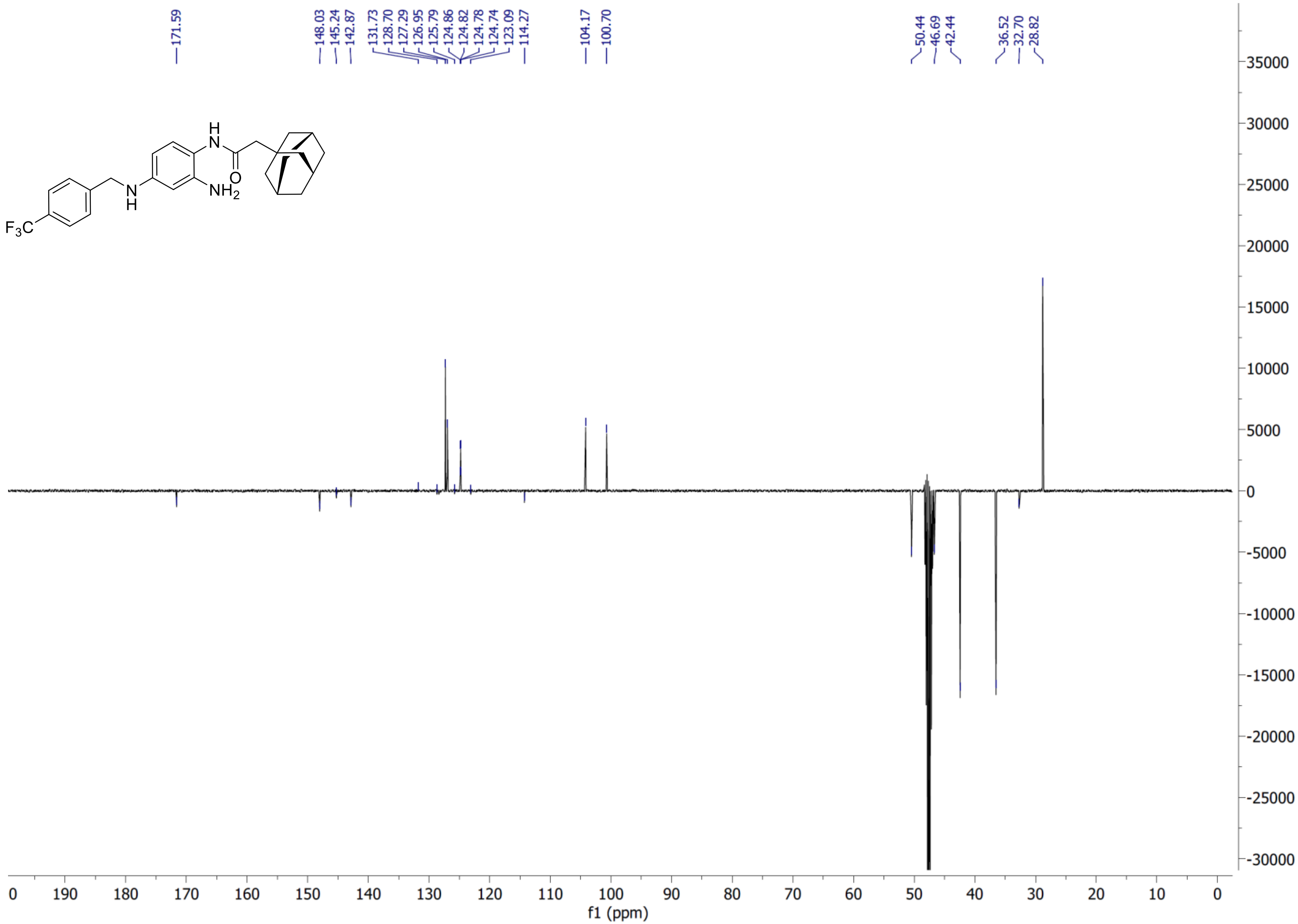

Figure S11: Compound **c101 q**DEPT NMR spectrum

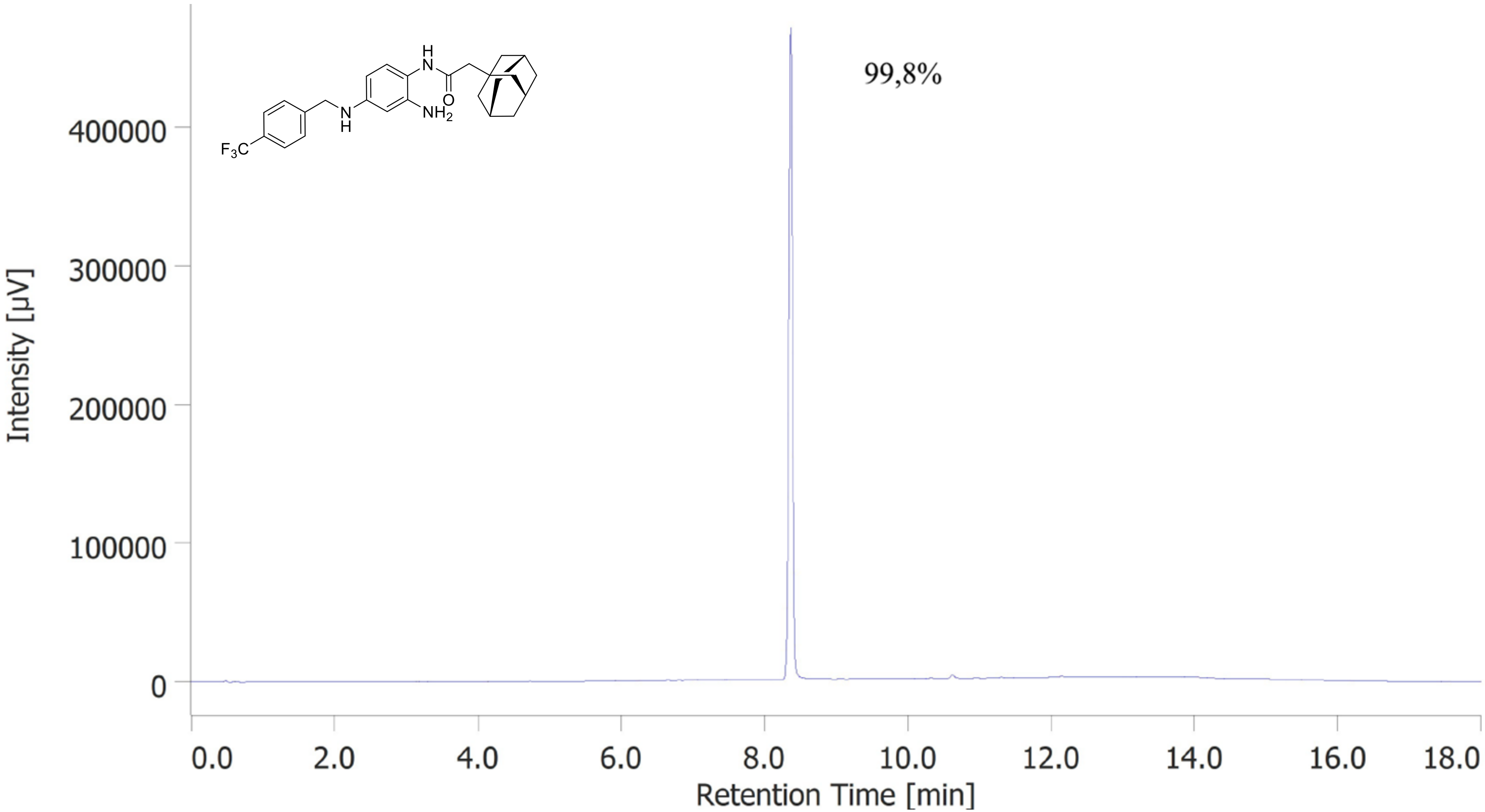

Figure S12: Compound **c101** HPLC trace

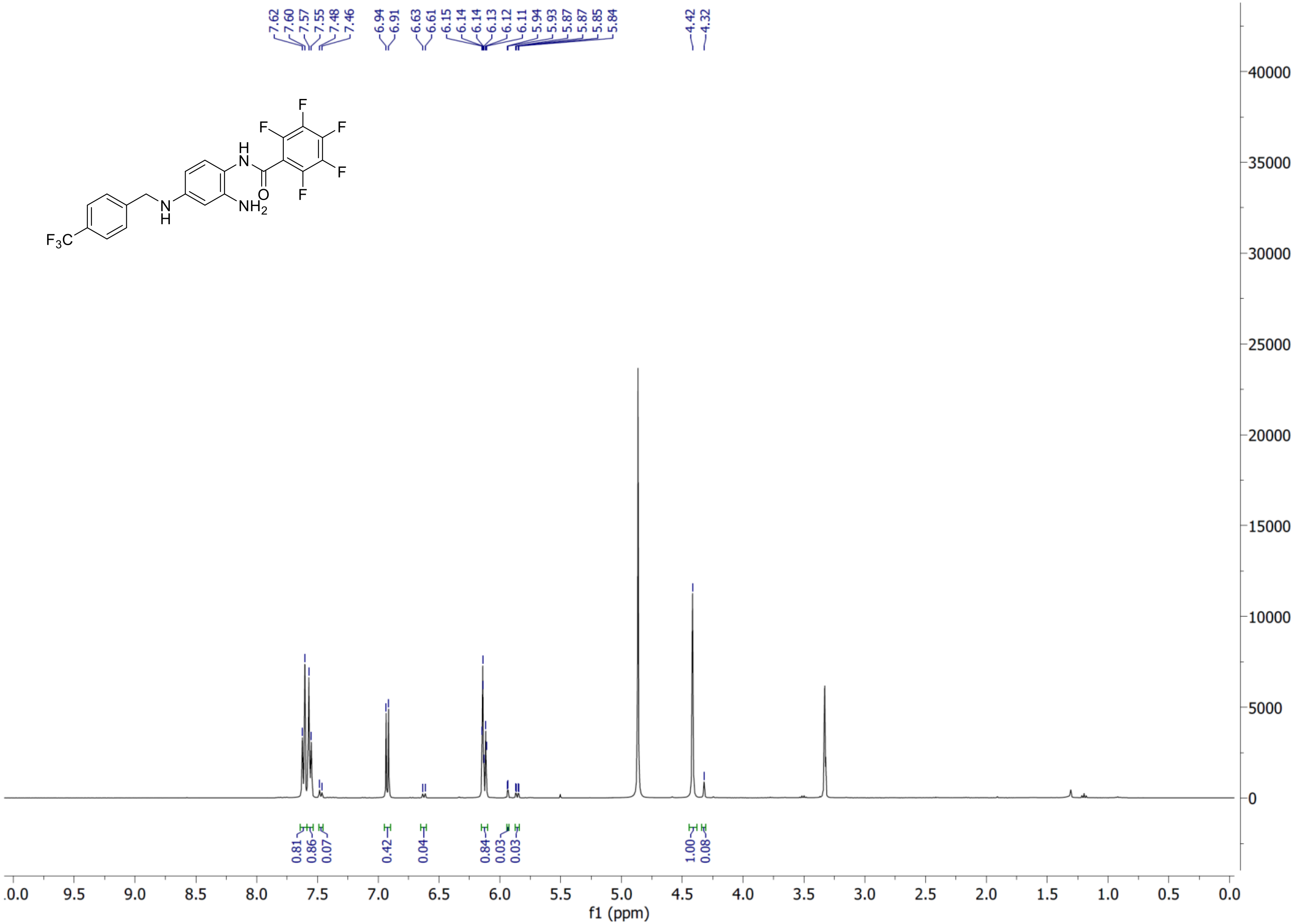

Figure S13: Compound **c102** ^{1}H-NMR spectrum

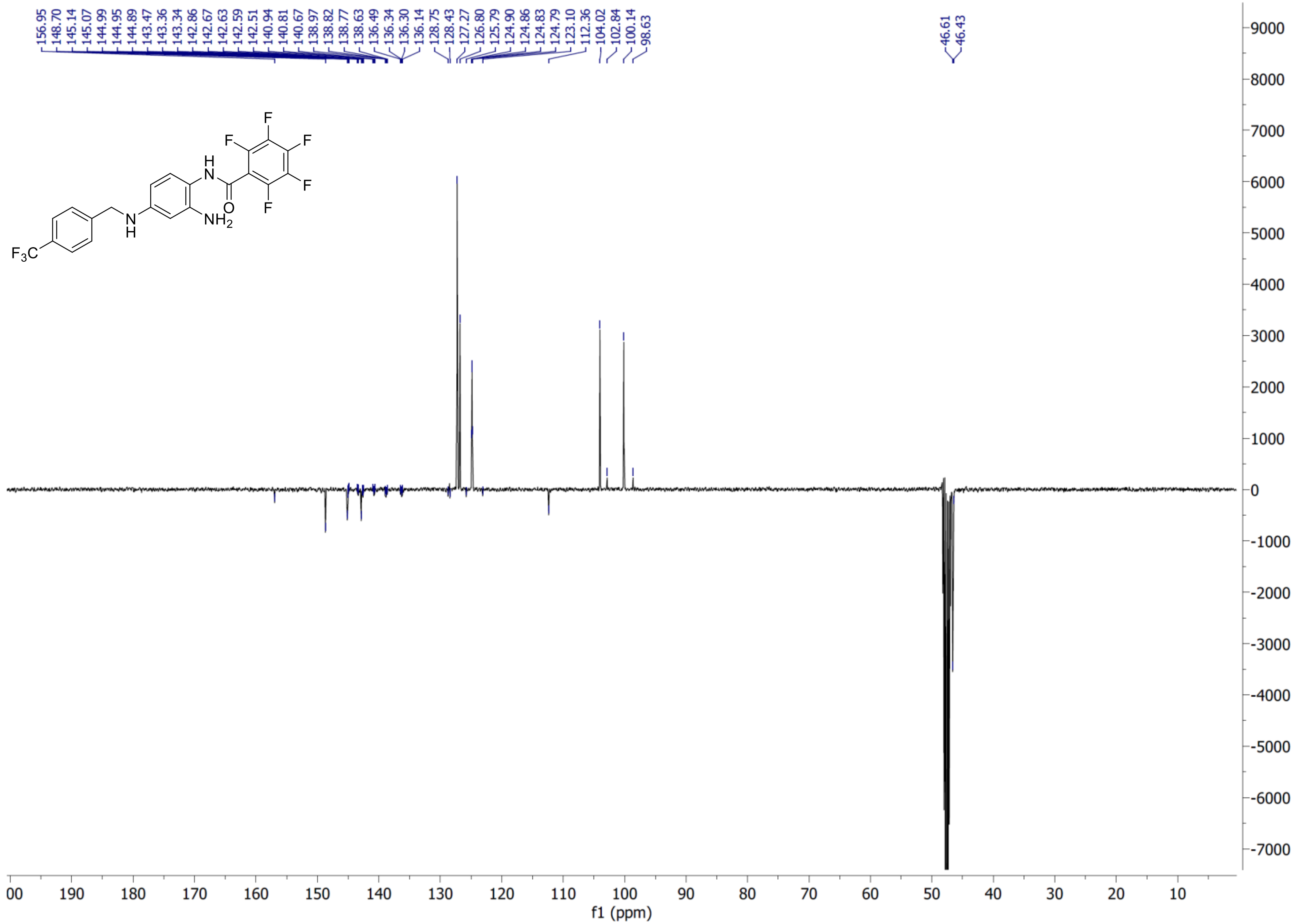

Figure S14: Compound **c102 q**DEPT NMR spectrum

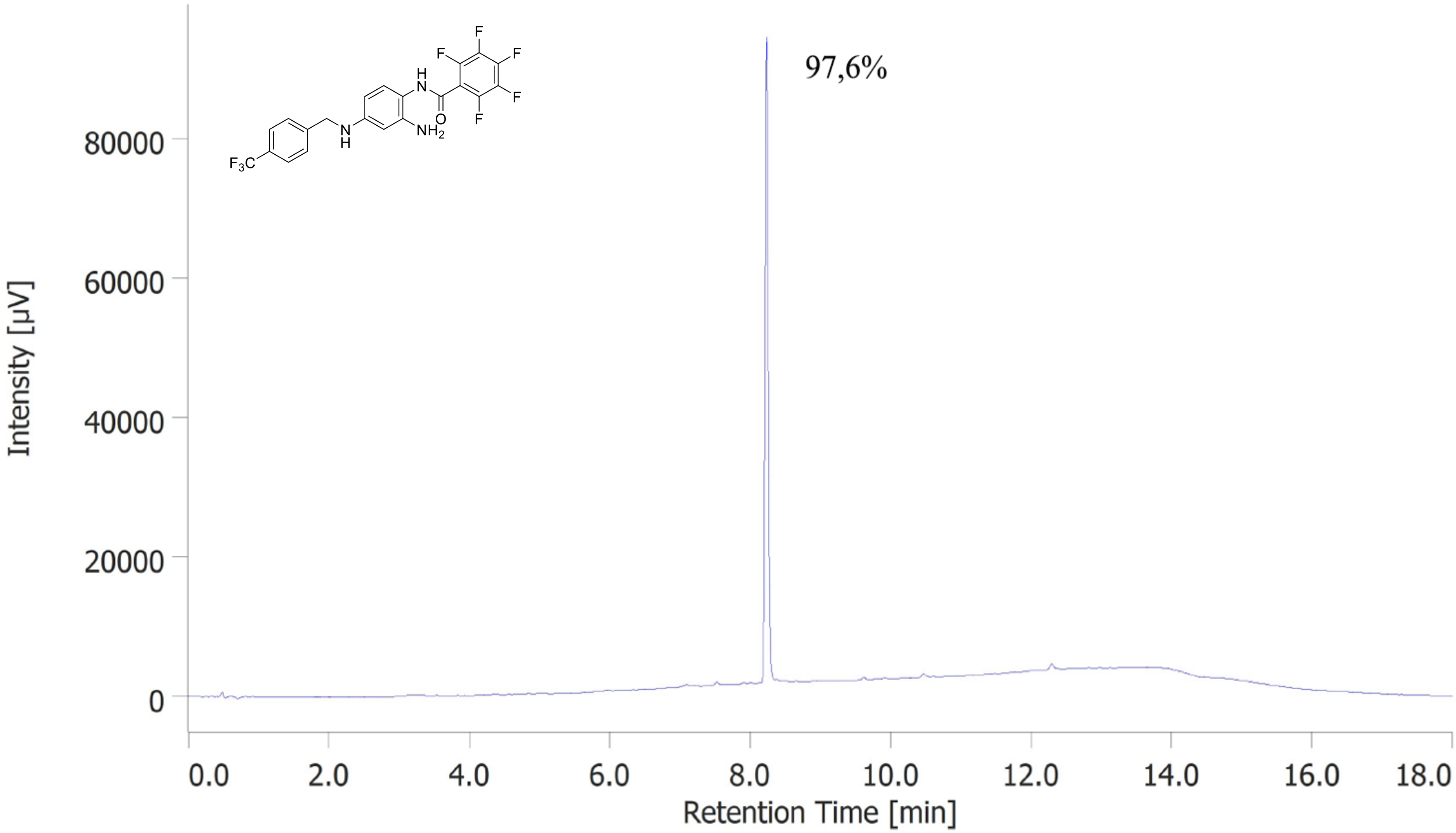

Figure S15: Compound **c102** HPLC trace

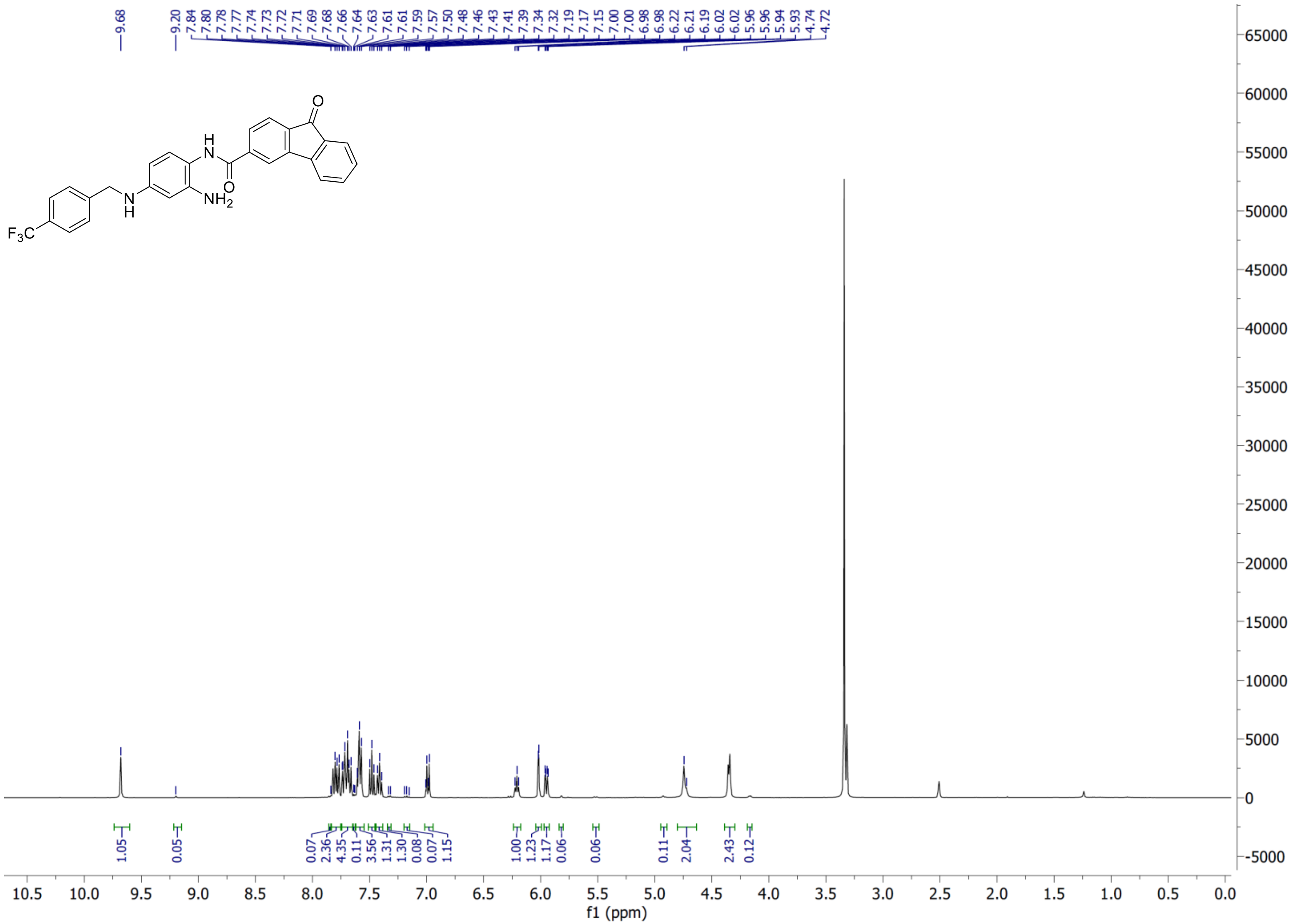

Figure S16: Compound **c103** ^{1}H-NMR spectrum

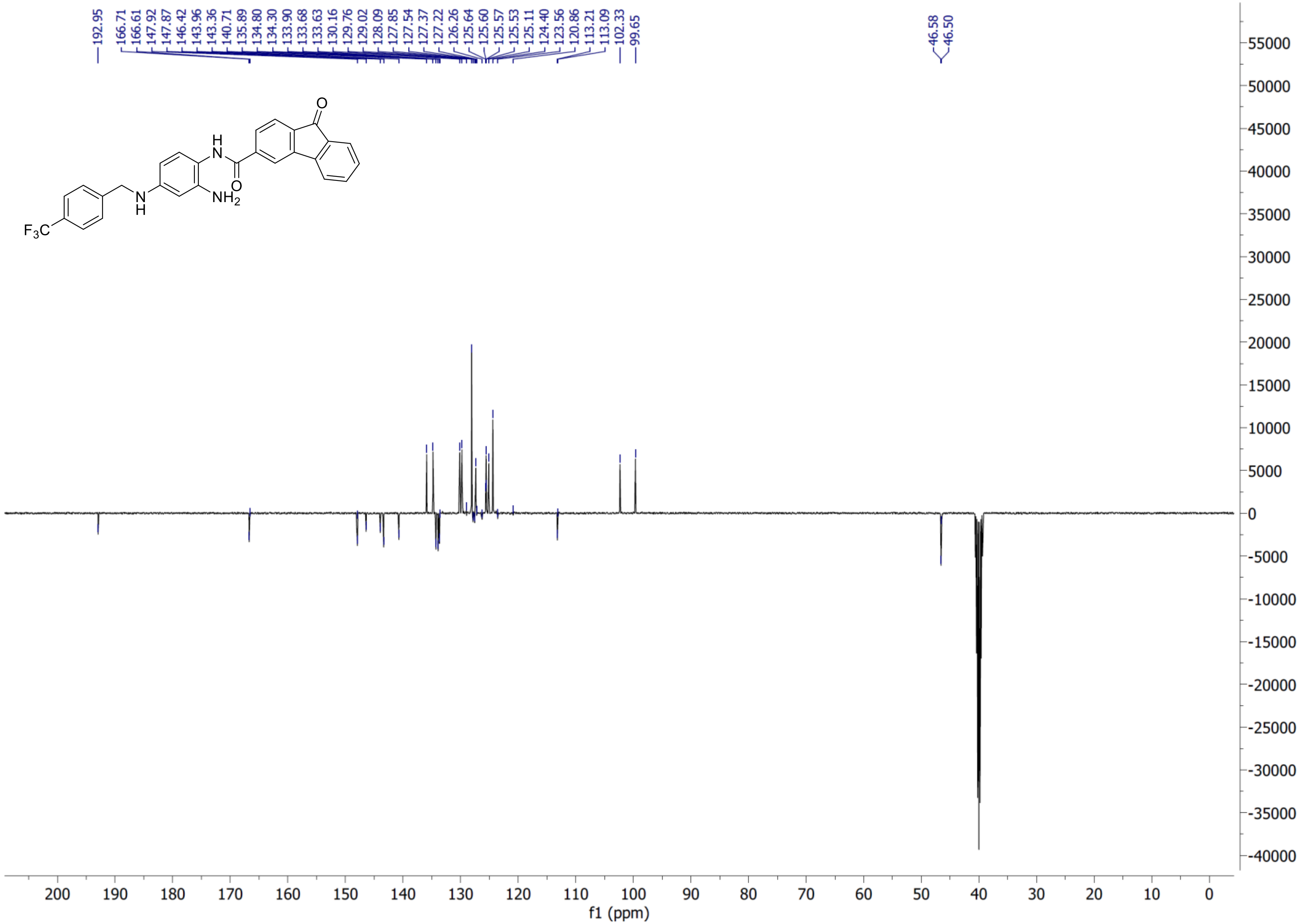

Figure S17: Compound **c103 q**DEPT NMR spectrum

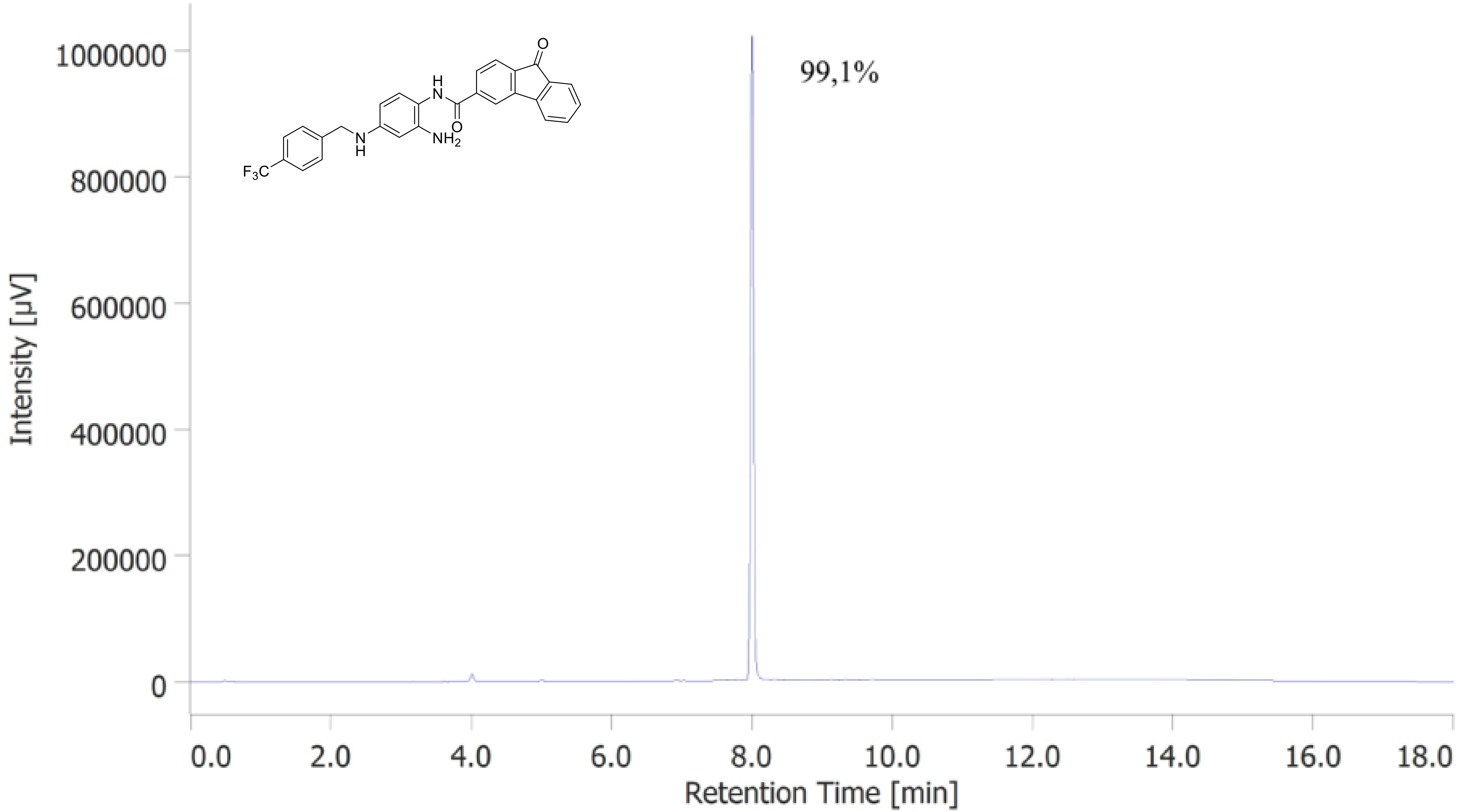

Figure S18: Compound **c103** HPLC trace

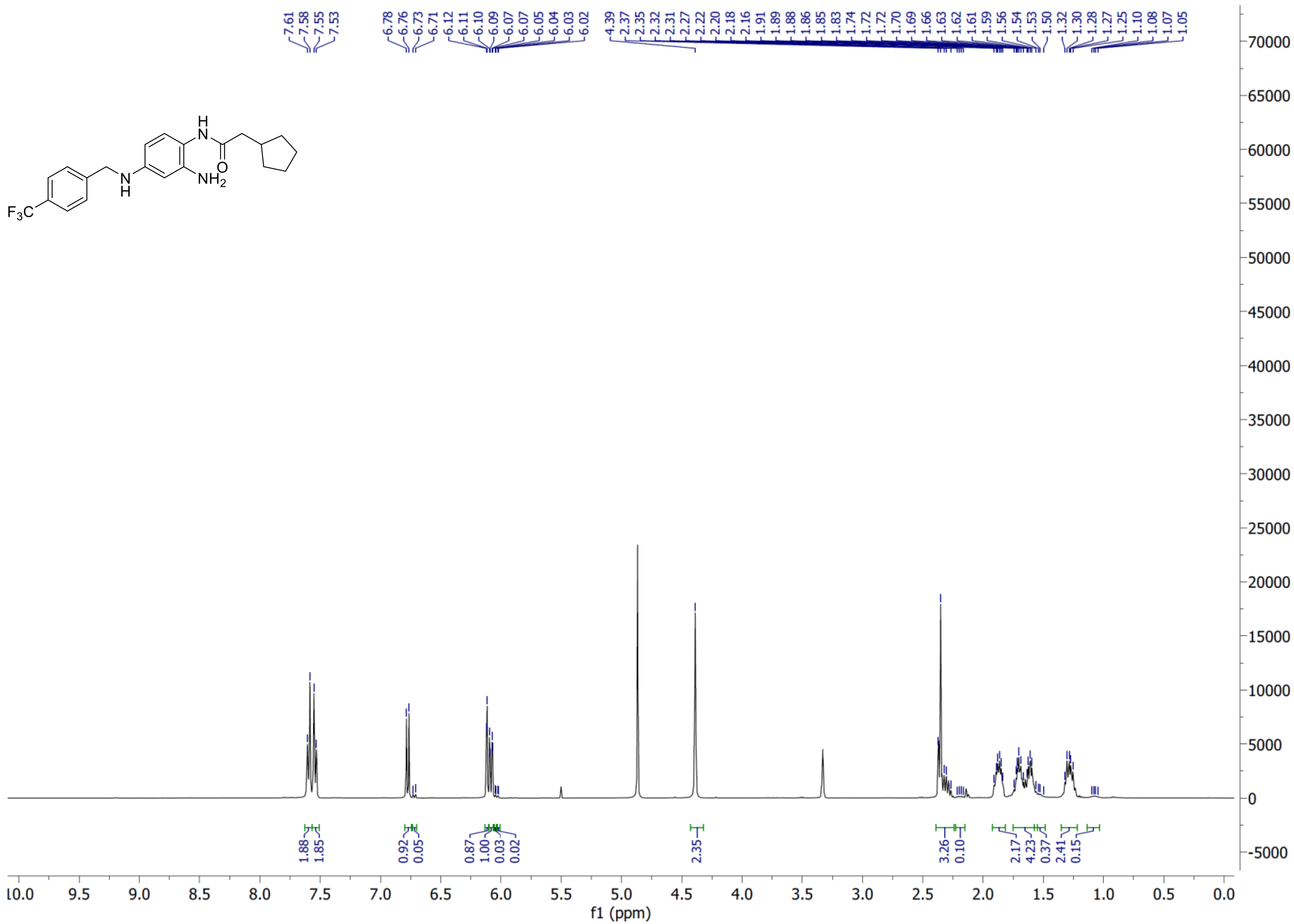

Figure S19: Compound **c104** ^{1}H-NMR spectrum

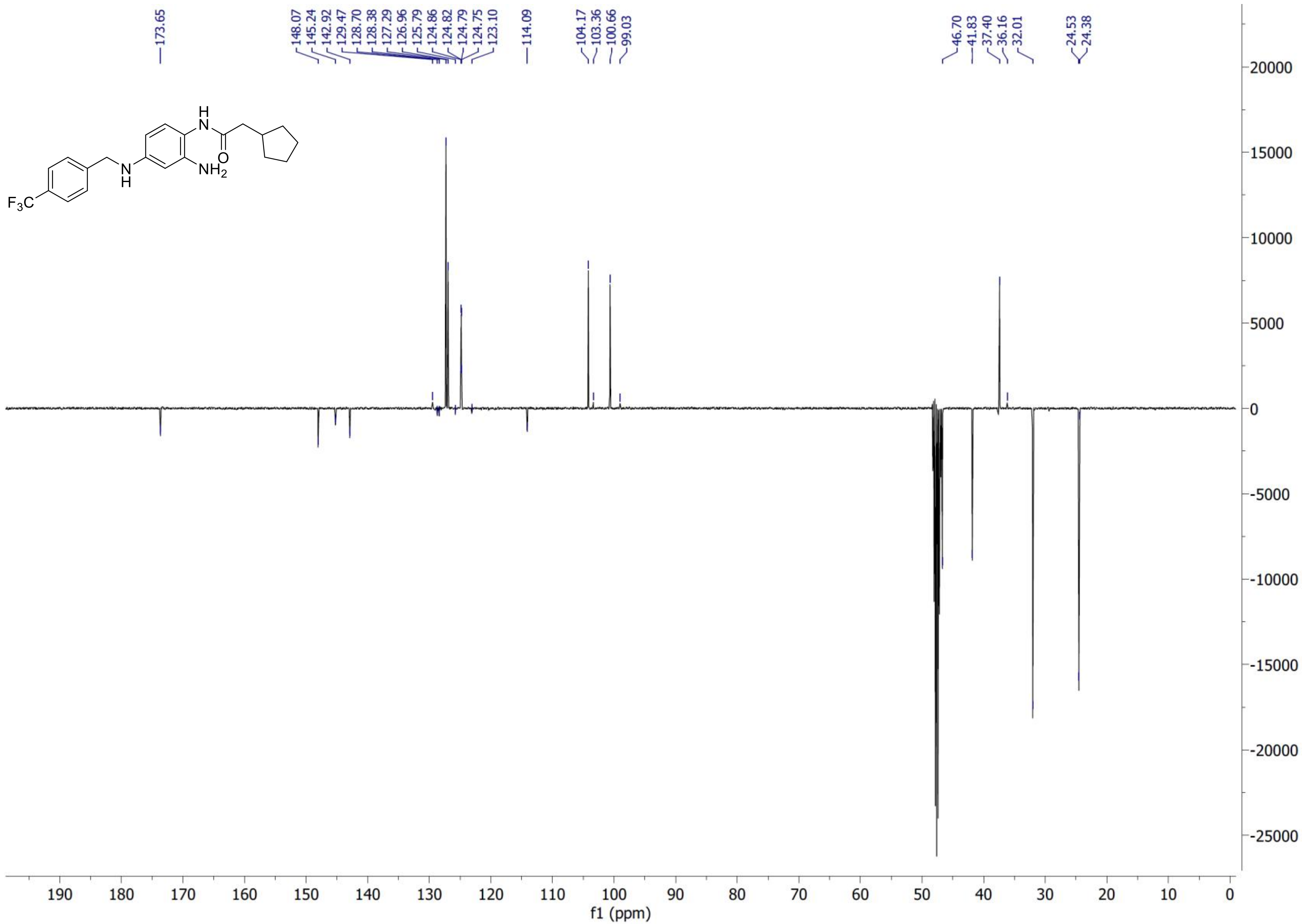

Figure S20: Compound **c104 q**DEPT NMR spectrum

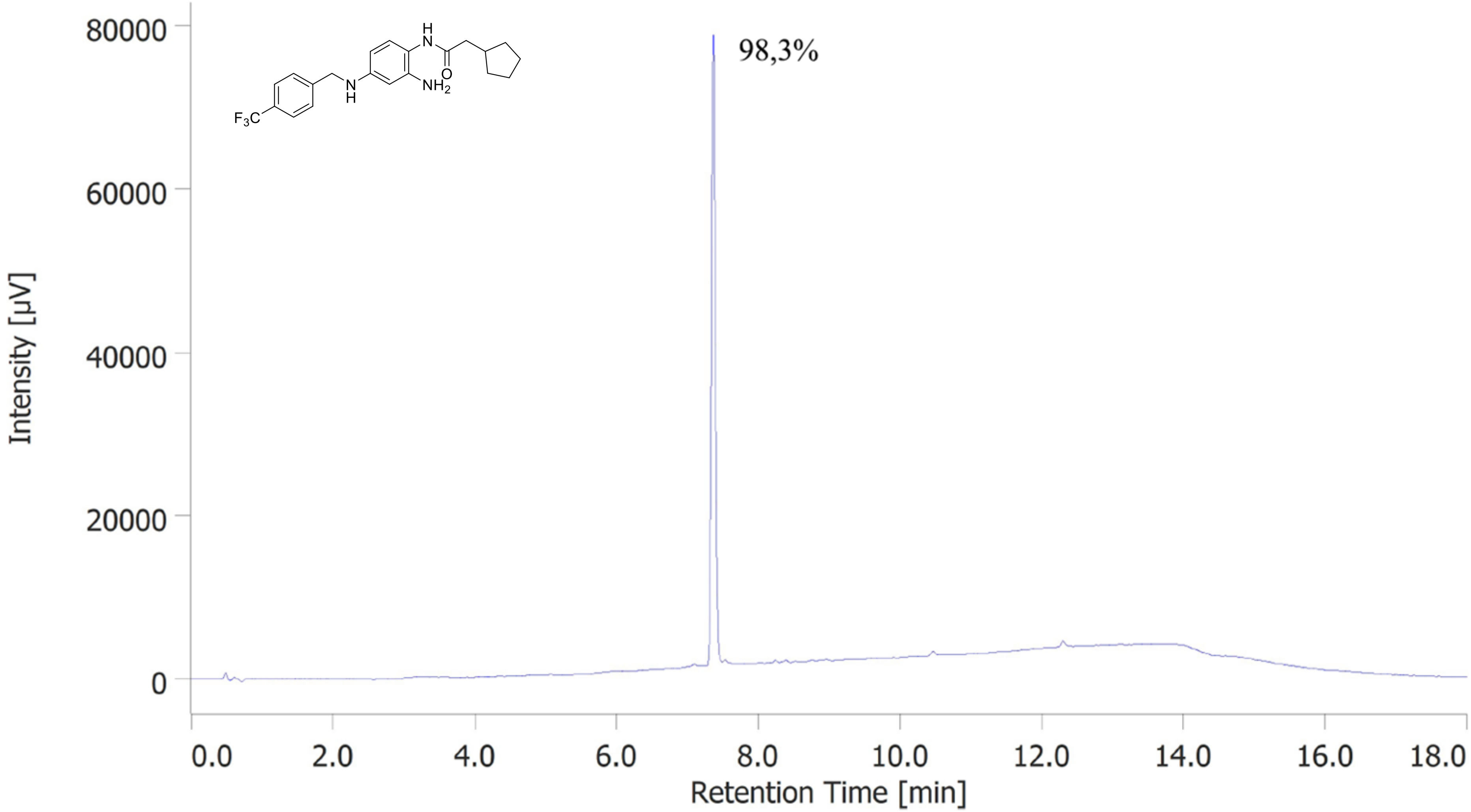

Figure S21: Compound **c104** HPLC trace

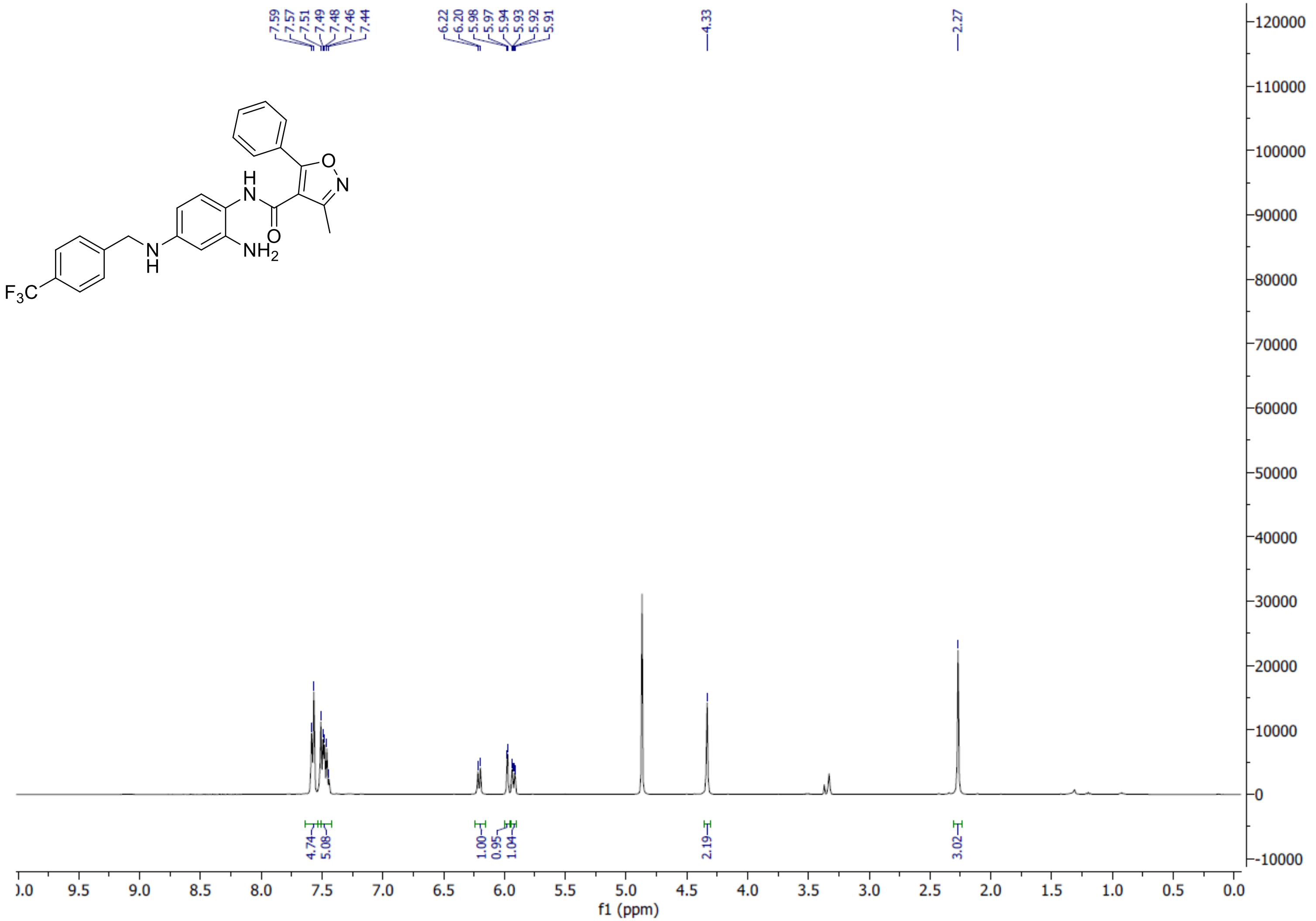

Figure S22: Compound **c105** ^{1}H-NMR spectrum

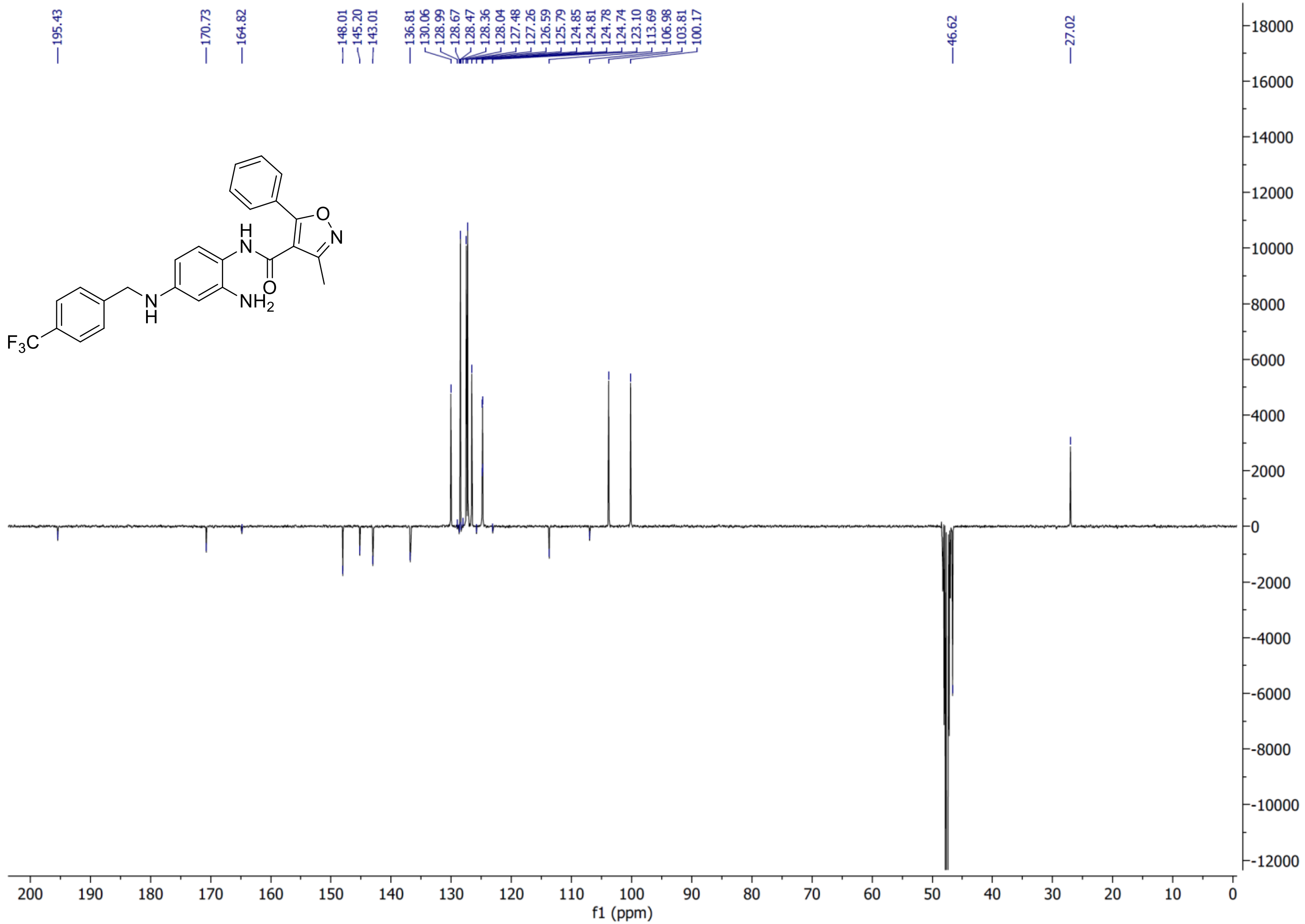

Figure S23: Compound **c105 q**DEPT NMR spectrum

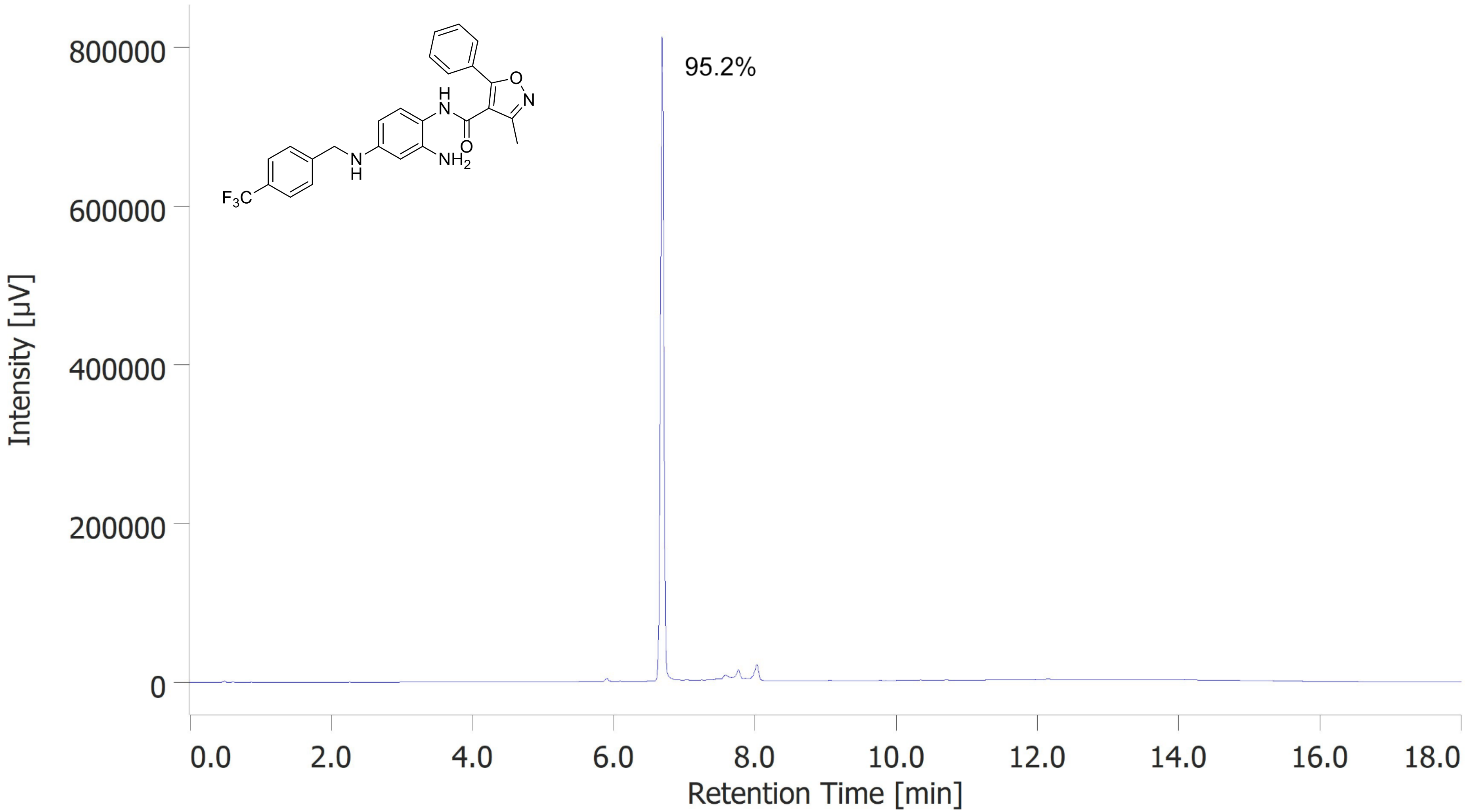

Figure S24: Compound **c105** HPLC trace

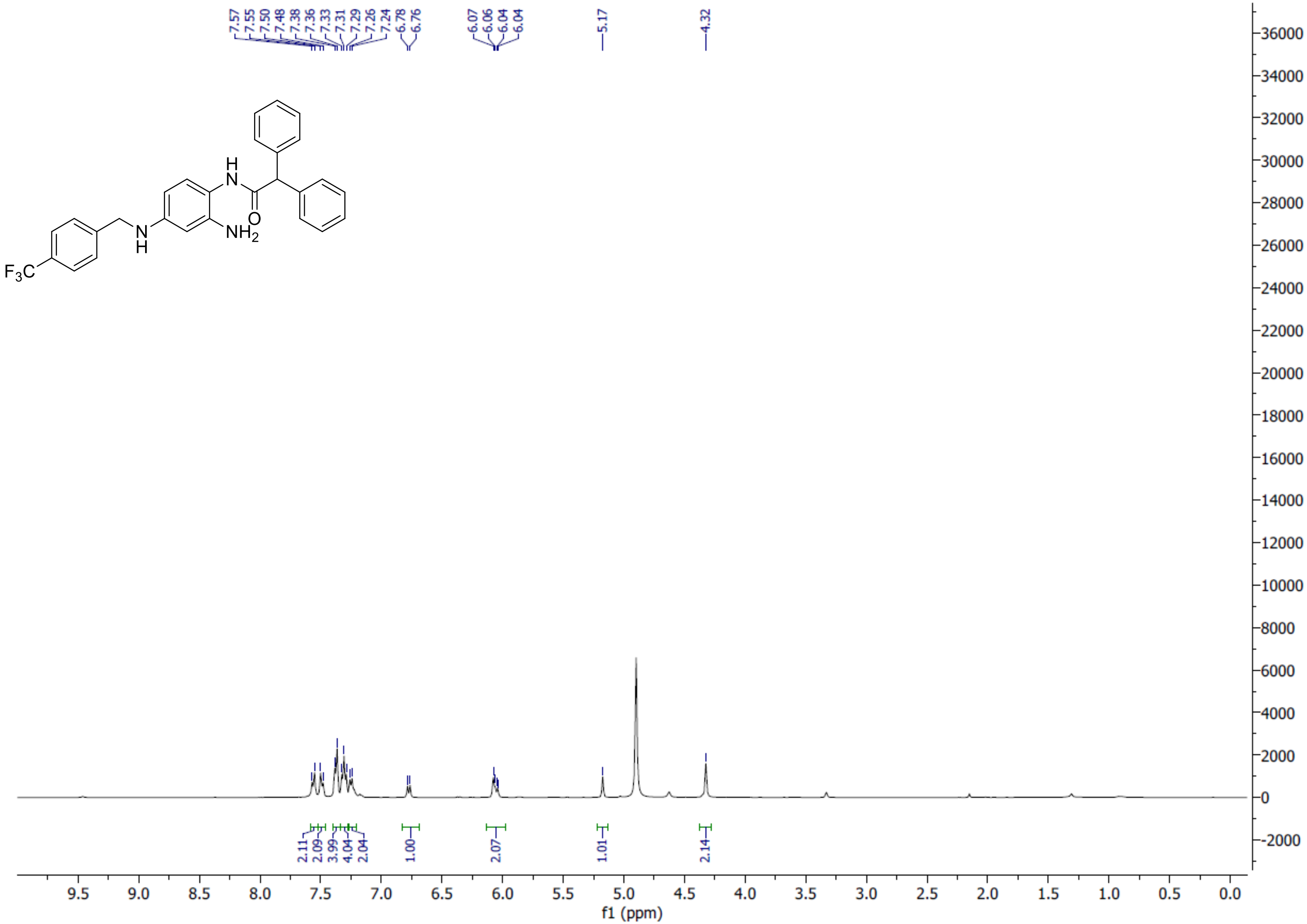

Figure S25: Compound **c106** ^{1}H-NMR spectrum spectra

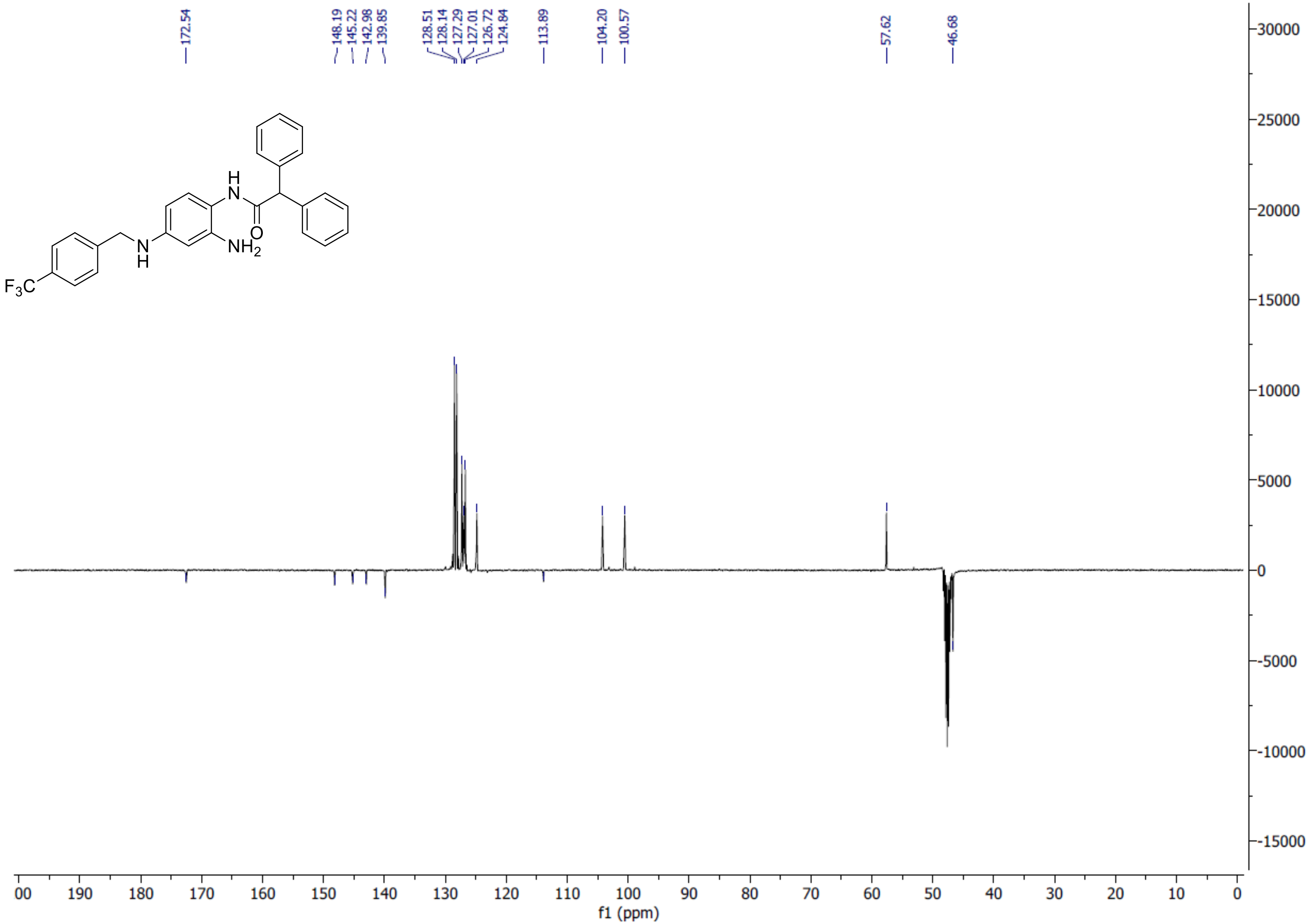

Figure S26: Compound **c106 q**DEPT NMR spectrum

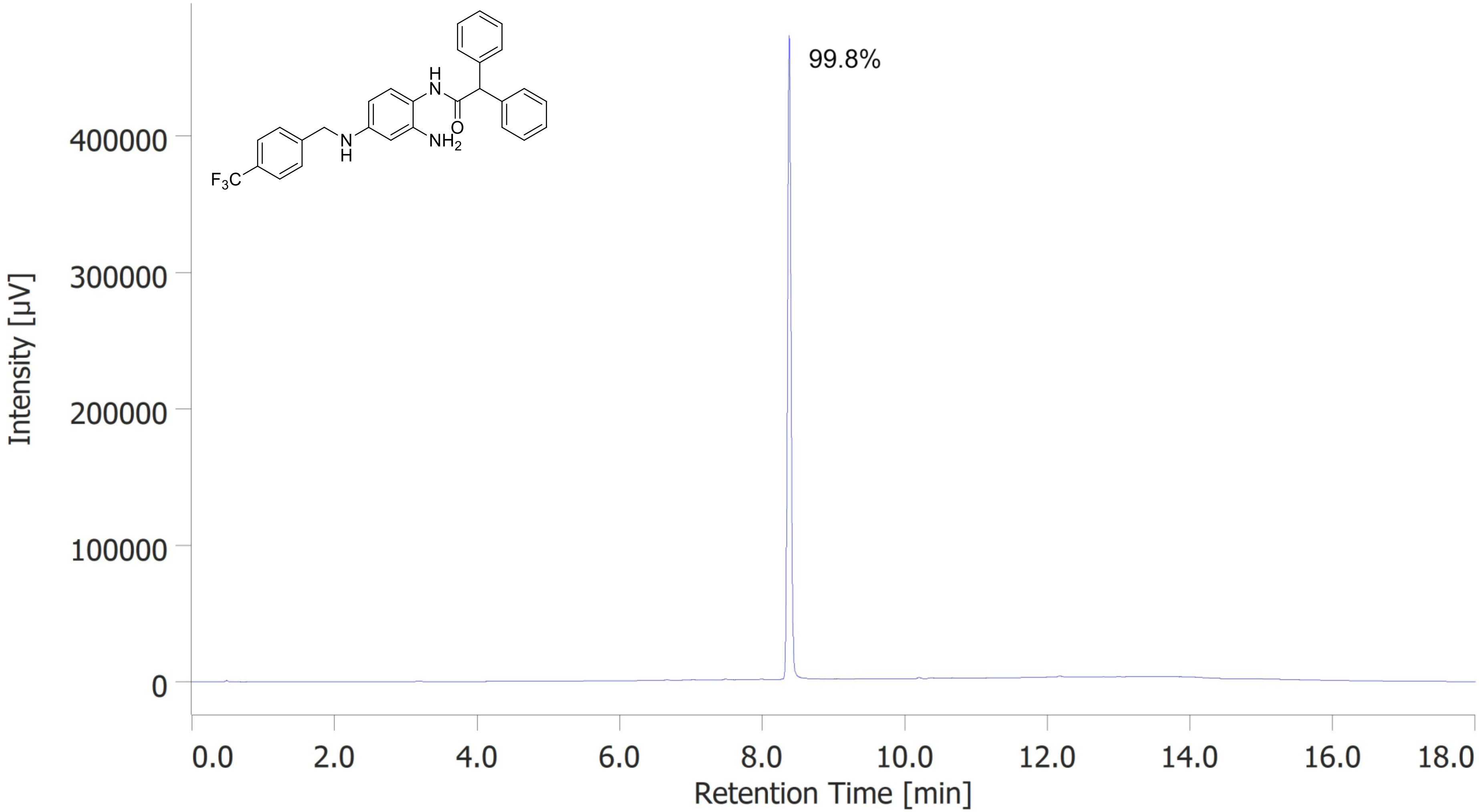

Figure S27: Compound **c106** HPLC trace

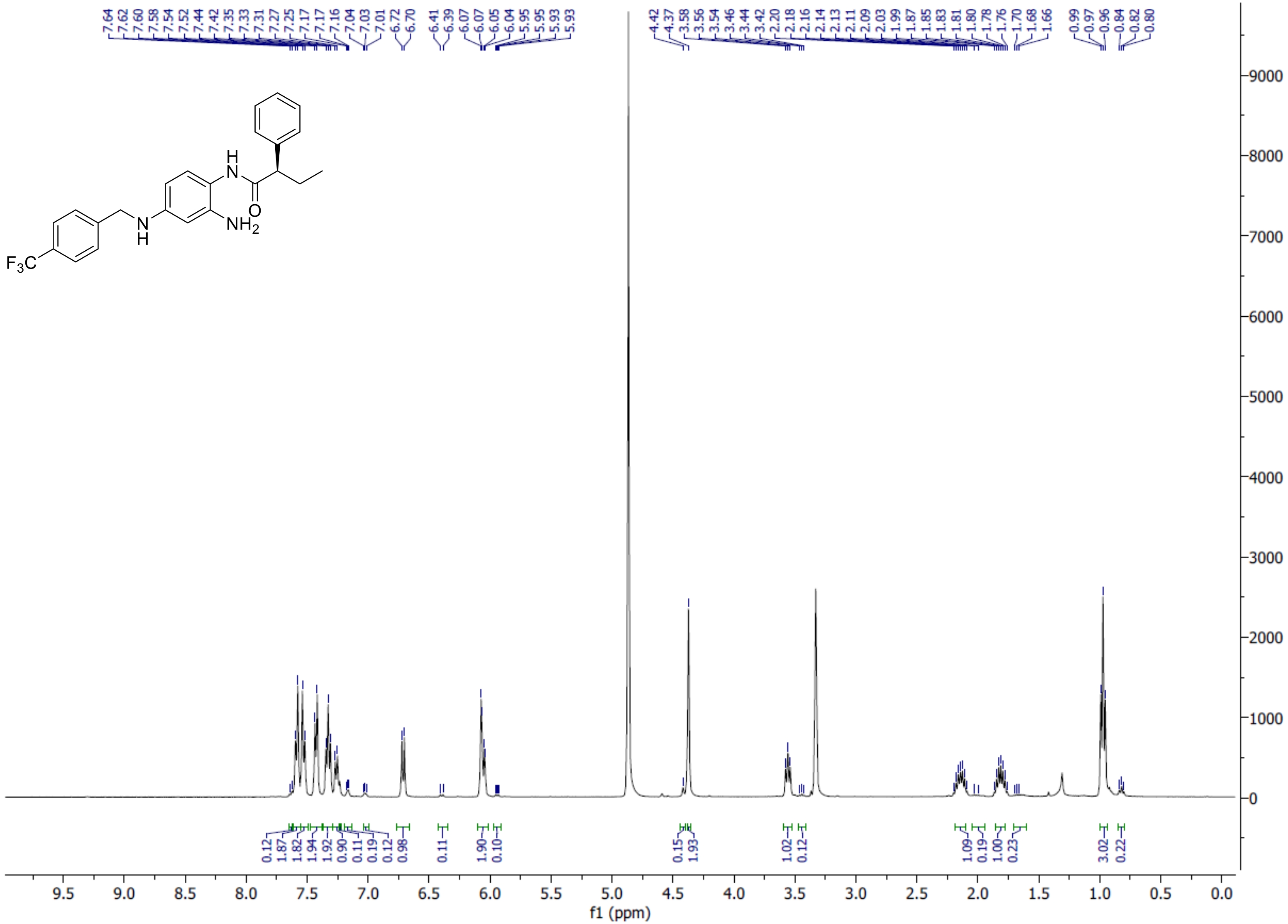

Figure S28: Compound **c107R** ^{1}H-NMR spectrum

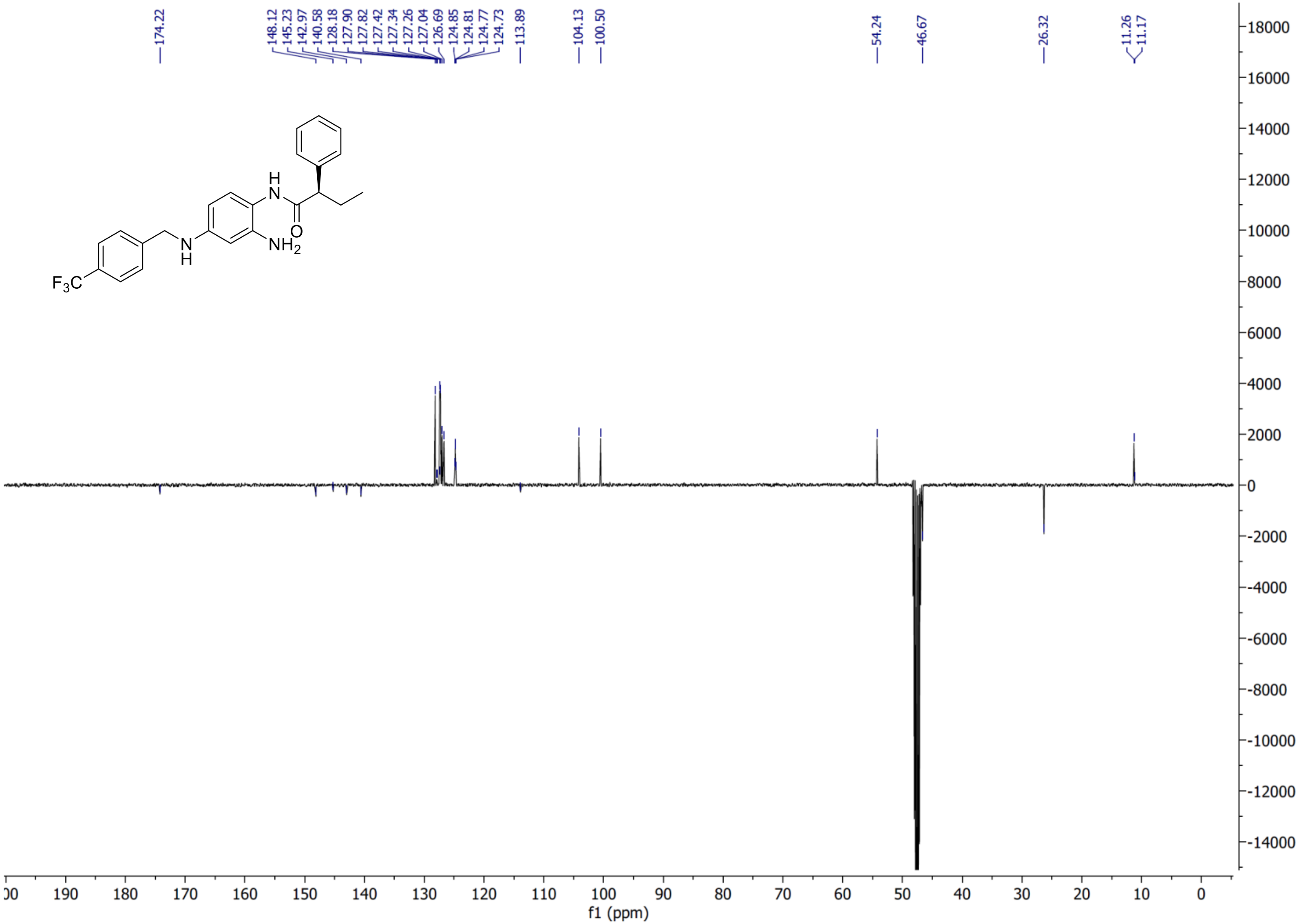

Figure S29: Compound **c107R q**DEPT NMR spectrum

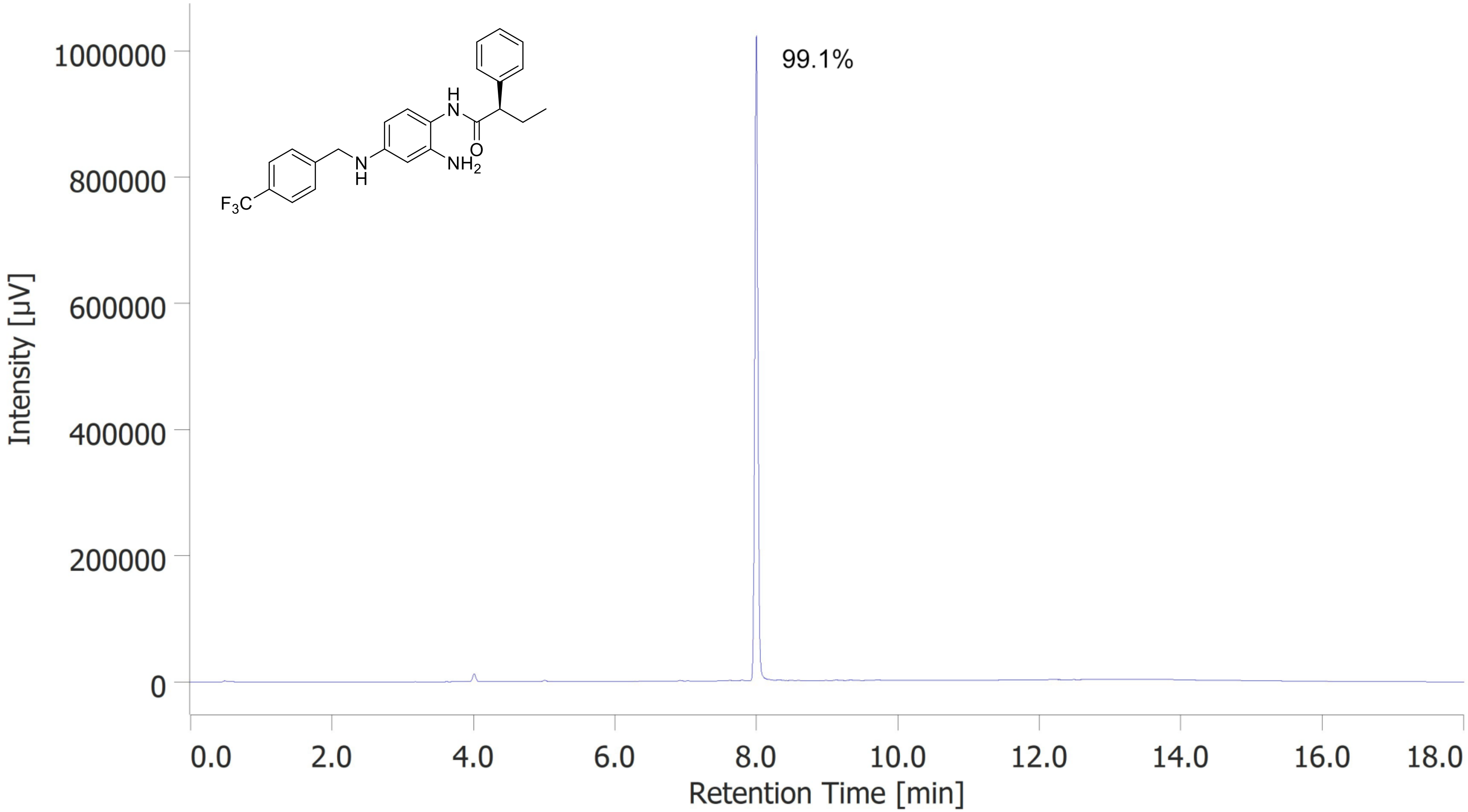

Figure S30: Compound **c107R** HPLC trace

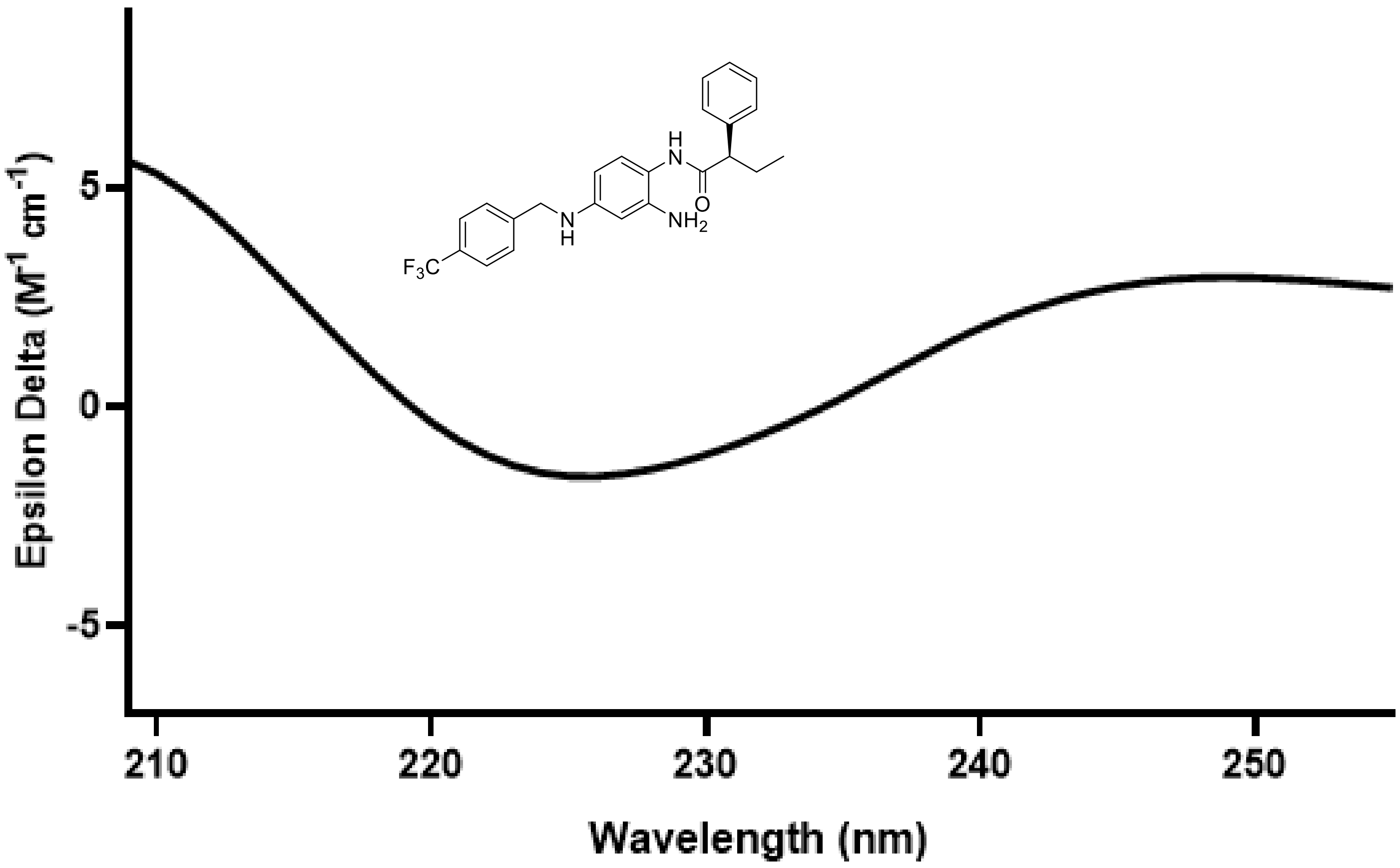

Figure S31: Compound **c107R** CD spectrum

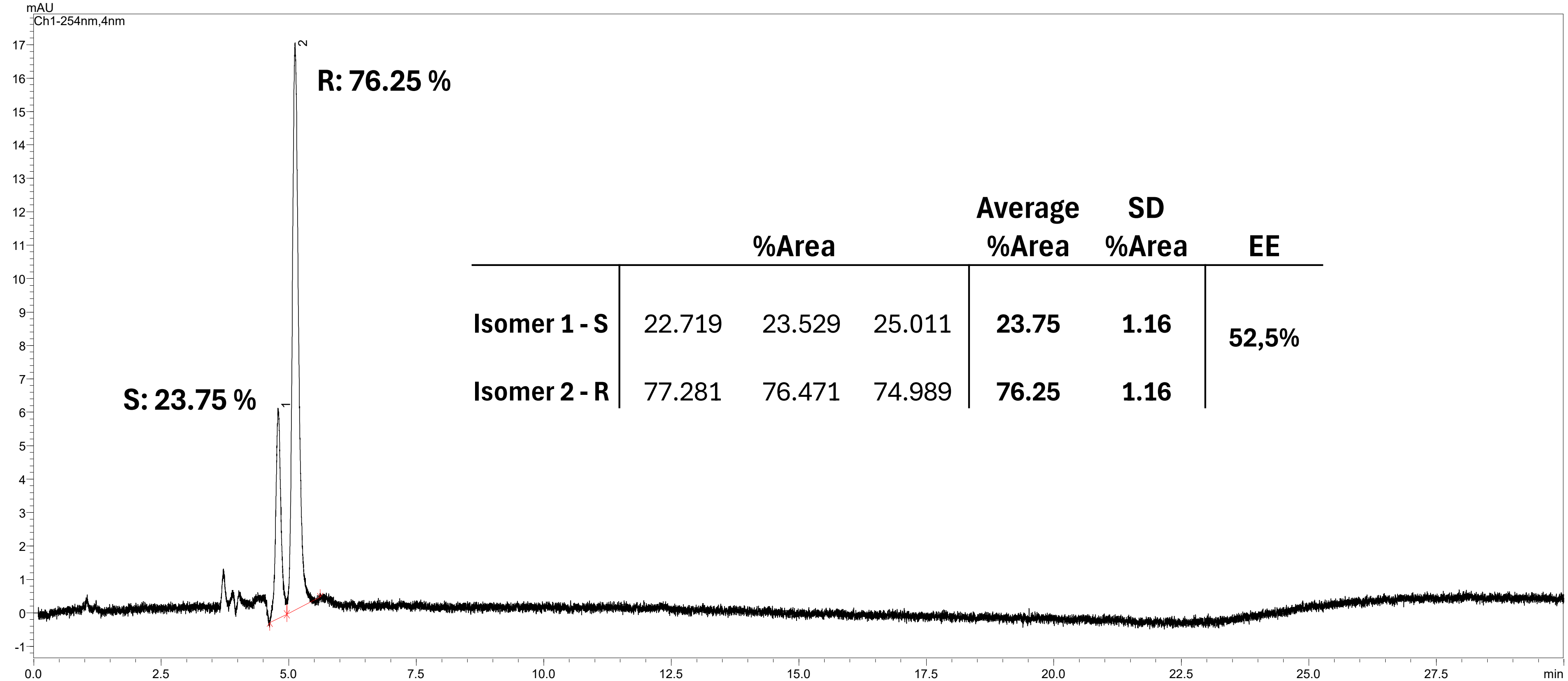

	%Area			Average %Area	SD %Area	EE
**Isomer 1 - S**	22.719	23.529	25.011	**23.75**	**1.16**	**52,5%**
**Isomer 2 - R**	77.281	76.471	74.989	**76.25**	**1.16**	

Figure S32: Compound **c107R** chiral HPLC trace with calculated enantiomeric excess (EE)

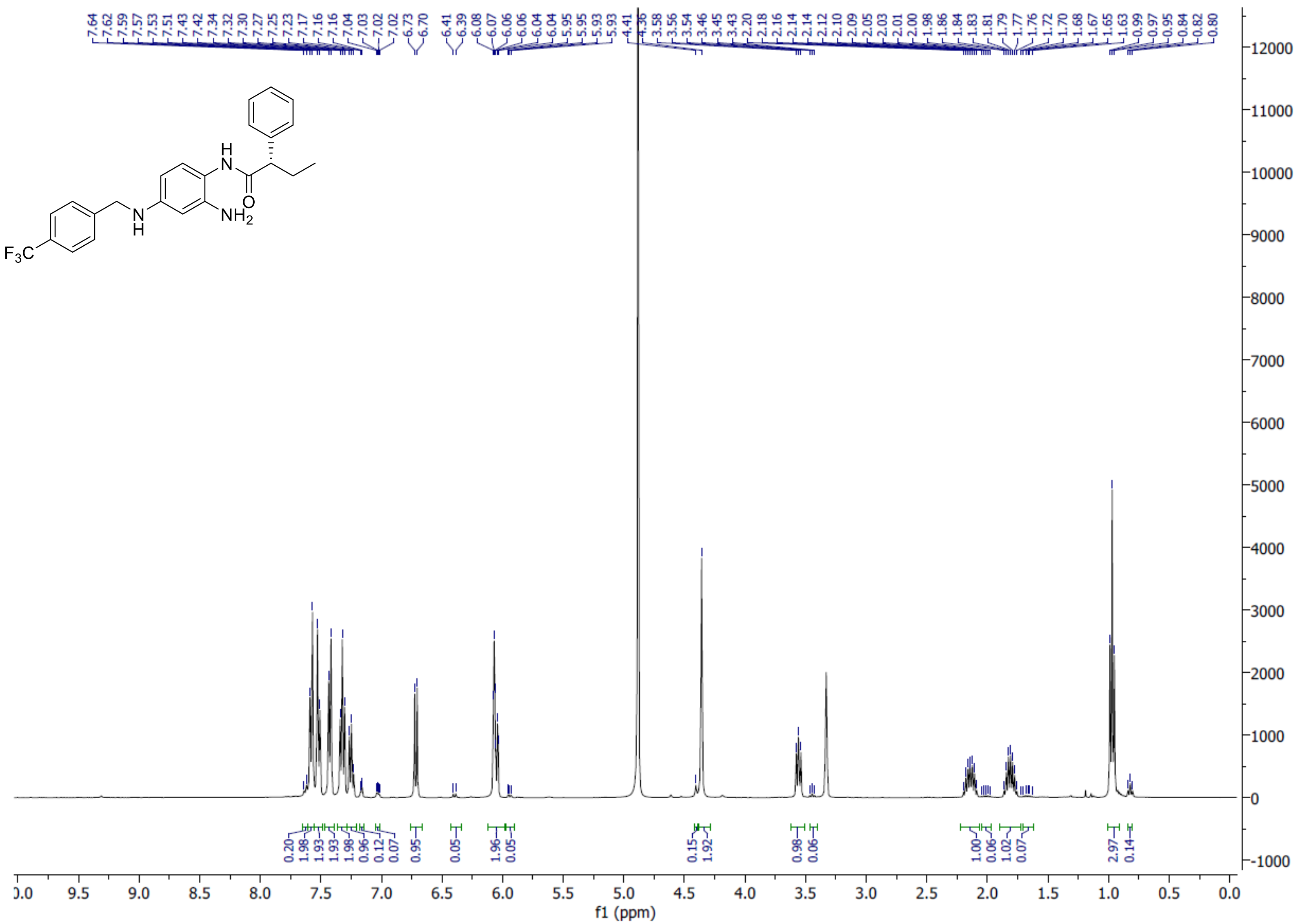

Figure S33: Compound **c107S** ^{1}H-NMR spectrum

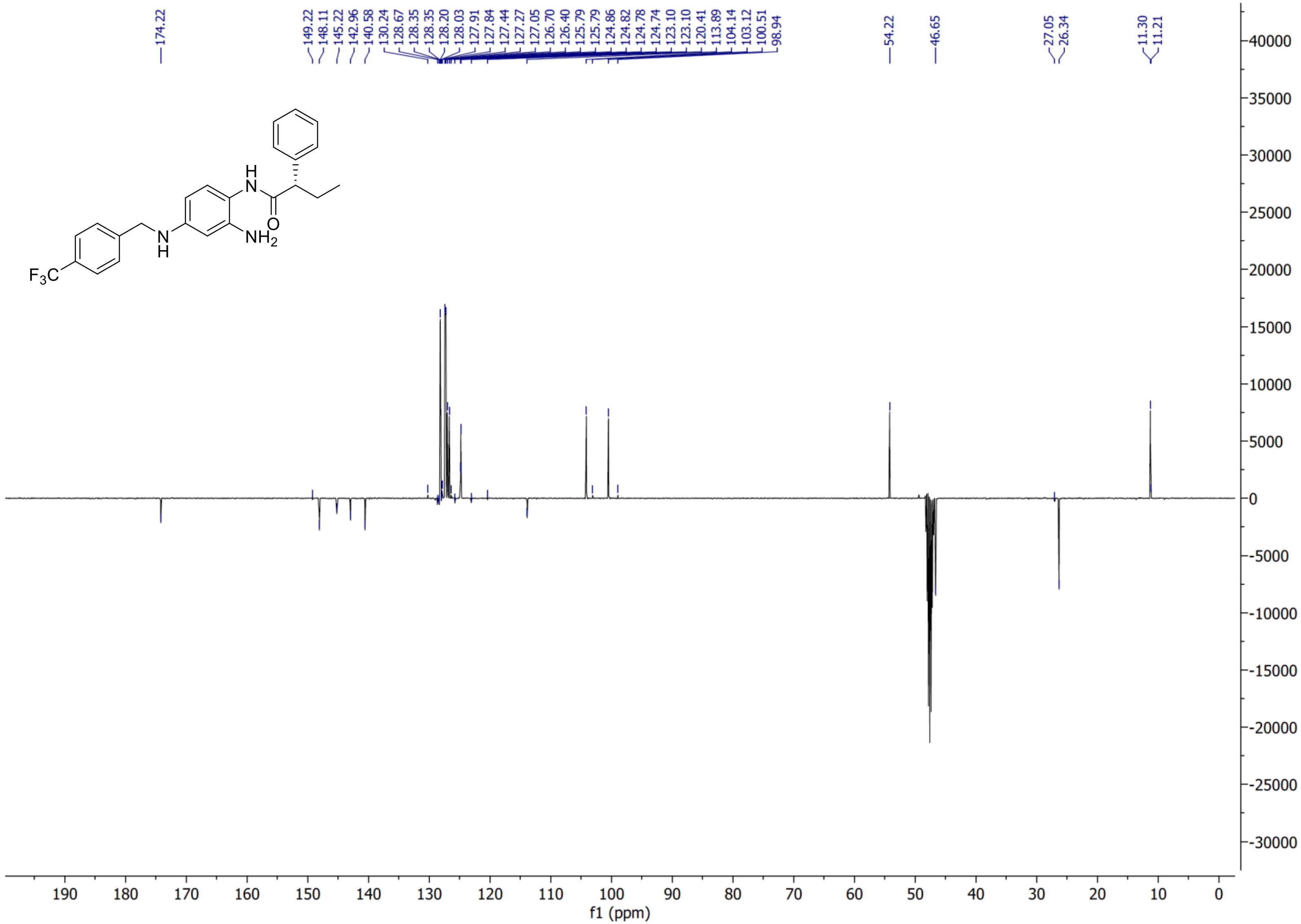

Figure S34: Compound **c107S q**DEPT NMR spectrum

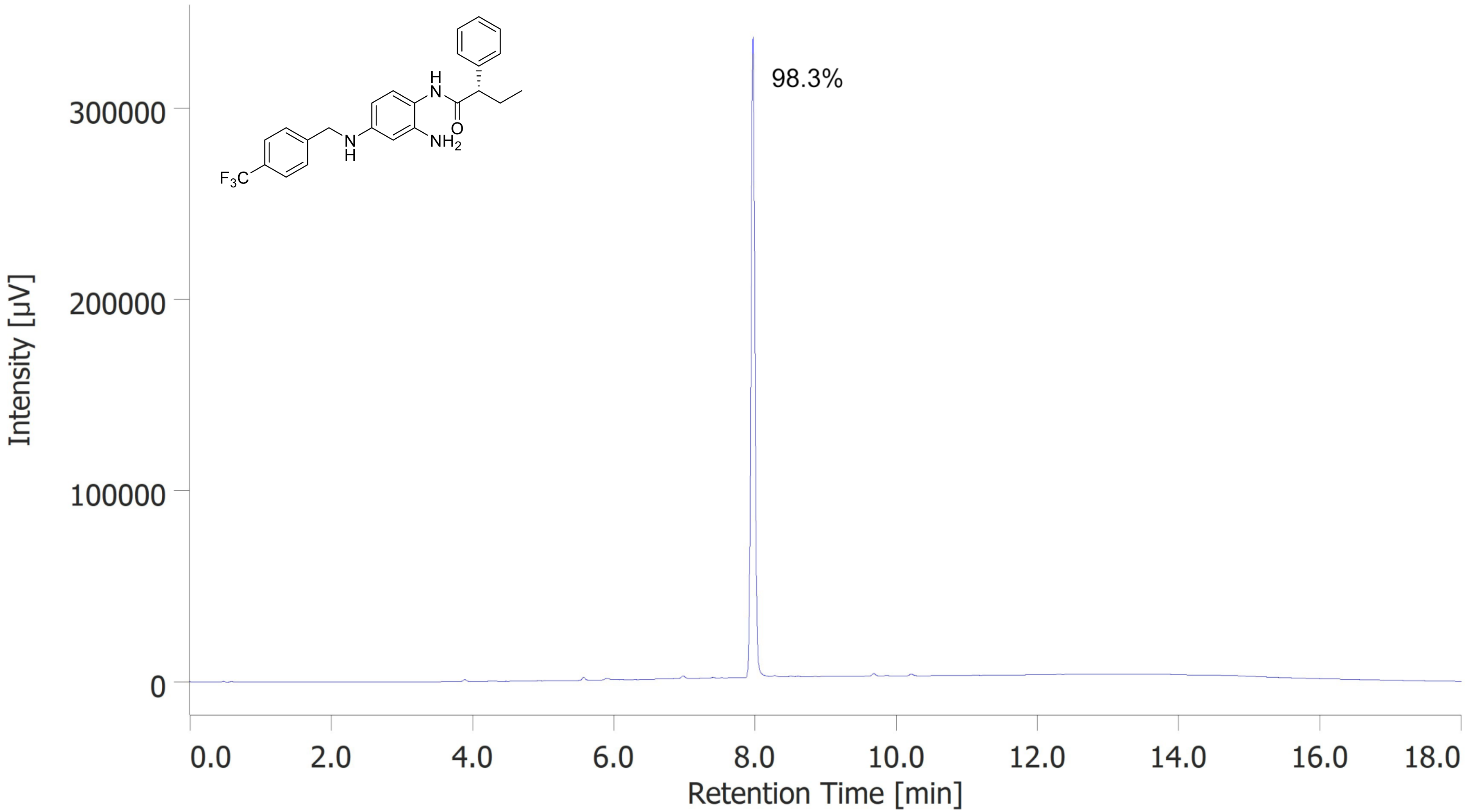

Figure S35: Compound **c107S** HPLC trace

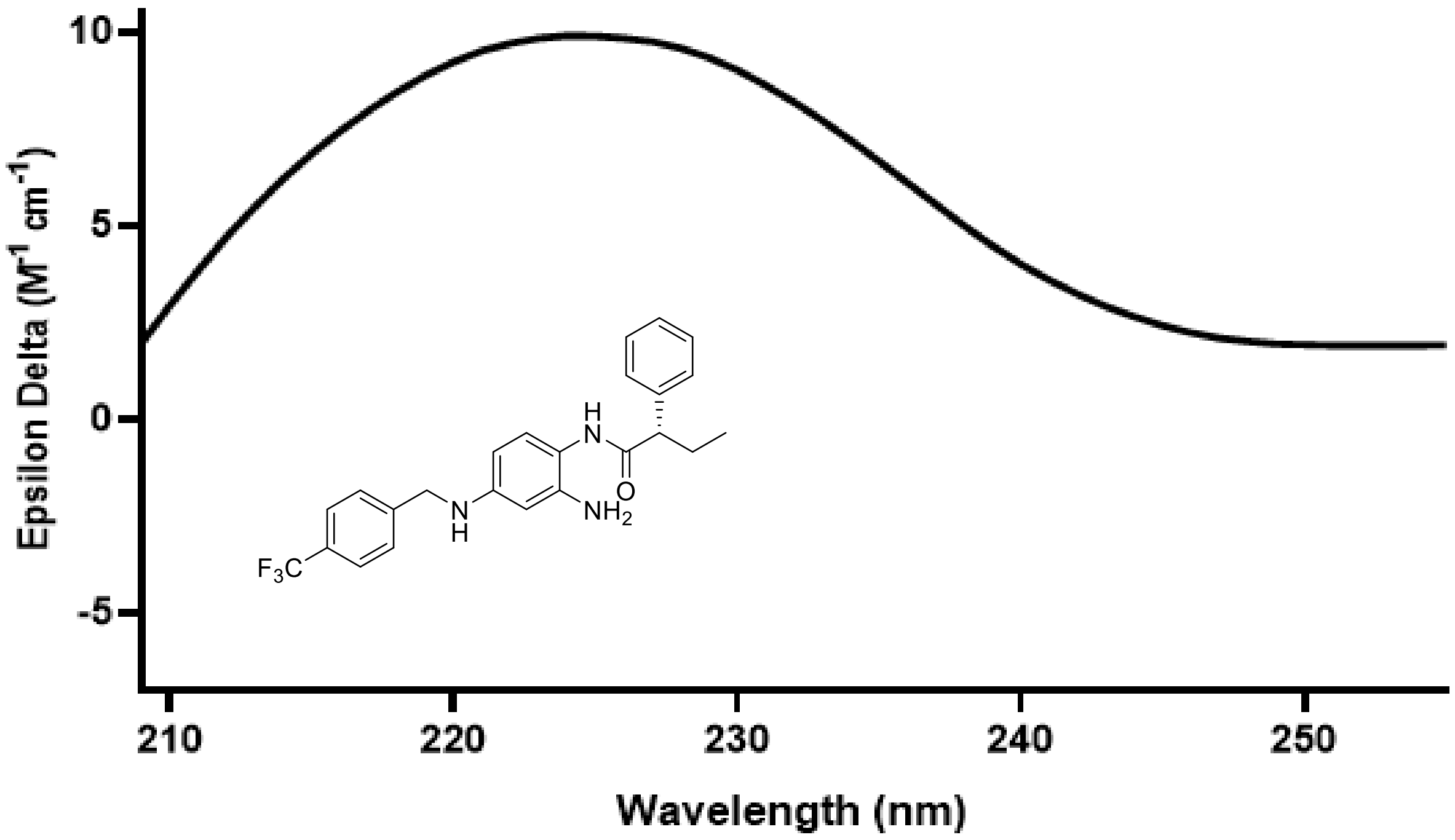

Figure S36: Compound **c107S** CD spectrum

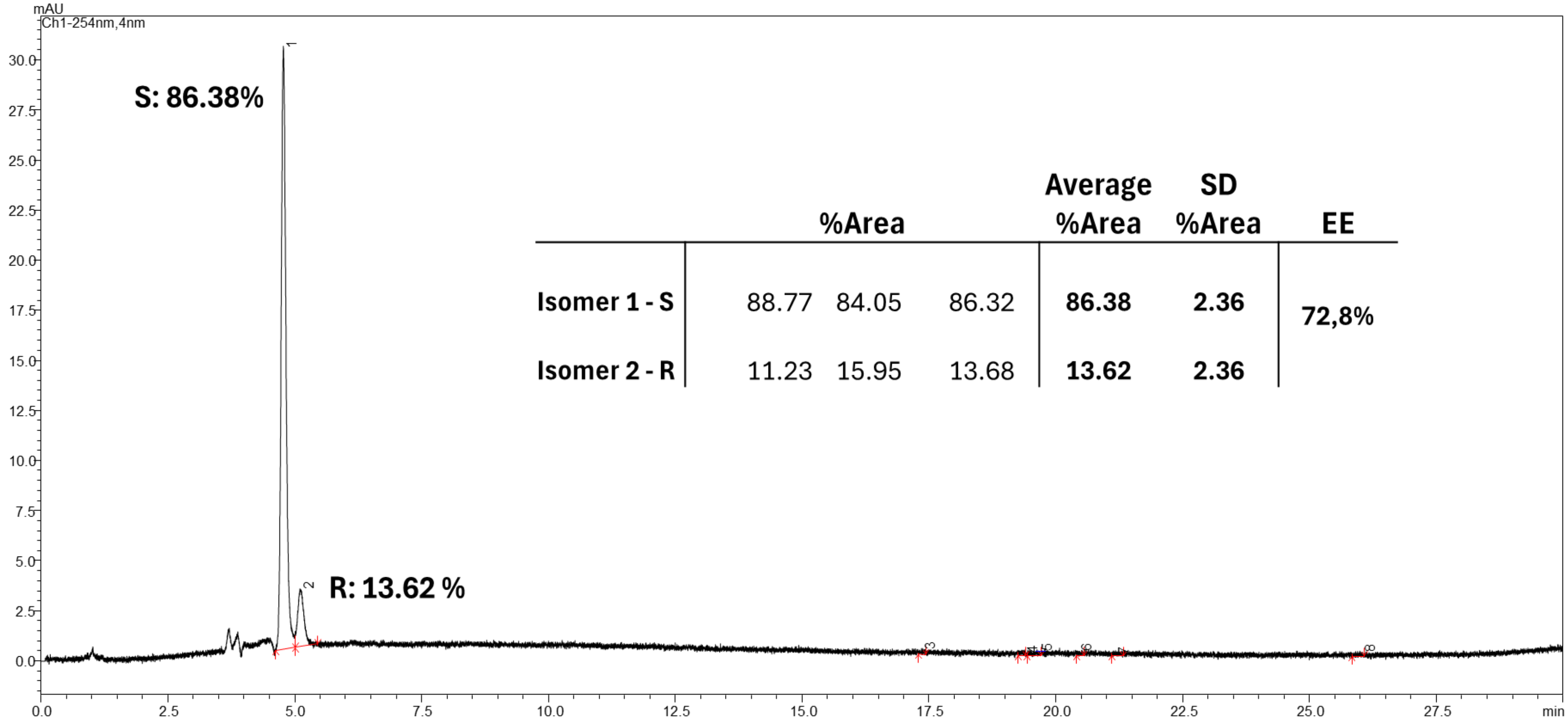

	%Area			Average %Area	SD %Area	EE
**Isomer 1 - S**	88.77	84.05	86.32	**86.38**	**2.36**	**72,8%**
**Isomer 2 - R**	11.23	15.95	13.68	**13.62**	**2.36**	

Figure S37: Compound **c107S** chiral HPLC trace with calculated enantiomeric excess (EE)

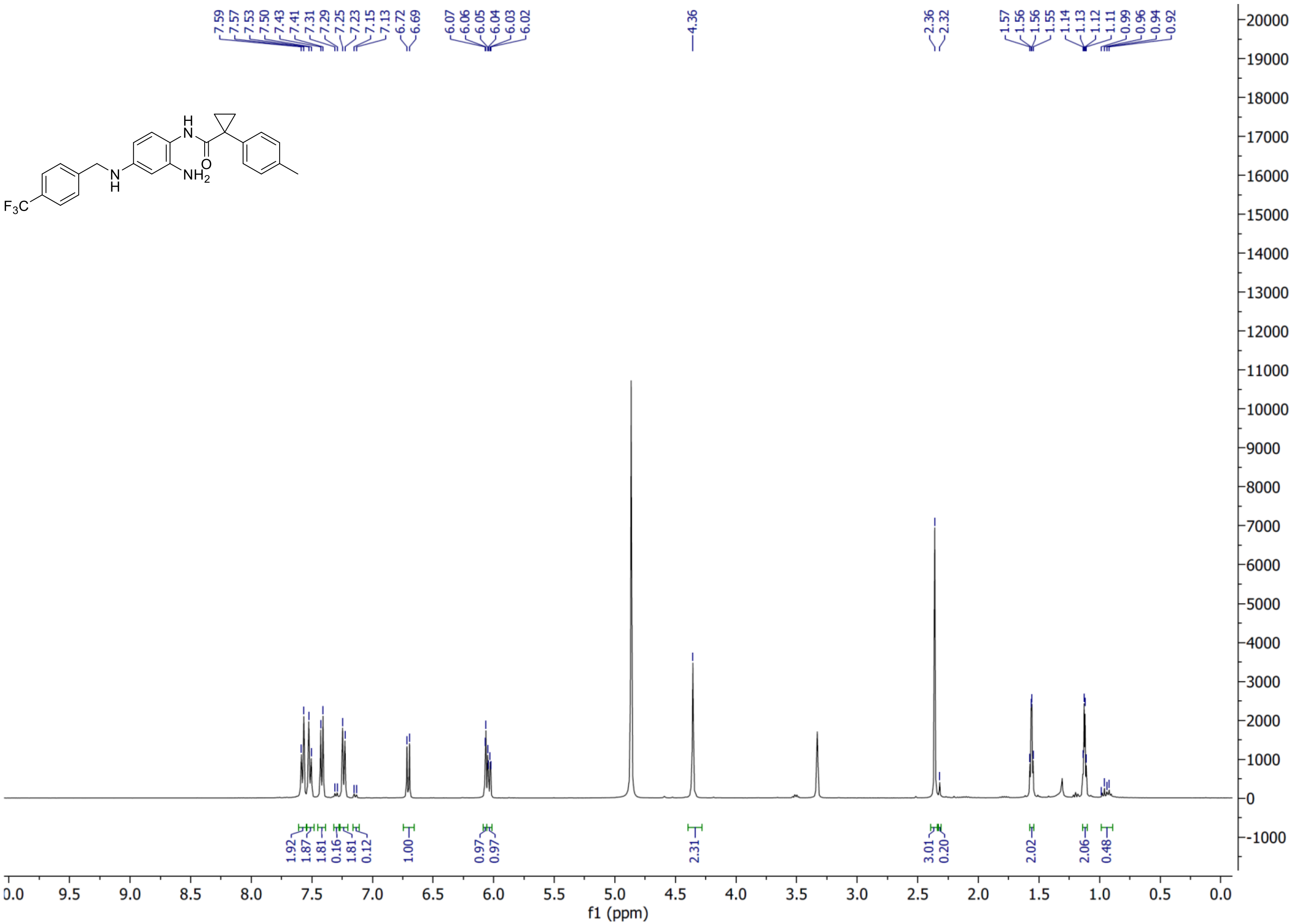

Figure S38: Compound **c108** ^{1}H-NMR spectrum

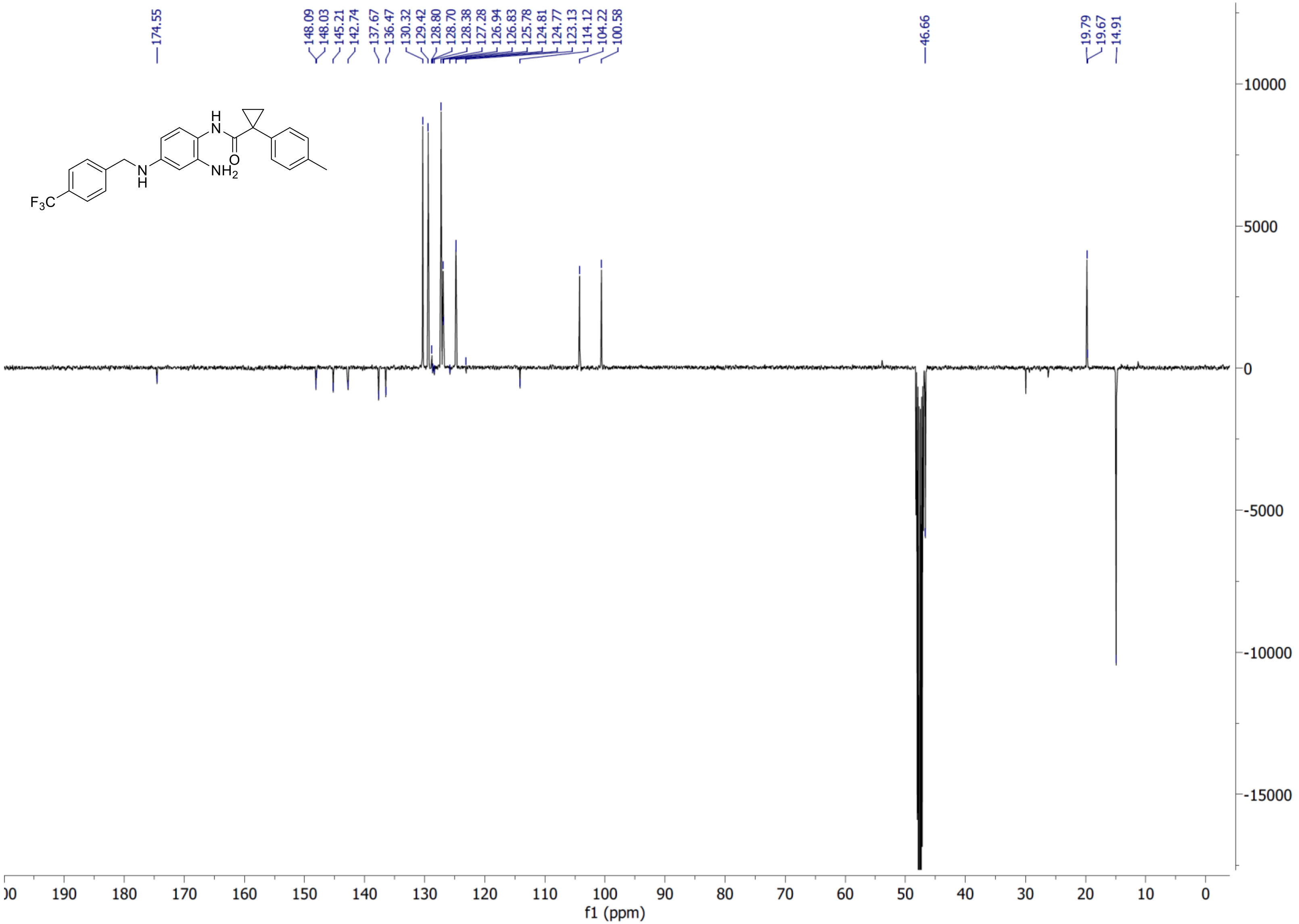

Figure S39: Compound **c108** qDEPT NMR spectrum

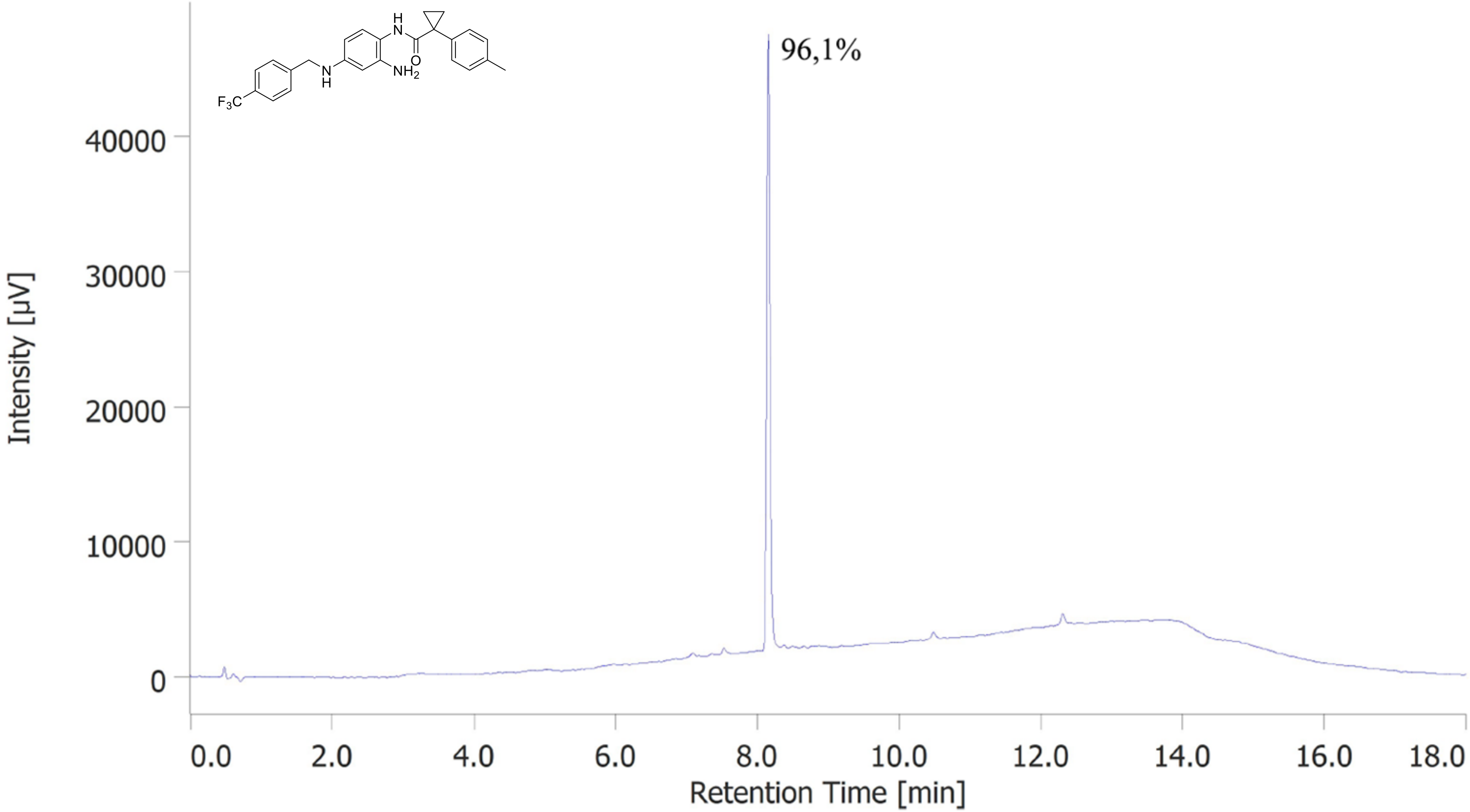

Figure S40: Compound **c108** HPLC trace

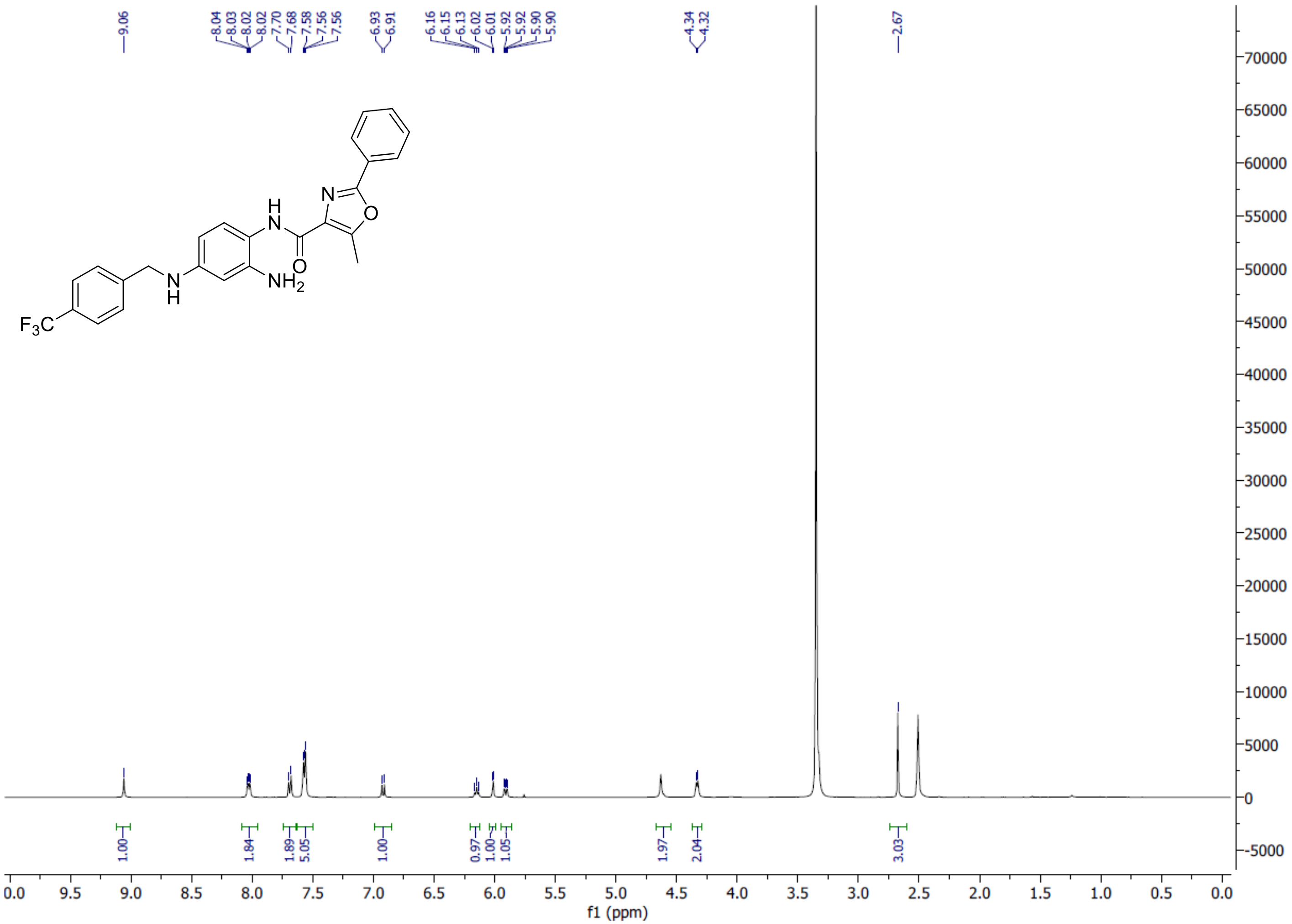

Figure S41: Compound **c109** ^{1}H-NMR spectrum

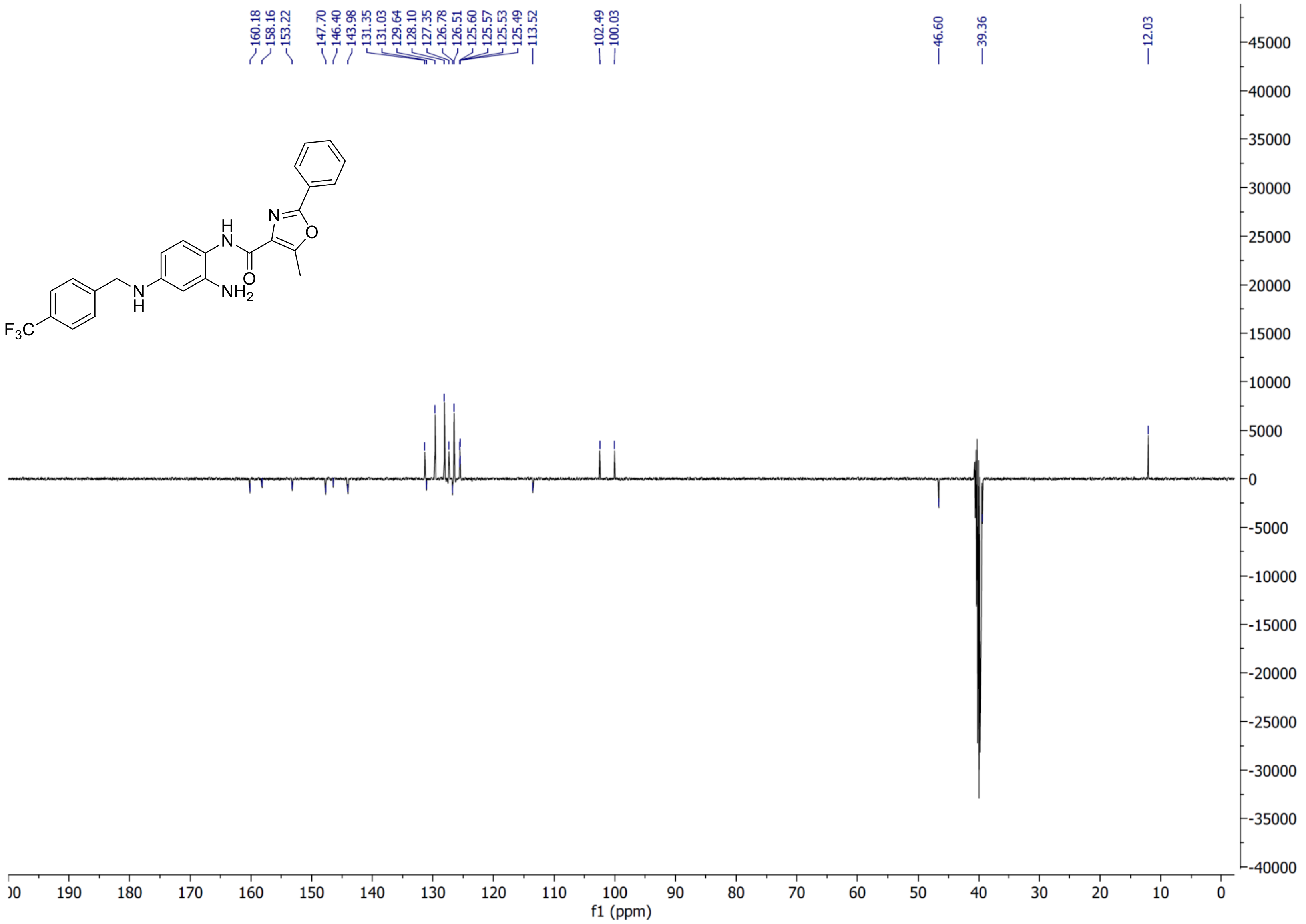

Figure S42: Compound **c109 q**DEPT NMR spectrum

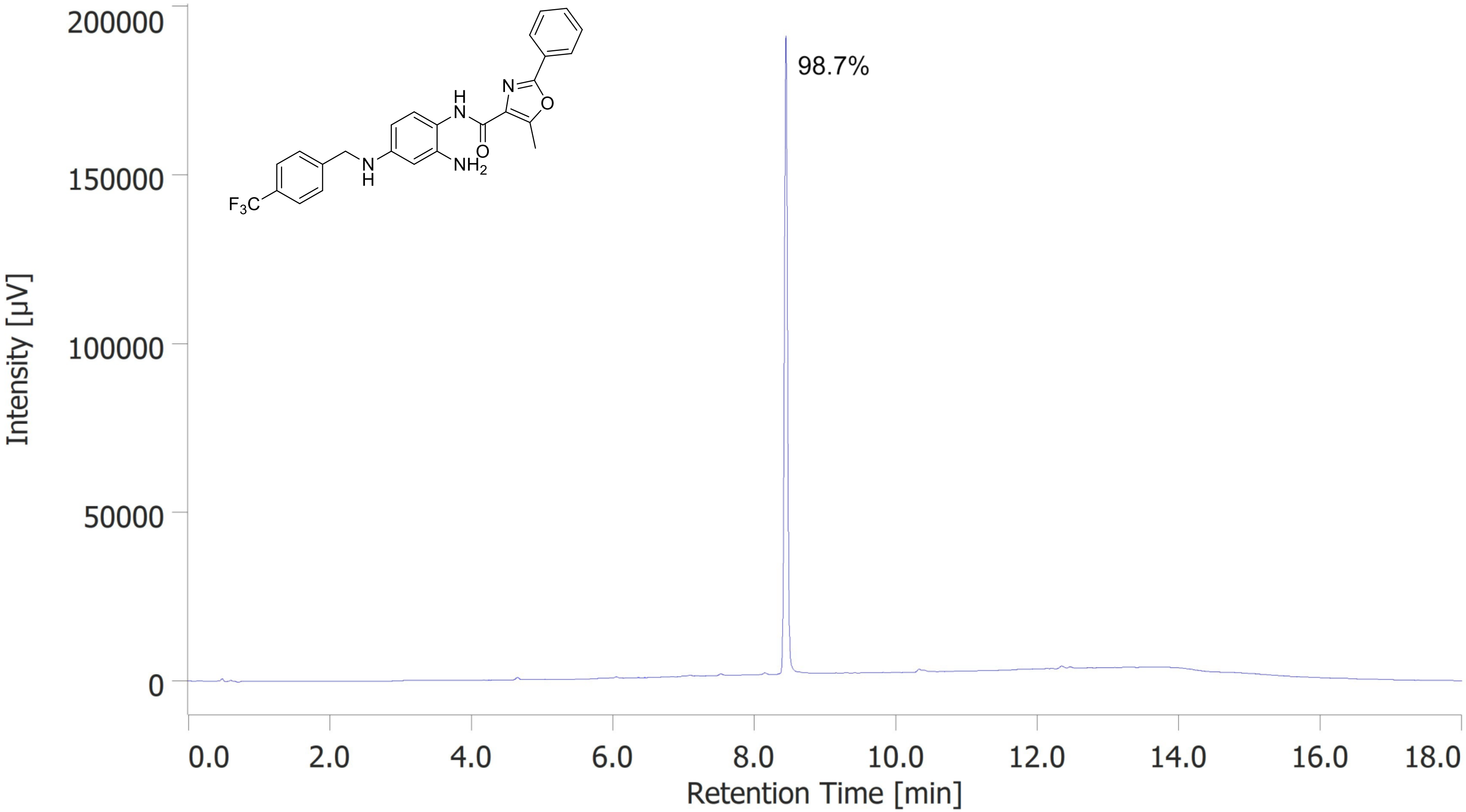

Figure S43: Compound **c109** HPLC trace

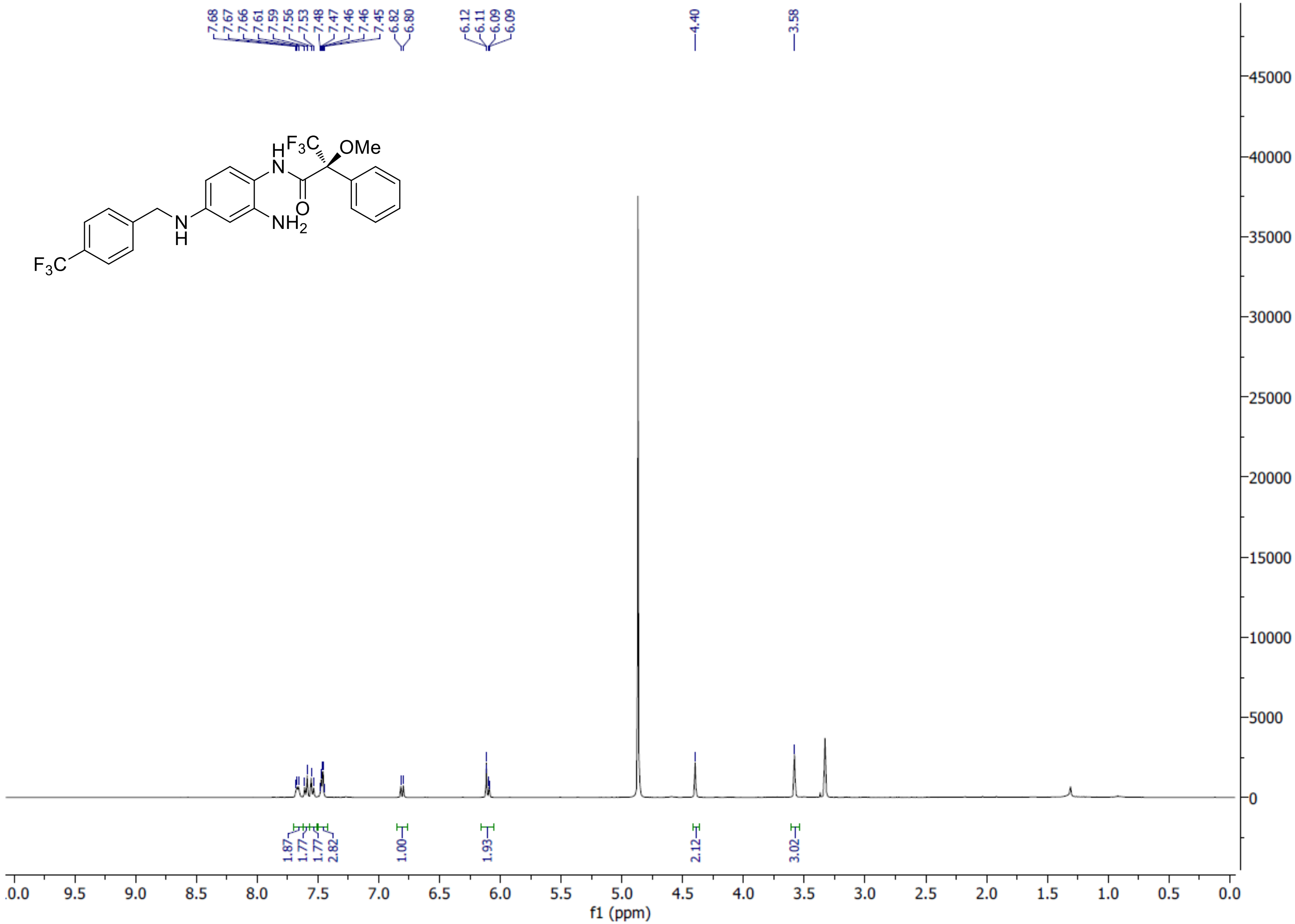

Figure S44: Compound **c111** ^{1}H-NMR spectrum

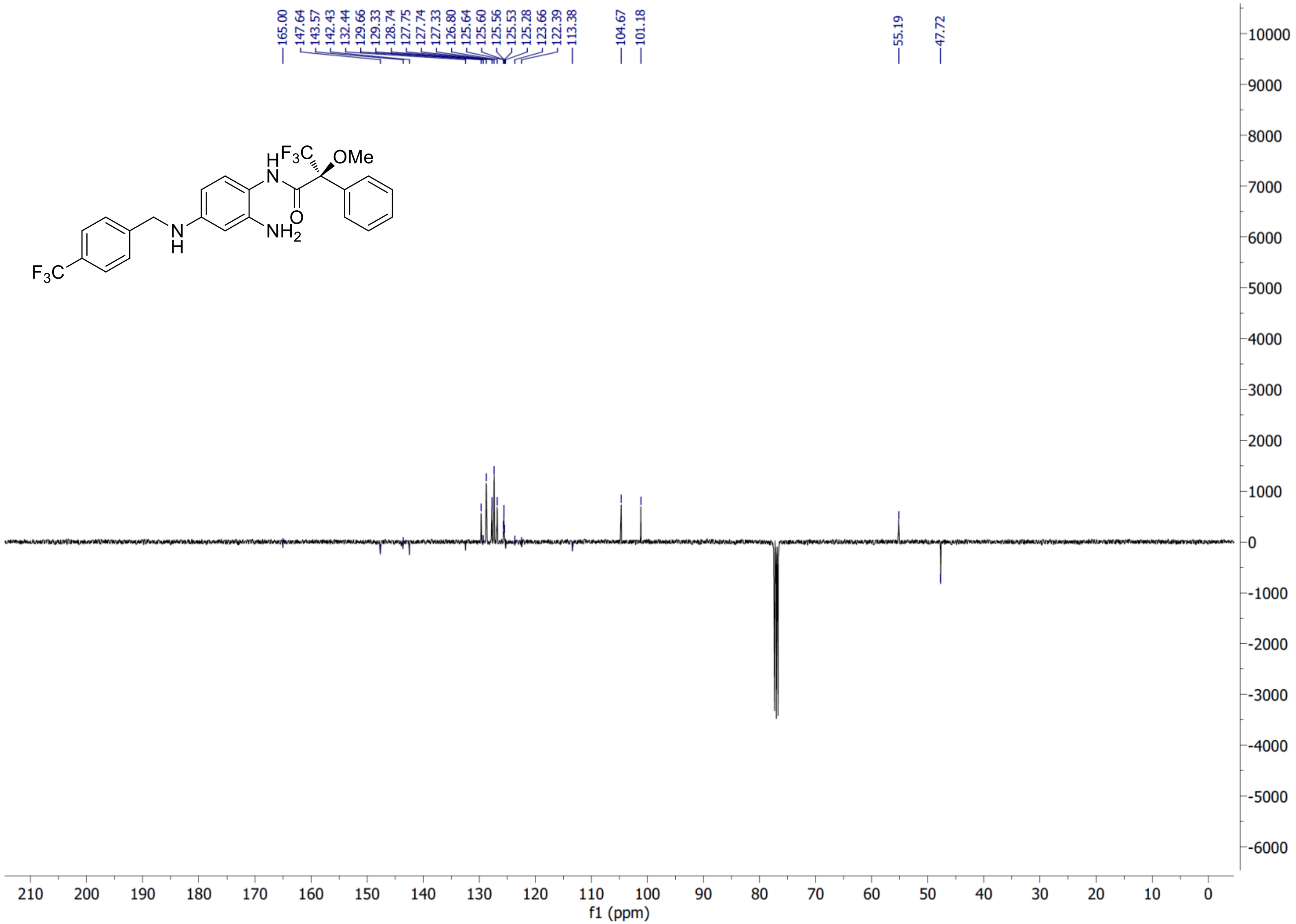

Figure S45: Compound **c111 q**DEPT NMR spectrum

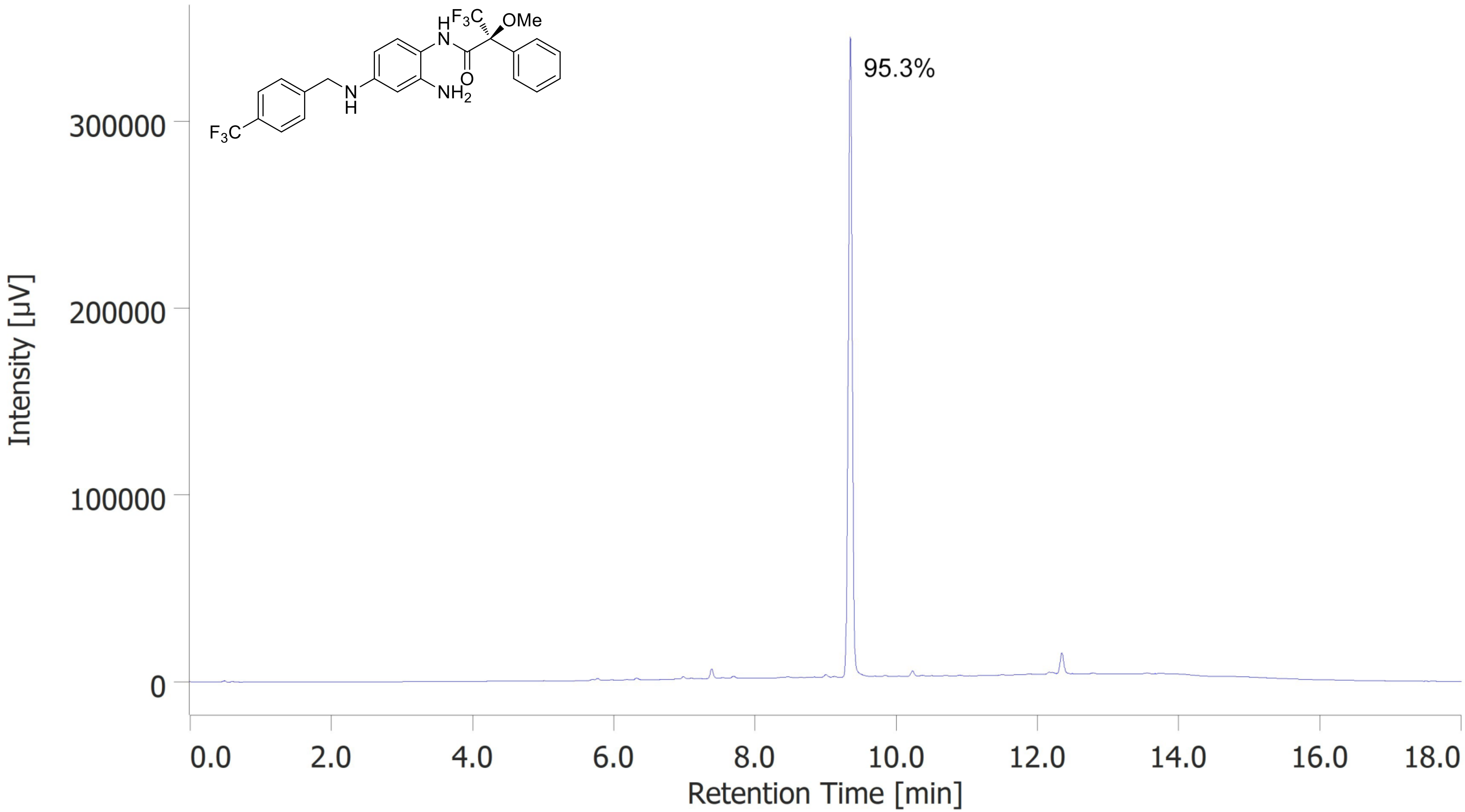

Figure S46: Compound **c111** HPLC trace

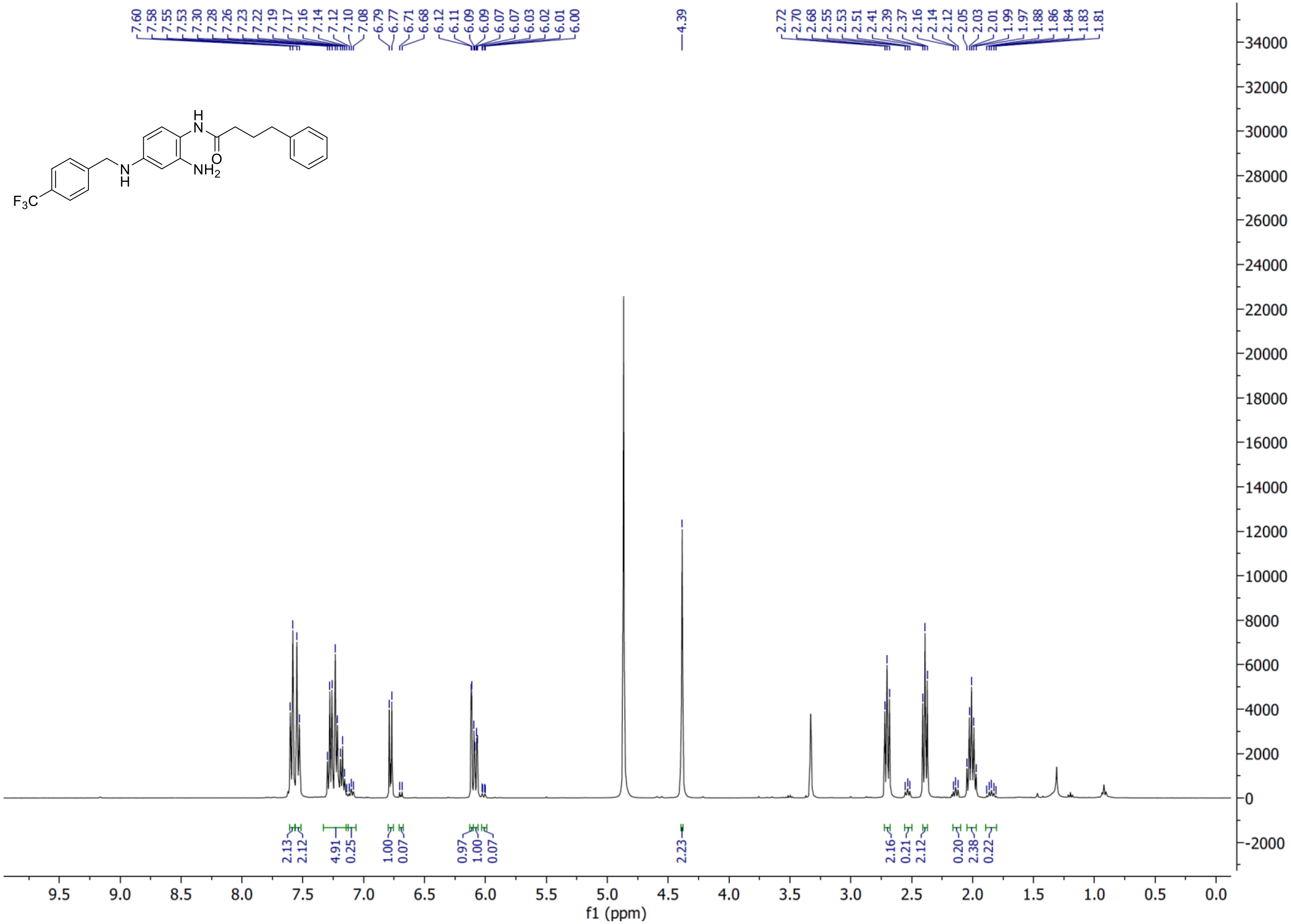

Figure S47: Compound **c112** ^{1}H-NMR spectrum

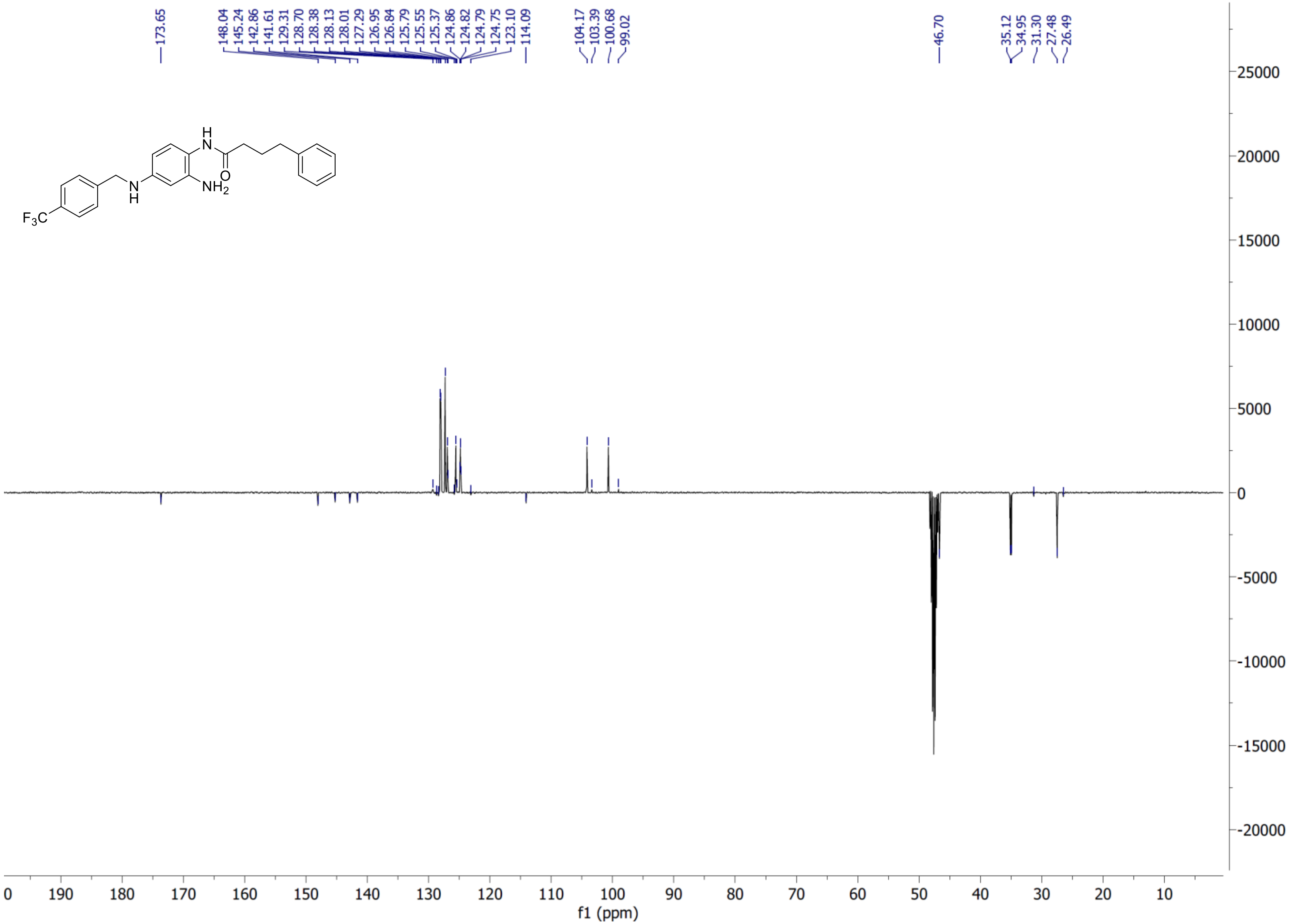

Figure S48: Compound **c112 q**DEPT NMR spectrum

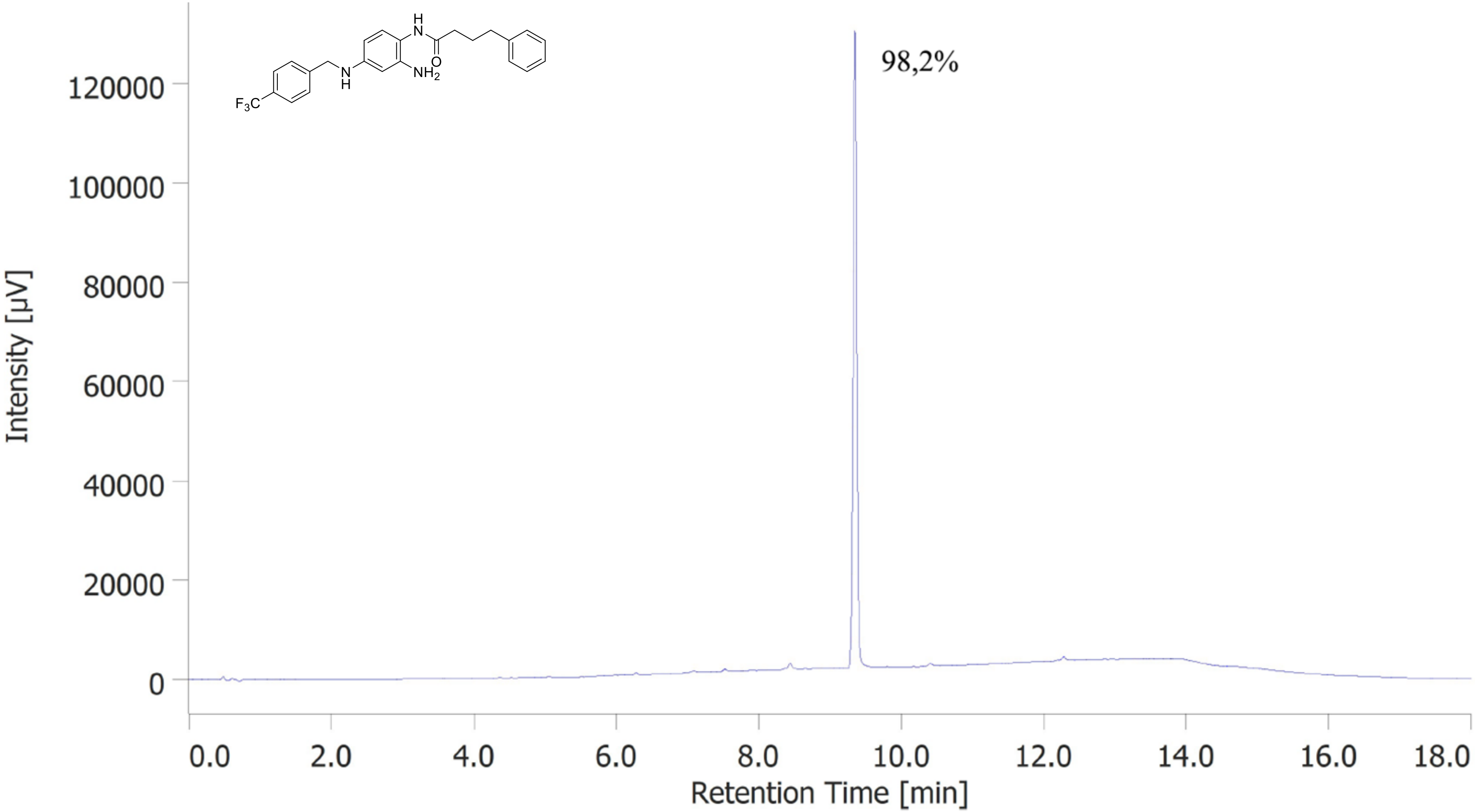

Figure S49: Compound **c112** HPLC trace

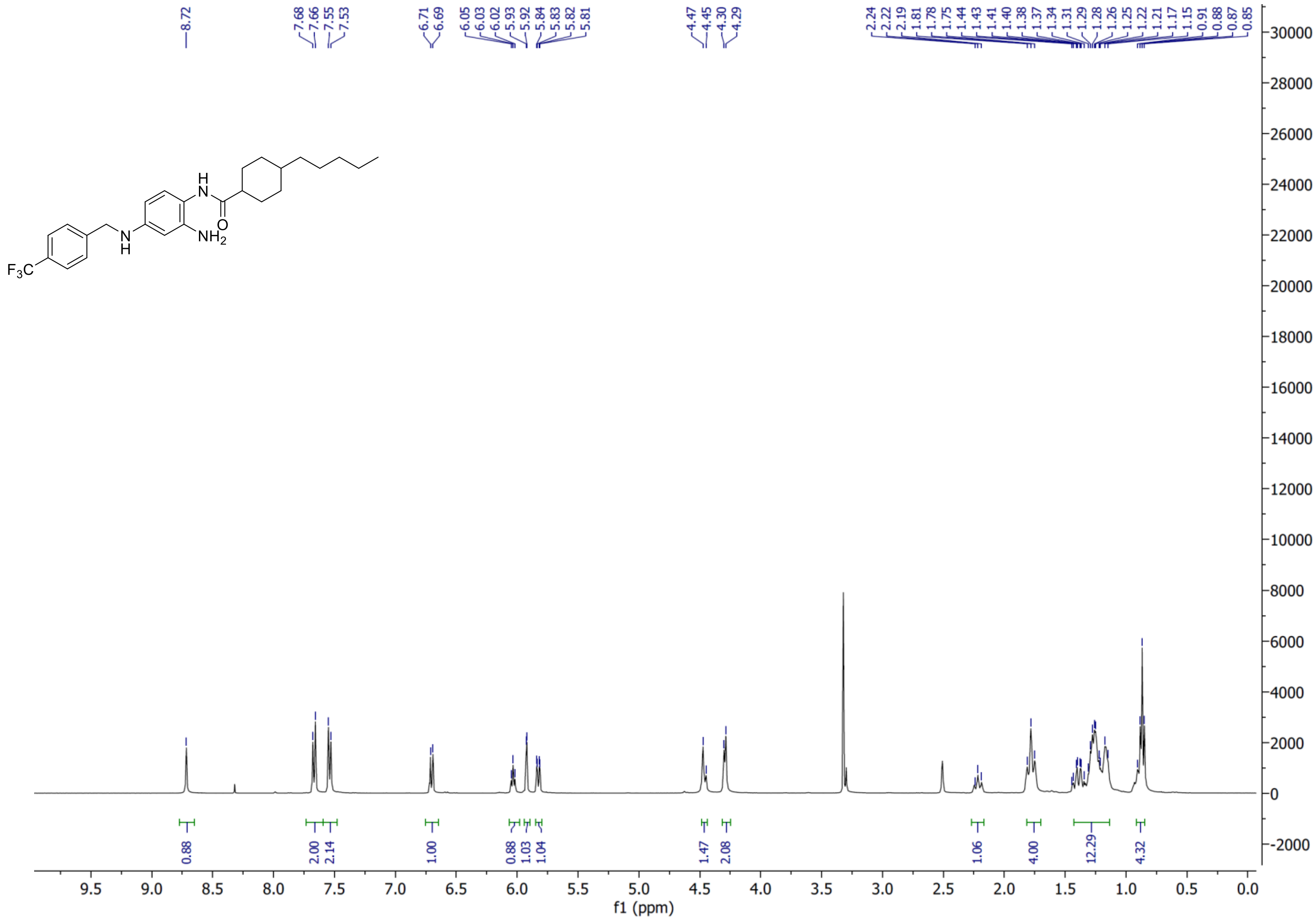

Figure S50: Compound **c113** ^{1}H-NMR spectrum

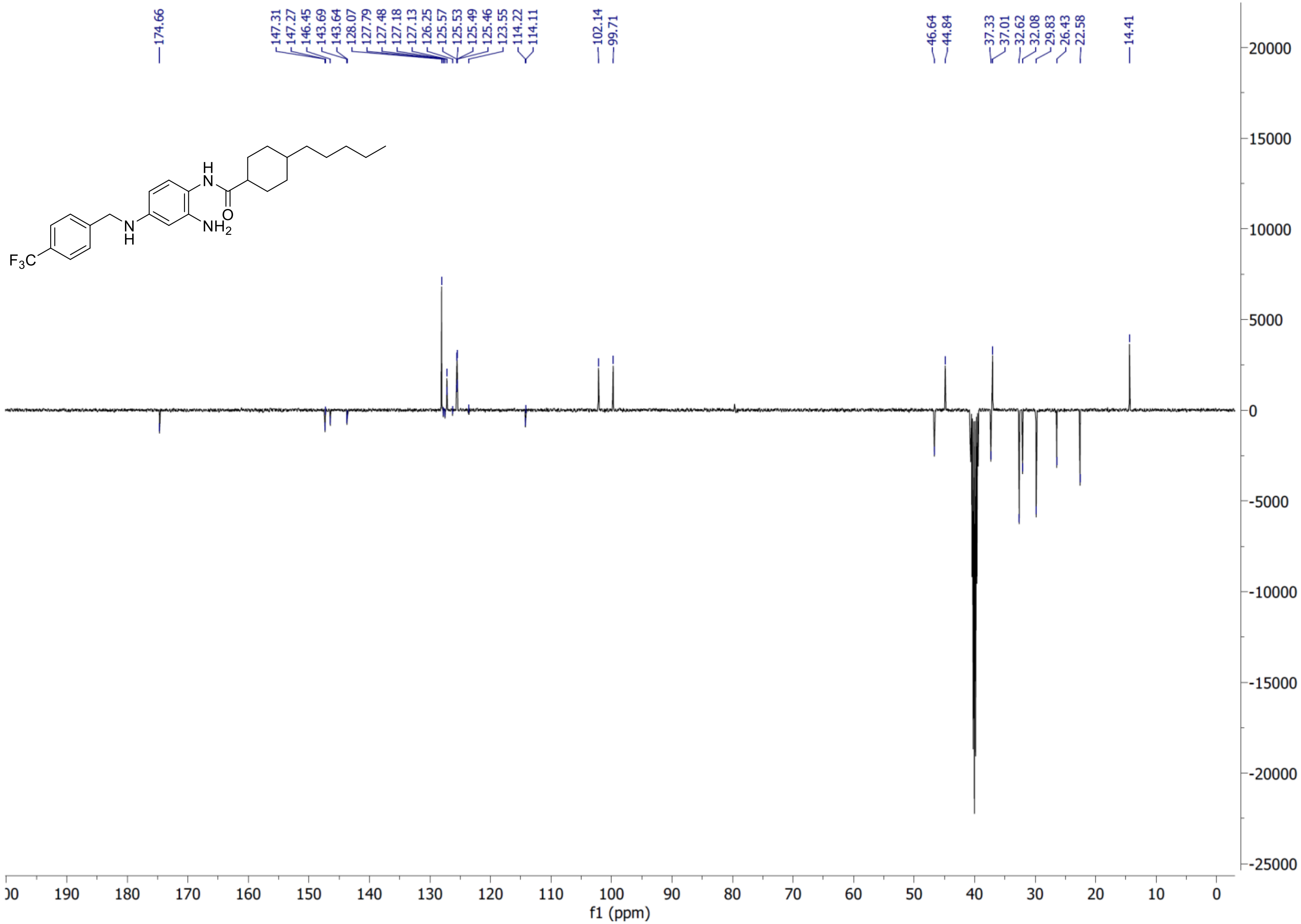

Figure S51: Compound **c113 q**DEPT NMR spectrum

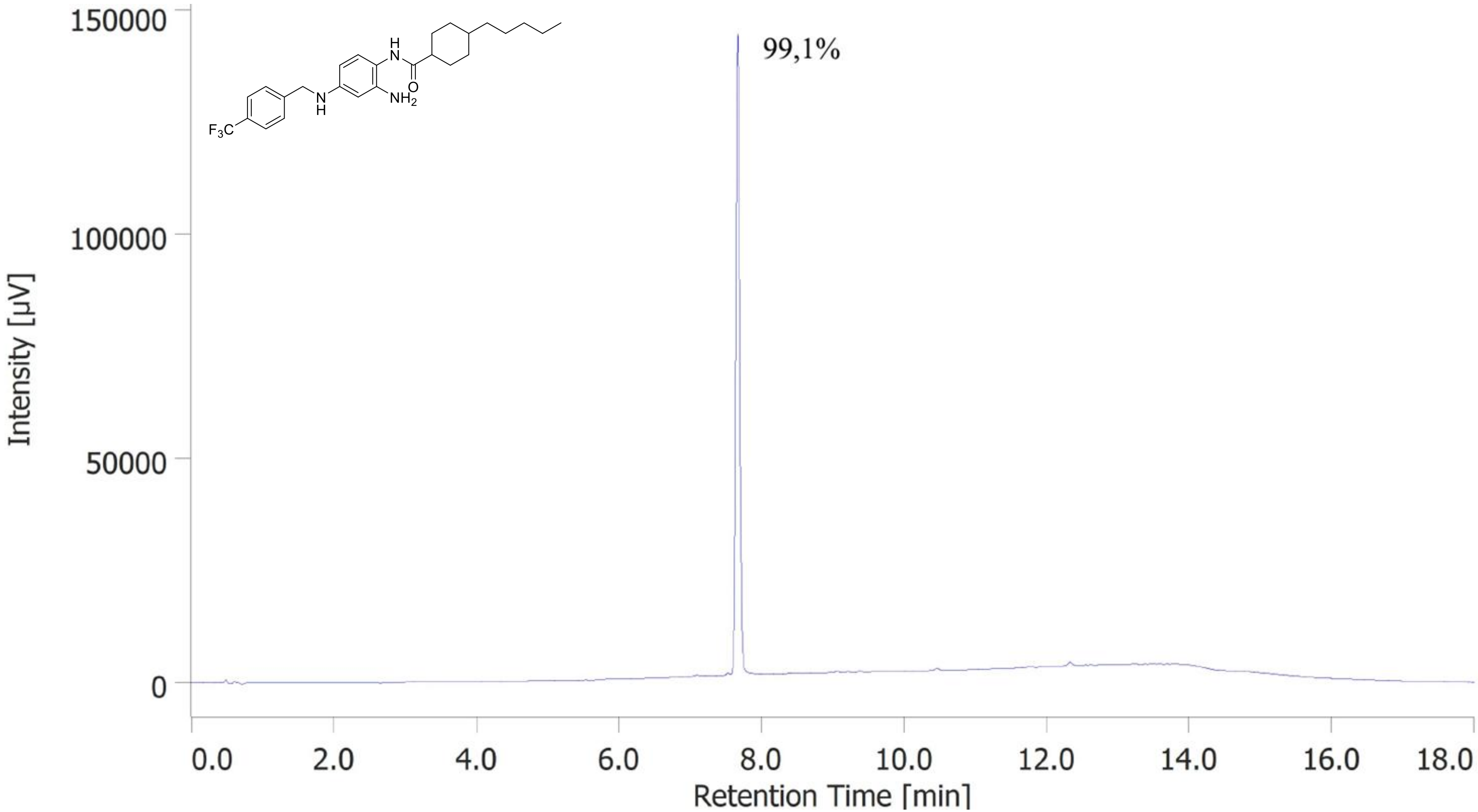

Figure S52: Compound **c113** HPLC trace

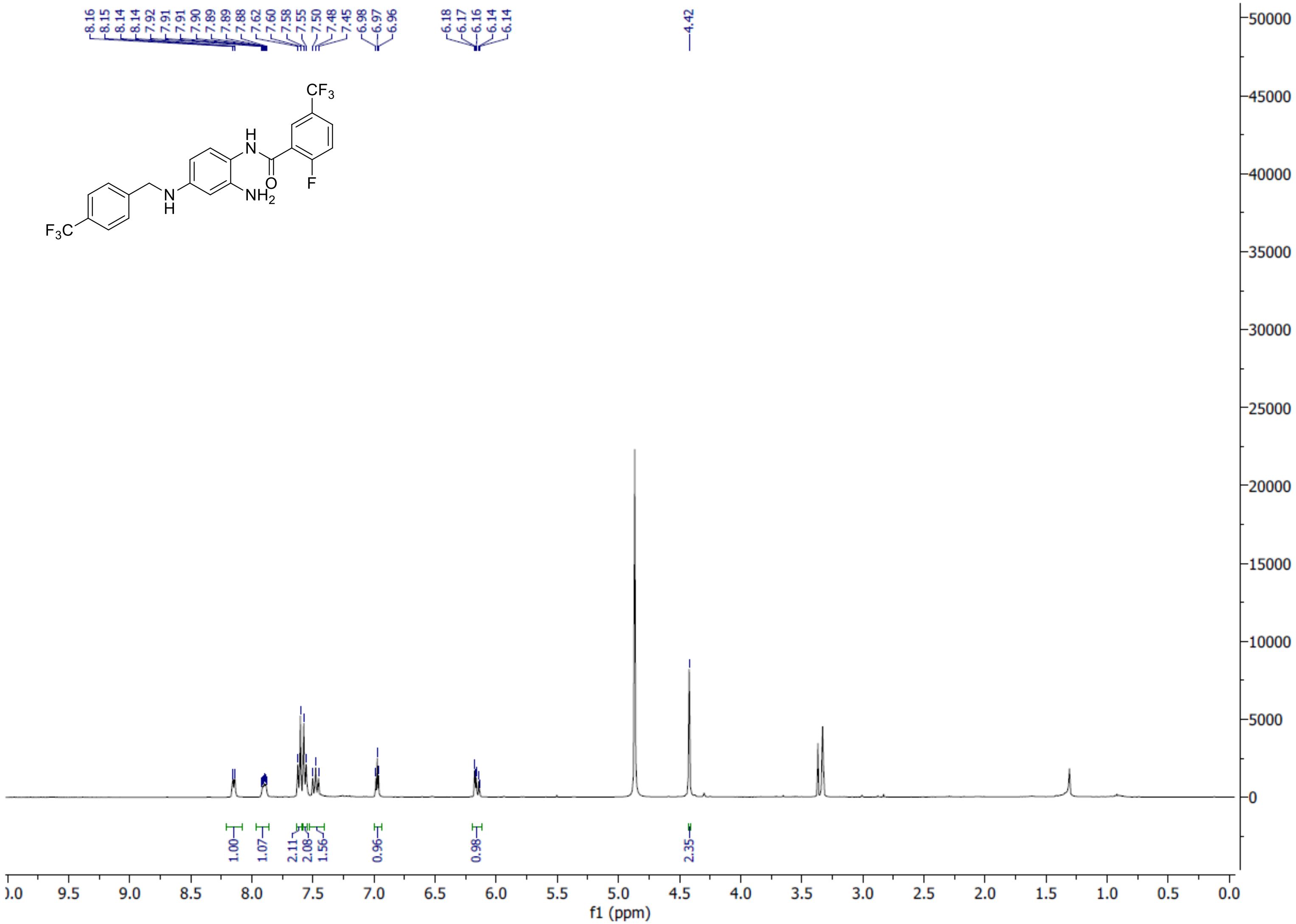

Figure S53: Compound **c114** ^{1}H-NMR spectrum

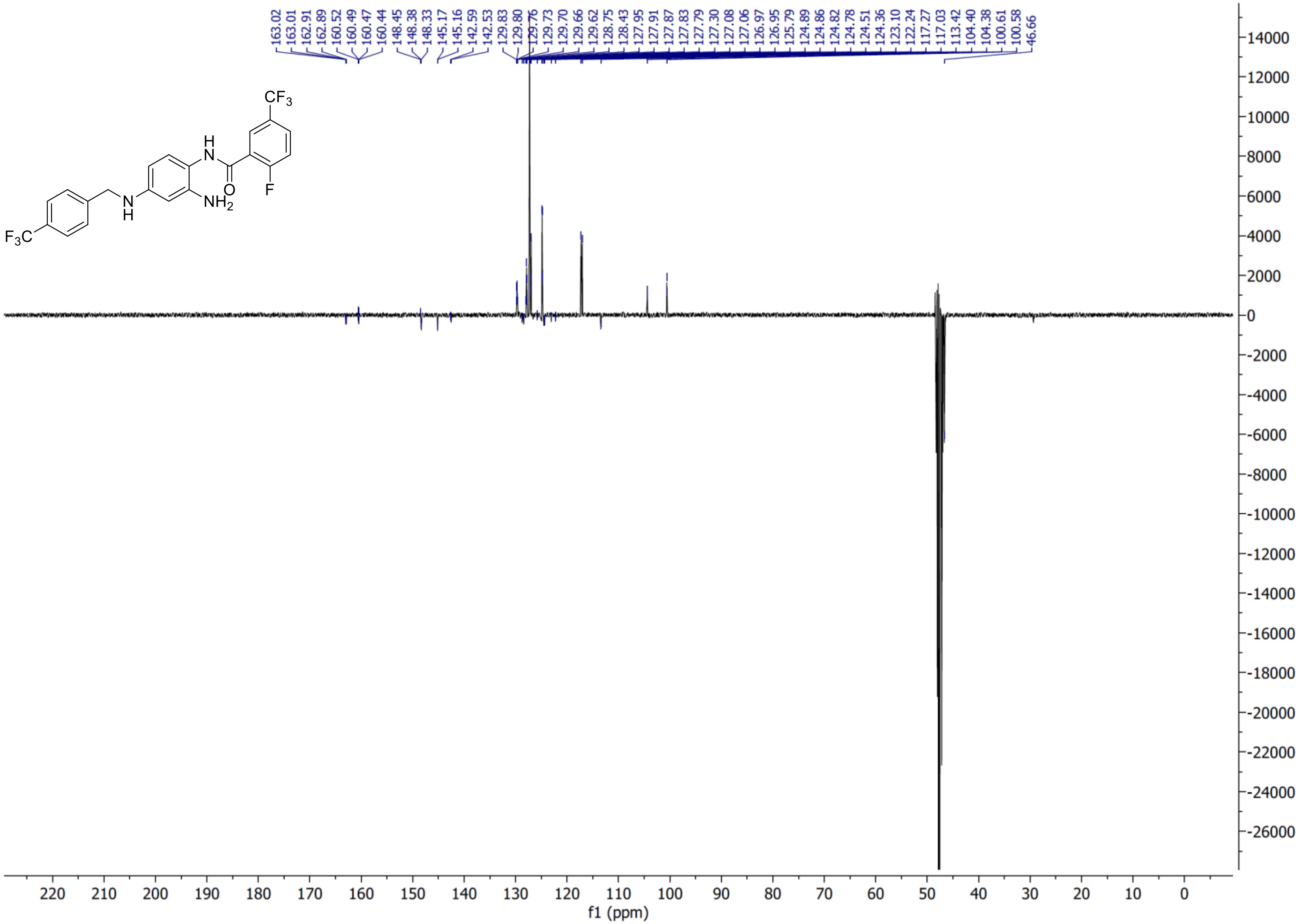

Figure S54: Compound **c114 q**DEPT NMR spectrum

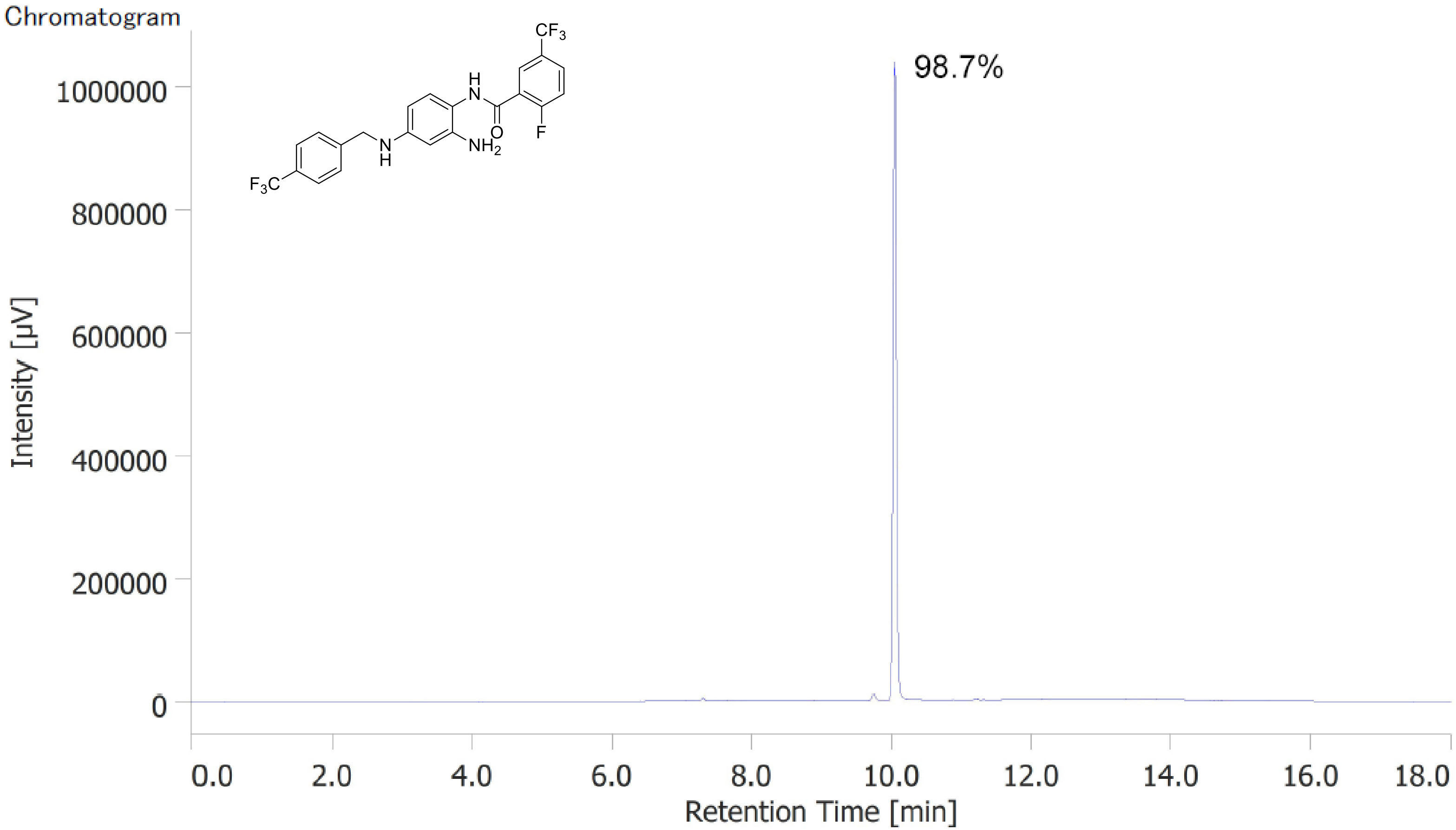

Figure S55: Compound **c114** HPLC trace

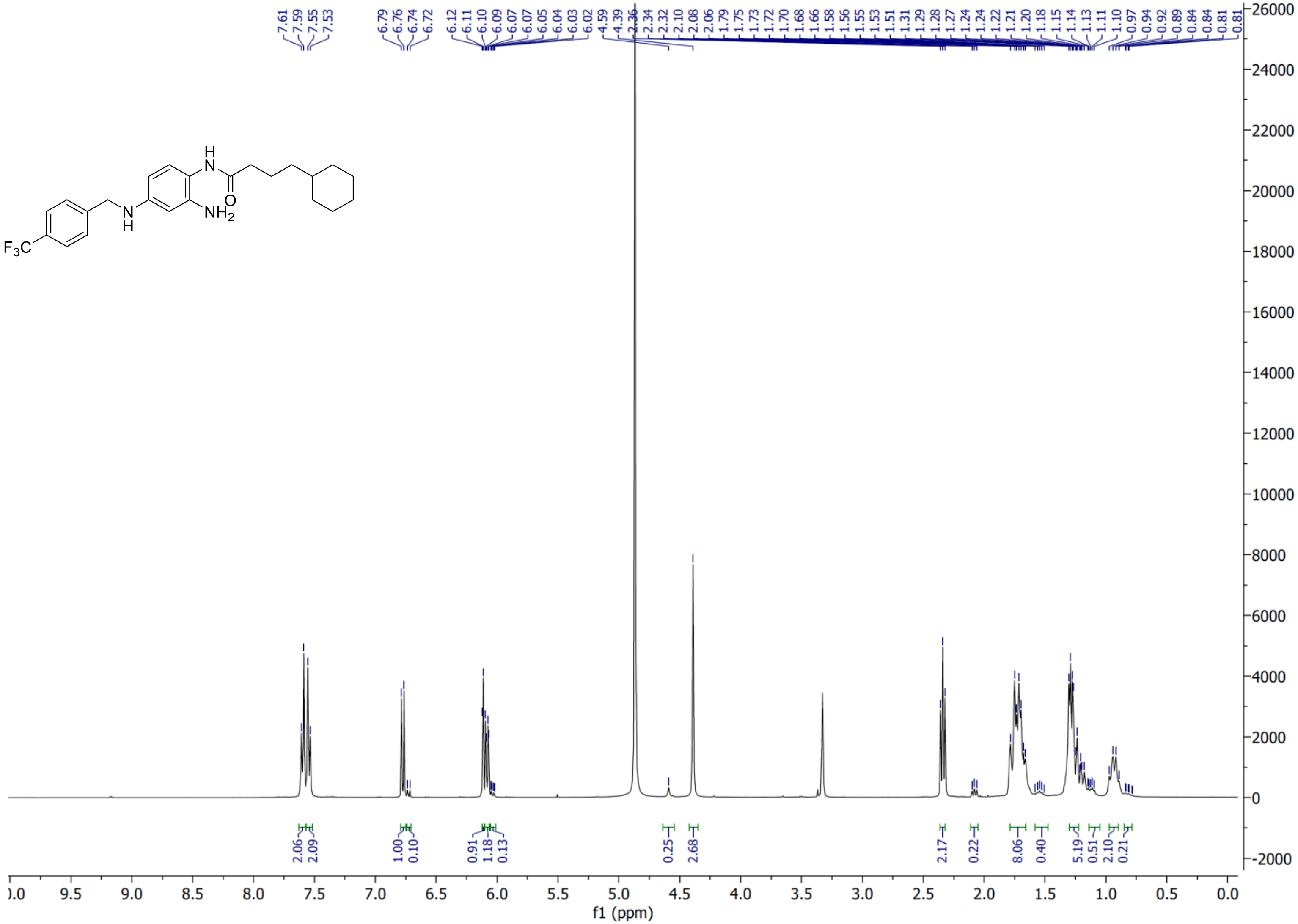

Figure S56: Compound **c115** ^{1}H-NMR spectrum

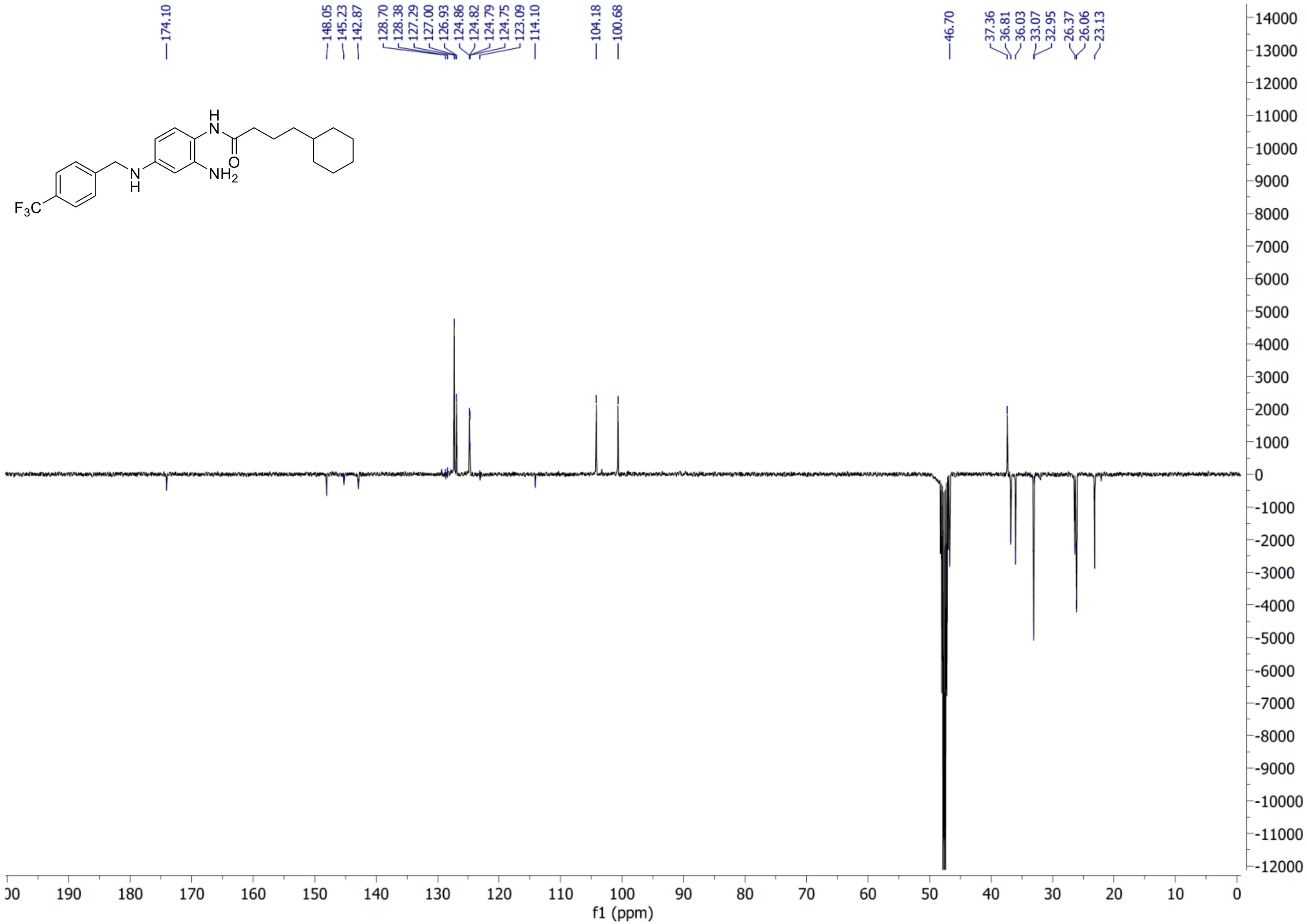

Figure S57: Compound **c115** qDEPT spectrum

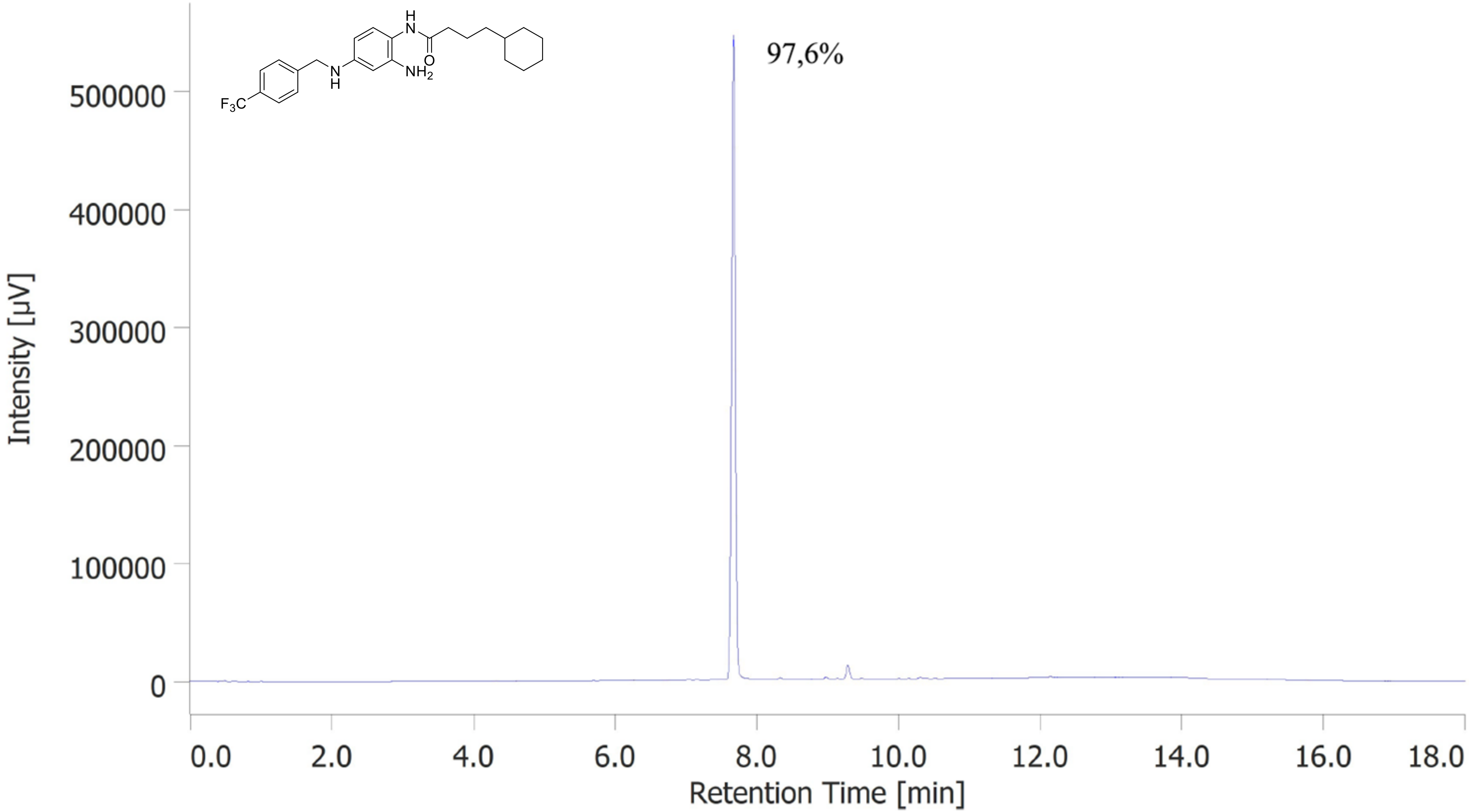

Figure S58: Compound **c115** HPLC trace

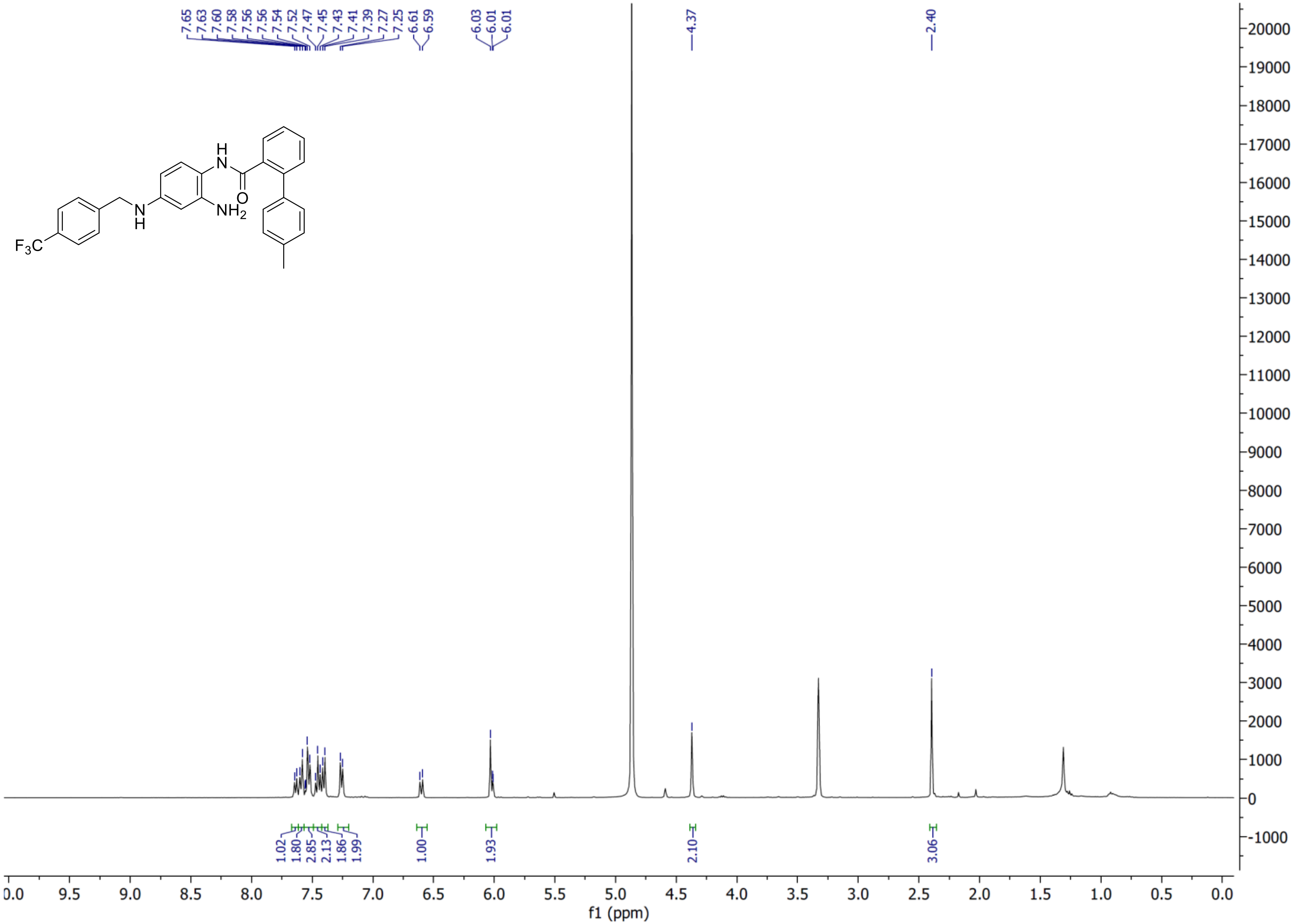

Figure S59: Compound **c116** ¹H-NMR spectrum

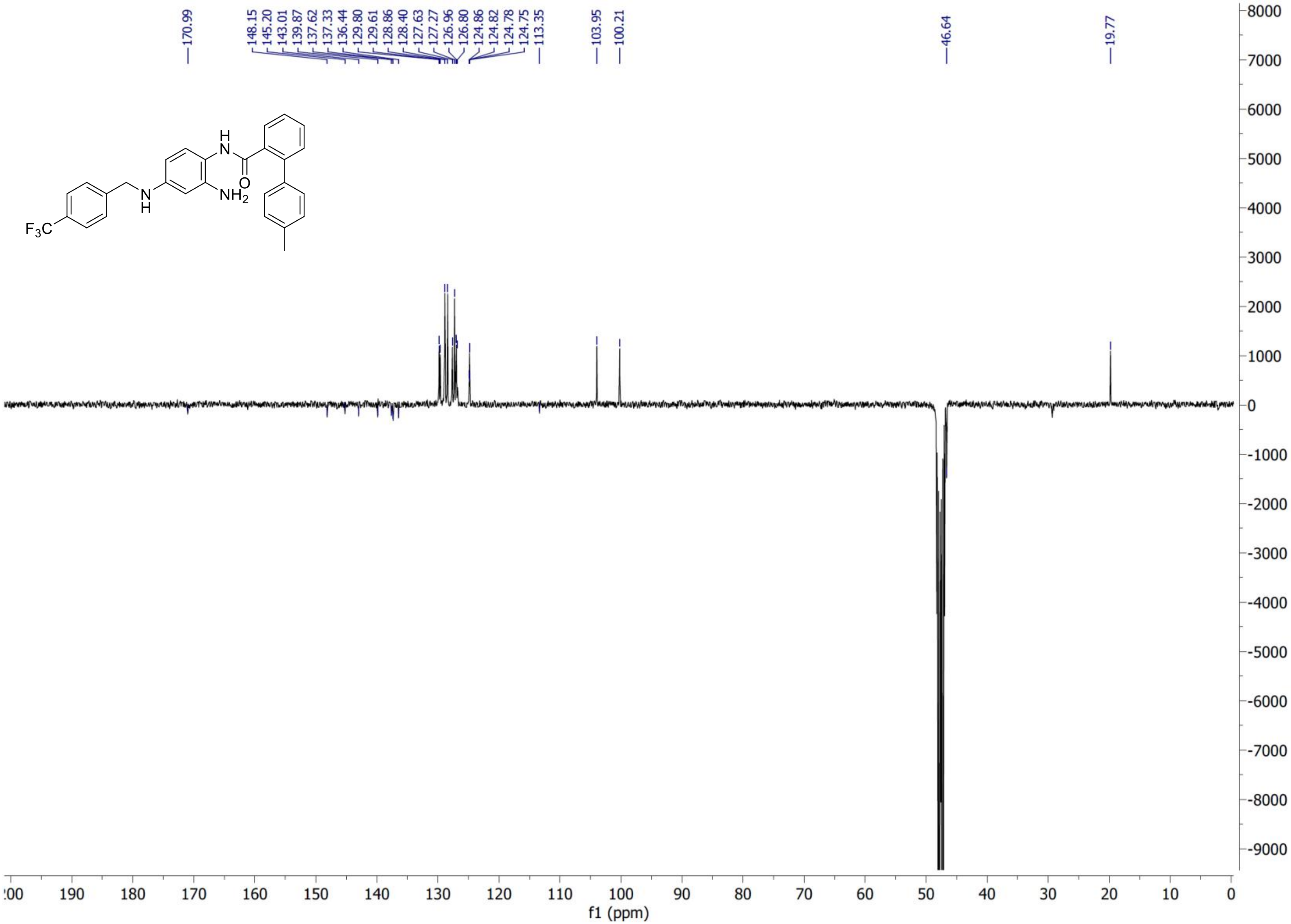

Figure S60: Compound **c116 q**DEPT NMR spectrum

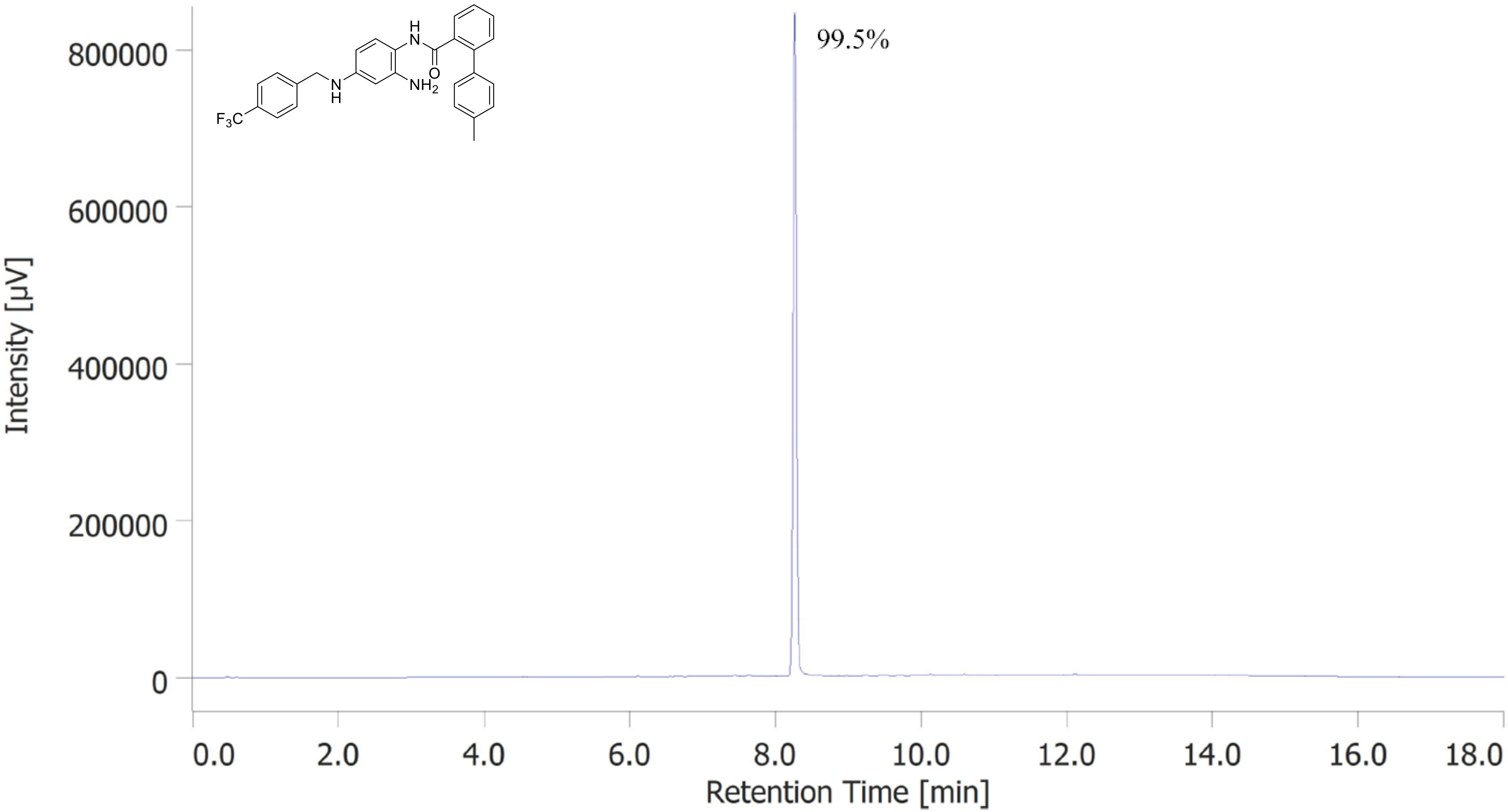

Figure S61: Compound **c116** HPLC trace

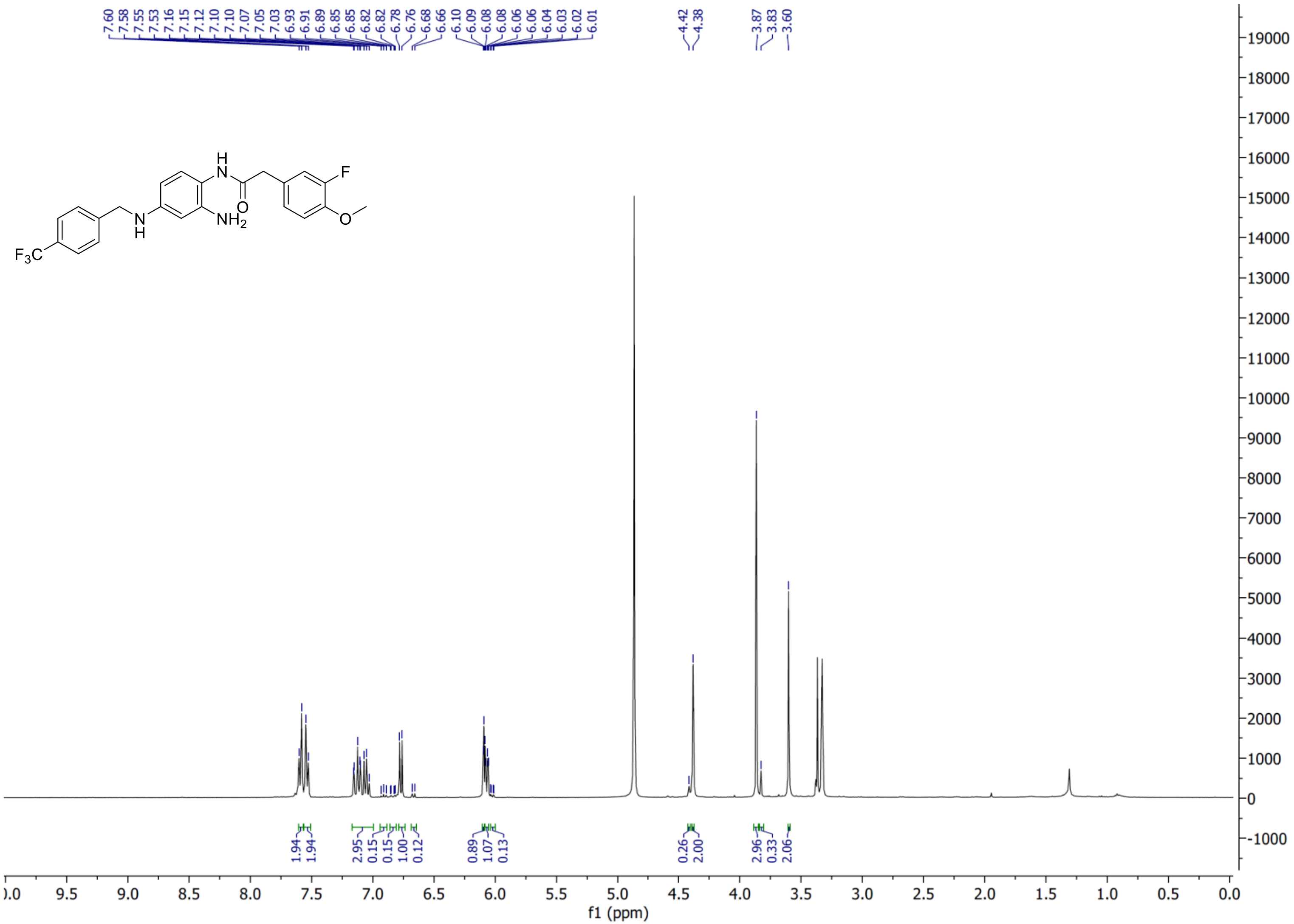

Figure S62: Compound **c117** ^{1}H-NMR spectrum

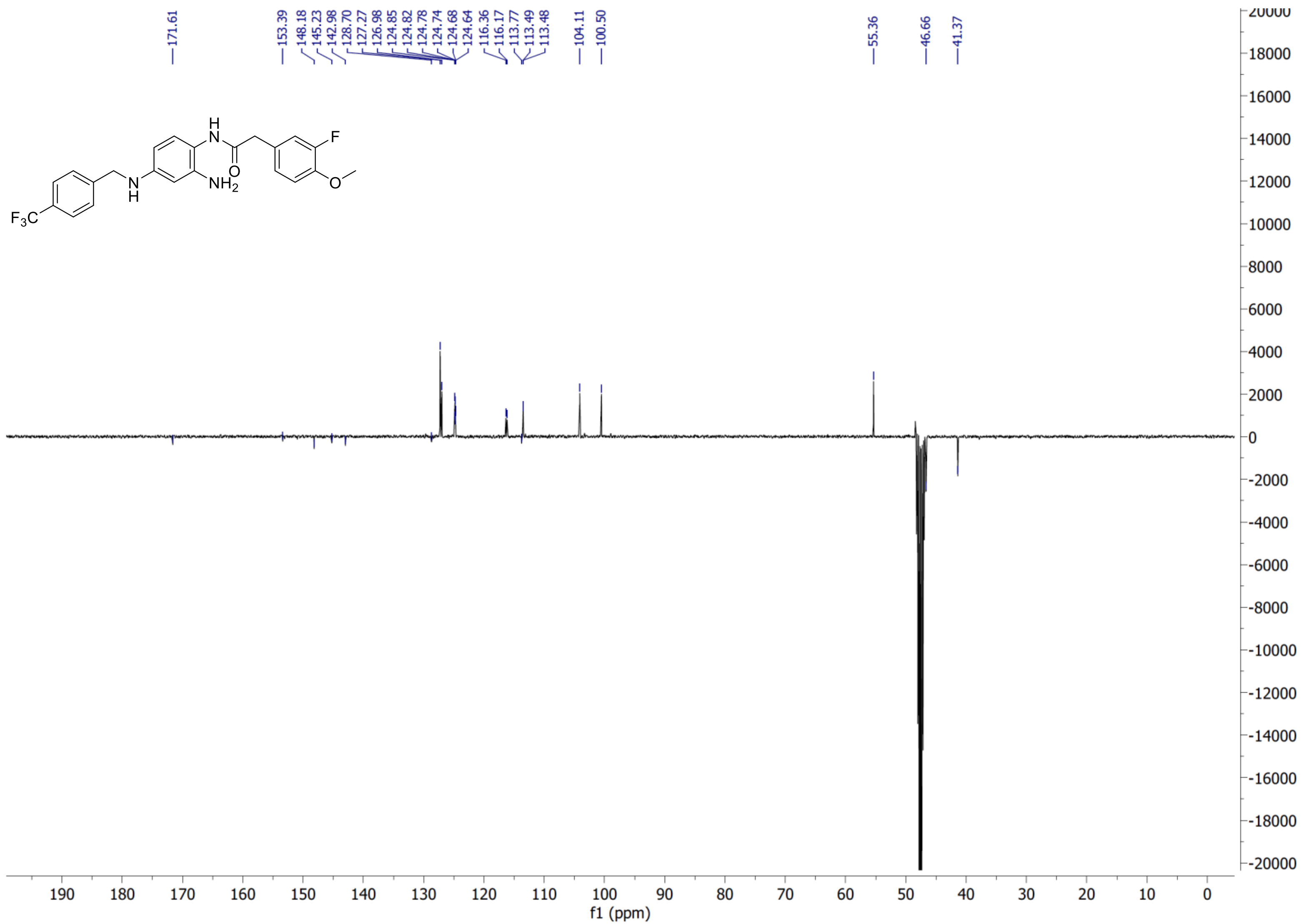

Figure S63: Compound **c117 q**DEPT NMR spectrum

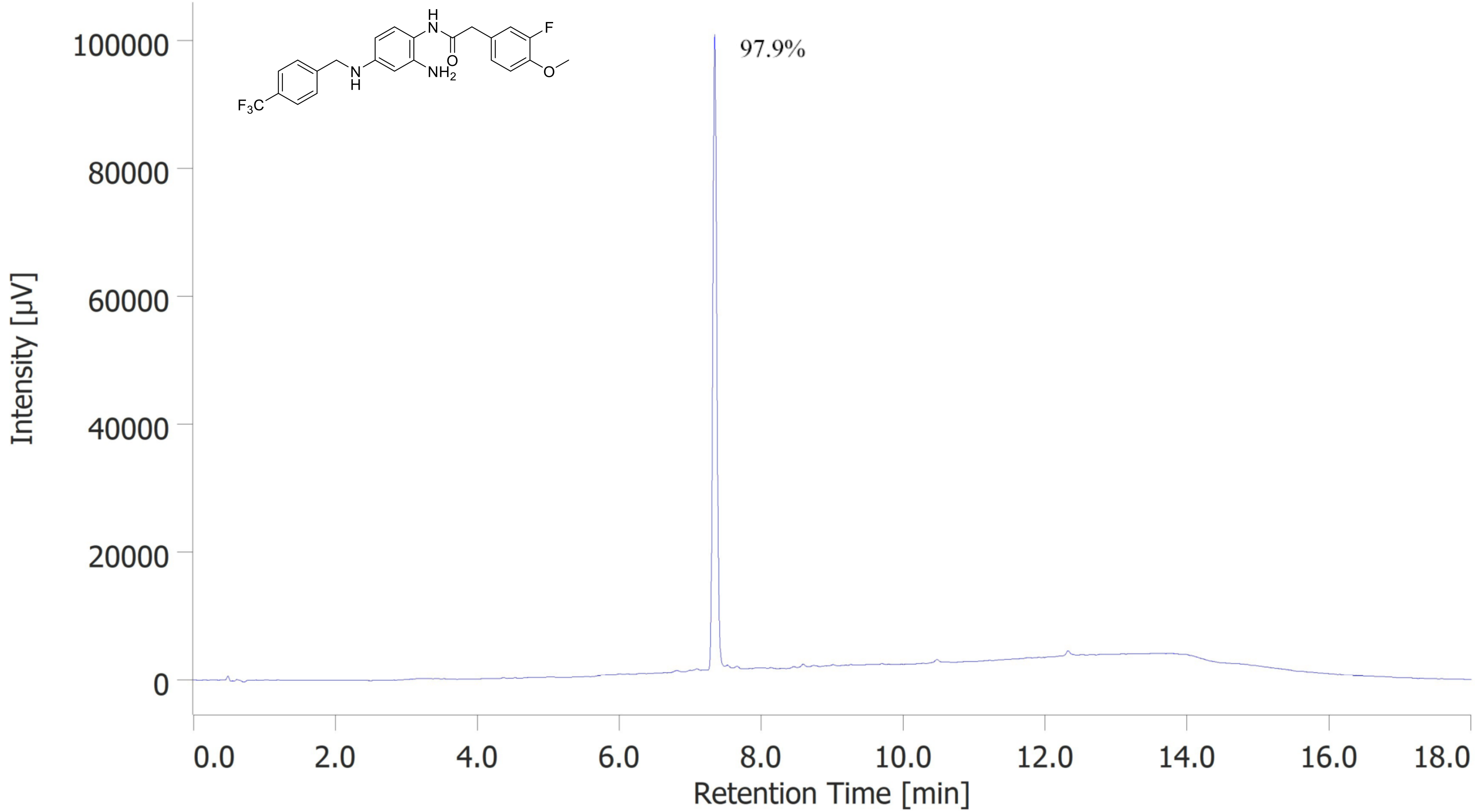

Figure S64: Compound **c117** HPLC trace

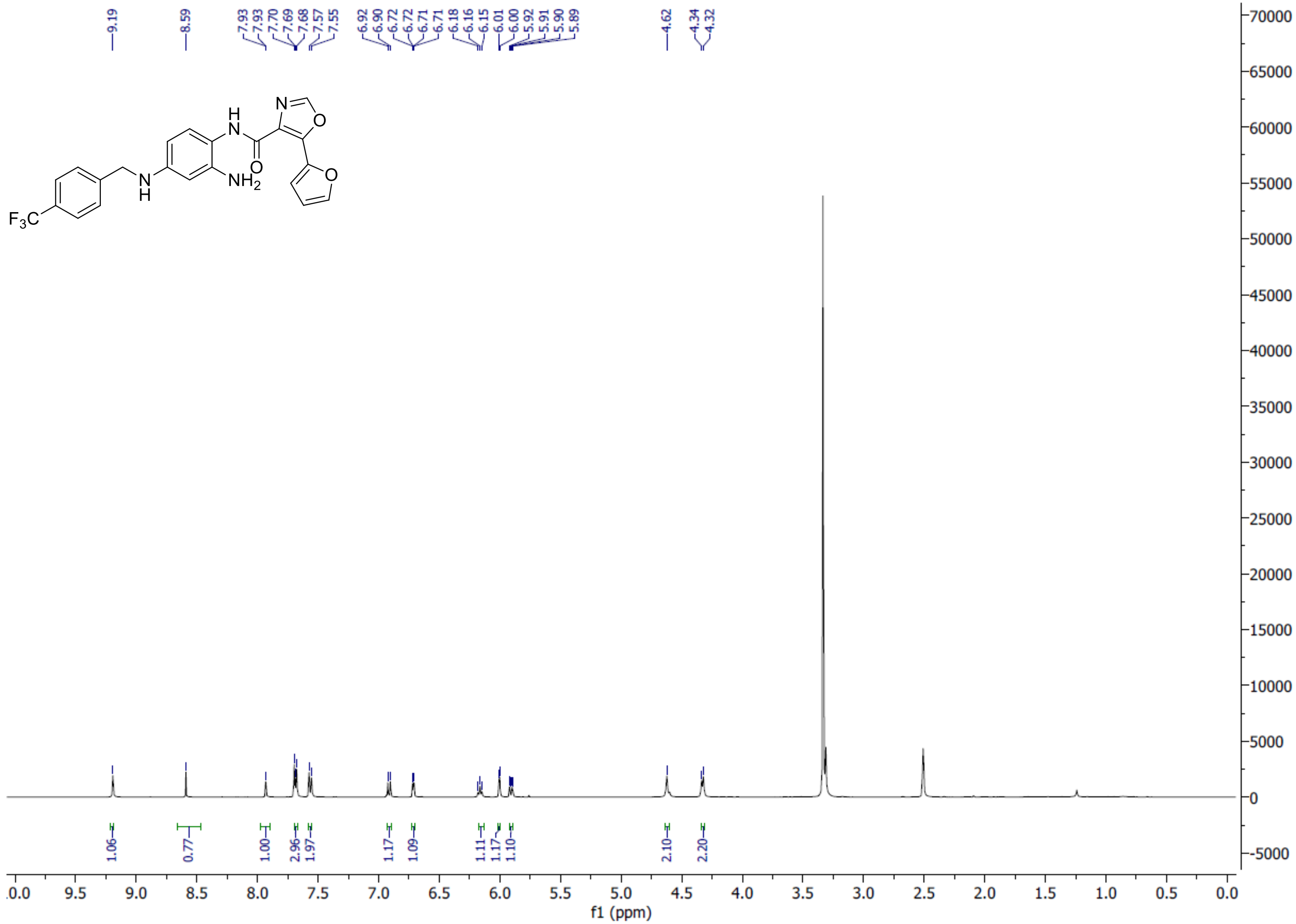

Figure S65: Compound **c118** ^{1}H-NMR spectrum

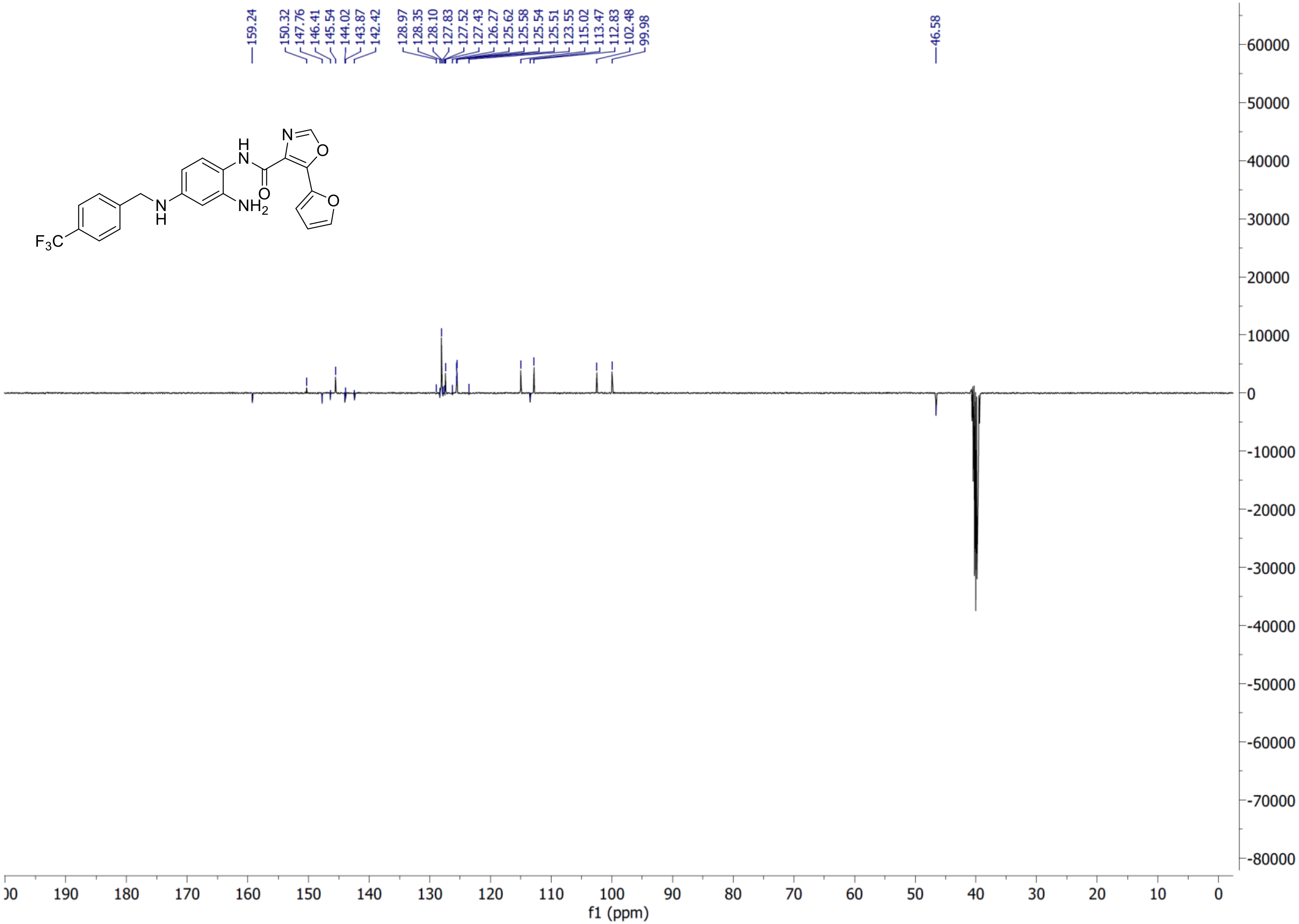

Figure S66: Compound **c118 q**DEPT NMR spectrum

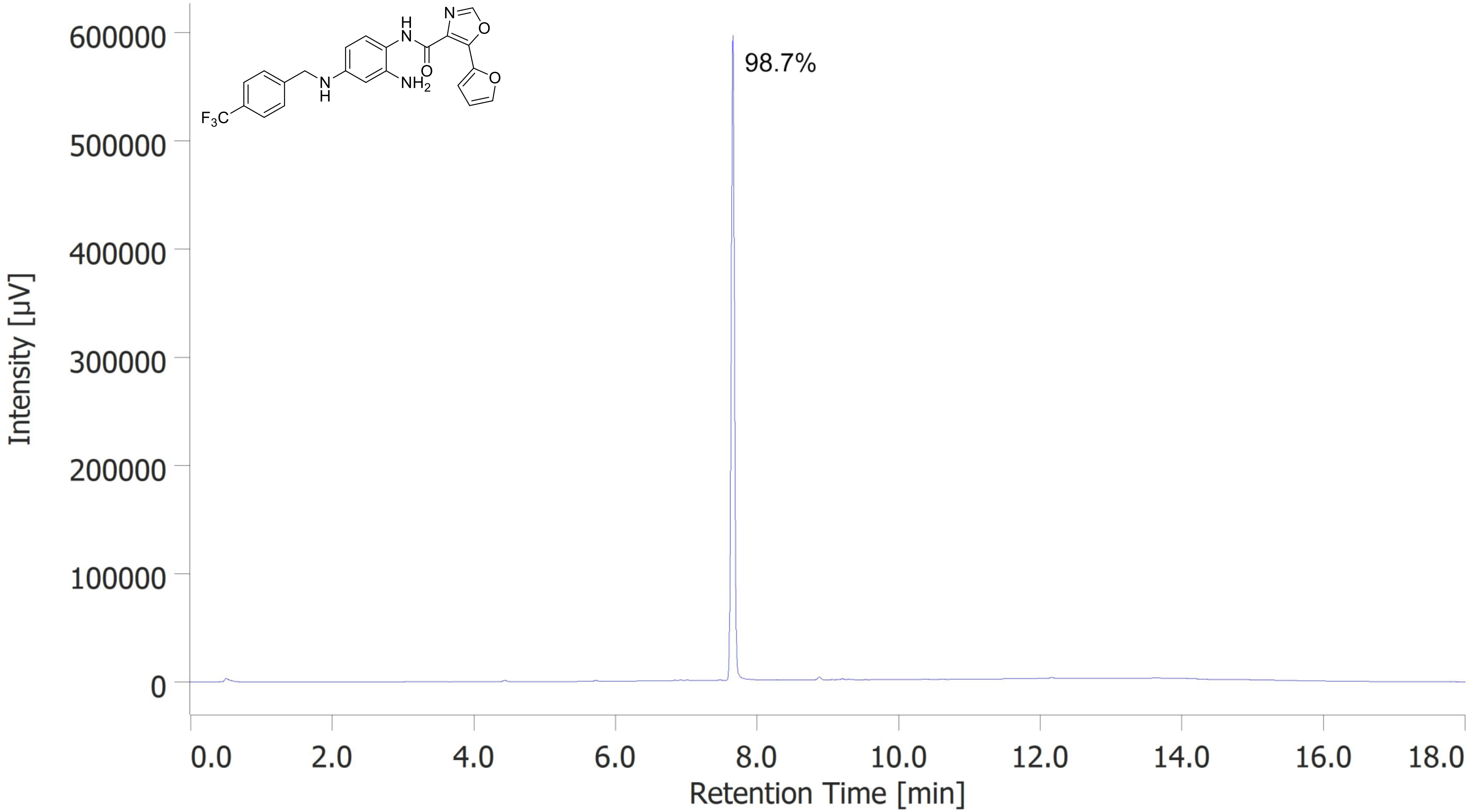

Figure S67: Compound **c118** HPLC trace

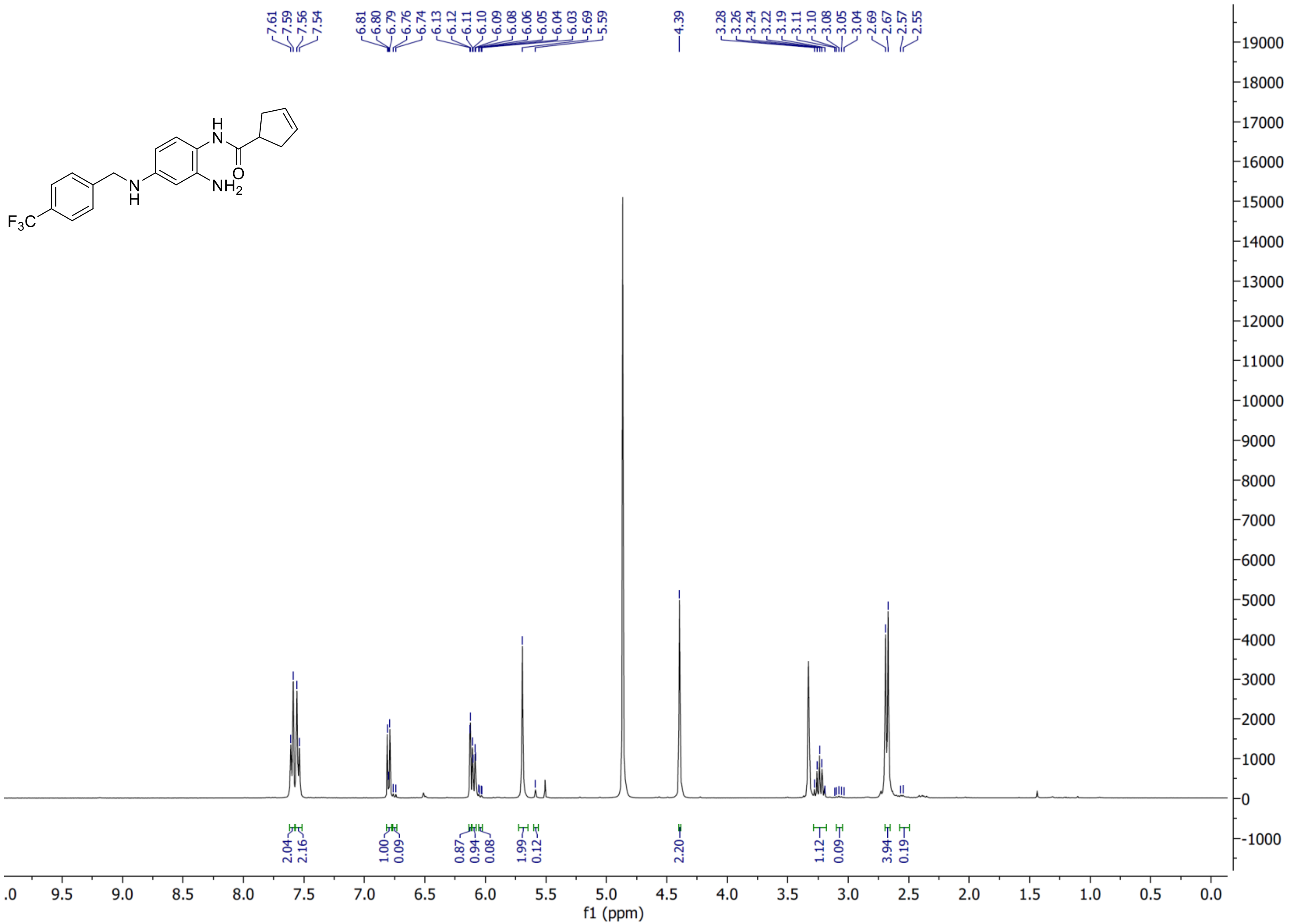

Figure S68: Compound **c119** ^{1}H-NMR spectrum

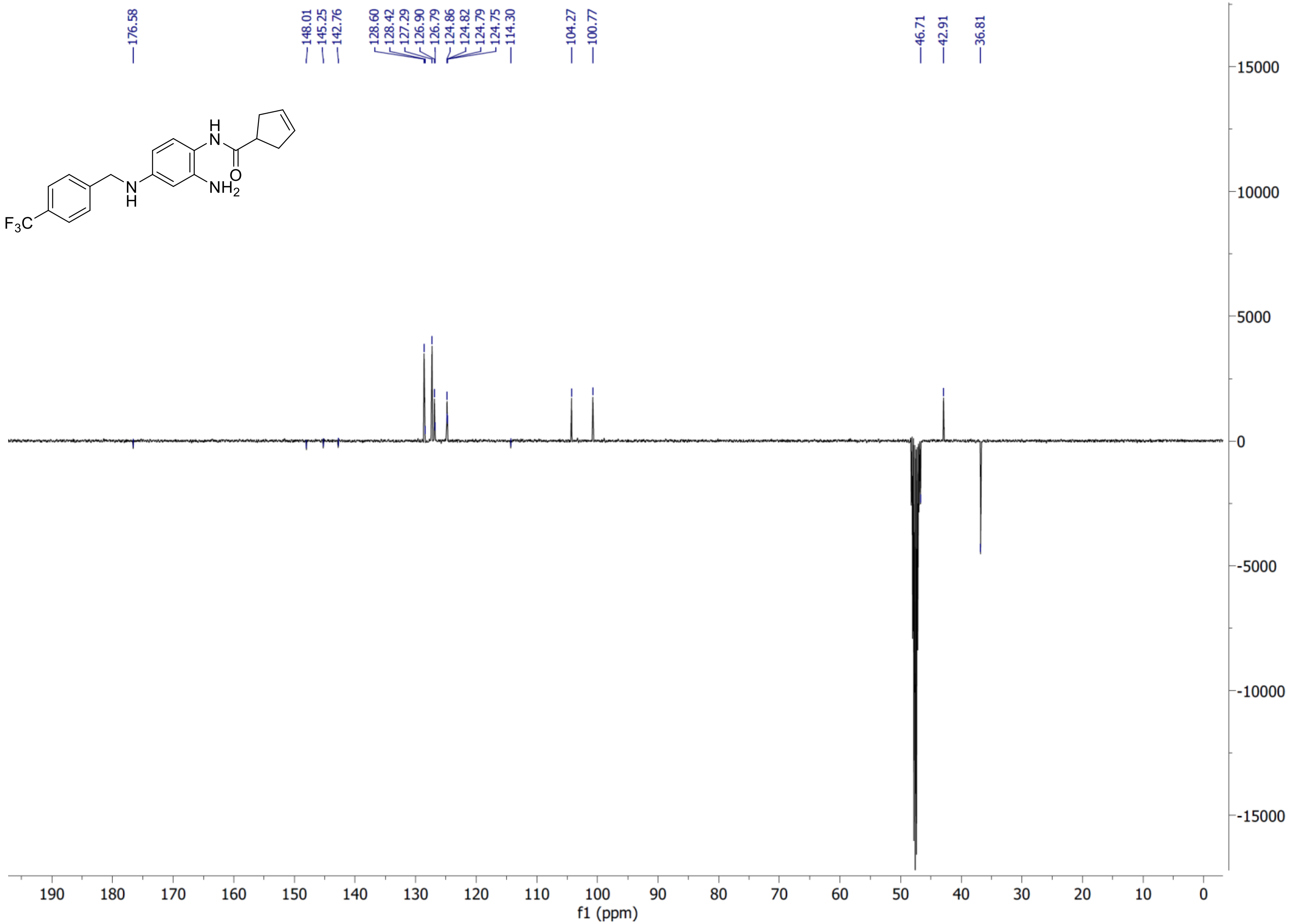

Figure S69: Compound **c119 q**DEPT NMR spectrum

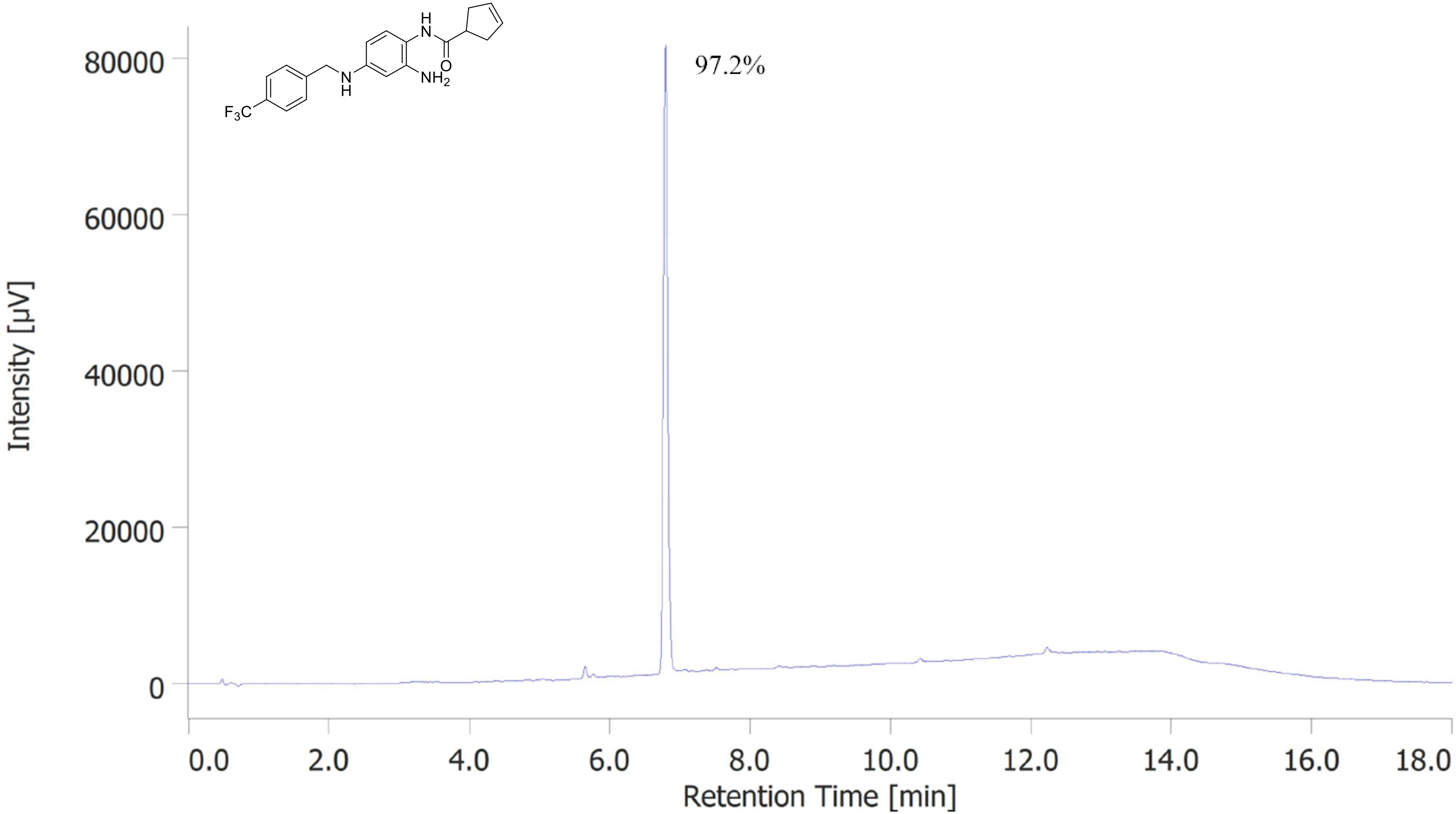

Figure S70: Compound **c119** HPLC trace

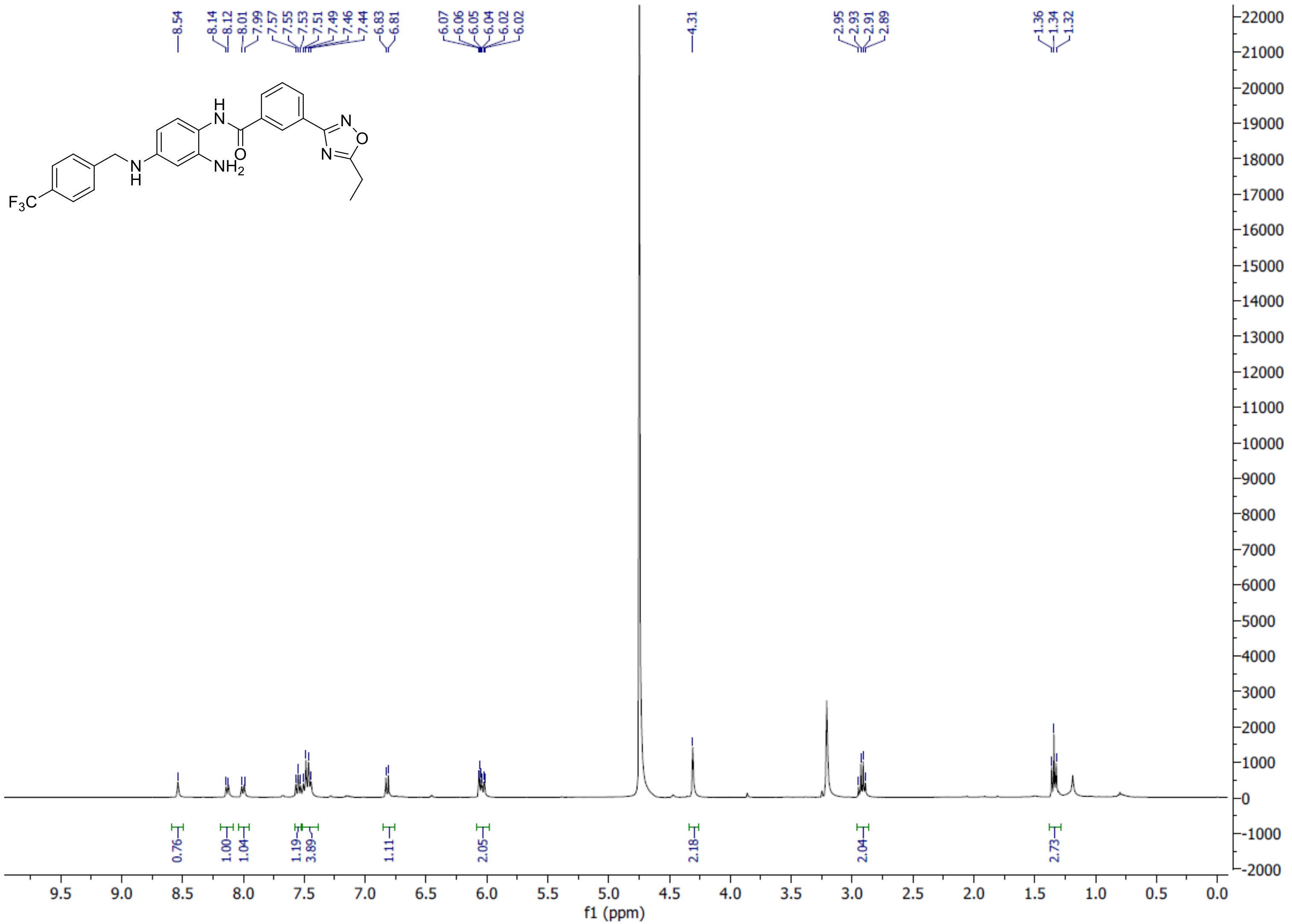

Figure S71: Compound **c120** ^{1}H-NMR spectrum

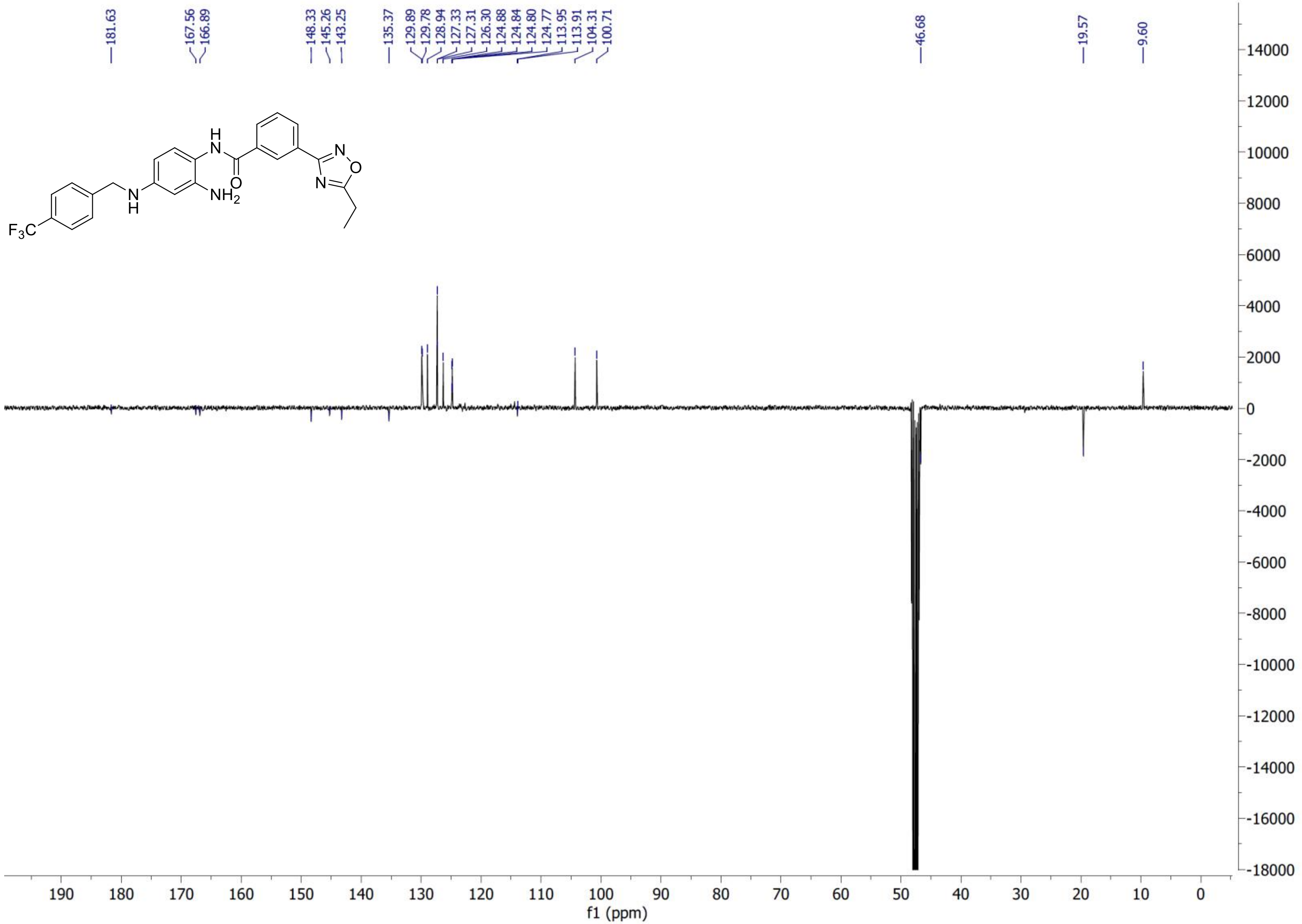

Figure S72: Compound **c120 q**DEPT NMR spectrum

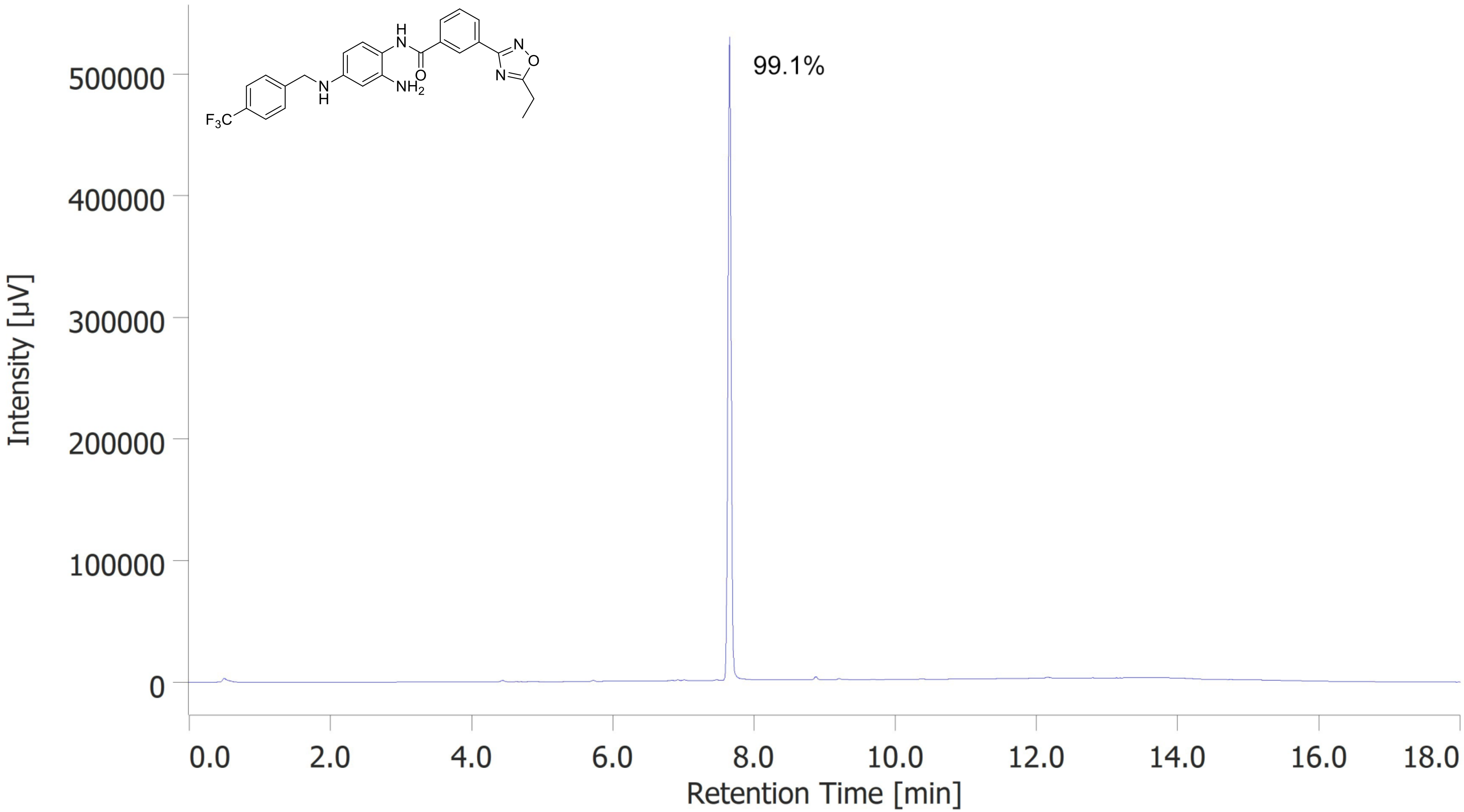

Figure S73: Compound **c120** HPLC trace

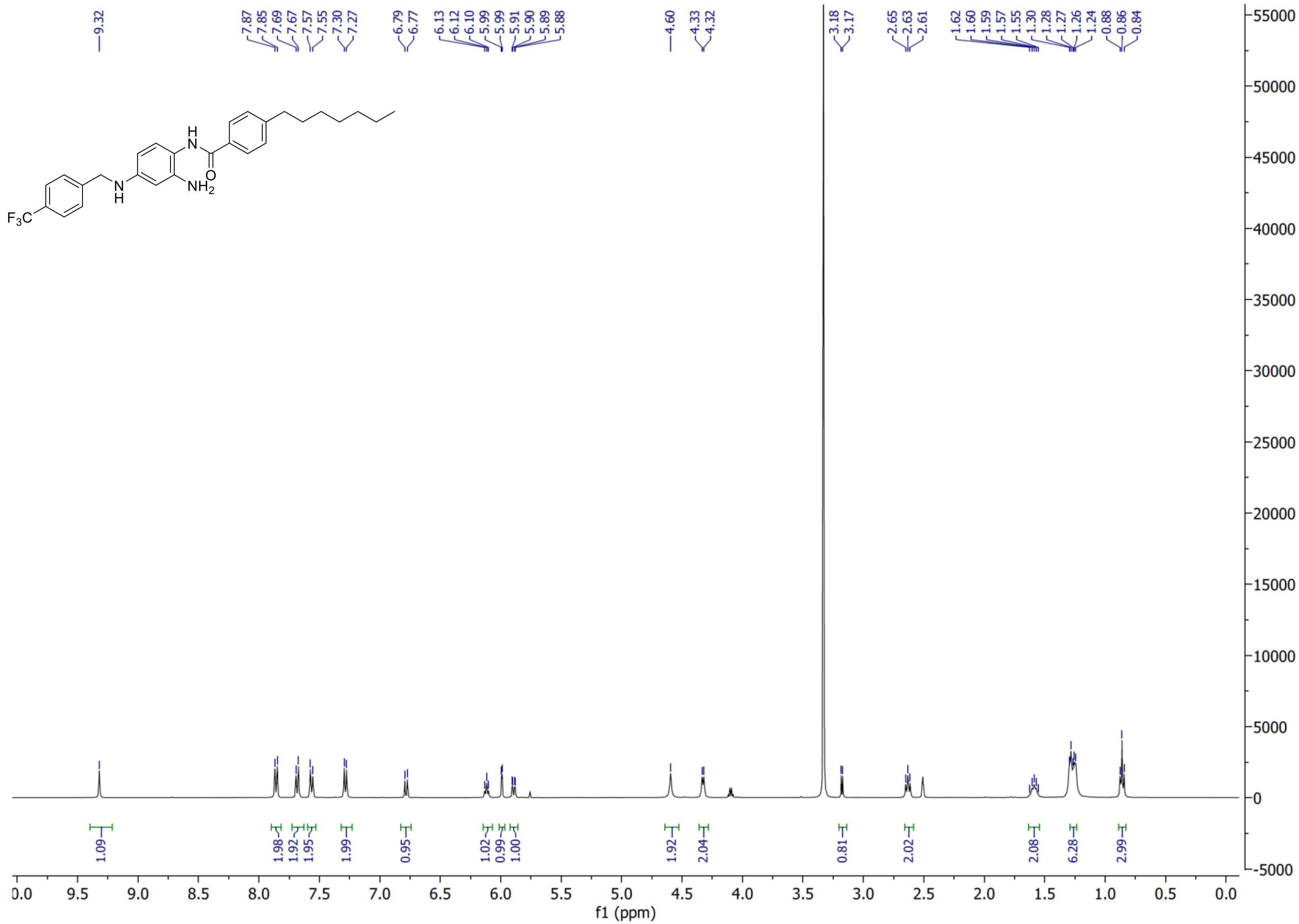

Figure S74: Compound **c121** ^{1}H-NMR spectrum

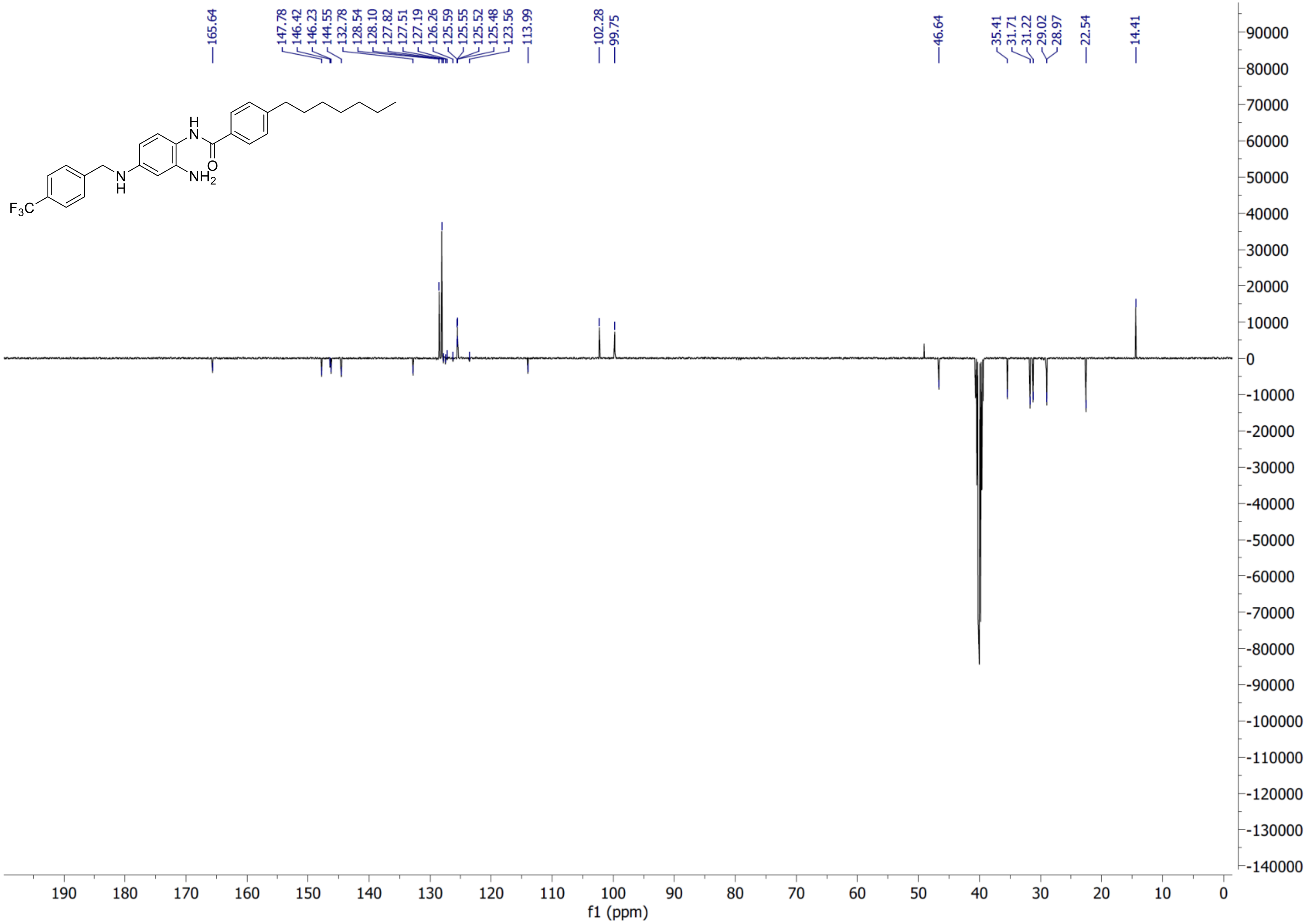

Figure S75: Compound **c121 q**DEPT NMR spectrum

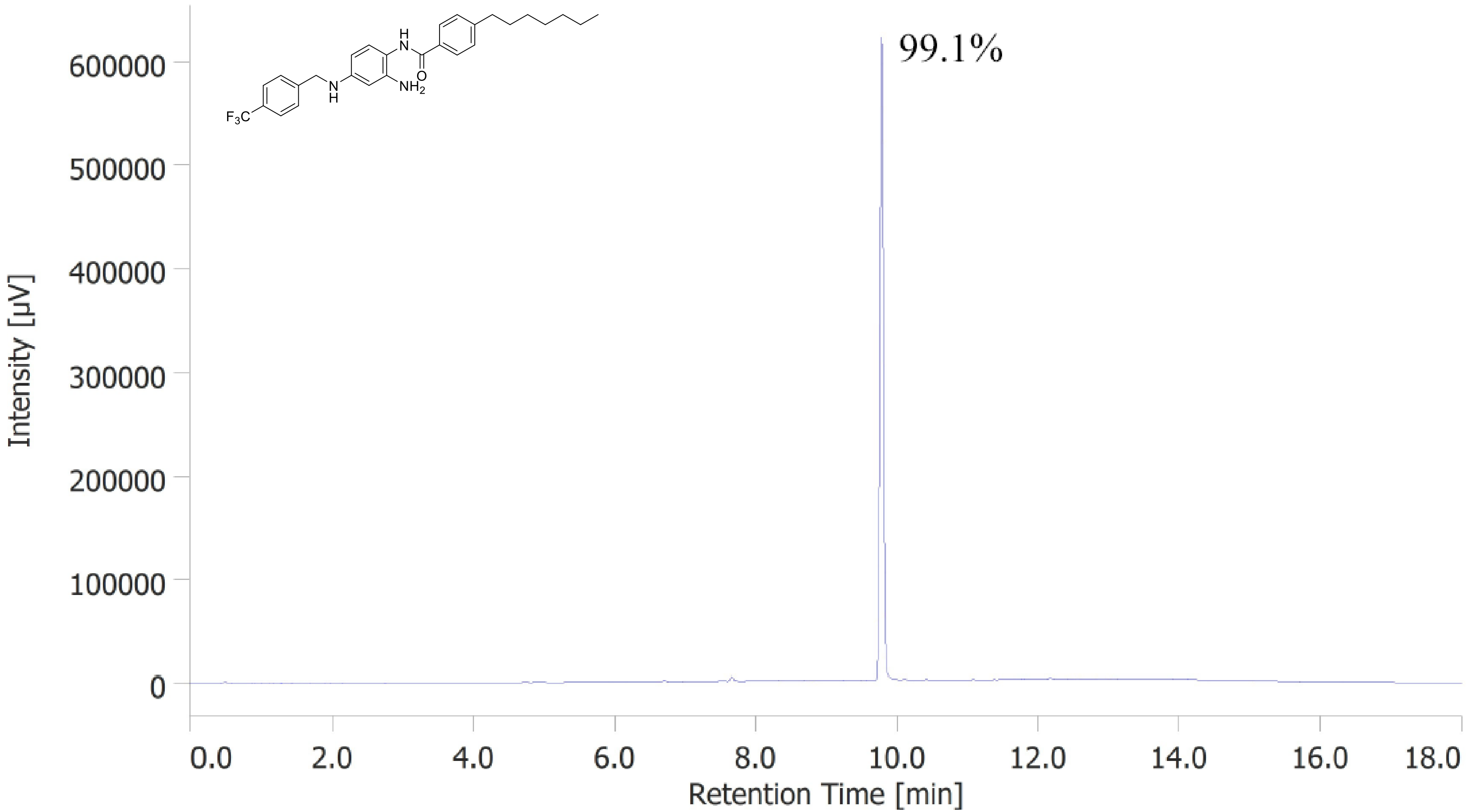

Figure S76: Compound **c121** HPLC trace

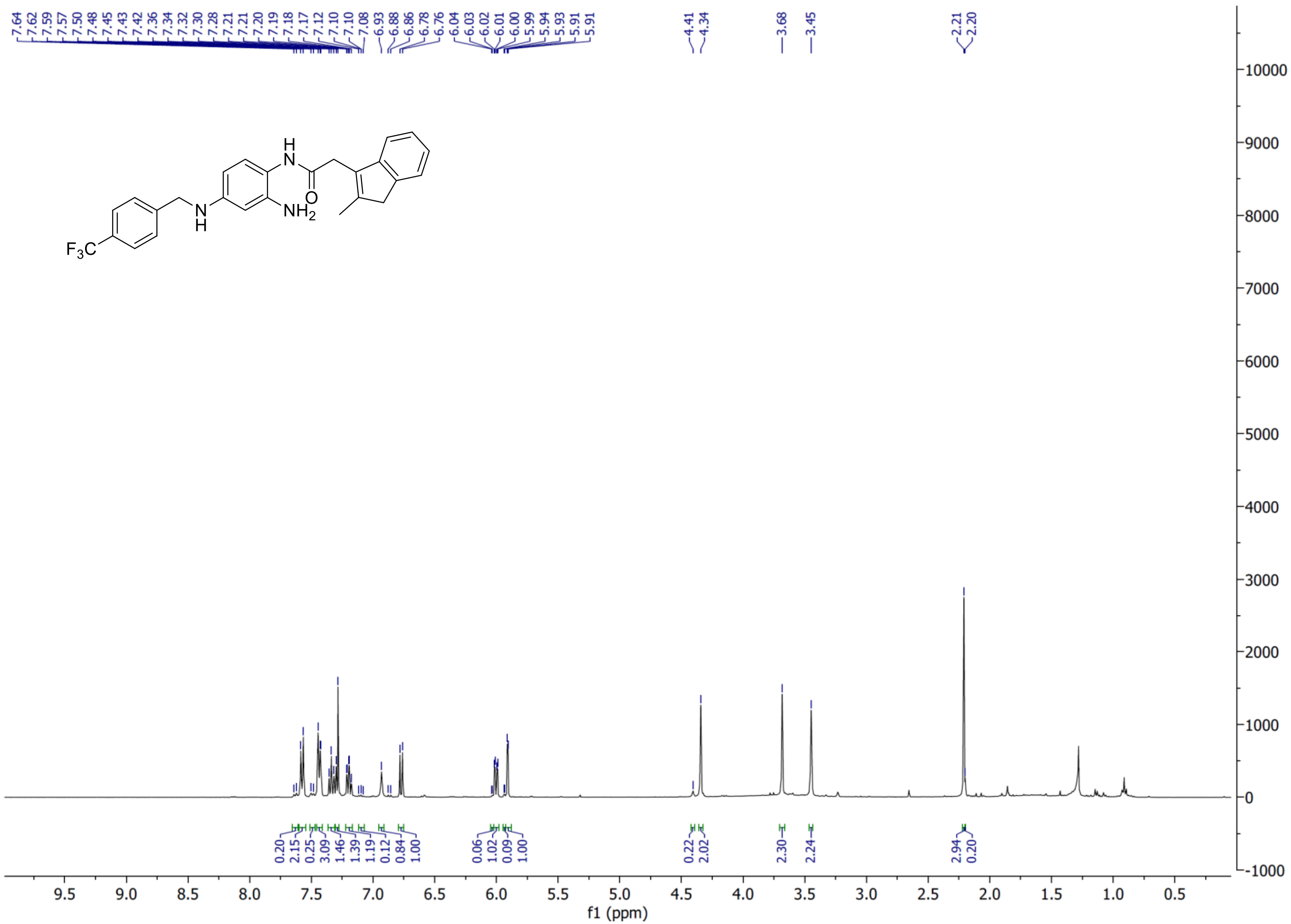

Figure S77: Compound **c122** ^{1}H-NMR spectrum

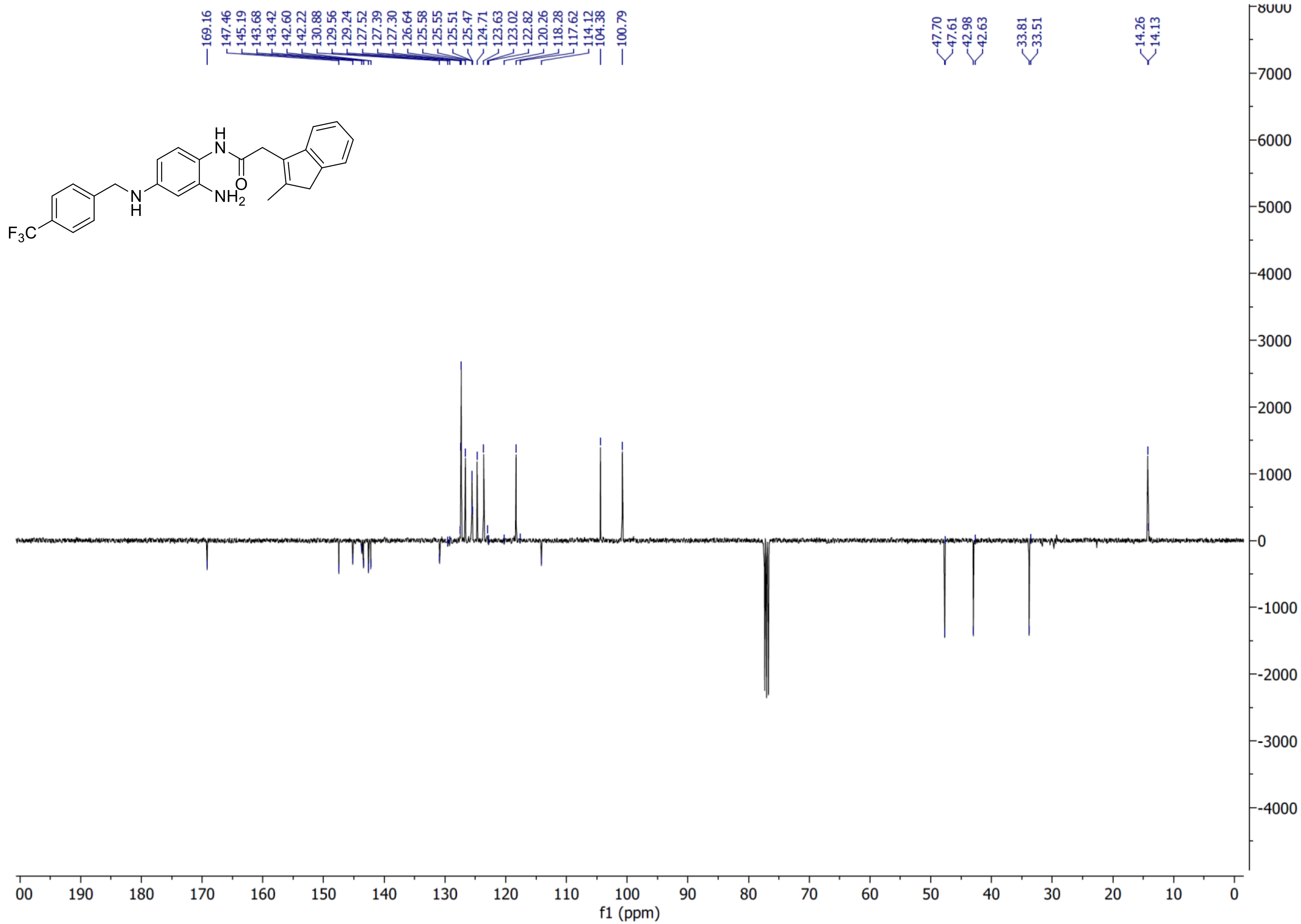

Figure S78: Compound **c122 q**DEPT NMR spectrum

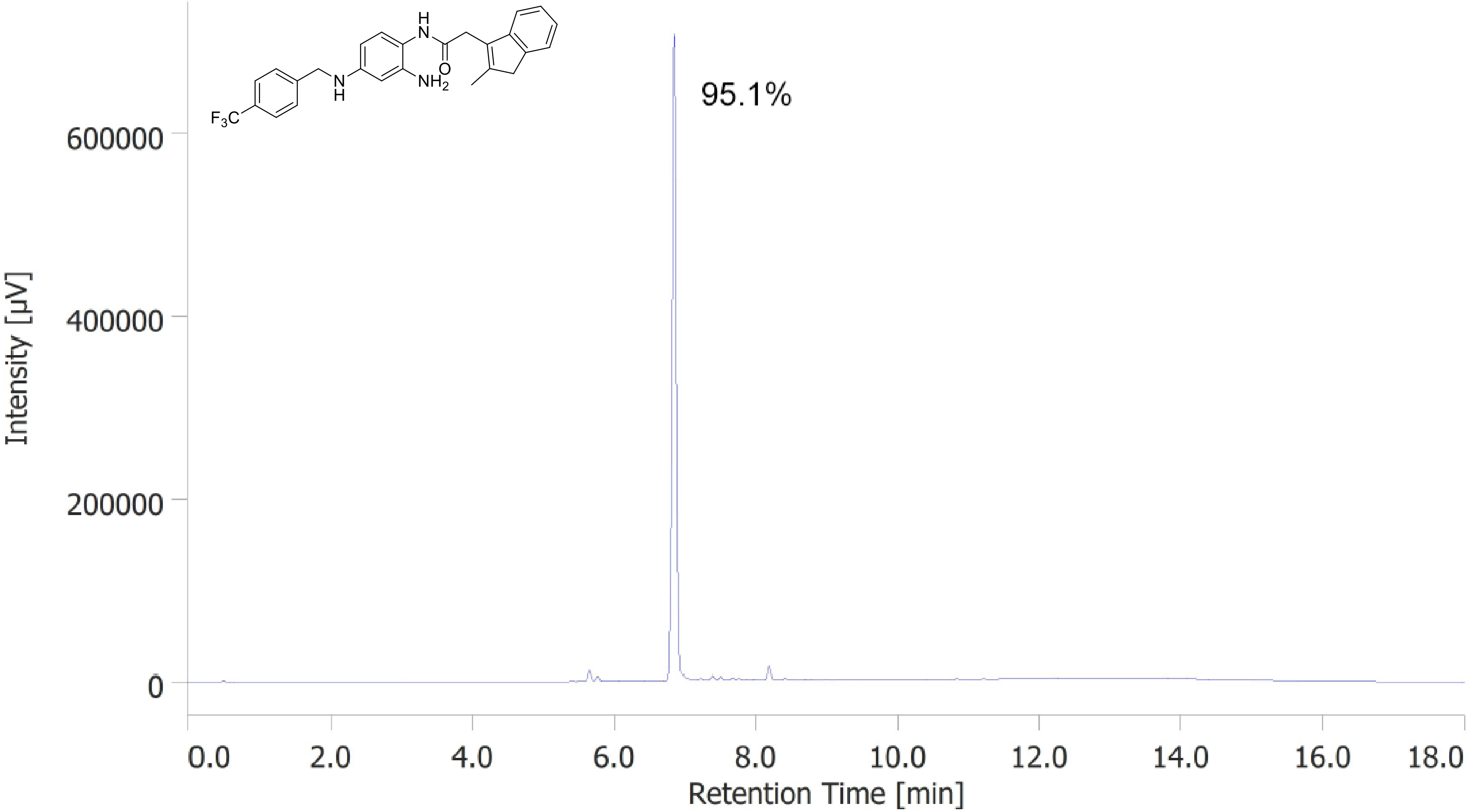

Figure S79: Compound **c122** HPLC trace

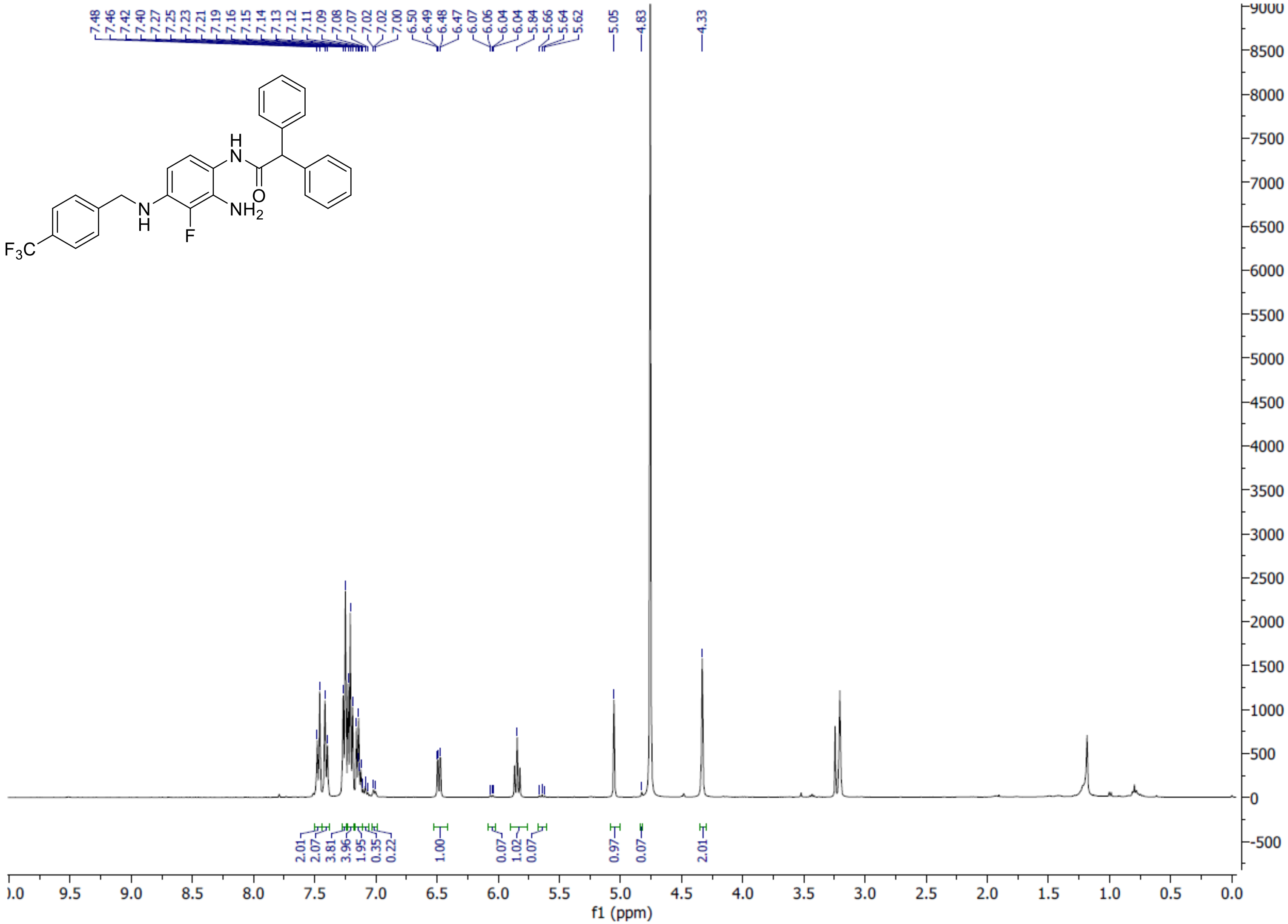

Figure S80: Compound **c106F** ^{1}H-NMR spectrum

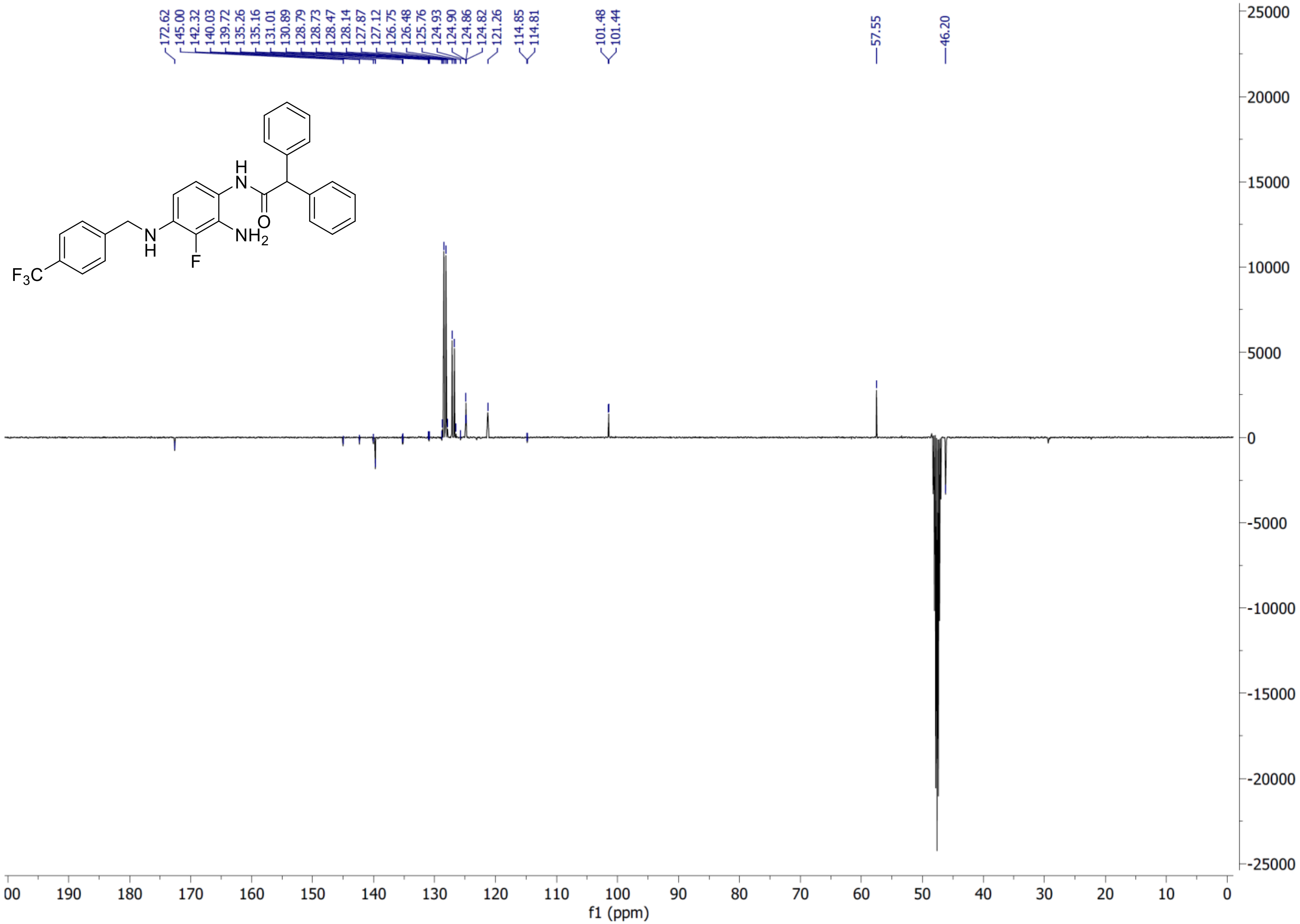

Figure S81: Compound **c106F q**DEPT NMR spectrum

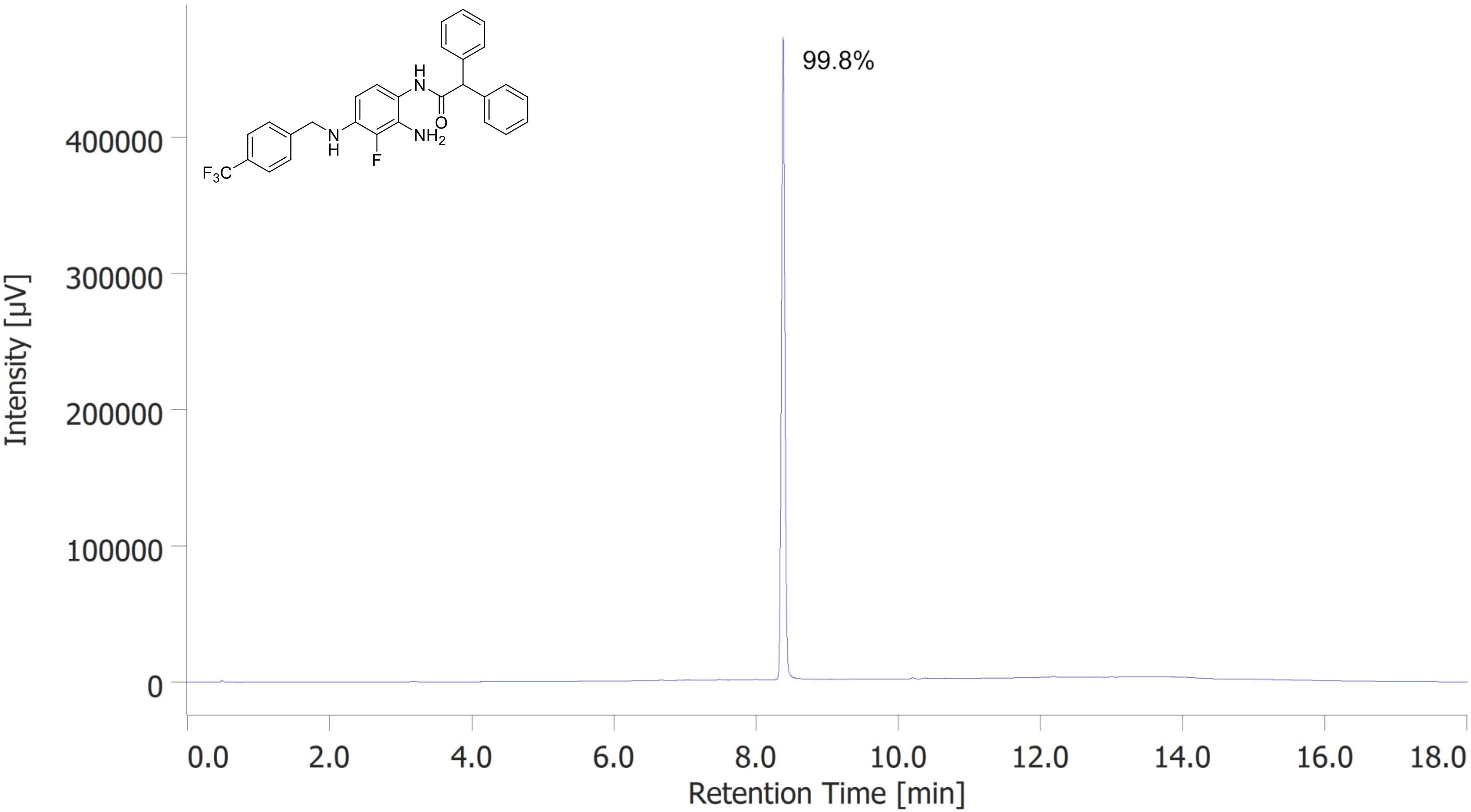

Figure S82: Compound **c106F** HPLC trace

2. AUTOMATED PATCH-CLAMP EXPERIMENTS

2.1 General.

Automated patch-clamp experiments were performed via a high throughput 384-well automated electrophysiology platform (Qube®) using a commercially-available provider (Ion Channel Profiler hKv7.2/hKv7.3 Agonist Qube Assay at Eurofins; https://emea.eurofinsdiscovery.com/catalog/hkv7-2-7-3-potassium-channel-agonist-qube-apc-1-conc/CYL8059QB; Study code: US034-0018912). In this assay, CHO cells stably expressing Kv7.2/3 subunits were held at -120 mV and depolarized at -40 mV for 1 sec, followed by a return to -120mV. Each compound was tested at a single concentration (10 μM) for its ability to increase the peak current amplitude measured at -40 mV, a membrane potential value very close to the current activation threshold and where most channels have low open probabilities (agonist mode).

Table S1. Molecular structures of the synthesized compounds and their effects on Kv7.2/7.3 currents assessed with automated patch-clamp

Compound	Chemical structure	Peak currents @-40 mV (pA)	I_{drug}/I_{RET} (%)
**Vehicle**		-133.3±40.7	0.0±0.83
**RET**	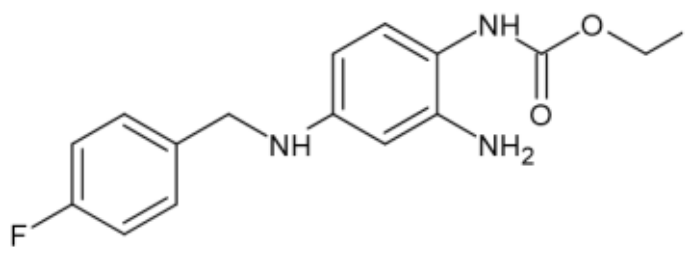	4917.53±31.32	100.0±0.64
**c60**	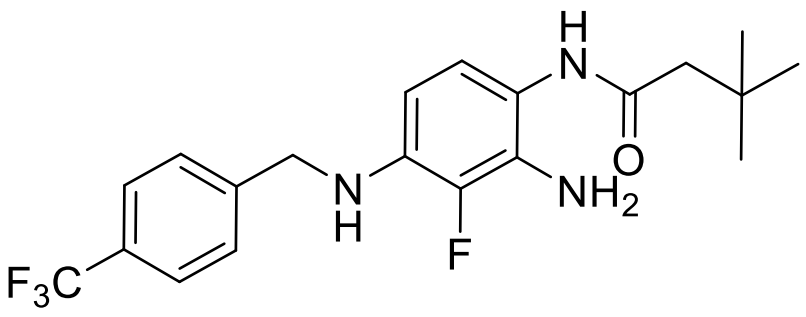	7391.80±96.83	150.32±1.97
**c97**	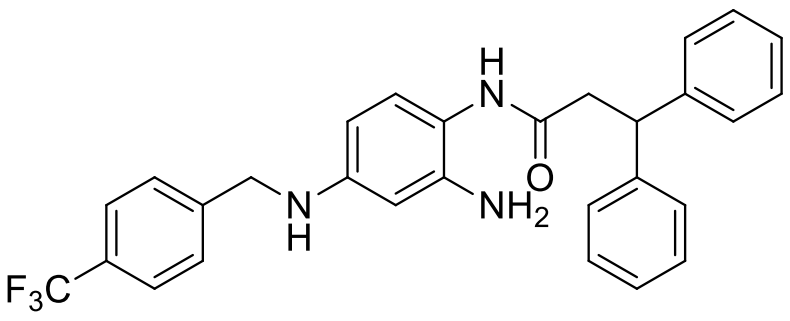	-150.56±0.48	-3.06±0.02
**c98**	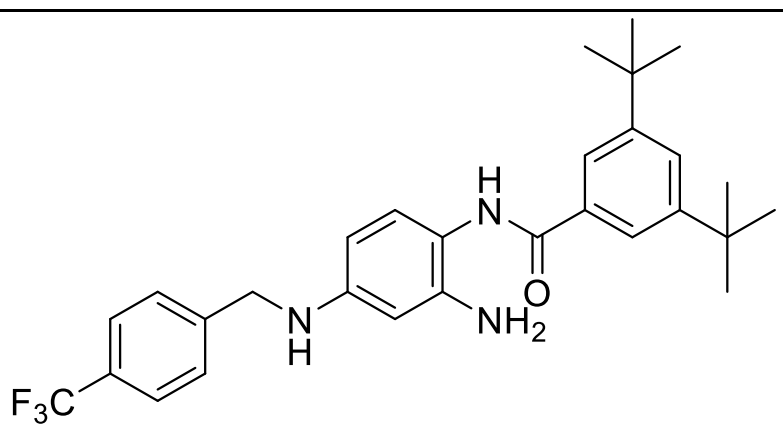	-37.18±30.72	-0.76±0.62
**c100**	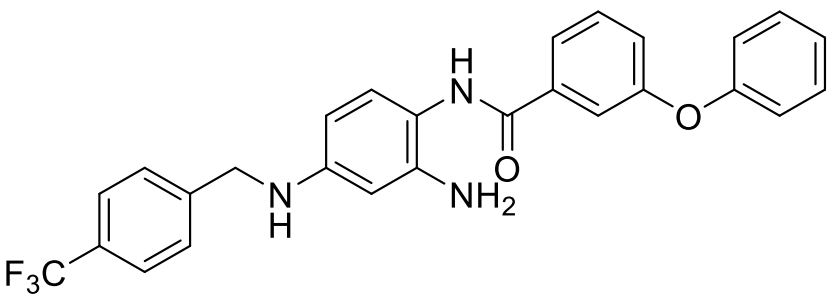	34.00±20.44	0.69±0.42
**c101**	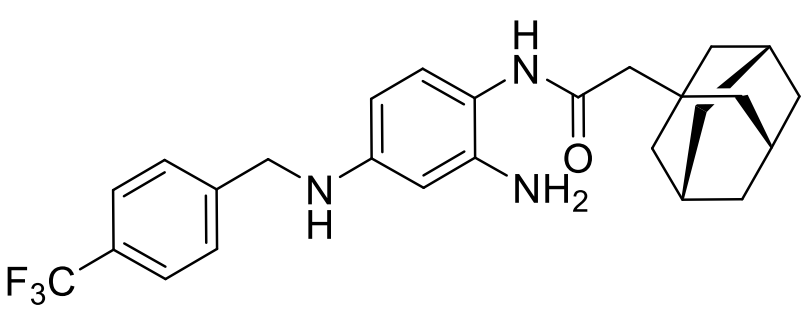	2575.28±45.14	52.40±0.94

**c102**	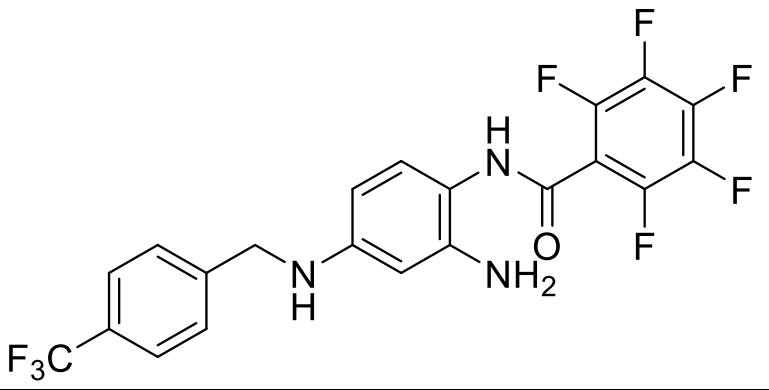	252.98±14.82	5.14±0.30
**c103**	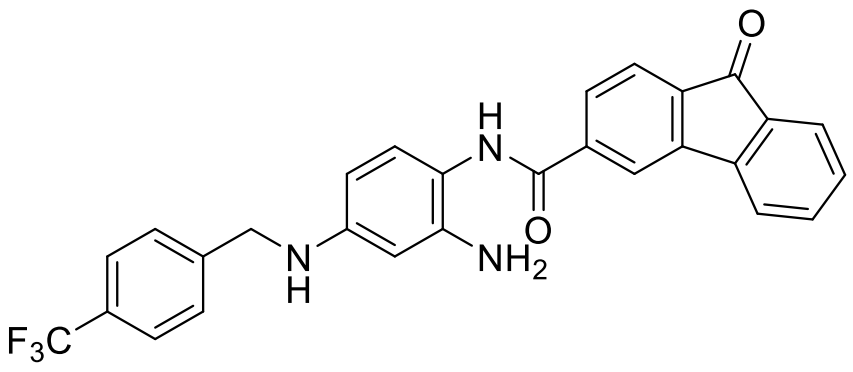	-113.64±39.79	-2.31±0.81
**c104**	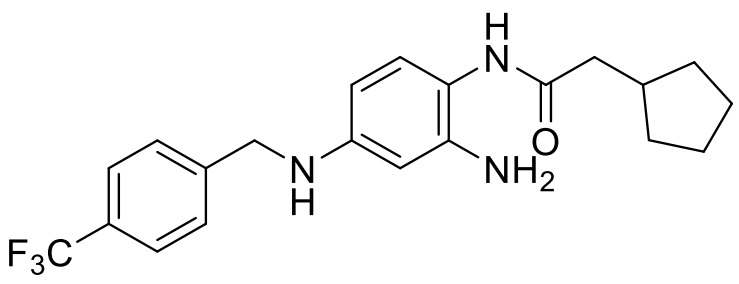	6536.08±23.43	132.91±0.45
**c105**	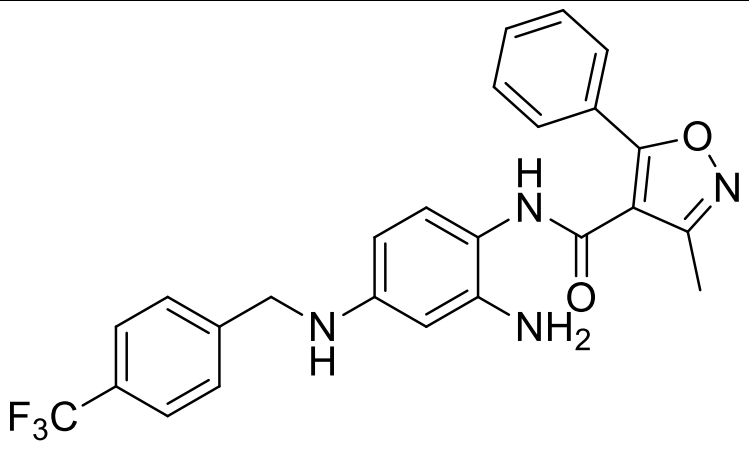	-164.31±90.11	-3.34±1.83
**c106**	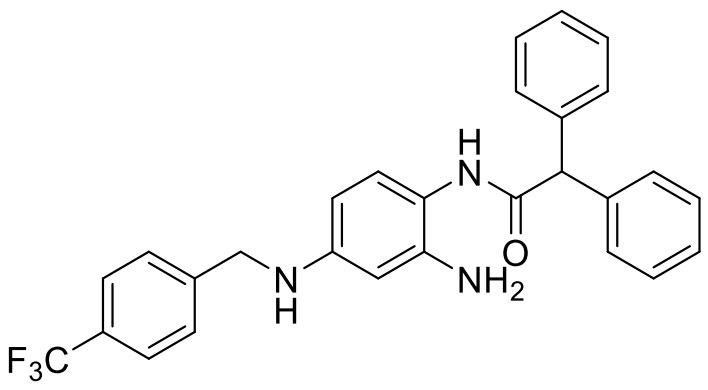	-351.14±69.53	-7.14±1.41
**c107R**	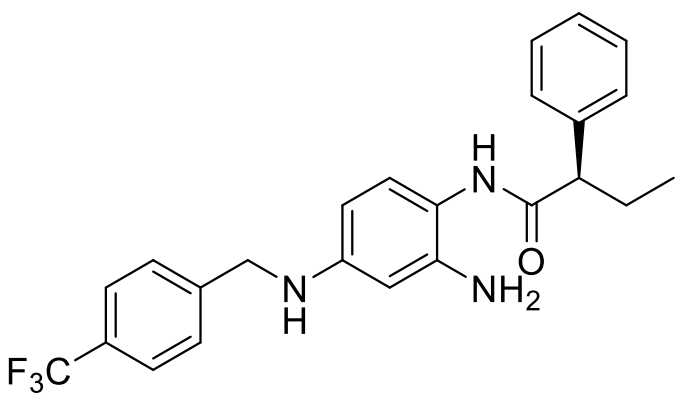	-265.94±1.86	-5.41±0.04
**c107S**	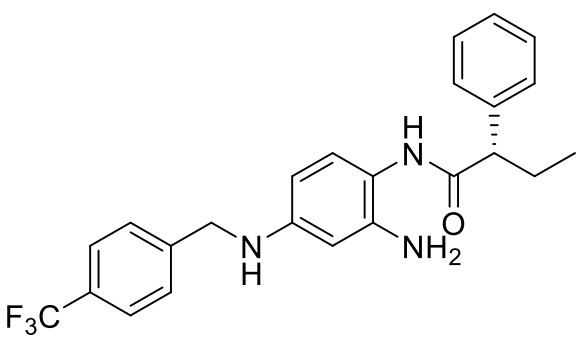	ND	ND
**c108**	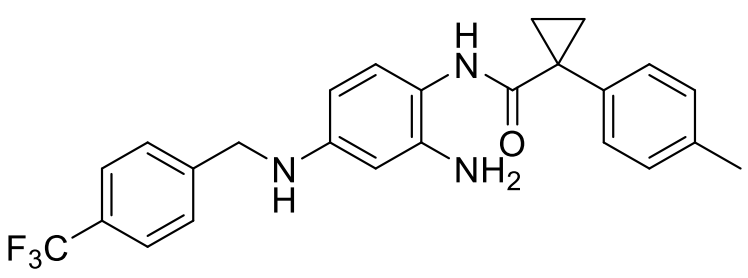	865.98±200.02	17.61±4.07
**c109**	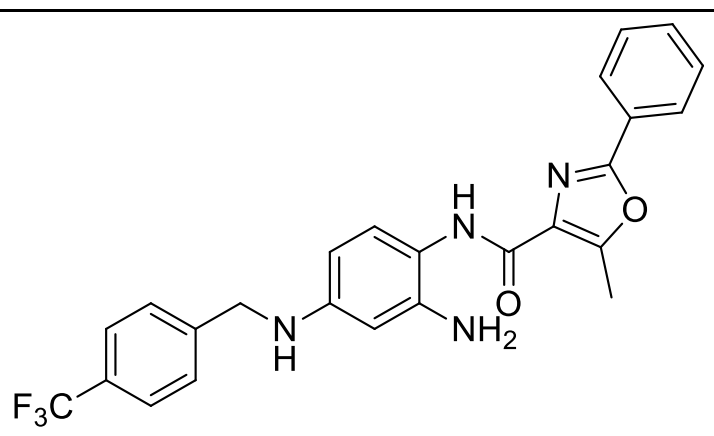	-157.62±11.38	-3.21±0.23

**c111**	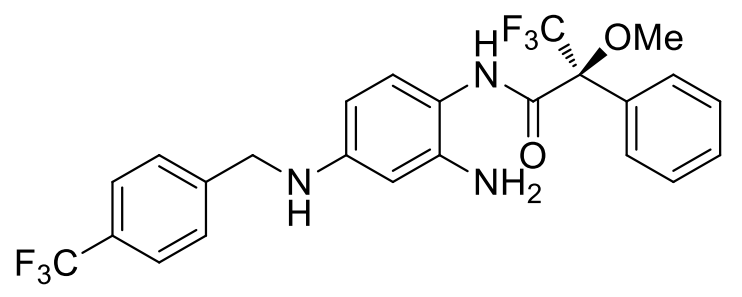	-160.58±23.08	-3.27±0.47
**c112**	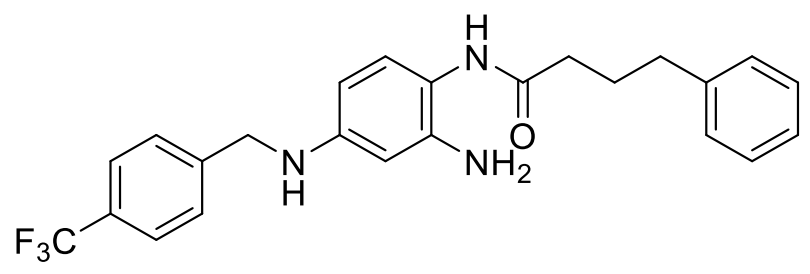	5140.82±353.60	104.54±7.19
**c113**	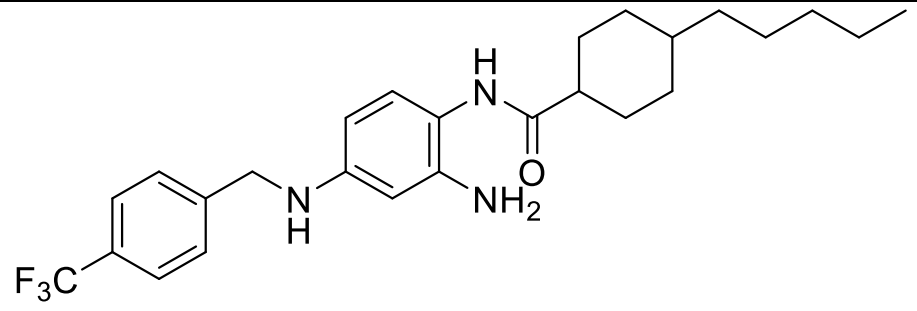	-93.53±73.40	-1.81±1.49
**c114**	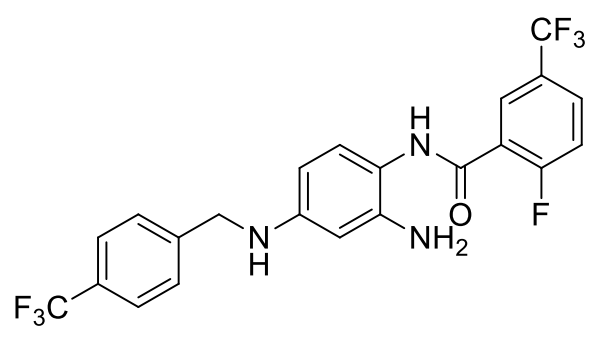	-242.22±17.15	-4.93±0.35
**c115**	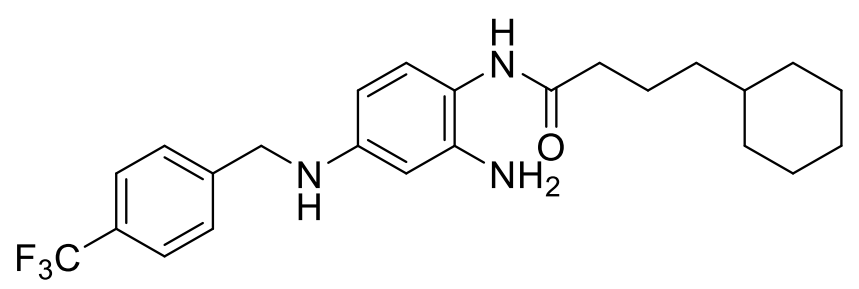	2109.17±282.18	42.89±5.74
**c116**	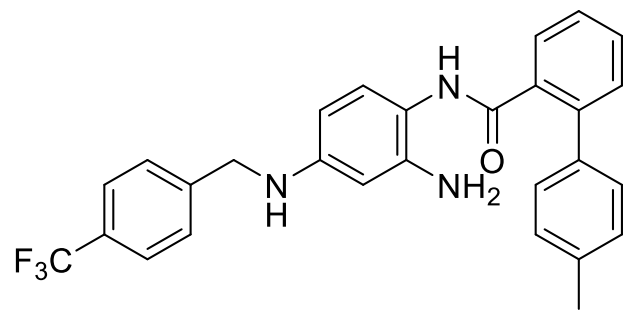	395.36±377.09	8.04±7.67
**c117**	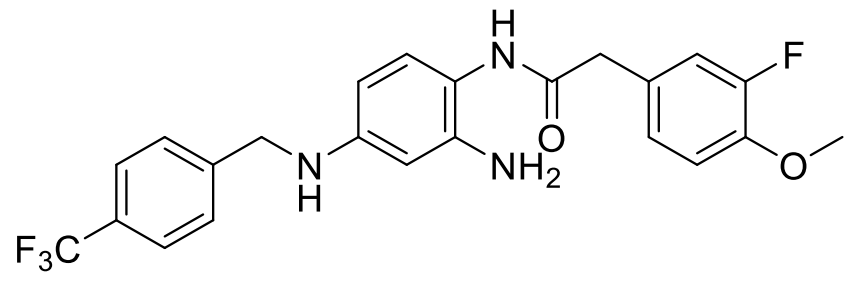	4070.83±486.89	82.78±10.10
**c118**	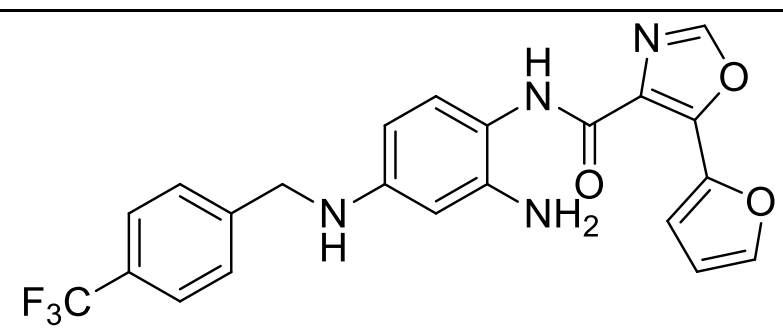	-143.88±62.66	-2.93±1.27
**c119**	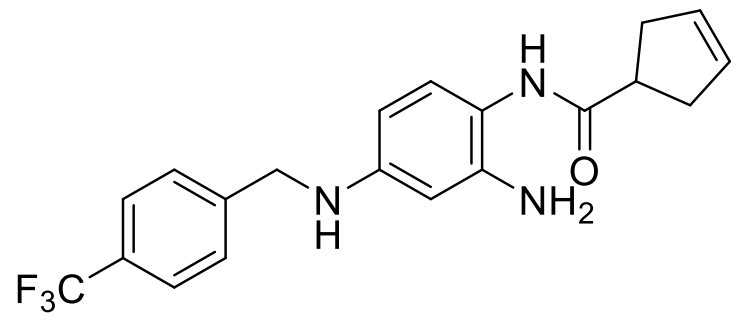	2600.31±21.83	52.58±0.44
**c120**	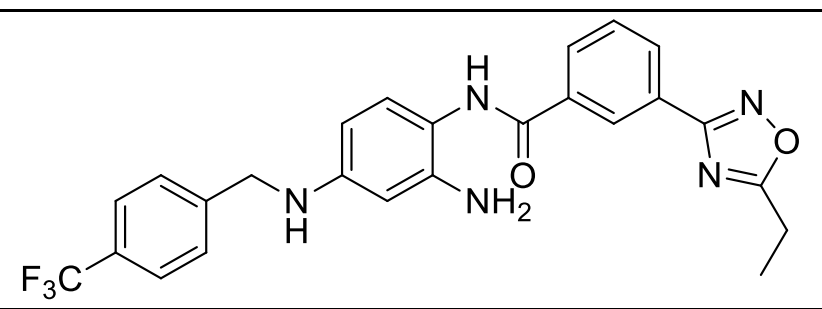	-13.62±17.05	-0.28±0.35
**c121**	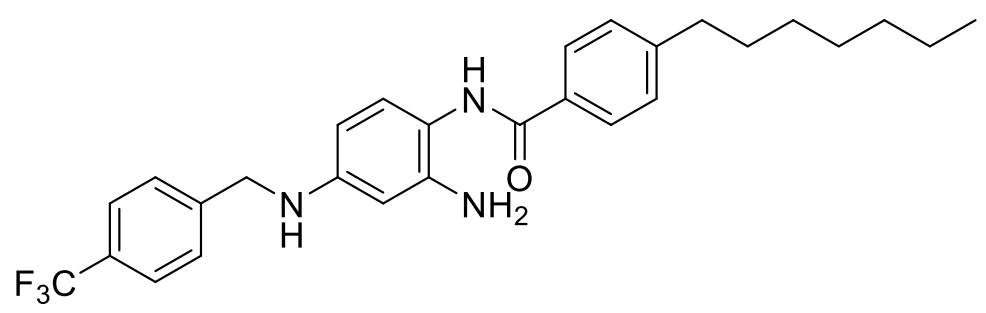	-88.98±73.60	-1.81±1.50

**c122**	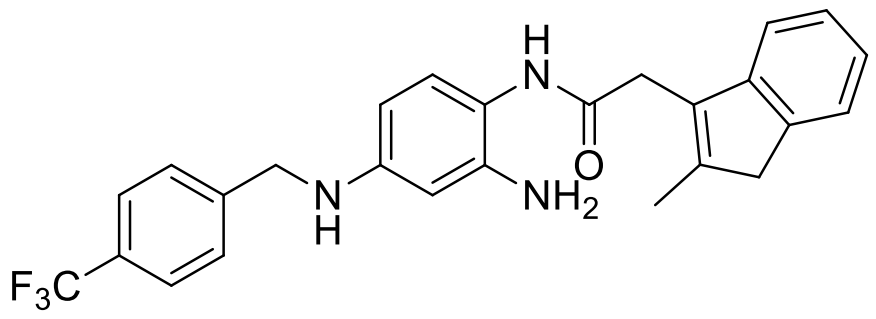	-133.35±6.78	2.71±0.14
**c106F**	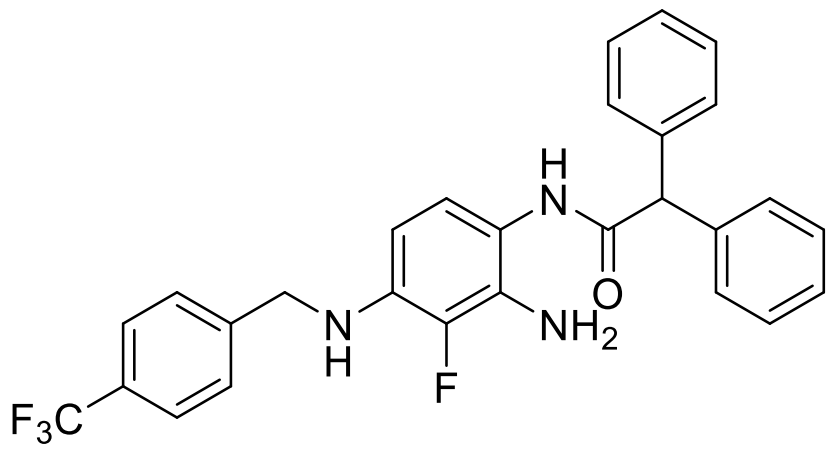	ND	ND

Each data point is the mean ± SEM of 2 cells (n); N=1; ND: Not determined. In red, are the molecules selected for a second screening in the antagonist mode (see text).

3. MANUAL PATCH-CLAMP EXPERIMENTS

3.1 CELL CULTURE, MUTAGENESIS AND TRANSIENT TRANSFECTION

Chinese Hamster Ovary (CHO-K1, RRID: CVCL_0214) cells were cultured in 100-mm plastic Petri dishes in Dulbecco's Modified Eagle Medium (DMEM) containing 10% fetal bovine serum (FBS), nonessential amino acids (0.1 mM), penicillin (50 U/ml), and streptomycin (50 mg/ml) in a humidified atmosphere at 37°C with 5% CO_2. For patch-clamp experiments, cells were plated on glass coverslips coated with poly-L-lysine and transfected with KCNQ1, KCNQ2, KCNQ3, KCNQ4, KCNQ5, KCNQ3 A315T cDNAs, all cloned in pcDNA3.1, together with an enhanced green fluorescent protein plasmid (Clontech, Palo Alto, CA) using Lipofectamine 2000 (Life Technologies). Site-directed mutagenesis was performed by Genscript. Total cDNA in the transfection mixture was kept at 4 µg. Cells were used 24 hours after transfection.

3.2 MANUAL PATCH-CLAMP

Whole-cell patch-clamp recordings in voltage-clamp configuration were performed at room temperature (22-24°C) using a commercially available amplifier (Axopatch 200B, Molecular Devices). Data were sampled at a frequency of 15 kHz, filtered at 5 kHz with the 4-pole low-pass Bessel filter of the amplifier, and digitized using a Digidata 1440A (Molecular Devices, USA). The pCLAMP software (version 10.0.2) was used for data acquisition and analysis. Recording pipettes (5-6 MΩ resistance) were pulled from borosilicate glass capillaries. The extracellular solution contained (in mM): 138 NaCl, 2 CaCl2, 5.4 KCl, 1 MgCl2, 10 glucose, and 10 HEPES, pH 7.4 with NaOH, osmolarity 305-310 mOsm. The pipette (intracellular) solution contained (in mM): 140 KCl, 2 $MgCl_2$,10 EGTA,10 HEPES, 5 Mg ATP, pH 7.3–7.4 with KOH, osmolality 280-290 mOsm. No corrections were made for liquid junction potentials. Cells were clamped at -80 mV and depolarized for 1.5 sec from -120/-80 mV to +20 mV in 10 mV increments, followed by an isopotential pulse to 0 mV (500 ms duration). Conductance-voltage curves (G/V curves) were generated by normalizing to the maximal value of the instantaneous isopotential currents at 0 mV and expressing the normalized values as a function of the preceding voltages. Data were fit to the Boltzmann distribution: $y=max/[1+\exp(V1/2-V)/k]$, where V is the test potential, $V_{1/2}$ the half-activation potential, and *k* the slope factor. To evaluate the activity of each compound on Kv7.2 currents, the cells were clamped at -80 mV, and currents were elicited by 3 s voltage ramps from −80 to +20 mV in the presence and absence of each compound. To estimate the IC_{50}s, the ratio between the maximal current calculated at 0 mV in the presence and in the absence of each compound (I_{DRUG}/I_{Ctl}) at different concentration, was plotted versus log[concentration] of the compound and fitted with the following four-parameters logistic equation: *y=min+(max-min)/(1+(x+EC*$_{50}$*)-HillSlope)*.

3.3 STATISTICAL ANALYSIS

Student t-test or one-way ANOVA followed by post-hoc analysis were used to determine statistical significance between groups according to the different experiments. Differences were considered

statistically significant when $p<0.05$. Data were analysed using the SigmaPlot 12.3 for Windows (Systat Software Inc) or GraphPad Prism 10.2. Values are expressed as the mean ± SEM. For each experiment, the number of individual measures (n; cells patched) and the number of experimental replicas (N; transfections) is given.

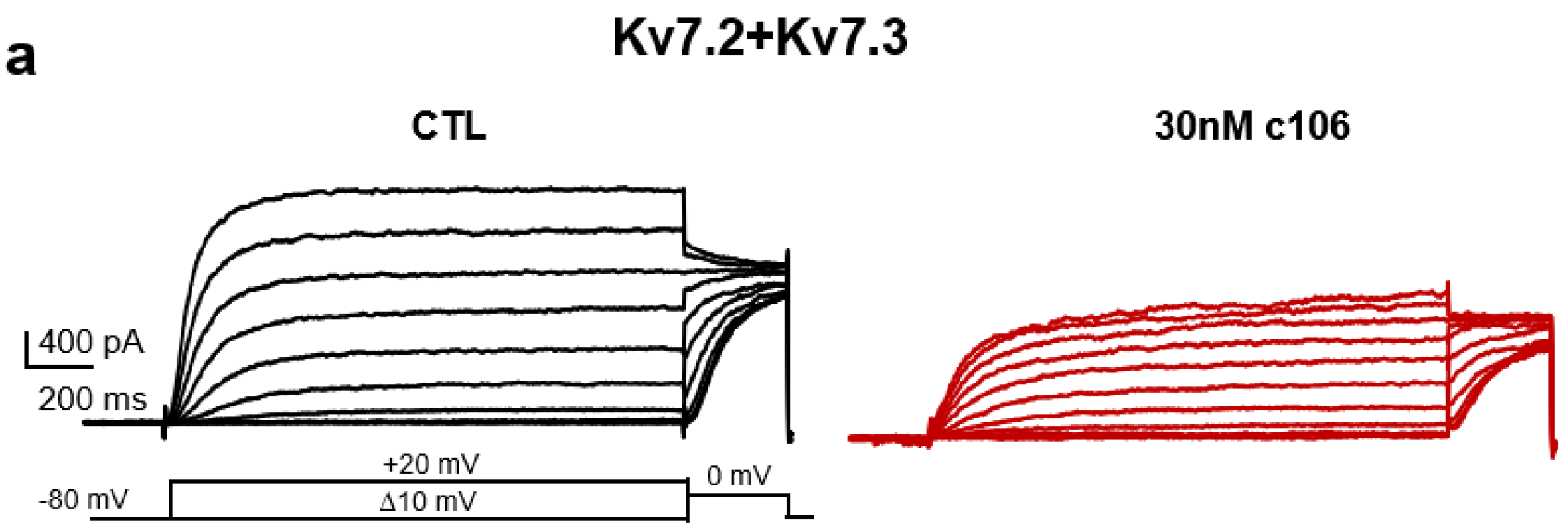

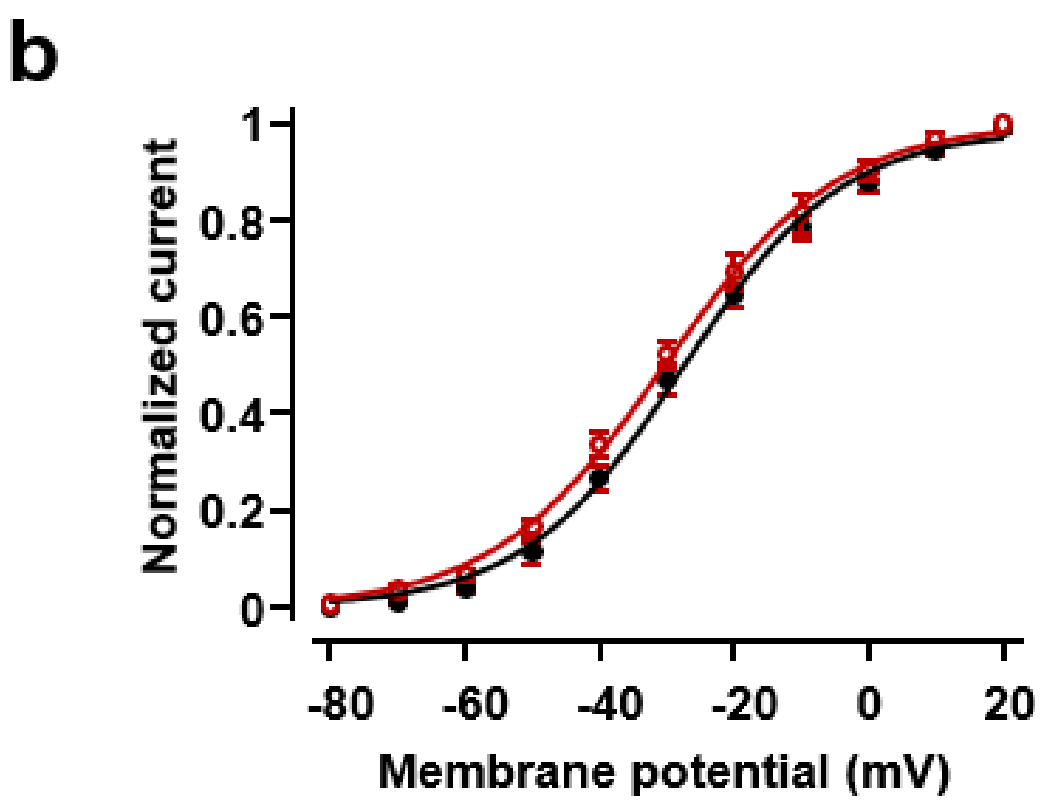

c

	**n, N**	**V½ (mV)**	**k (mV/efold)**
**CTL**	9, 3	-28.5±1.4	13.2±0.9
**30nM c106**	9, 3	-28.7±2.5	14.4±2.0

Figure S83. Effect of c106 on the activation voltage-dependence of Kv7.2/3 current. a Representative macroscopic current traces recorded from a CHO cell expressing Kv7.2/3 channels in response to the indicated voltage protocol before (left) and after (right) application of 0.03 µM **c106**. **b** Normalized Kv7.2/3 conductance/voltage curves before and after application of 30 nM **c106**. Continuous lines represent fits of the experimental data to a Boltzmann distribution. **c** Table showing the effect of **c106** on the midpoint ($V_{1/2}$) and the slope (*k*) of the Boltzmann distribution describing the activation voltage-dependence of Kv7.2/3 currents. The numbers of recorded cells (n) and experimental sessions (N) are also indicated.

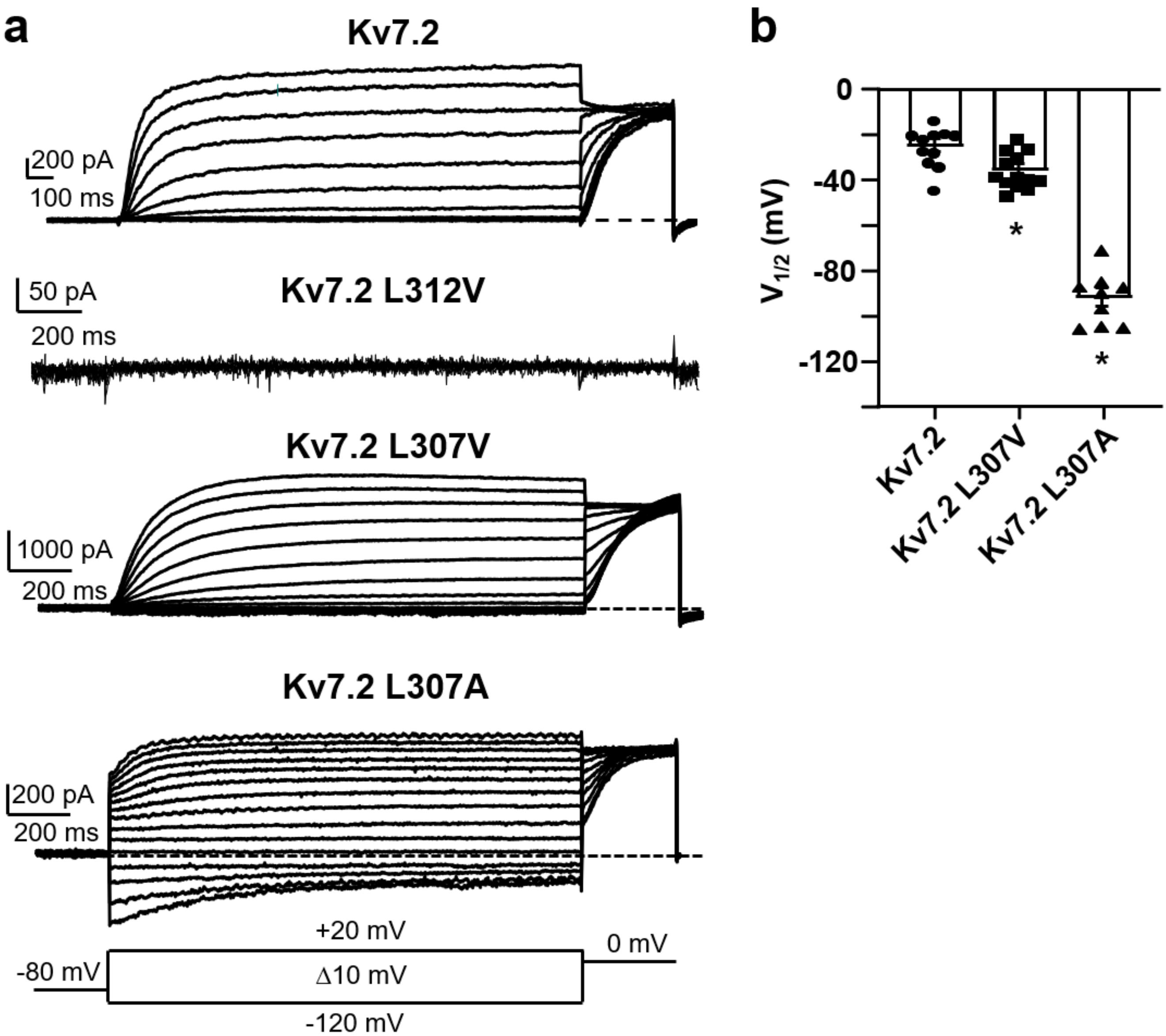

Figure S84. Functional properties of the currents carried by homomeric Kv7.2 (CTL), Kv7.2 L312V, Kv7.2 L307V, and Kv7.2 L307A channels. a Representative macroscopic current traces recorded from a CHO cell expressing Kv7.2, Kv7.2 L312V, Kv7.2 L307V and Kv7.2 L307A homomeric channels in response to the indicated voltage protocol. **b** Representation of the current activation $V_{½}$ values for the indicated channels. Each bar represents the mean ± SEM of 9-13 recorded cells (n), as indicated by the individual dots (N=3). Asterisks denote values significantly different ($p<0.05$) from Kv7.2.

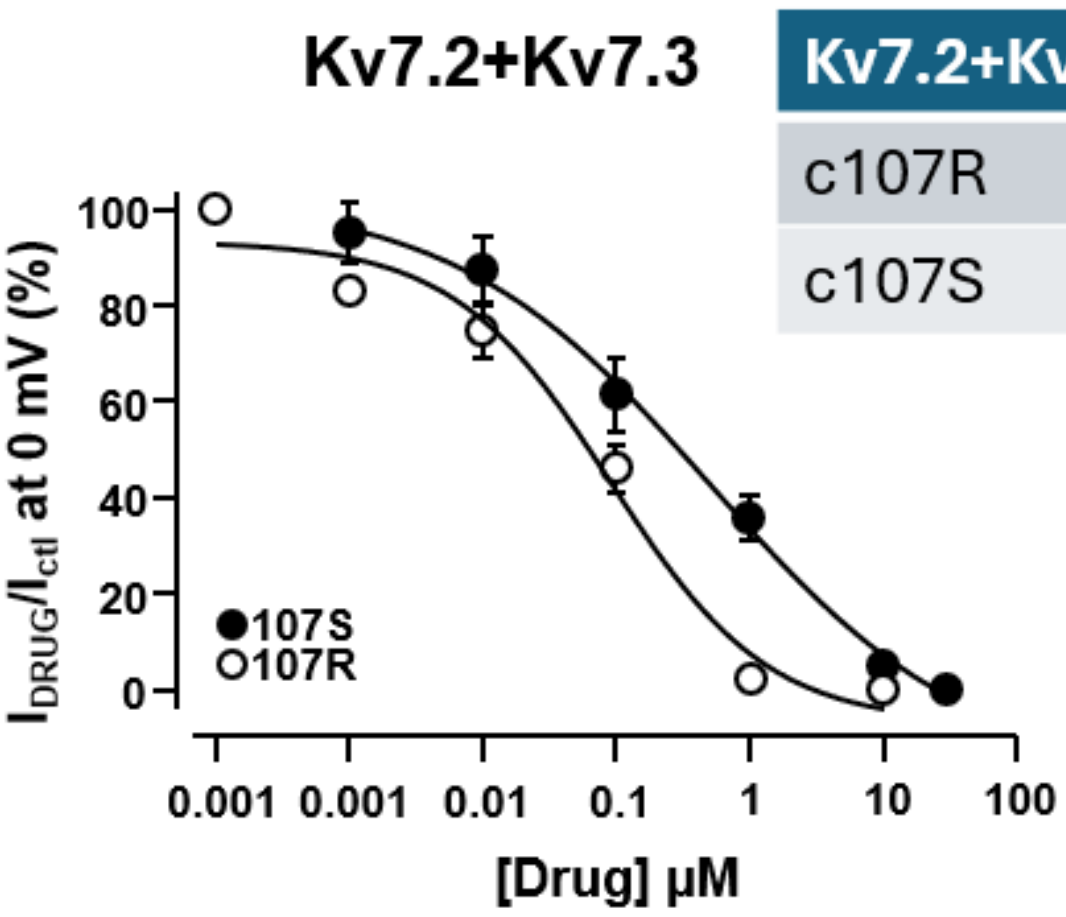

Kv7.2+Kv7.3	IC50 (µM)
c107R	0.08±0.05
c107S	0.51±0.19*

Figure S85. Effects of c107R and c107S on Kv7.2/3 currents. Concentration-response curves reporting the effects of the **c107R** and **c107S** on $Kv_{7.2}$+$Kv_{7.3}$ maximal current density ($I_{drug}/I_{control}$) calculated at +20 mV for and the corresponding IC_{50s}. Each data point is mean ± SEM. The numbers of recorded cells (n) and experimental sessions (N) are the following: c107R (n = 51, N =6) and c107S (n = 46, N =5). * $p < 0.05$ versus C107R.

4. MOLECULAR MODELING

4.1 LIGANDS PREPARATION FOR MOLECULAR MODELLING EXPERIMENTS

Ligands were sketched through the Schrodinger Maestro GUI (*Schrödinger Release 2022−1: Maestro;* Schrödinger, LLC: New York, NY, 2022) and processed through the LigPrep utility (*Schrödinger Release 2022−1: LigPrep;* Schrödinger, LLC: New York, NY, 2022), to get low-energy conformations and account for tautomeric and ionization states at a pH range of 5−9. For each compound, the protonation/tautomeric state displaying the lowest penalty score was selected and used in all subsequent computational studies.

4.2 hKv7.2$_{216\text{-}330}$ MODELS PREPARATION

The Cryo-EM structures of human Kv7.2 in complex with retigabine (PDB 7CR2 – closed state),[27] **c60** (open state), and **c106** (closed state) were processed by retaining only the residues 216–330 for each monomer, corresponding to the Pore Domain region, while the remaining portions of the protein, as well as calmodulin, were removed. Simplified models of the Kv7.2 channel were thus obtained, hereafter referred to as "Kv7.2$_{RET/216\text{-}330\text{-}closed}$" "Kv7.2$_{c60/216\text{-}330\text{-}open}$" and "Kv7.2$_{c106/216\text{-}330\text{-}closed}$". For each structure, only the ligand bound to S6$_{I}$, S5$_{II}$ and S6$_{II}$ was retained.
The Kv7.2$_{216\text{-}330}$ tetrameric structures were processed through the Schrodinger Protein Preparation Workflow,[48] which included capping the protein termini with ACE and NMA residues, optimization of the H-bond network, and restrained energy minimization using the OPLS 2005 force field.[49] The minimization was stopped when the RMSD of heavy atoms reached the default convergence threshold of 0.30 Å.

4.3 INDUCED FIT DOCKING

Standard protocol of Schrodinger Induced Fit Docking [50] was used to dock the previous prepared ligands into the "Kv7.2$_{RET/216\text{-}330\text{-}closed}$" model. Docking space for the initial Glide docking was defined as a 27,000 Å^3 cubic box centered on the experimental bound pose of retigabine, and the ligand diameter midpoint was required to stay inside a smaller, nested 1000 Å^3 box. Ligand and receptor nonpolar atom van der Waals (vdW) radii were scaled by 0.50 factors. A maximum number of 20 poses per ligand was advanced to the Prime refinement stage, where all residues within 5 Å from ligand poses were optimized. For the refinement stage, an implicit membrane, based on OPM [50] entry 7CR2, was set. Final docking was carried out using Glide SP with default settings. A H-bond to residue W236 was imposed as constraint in both docking stages. For **c60** and **c106**, only the best scoring complex was retrieved and used to set MD simulations up.

4.4 GRID PREPARATION OF "Kv7.2$_{c106/216\text{-}330\text{-}CLOSED}$"

The prepared "Kv7.2$_{c106/216\text{-}330\text{-}closed}$" model was used to generate a cubic Glide docking grid, centered on the centroid of **c106** and its volume set to about 23,000 Å^3, requiring the diameter midpoint of each docking ligand to stay with in a smaller, nested 1000 Å^3 cubic grid. No scaling was applied for the van der Waals radii of nonpolar receptor atoms, while H-bond to residue W236 was set as the docking constraint.

4.5 MOLECULAR DOCKING OF c106F

Compound **c106F** was flexibly docked into the "Kv7.2$_{c106/216\text{-}330\text{-}closed}$" model. using Glide SP, [51, 52] retrieving the best scoring pose only. Ligand non polar atom vdW radii were scaled by a factor of 0.80.

4.6 MOLECULAR DOCKING OF c107R, c107S AND ML252

Compounds **c107R**, **c107S** and **ML252** were first docked flexibly into the "Kv7.2$_{c106/216\text{-}330\text{-}closed}$" model using Glide SP. [51, 52] Fifty docking poses per ligand were retrieved and rescored using Glide XP.[53] To avoid redundancy, all poses with an RMSD (Root Mean Square Deviation) or a maximum atomic deviation of any atom below 2.0 Å were discarded as duplicates. For **c107**, the best-scoring predicted bound conformations in both the *ethyl-in* and *phenyl-in* poses were selected for each enantiomer and

used to set MD simulations up. For **ML252**, only the best-scoring predicted bound conformation was retained.

4.7 MOLECULAR DYNAMICS SIMULATIONS OF "c60/Kv7.2$_{RET/216-330-\ CLOSED}$", "c106/Kv7.2$_{RET/216-330-\ CLOSED}$" "Kv7.2$_{c60/216-330-OPEN}$", "Kv7.2$_{c106/216-330-\ CLOSED}$", "c106F/Kv7.2$_{c106/216-330-\ CLOSED}$", "c107R/Kv7.2$_{c106/216-330-\ CLOSED}$", "c107S/Kv7.2$_{c106/216-330\ CLOSED}$", and "ML252/Kv7.2$_{c106/216-330-\ CLOSED}$" COMPLEXES

All MD simulations were set up using Desmond (*Schrödinger Release 2022−1: Desmond Molecular Dynamics System*; D. E. Shaw Research: New York, NY, 2022. Maestro-Desmond Interoperability Tools; Schrödinger: New York, NY, 2022). The complexes were inserted into a POPC bilayer, based on the coordinates of the 7CR2 entry from the OPM database. Solvation was treated explicitly using the TIP3P water model.[54] The systems were neutralized by Na^+ and Cl^-, which were added to a final concentration of 0.15 M. Prior to MD production stage, membrane/ligand/protein systems were equilibrated by means of the standard equilibration stepwise protocol for membrane proteins distributed with Desmond. After system equilibration, 1200 ns-long MD simulations were carried out in the NpγT ensemble, using a Nose−Hoover chain thermostat (300 K) and a Martyna−Tobias−Klein barostat (1atm). Backbone heavy atoms were constrained during the production stage (1 kcal/mol). Time steps were set to 2, 2, and 6 fs for bonded, near, and far interactions, respectively. MD trajectories were analyzed using VMD [55] and the Simulation Interaction Diagram embedded in the Schrodinger Suite. OPLS 2005 [49] was used as force field in all the simulations.

Table S2: Docking-predicted residue interaction scores of **c107R**, **c107S** and **ML252**. Interaction terms are defined as follows: vdW - van der Waals contribution; coul - Coulombic (electrostatic) contribution; hbond - hydrogen-bond contribution; dist - ligand-residue distance; Eint, total interaction energy, calculated as the sum of the vdW, Coulombic, and hydrogen-bond contributions.

Residue	Contribution	c107R (phenyl-in)	c107S (phenyl-in)	c107R (ethyl-in)	c107S (ethyl-in)	ML252
L221	vdW	-0.45	-0.394	-0.741	-0.899	-0.739
	coul	0.081	0.111	0.058	0.065	0.061
	hbond	0	0	0	0	0
	dist	3.789	4.568	3.827	3.379	3.808
	Eint	-0.369	-0.282	-0.683	-0.834	-0.678
V225	vdW	-0.53	-0.41	-1.479	-1.515	-1.637
	coul	-0.012	0.073	0.107	0.057	0.064
	hbond	0	0	0	0	0
	dist	3.524	4.427	2.467	2.291	2.215
	Eint	-0.542	-0.338	-1.372	-1.458	-1.573
L232	vdW	-1.313	-1.564	-1.934	-1.884	-2.296
	coul	0.036	0.257	0.193	0.025	0.098
	hbond	0	0	0	0	0
	dist	2.73	2.493	2.789	2.6	2.232
	Eint	-1.277	-1.307	-1.741	-1.859	-2.199
W236	vdW	-5.253	-3.766	-5.43	-6.441	-6.382
	coul	-2.811	-4.205	-2.706	-2.951	-2.494
	hbond	-0.567	-1	-0.764	-0.826	-0.797
	dist	2.081	1.686	1.934	1.896	1.969
	Eint	-8.632	-8.97	-8.9	-10.218	-9.673
L312	vdW	-1,618	-1,381	-1,349	-1,555	-1,801
	coul	0,088	-0,046	-0,011	0,032	0,03
	hbond	0	0	0	0	0
	dist	2,235	2,161	2,208	2,375	2,166
	Eint	-1,53	-1,427	-1,36	-1,523	-1,771
I300	vdW	-4.935	-5.023	-4.853	-4.829	-5.08
	coul	-0.583	0.267	-0.329	-0.648	-0.498
	hbond	0	0	0	0	0
	dist	2.15	1.998	2.255	2.376	1.96
	Eint	-5.518	-4.757	-5.182	-5.477	-5.578
F304	vdW	-4.093	-3.481	-2.493	-2.762	-2.339
	coul	0.064	0.101	-0.087	0.058	-0.118
	hbond	0	0	0	0	0
	dist	2.33	2.111	2.349	2.512	2.669
	Eint	-4.029	-3.38	-2.58	-2.704	-2.456
L307	vdW	-1,445	-1,678	-0,396	-0,668	-0,41
	coul	0,114	0,126	0,13	0,125	0,106
	hbond	0	0	0	0	0
	dist	2,448	2,29	3,514	3,01	3,54
	Eint	-1,33	-1,551	-0,266	-0,543	-0,304

Table S3: Docking scores of **c107R**, **c107S** and **ML252.**

Compound	*Docking Score*
**c107R (*phenyl-in*)**	-11.300
**c107S (*phenyl-in*)**	-10.918
**c107R (*ethyl-in*)**	-9.655
**c107S (*ethyl-in*)**	-9.232
**ML252**	-8.686

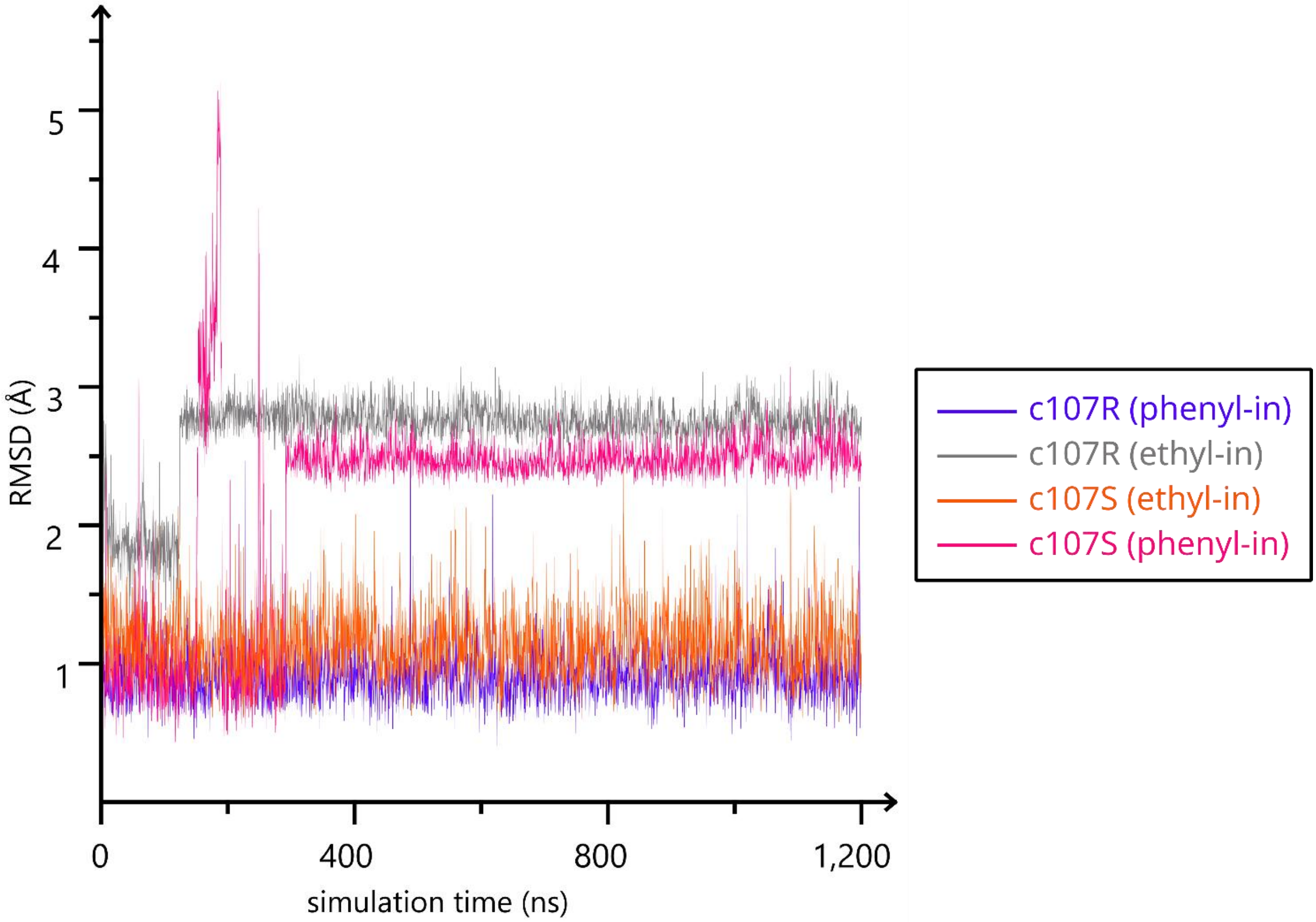

Figure S86: RMSD values of **c107R** and **c107S** (*phenyl-in* and *ethyl-in* pose) as a function of MD simulation time.

Table S4: Average distances (Å) between Kv7.2 residues F304, L307, and L312 and the terminal groups of activators **c60** and retigabine (**RET**), and Kv7 blockers. Distances between ligands and protein atoms are averaged over 1200ns-long MD simulations and are reported together with their standard deviations (4000 frames). MD frames were superimposed by their protein backbone. Distances have been calculated between the centroids of the side-chain heavy atoms of the residues and the centroids of the ligand terminal groups: tert-butyl for **c60**; phenyl for **c106** and **c106F;** ethyl for **ML252** and **RET**; phenyl and ethyl for **c107R** and **c107S.** For the ligands, only heavy atoms of the substituents closest to the protein residues were considered for distance calculations.

Compound	*F304*	*L307*	*L312*
**RET**	5.13±0.33	7.40±0.47	6.17±0.43
**c60**	6.45±0.32	9.48±0.39	6.27±0.37
**c106**	4.89±0.19	5.63±0.19	5.20±0.21
**c106F**	5.07±0.30	5.76±0.43	5.06±0.23
**c107R (*phenyl-in*)**	5.00±0.23	5.73±0.24	5.21±0.21
**c107R (*ethyl-in*)**	5.04±0.25	5.81±0.27	5.18±0.21
**c107S (*phenyl-in*)**	5.35±0.43	5.97±0.37	5.06±0.27
**c107S (*ethyl-in*)**	5.35±0.43	5.96±0.37	5.06±0.28
**ML252**	4.70±0.66	6.35±0.82	5.26±0.56

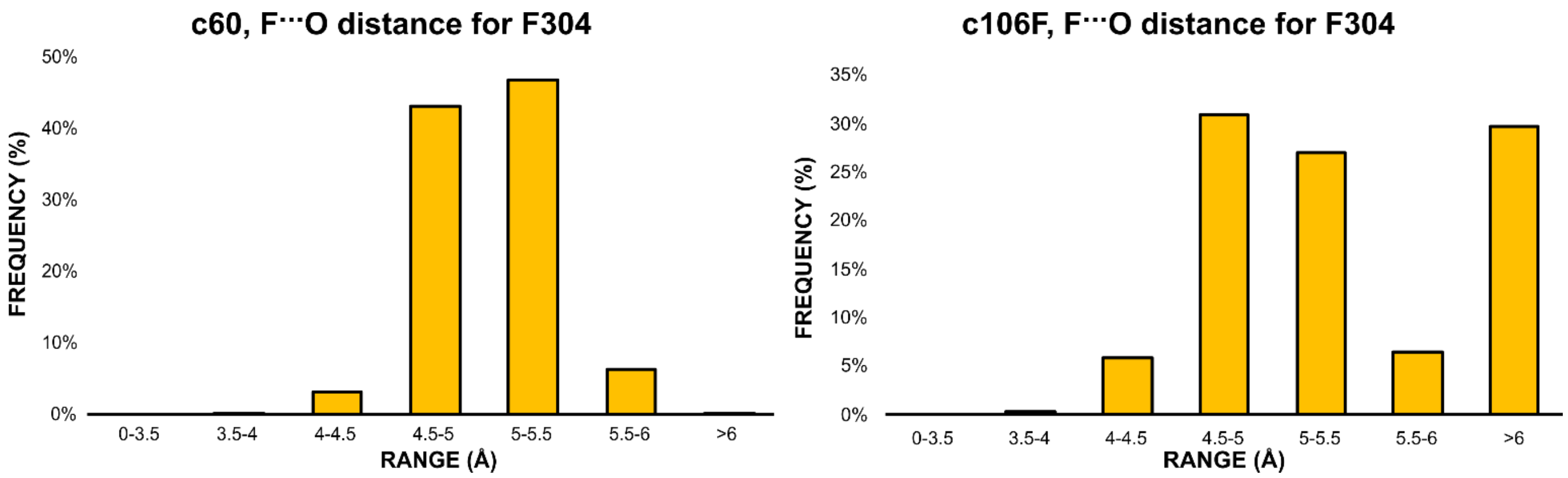

Figure S87: Distance distribution calculated between the fluorine atom of **c60** (left) and **c106F** (right) and the carbonyl oxygen of the backbone of F304 during 1200ns-long MD simulations.

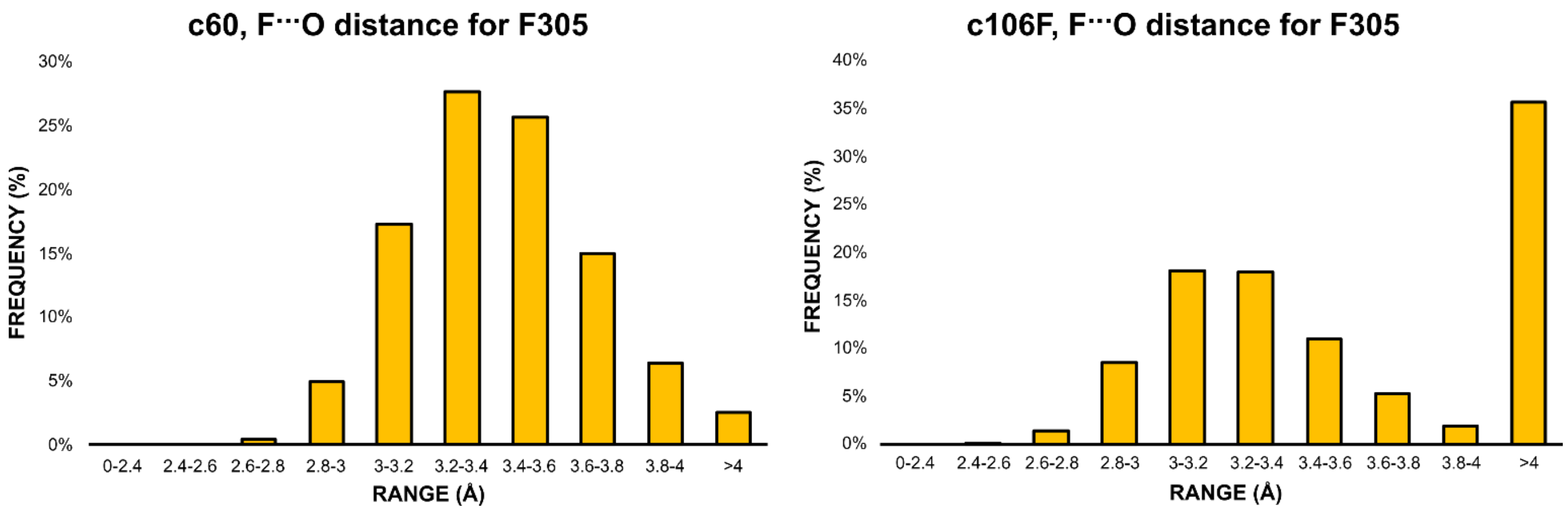

Figure S88: Distance distribution calculated between the fluorine atom of **c60** (left) and **c106F** (right) and the carbonyl oxygen of the backbone of F305 during 1200ns-long MD simulations.

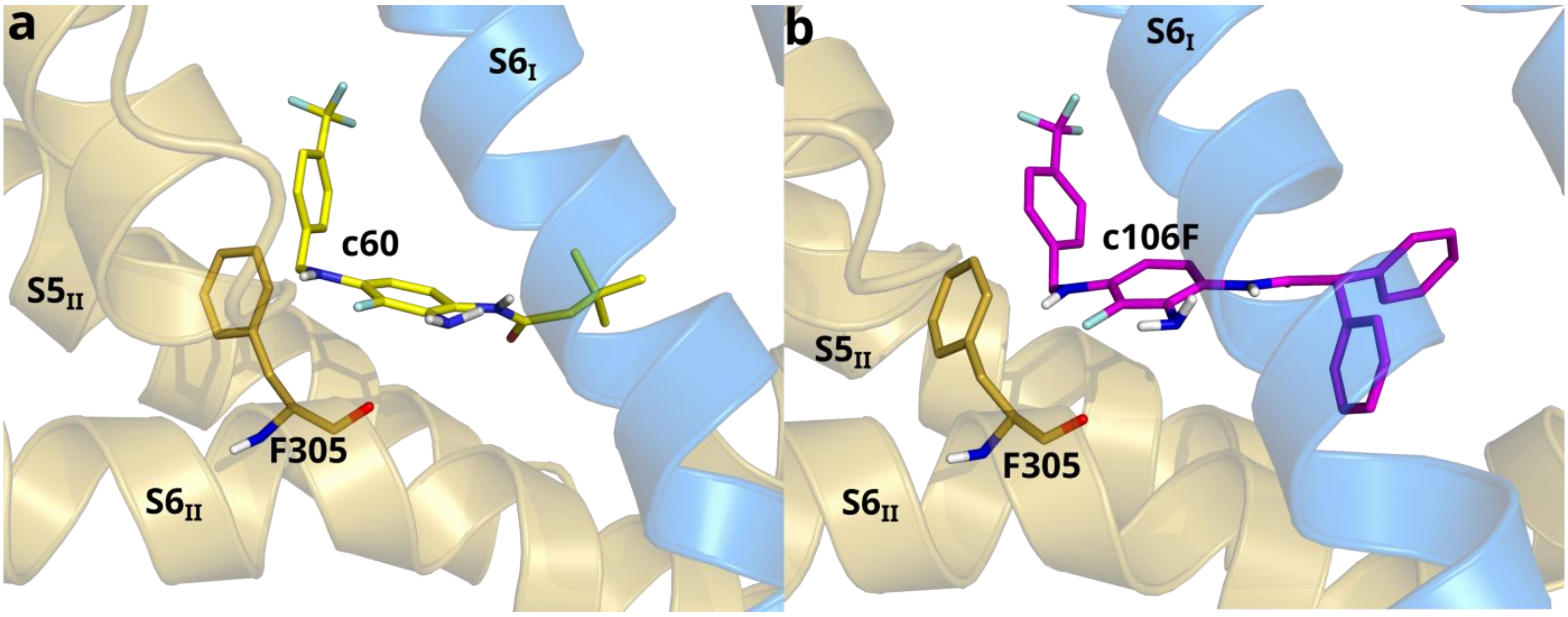

Figure S89: a-b Representative bound conformations of compound **c60** (**a** – yellow sticks) and **c106F** (b – magenta sticks) from the most populated conformational clusters from the MD simulations. The two different Kv7.2 monomers are depicted in sticks and transparent cartoons and colored in gold and marine blue.

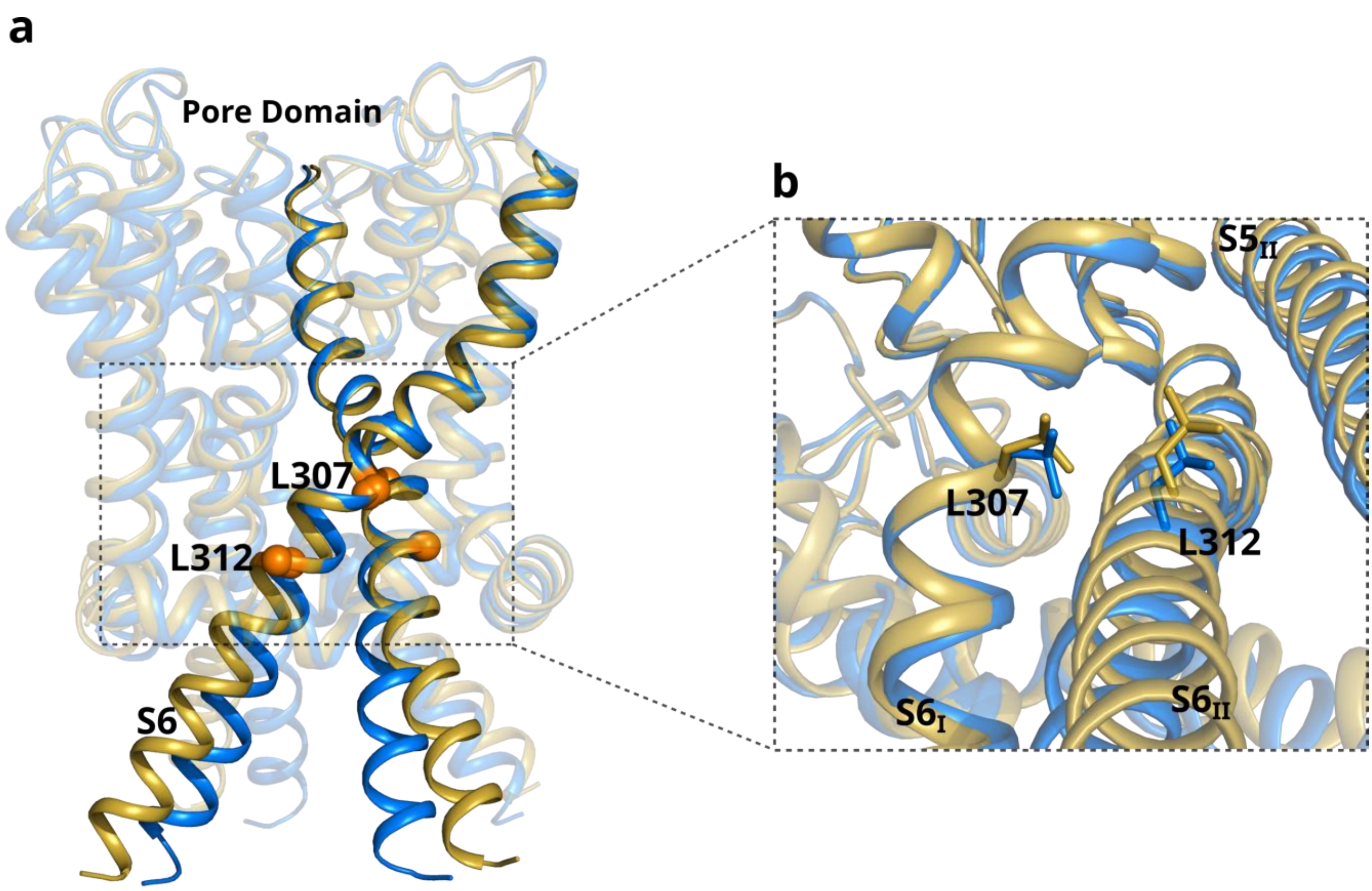

Figure S90: **a** Superimposed transmembrane Pore Domains of Kv7.2-CaM-PIP2-HN37 (open state – PDB 8W4U) and Kv7.2 -CaM-HN37 (closed state – PDB 8J04) cryo-EM structures, depicted in cartoons and colored in gold and marine blue, respectively. The Cα of residues L307 and L312 are shown as orange spheres. **b** Conformational changes of residue L312, depicted in sticks.

5. SINGLE-PARTICLE CRYO-ELECTRON MICROSCOPY

5.1 PROTEIN EXPRESSION AND PURIFICATION

Genes encoding human Kv7.2 (Homo sapiens: NP_004509.2) and calmodulin (CaM; Homo sapiens: NP_001734.1) were synthesized by GenScript. A truncated human Kv7.2 construct encompassing residues 64–674 (with N-terminal and C-terminal truncations) was cloned into the pEZT-BM vector, which harbored a C-terminal Strep-tag.[56] A separate pEZT-BM vector carrying a C-terminal histidine tag was constructed for CaM expression. Heterologous expression of Kv7.2 and CaM was conducted in HEK293S cells (Life Technologies) cultured in SMM 293-TI complete medium (Sino Biological Inc.) supplemented with 2% fetal bovine serum (FBS; Yeasen Biotechnology (Shanghai) Co., Ltd.) at 37°C under 8% CO_2. Baculoviruses were amplified through three sequential passages in suspension-cultured SF9 cells maintained at 27°C. P2-generation baculoviruses encoding Kv7.2 and CaM were co-infected into HEK293S cells (density: 4×10^6 cells/mL) at a volume ratio of 4:1, followed by incubation at 37°C for 12 h. Protein expression was induced by 10 mM sodium butyrate, and cells were subsequently cultured at 30°C. After 48 h of induction, cells were harvested, flash-frozen in liquid nitrogen, and stored at −80°C until further use.

For extraction of the Kv7.2-CaM complex, frozen cells were resuspended in Buffer A (20 mM Tris-HCl, pH 8.0, 150 mM KCl) supplemented with a protease inhibitor cocktail (2 µg/mL DNase I, 0.5 µg/mL pepstatin, 2 µg/mL leupeptin, 1 µg/mL aprotinin, and 1 mM PMSF) and lysed by sonication on ice. To solubilize the Kv7.2-CaM complex from cellular membranes, 2% n-dodecyl-β-D-maltoside (DDM; Anatrace) and 0.4% cholesteryl hemisuccinate tris salt (CHS; Anatrace) were added to the cell lysate, which was then gently agitated at 4°C for 2.5 h. The solubilized lysate was centrifuged at 48,000 × g for 60 min at 4°C, and the supernatant was collected and incubated with Strep-Tactin Sepharose resin (IBA) at 4°C for 2.5 h with gentle rotation. The resin was transferred to a gravity flow column and sequentially washed with 4 column volumes (CVs) of Buffer B (Buffer A supplemented with 0.05% DDM and 0.01% CHS) and 16 CVs of Buffer C (Buffer A supplemented with 0.06% GDN). The Kv7.2-CaM complex was eluted from the resin using Buffer C containing 10 mM desthiobiotin (Sigma). The eluted protein was further purified by size-exclusion chromatography (SEC) using a Superose 6 Increase 10/300 GL column (Cytiva) equilibrated with Buffer D (Buffer A supplemented with 0.02% GDN).

Stock solutions of c60 and c106 (100 mM each) were prepared by dissolving the compounds in 100% DMSO, and further diluted to a final concentration prior to use. A 10 mM stock solution of PIP_2 was prepared in ddH_2O and diluted to a final concentration of 1 mM. For preparation of c60- or c106-bound Kv7.2-CaM complexes, peak fractions of the SEC-purified Kv7.2-CaM complex were collected and incubated with 125 µM c60 or 100 µM c106 at 4°C for 1 h. The mixture was then concentrated to ~22 mg/mL using a 100-kDa molecular weight cutoff concentrator (Amicon Ultra, Millipore Sigma) for cryo-EM sample preparation. For Kv7.2-CaM bound to c60 with PIP_2, incubate the concentrated sample with 1 mM PIP_2 for 1 hour at 4°C before with c60. Both c60 and c106 were provided by the Department of Pharmacy, University of Salerno (Fisciano, Italy) and synthesized as described above. The PIP_2 (phosphatidylinositol-4,5-bisphosphate) was purchased from Merck.

5.2 CRYO-EM DATA ACQUISITION

For cryo-EM grid preparation, 3 µL of the concentrated protein sample was applied to a Quantifoil R1.2/1.3 grid. Excess sample was blotted for 4.5 s at 4°C under 100% humidity using a Vitrobot Mark IV (FEI), and the grid was subsequently plunge-frozen in liquid ethane. Cryo-EM data were collected on a 300-keV FEI Titan Krios transmission electron microscope equipped with a Falcon 4 direct electron detector (Thermo Fisher Scientific), located at the Center of Cryo Electron Microscopy, Zhejiang University. Data acquisition was performed using SerialEM software following standard protocols. Movies were recorded at a magnification of 130,000×, corresponding to a calibrated pixel size of 0.93 Å. The defocus range for data collection was set to −1.5 µm to −0.8 µm. Each movie stack consisted of 40 frames, with a total electron dose of 52 $e^-/Å^2$.

5.3 IMAGE PROCESSING

Motion correction of movie stacks was performed using MotionCorr2,[57] and contrast transfer function (CTF) parameters of the corrected micrographs were estimated using Gctf.[58] All subsequent image processing steps were carried out using RELION 3.1.[59] For the Kv7.2-c60 dataset, a total of 1,367,774 particles were autopicked from 3181 micrographs and extracted with a binning factor of 3 for two-dimensional (2D) classification. 1,236,785 particles were selected from 2D classification and subjected to three-dimensional (3D) classification using the human Kv7.2-CaM complex structure (PDB: 7CR3) [27] as the initial reference. Following two rounds of 3D classification, a single 3D class containing 104,602 particles was selected for 3D reconstruction. These particles were re-extracted at the original pixel size (0.93 Å) and subjected to 3D refinement with *C4* symmetry imposed, yielding a final density map with a resolution of 3.1 Å. Image processing procedures for the Kv7.2-c106 dataset were identical to those used for the Kv7.2-c60 dataset.

5.4 MODEL BUILDING, REFINEMENT, AND VALIDATION

Model building was performed in Coot [60] using the Kv7.2-CaM complex structure (PDB: 7CR3) [27] as the initial template. Assignment of amino acid residues was facilitated by well-resolved electron density for bulky side chains (e.g., F, W, Y, and R). Both the transmembrane domain (TMD) and cytosolic domains were built into the density map. Real-space refinement was performed in PHENIX [61] with secondary structure restraints and non-crystallographic symmetry (NCS) constraints applied. Geometric statistics of the refined models are summarized in Table S1, which were generated using MolProbity.[62] All structural figures were prepared using UCSF ChimeraX.[63]

Table S5. Data collection and refinement statistics.

	Kv7.2-c60	Kv7.2-c106	Kv7.2-PIP$_2$(-)-c106
Data collection and processing			
Magnification	13000	13000	13000
Voltage (kV)	300	300	300
Electron exposure (e-/Å2)	~52	~52	~52
Defocus range (μm)	-0.8 to -1.5	-0.8 to -1.5	-0.8 to -1.5
Pixel size (Å)	0.93	0.93	0.93
Symmetry imposed	*C4*	*C4*	*C4*
Initial particle images (no.)	1,367,774	1,804,325	991,535
Final particle images (no.)	104,602	188,834	74,066
Map resolution (Å)	3.1	3.0	3.8
FSC threshold	0.143	0.143	
Refinement			
Initial model used (PDB code)	PDB 7CR3	PDB 7CR3	
Model resolution (Å)	3.25	7.10	
FSC threshold	0.5	0.5	
Map sharpening B factor (Å^2)	-55	-75	
Model composition			
Non-hydrogen atoms	16164	15424	
Protein residues	1896	1896	
Ligands	8	4	
B factors (Å2)			
Protein	109.15	74.66	
Ligand	76.29	47.50	
R.m.s. deviations			
Bond lengths (Å)	0.007	0.004	
Bond angles (°)	0.982	0.906	
Validation			
MolProbity score	1.66	2.25	
Clashscore	9.51	12.51	
Rotamer outliers (%)	0.24	2.99	
Ramachandran plot			
Favored (%)	97.14	95.92	
Allowed (%)	2.86	4.08	
Disallowed (%)	0.00	0.00	

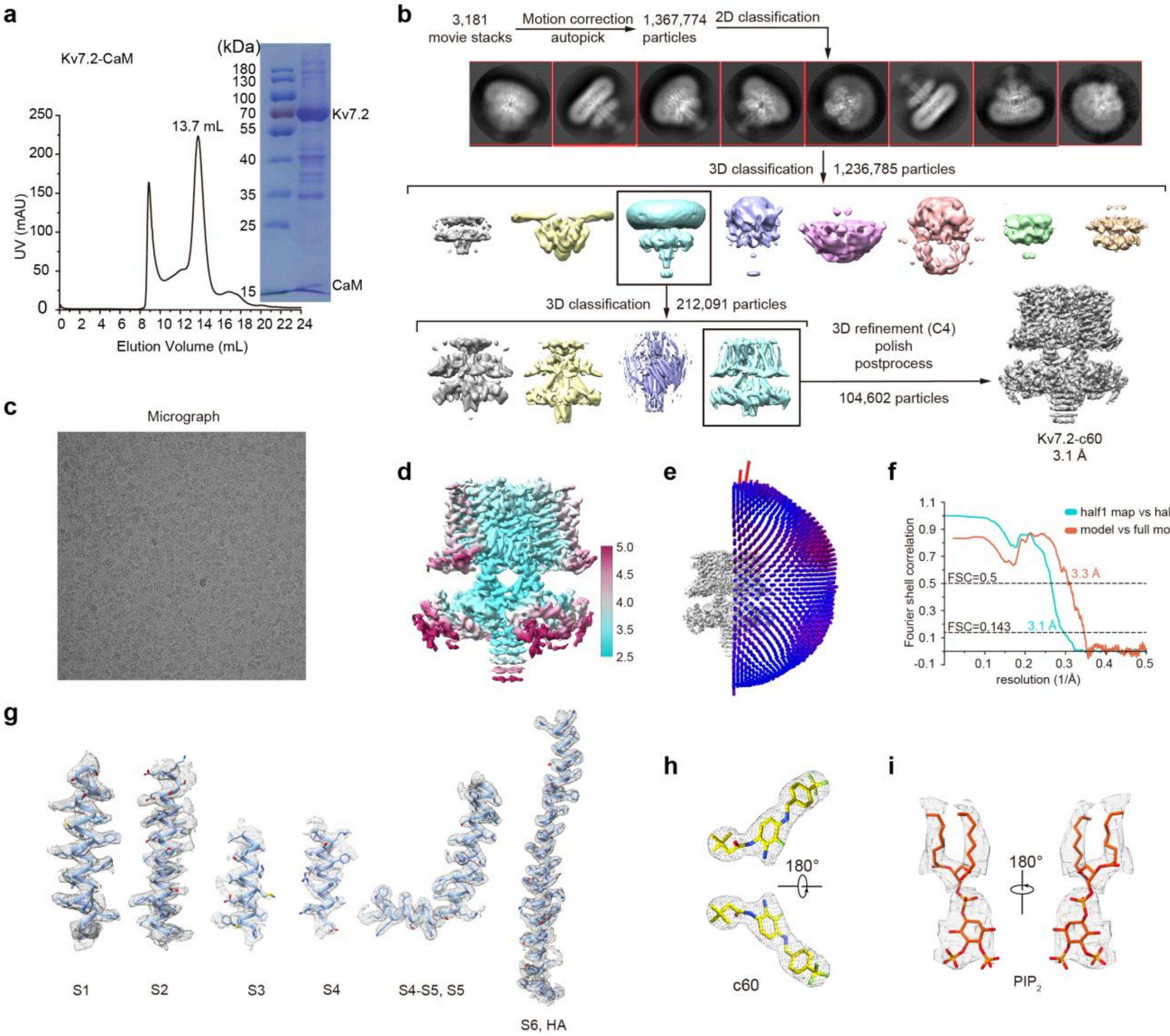

Figure S91. Structure determination of Kv7.2-c60. (**a**) Size-exclusion chromatogram of Kv7.2-CaM on Superose 6 (GE Healthcare) and representative SDS-PAGE analysis (Coomassie stained) of purified protein used for cryo-EM grid preparation. (**b**) Cryo-EM data processing workflow for Kv7.2-c60 particles. (**c**) Cryo-EM micrograph for Kv7.2-c60 sample (of representative quality for all collected cryo-EM data reported in this study). (**d**) Local resolution analysis of the final reconstruction. (**e**) Euler angle distribution of Kv7.2-c60 particles used in the final 3D reconstruction, with the heights of the cylinders corresponding to the number of particles. (**f**) The Gold-standard Fourier shell correlation (FSC) curves for the final 3D reconstruction of Kv7.2-c60 and the cross validation between the map and the model of Kv7.2-c60. (**g**) Cryo-EM density corresponding to KV7.2 S1–S6 and HA at the contour level of 0.007 in UCSF ChimeraX. (**h**) The density map of **c60** molecules in different orientations at the contour level of 0.007 in UCSF ChimeraX. (**i**) The density map of PIP_2 molecule in different orientations at the contour level of 0.007 in UCSF ChimeraX.

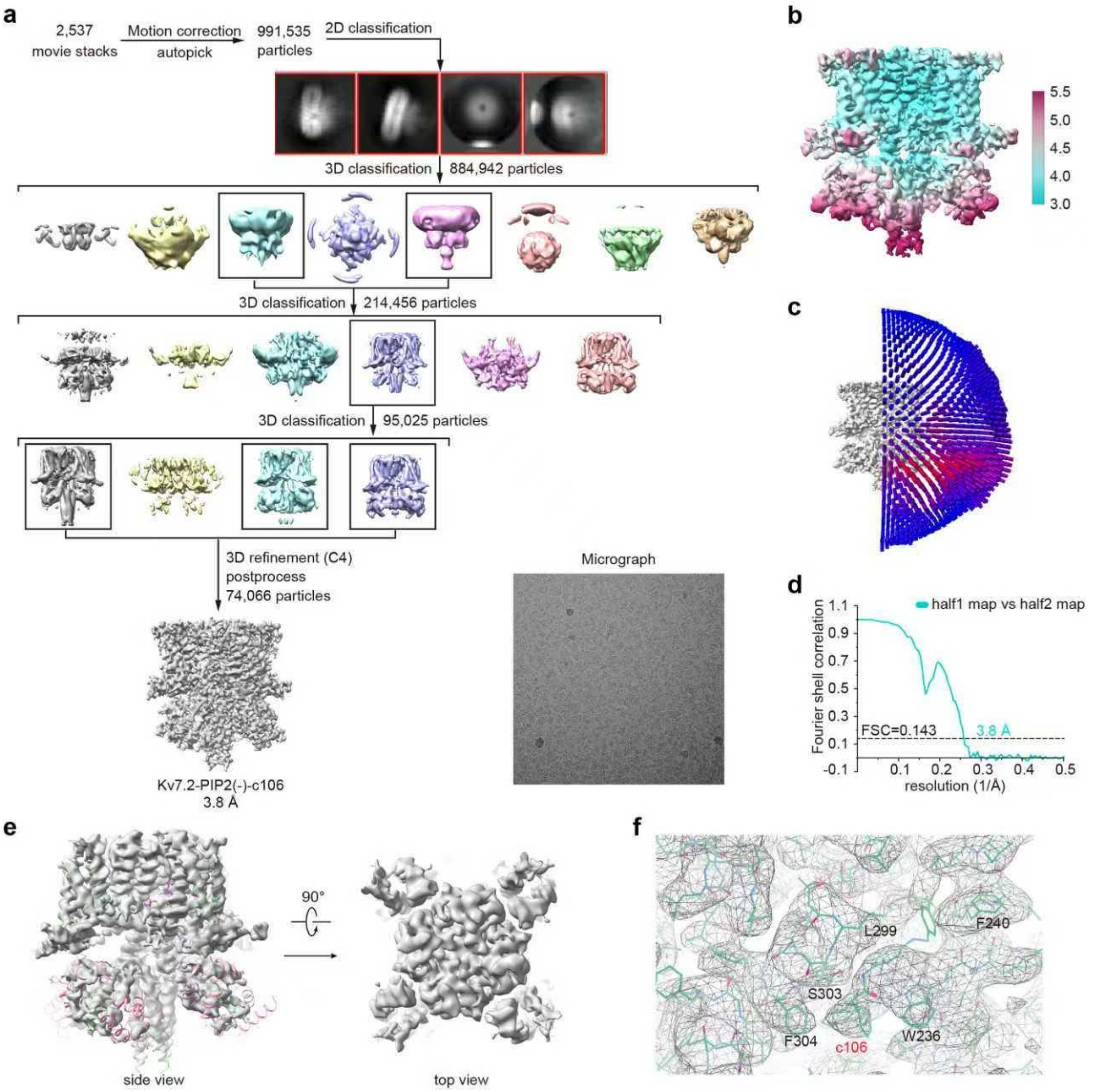

Figure S92. Structure determination of Kv7.2-PIP$_2$(-)-c106 in the presence of PIP$_2$. (**a**) Cryo-EM data processing workflow for Kv7.2-PIP$_2$(-)-c106 particles and representative cryo-EM micrograph. (**b**) Local resolution analysis of the final reconstruction. (**c**) Euler angle distribution of Kv7.2-PIP$_2$(-)-c106 particles used in the final 3D reconstruction, with the heights of the cylinders corresponding to the number of particles. (**d**) The Gold-standard Fourier shell correlation (FSC) curves for the final 3D reconstruction of Kv7.2-PIP$_2$(-)-c106. (**e**) Alignment of the electron density map of Kv7.2-PIP$_2$(-)-c106 with the model of Kv7.2-c106 shown in side, top, and bottom views at the contour level of 0.009 in UCSF ChimeraX. (**f**) Density around **c106** binding site in Kv7.2-PIP$_2$(-)-c106 at the contour level of around 4.0 σ in Coot.

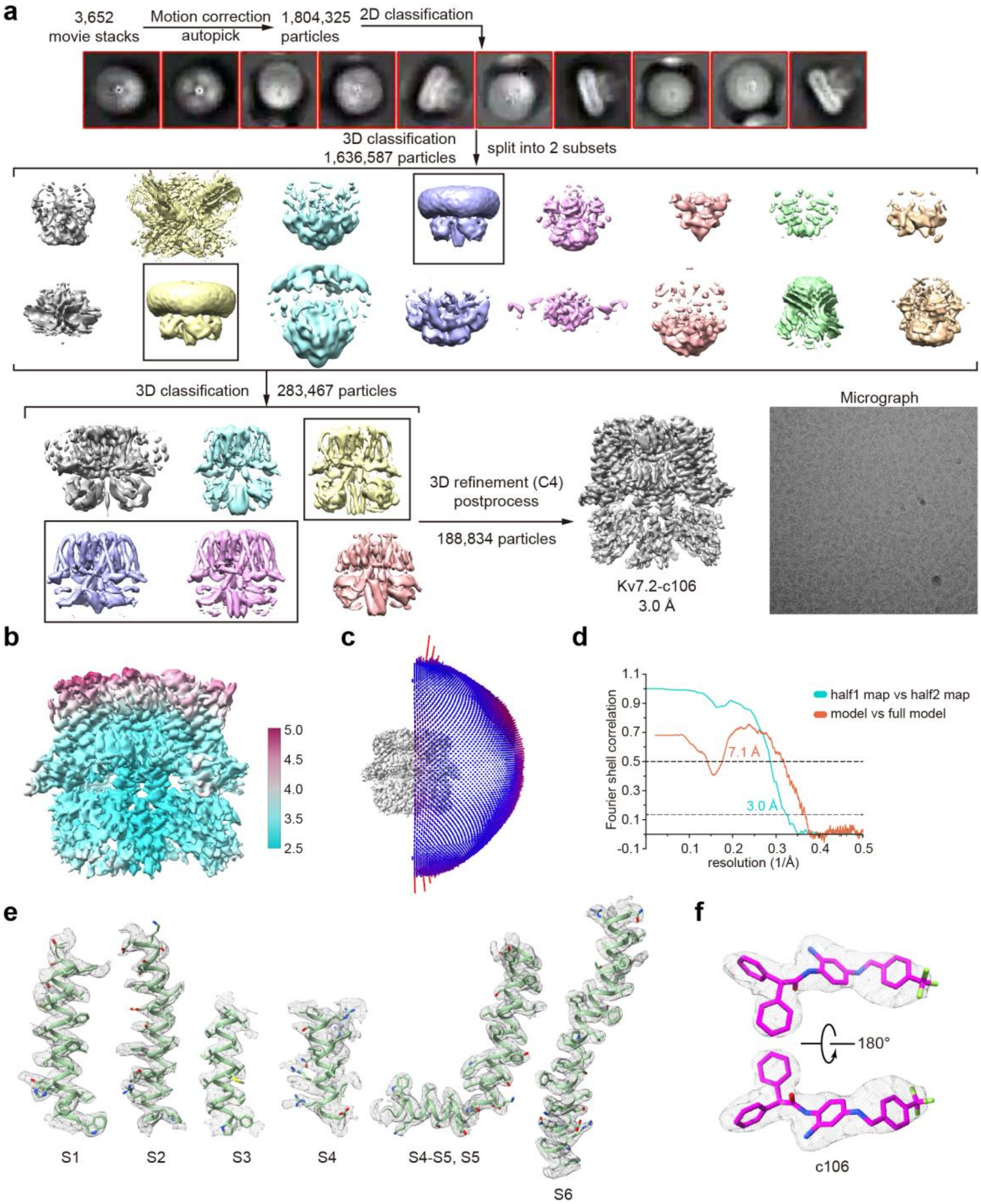

Figure S93. Structure determination of Kv7.2-c106. (**a**) Cryo-EM data processing workflow for Kv7.2-c60 particles and representative cryo-EM micrograph of Kv7.2-c106. (**b**) Local resolution analysis of the final reconstruction. (**c**) Euler angle distribution of Kv7.2-c106 particles used in the final 3D reconstruction, with the heights of the cylinders corresponding to the number of particles. (**d**) The Gold-standard Fourier shell correlation (FSC) curves for the final 3D reconstruction of Kv7.2-c106 and the cross validation between the map and the model of Kv7.2-c106. (**e**) Cryo-EM density corresponding to KV7.2 S1–S6 at the contour level of 0.008 in UCSF ChimeraX. (**f**) The density map of **c106** molecule in different orientations at the contour level of 0.008 in UCSF ChimeraX.

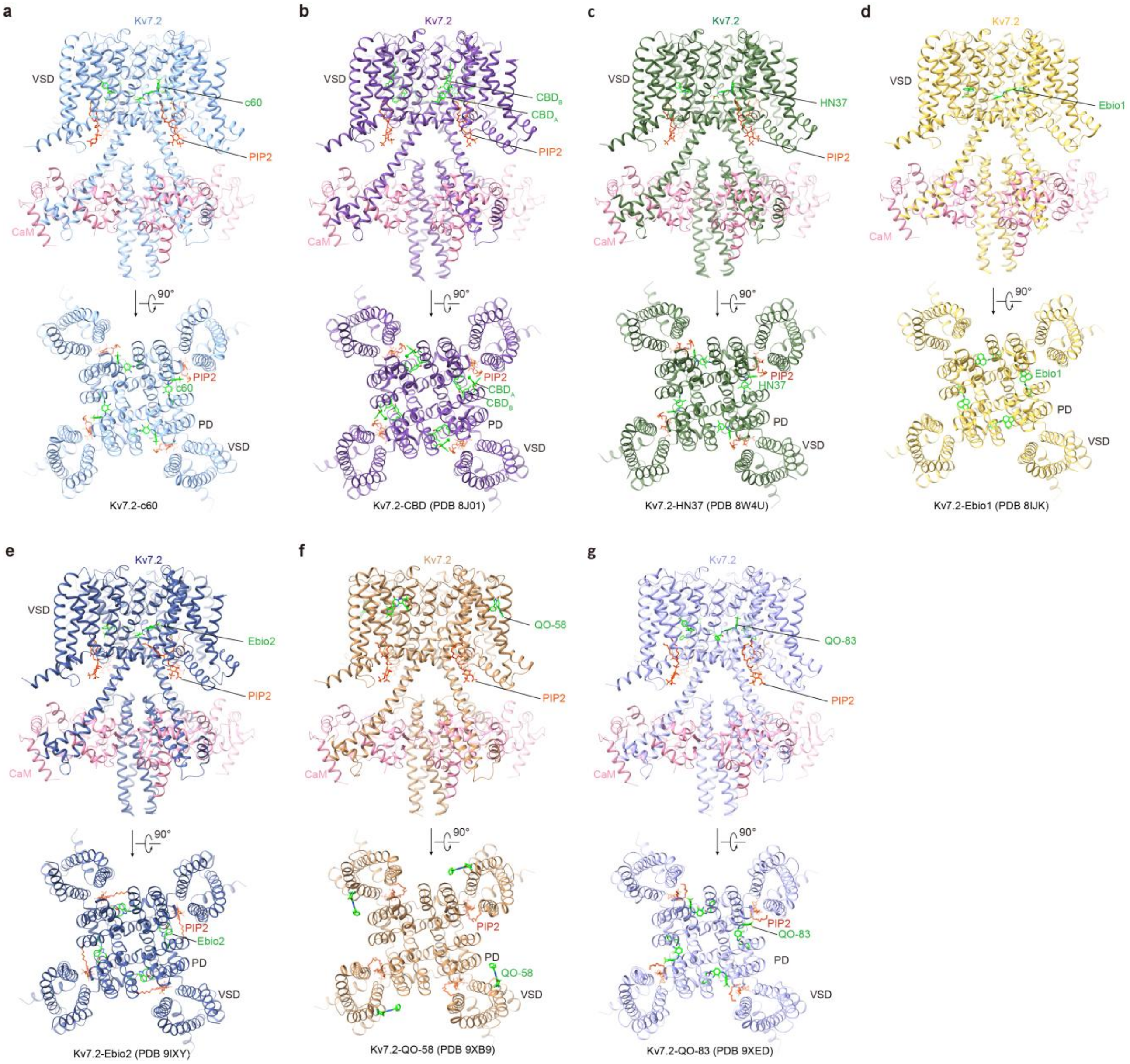

Figure S94. Comparisons of the Kv7.2-c60 structure with related Kv7.2 structures. (**a–g**) Side views of the whole channel (up) and top views (bottom) of open-state Kv7.2-CaM structures bound to the activators CBD, HN37, eBio1, eBio2, QO58, and QO-83.

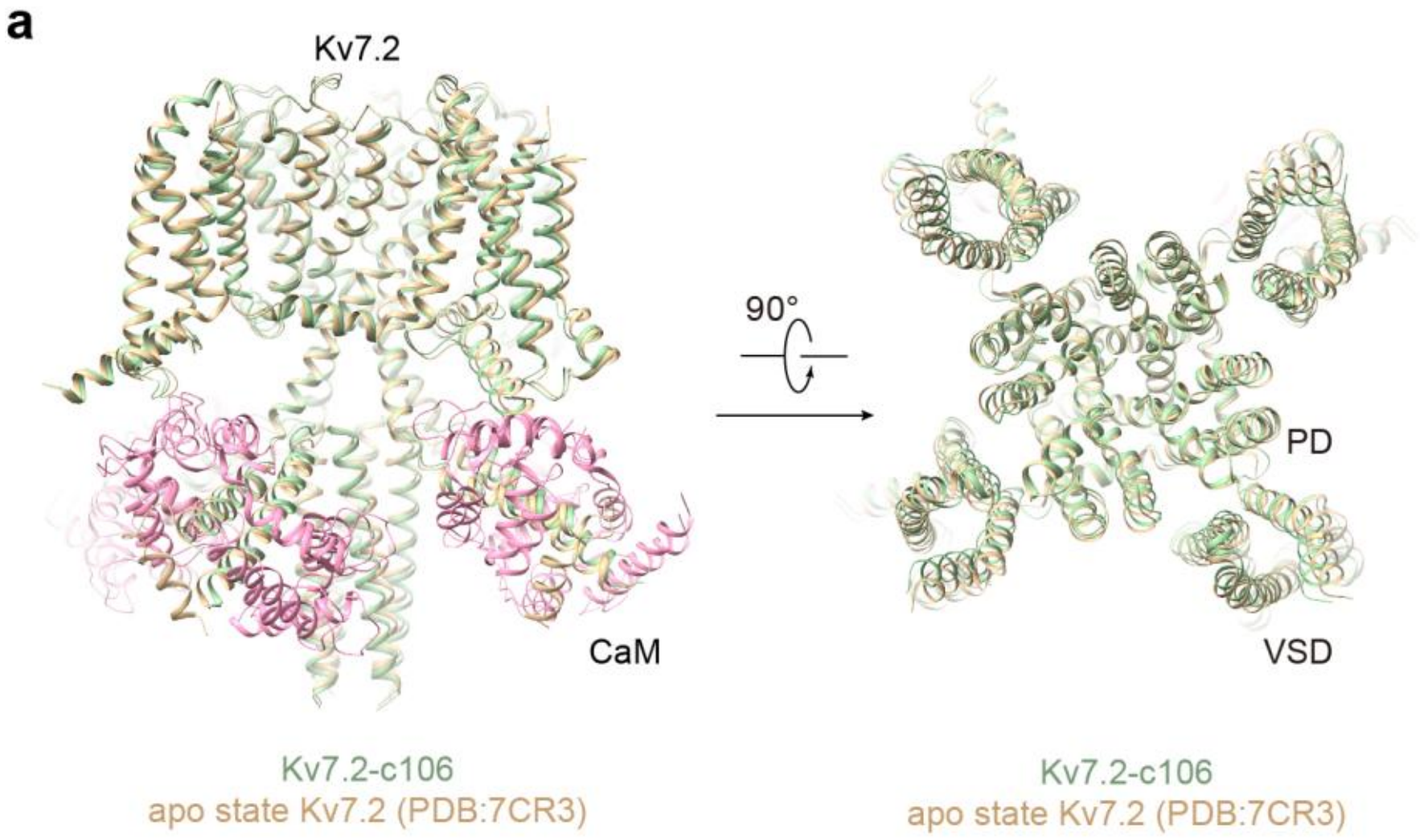

Figure S95. Structural comparison of Kv7.2-c106 (green) and Kv7.2 in apo state (tan) in side and top views. CaM is shown in pink.
